# Effectiveness of robot-assisted task-oriented training intervention for upper limb and daily living skills in stroke patients: A meta-analysis

**DOI:** 10.1371/journal.pone.0316633

**Published:** 2025-01-03

**Authors:** Chengzhu Jin, Yonghuan Chen, Yuanyuan Ma

**Affiliations:** 1 Physical education College, Yanbian University, Yanji, China; 2 Department of Marine Sports, Pukyong National University, Busan, South Korea; 3 Department of Physical Education, Beijing Wuzi University, Beijing, China; University of Naples Federico II: Universita degli Studi di Napoli Federico II, ITALY

## Abstract

**Purpose:**

Stroke is one of the leading causes of acquired disability in adults in high-income countries. This study aims to determine the intervention effects of robot-assisted task-oriented training on enhancing the upper limb function and daily living skills of stroke patients.

**Methods:**

A systematic search was conducted across PubMed, China National Knowledge Infrastructure, Web of Science, Cochrane Library, Embase, and Scopus databases through March 1, 2024. This process yielded 1,649 articles, from which 15 studies with 574 samples met the inclusion criteria for analysis. The quality of the included studies was evaluated using the Cochrane Risk of Bias tool. We performed meta-analyses, subgroup analyses, regression analyses, and sensitivity analyses using Review Manager 5.4 and Stata 17.0. Furthermore, publication bias was assessed using Begg’s and Egger’s tests. This study is registered with PROSPERO (No. CRD42024513483).

**Results:**

A random effects model was utilized. The results indicated that robot-assisted task-oriented training significantly improved Fugl-Meyer Assessment-Upper Extremity scores compared to the control group [SMD = 1.01, 95% CI (0.57, 1.45)]. Similarly, robot-assisted task-oriented training demonstrated a significant effect on the Modified Barthel Index scores [SMD = 0.61, 95% CI (0.41, 0.82)]. Subgroup and regression analyses revealed that the use of combined interventions, the geographical region of the first author, and the age of the subjects did not appear to be sources of high heterogeneity. Publication bias tests using the FMA-UE as an outcome measure yielded Begg’s test (p = 0.76) and Egger’s test (p = 0.93), suggesting no significant publication bias. Sensitivity analyses confirmed the robustness of the study findings.

**Conclusions:**

Robot-assisted task-oriented training significantly enhances the rehabilitation of upper limb function and the recovery of daily living skills in stroke patients.

## Introduction

"Stroke," also referred to as "cerebral stroke" or "cerebrovascular accident," is an acute cerebrovascular condition noted for its high rates of morbidity, mortality, disability, and recurrence [1]. Individuals who survive the acute phase of stroke often face functional impairments, including motor, speech, and perceptual deficits. Approximately 60–80% of stroke patients can regain the ability to walk following treatment [2]. Nonetheless, over 65% of patients continue to experience upper limb dysfunction six months post-onset, which significantly impacts their ability to perform daily living activities and diminishes their quality of life [3]. Recovery of upper limb function is particularly challenging and tends to be less effective and slower compared to lower limb recovery, making it a focal point and challenge in stroke rehabilitation [4]. Currently, rehabilitation that relies on manually assisted exercise training by physiotherapists faces several limitations, including a singular training approach, lack of engagement, and suboptimal patient adherence [5]. Additionally, quantifying the intensity of the training poses a challenge [6]. As rehabilitation therapy technology advances, robot-assisted rehabilitation has emerged as an innovative treatment approach within rehabilitation training. This method has proven effective in enhancing the limb motor function of stroke patients and has demonstrated tangible clinical benefits [7]. The use of robotic assistance in stroke rehabilitation offers several advantages over traditional manual assistance, including:1. Robots are capable of delivering high-precision repetitive movements, which can enhance the efficiency of the rehabilitation process. 2. They can offer therapists a range of practice strategies to tailor to individual patient needs. 3. The real-time human-computer interaction facilitated by robots allows for precise monitoring of the patient’s limb status, leading to more objective assessments and enabling adjustments to the intervention strategy through control parameter modifications [8]. Task-oriented training is a key functionality of rehabilitation robots and is regarded as a primary and highly effective method for functional rehabilitation of the upper limb [9]. This training approach, which focuses on tasks relevant to daily activities, has demonstrated superior rehabilitation outcomes when compared to traditional exercises that involve passive movements and are limited to the range of motion of affected joints [10].

Evidence to date suggests that robot-assisted task-oriented training can enhance upper limb function and daily living skills in stroke patients [11–25]. Nonetheless, the small sample sizes across studies, heterogeneity in study designs, and varying quality of the literature have resulted in inconsistent findings. This variability poses challenges in determining whether robot-assisted task-oriented training is more effective than traditional rehabilitation methods, task-oriented training alone, or the use of rehabilitation robots without human intervention. To address these uncertainties, the present study employs a meta-analytic approach to systematically review randomized controlled trials that satisfy the predefined inclusion criteria. The aim is to elucidate the intervention effects of robot-assisted task-oriented training on the upper limb function and daily living skills of stroke patients.

## Materials and methods

### Systematic review protocol registration

This study is registered with PROSPERO (No. CRD42024513483).

Inclusion and exclusion criteria for the literature

This study established literature screening criteria in accordance with the PICOS framework.

#### Inclusion criteria

1. Population (P): Participants were individuals diagnosed with stroke. 2. Intervention (I): The intervention included robot-assisted task-oriented training, without restrictions on the types of robots, training durations, intensities, or frequencies.3. Comparison (C): The control interventions comprised conventional therapies such as physiotherapy (functional training, manipulative therapy, neuromuscular electrical stimulation, etc.) and operational therapies (training in activities of daily living such as dressing, eating, grooming, and training in object manipulation). 4. Outcome (O): The primary outcome measures were the Fugl-Meyer Assessment-Upper Extremity (FMA-UE) and the Modified Barthel Index (MBI). 5. Study Design (S): Only randomized controlled trials (RCTs) were considered eligible for inclusion.

#### Exclusion criteria

1. Studies with incomplete original data, from which data could not be extracted, and for which no response was received after attempting to contact the authors. 2. Studies incorporating conventional interventions other than physical therapy and occupational therapy. 3. Literature not published in Chinese or English. 4. Conference abstracts and secondary research literature. 5. Duplicate publications of the same study identified in various databases. 6. Studies lacking the required outcome indicators.

### Literature retrieval strategy

A literature search was conducted in PubMed, China National Knowledge Infrastructure(CNKI), Web of Science(WOS), Cochrane Library, Embase, and Scopus databases. The search encompassed the period from the inception of each database up to March 1, 2024, utilizing both subject terms and free text terms. The search terms comprised "stroke," "cerebral stroke," "cerebrovascular accident," "hemiplegia," "cerebrovascular apoplexy," "robotics," "task-oriented training," and similar phrases. We also searched the GreyNet international, Opengrey, and OpenDoar databases. However, no literature was found that met the inclusion criteria.

### Literature screening and data extraction

Initially, two researchers independently screened the literature against the predefined inclusion and exclusion criteria and extracted pertinent information. After the extraction process was finalized, the two researchers cross-verified the collected data. In cases of disagreement, a third researcher was involved to facilitate a discussion and render a final decision. The extracted information encompassed the following details: 1. The first author’s name and affiliation, along with the publication date. 2. Demographic information of the participants, including sample size, age, and gender distribution. 3. The specifics of the conventional interventions administered to the control group and the interventions implemented in the experimental group. 4. The duration of the intervention period. 5. The outcome measures and the corresponding results used to evaluate the patients’ physical status.

### Literature quality assessment

This study utilized the Cochrane Risk-of-Bias Tool for Randomized Controlled Trials to assess the risk of bias and the quality of the included literature. The assessment criteria encompassed the following domains: Selection bias, Performance bias, Attrition bias, Detection bias, Reporting bias, and Other biases. The risk of bias for each included study was categorized as follows: "+" indicated a low risk of bias, and the presence of "?" denoted an unclear bias.

### Statistical analysis

Data were analyzed using Review Manager 5.4 and Stata 17.0. The risk of bias for the included studies was assessed using the Cochrane Risk of Bias Tool. Given that the outcome indicators in the study were continuous variables, the effect sizes were calculated using the standard mean difference (SMD) and accompanied by 95% confidence intervals (CI), with 0 set as the invalid threshold. Heterogeneity among studies was quantified using the I^2^ statistic, with a fixed-effects model applied for low heterogeneity (p ≥ 0.1, I^2^ ≤ 50%) and a random-effects model for high heterogeneity (p < 0.1, I^2^ > 50%). In cases of significant heterogeneity, subgroup analyses and meta-regression were employed to investigate potential sources. When the origins of heterogeneity could not be ascertained, a narrative synthesis was conducted. Publication bias was assessed using Egger’s test and Begg’s test, with a p-value greater than 0.05 suggesting no significant publication bias. Sensitivity analyses were conducted to evaluate the robustness of the findings.

## Result

### Basic features of the included literature

**Fig 1:**
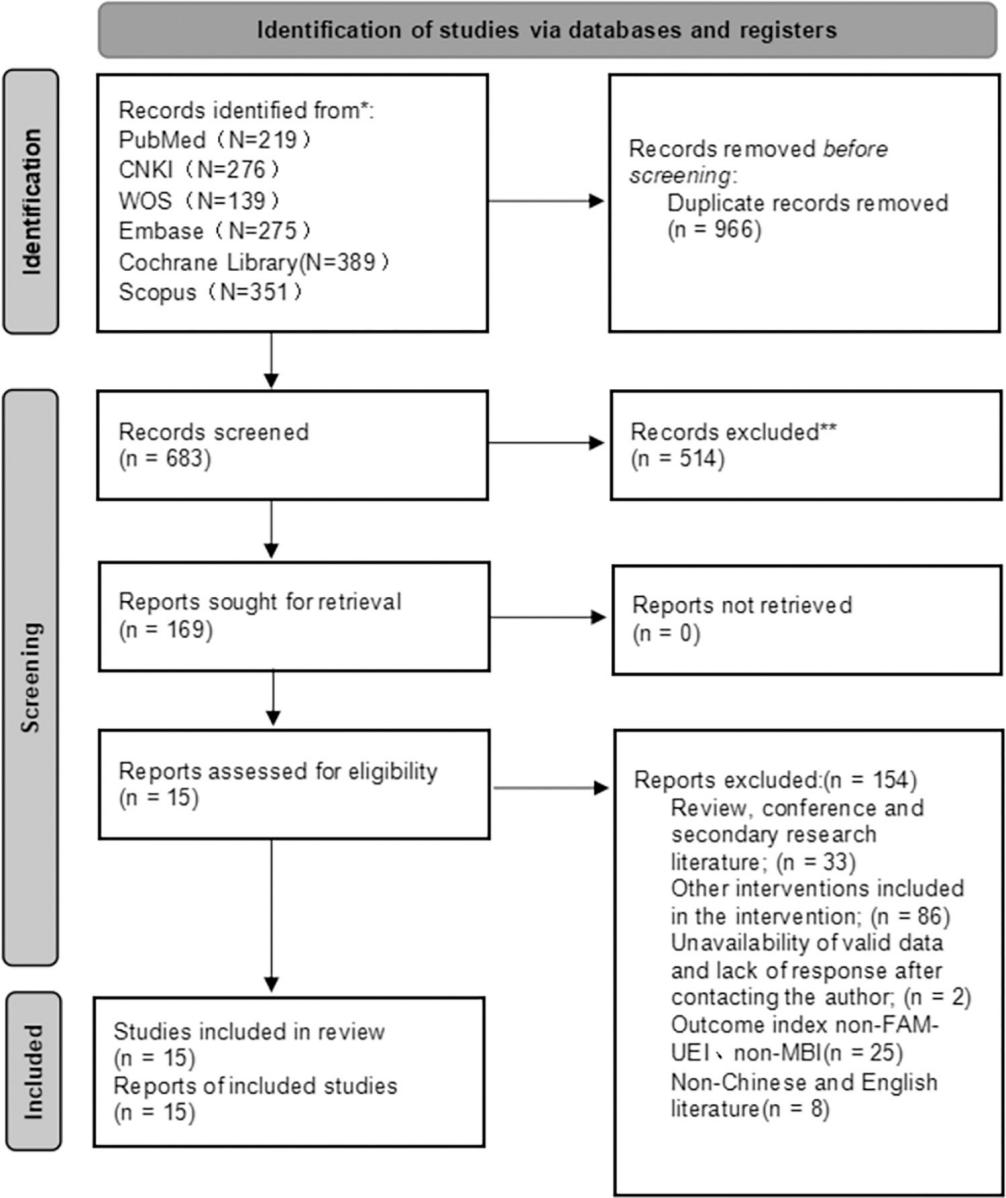
Literature screening process.

After the search, a total of 1,649 relevant articles were identified from the following databases: PubMed (219), CNKI (276), WOS (139), Cochrane Library (389), Embase (275), and Scopus (351). These articles were screened based on the study’s inclusion and exclusion criteria, resulting in 15 articles that met the study’s requirements. Additionally, a search of gray literature was conducted in databases such as GreyNet International, OpenGrey, and OpenDOAR, but no additional eligible articles were found. The literature screening process is depicted in (Fig 1). The study population consisted of stroke patients, with a total sample size of 574 participants, evenly distributed between the experimental and control groups at 287 each. Among the included articles, 13 utilized the FMA-UE as an outcome measure, while 9 used the MBI. The basic information of the included studies is presented in Table 1.

**Table 1:** Basic information of the included literature.

First author and date of publication	Area	Sample size (C/T)	Gender M/F	Ages (C/T)	Interventions (C/T)	Duration(Week)	Outcome indicators
Sun Ya 2023 [11]	China	26/26	33/19	58.23±9.61/ 56.15±8.01	CT/ CT+RATOT	6	①②
Gong Shunzhi 2023 [12]	China	32/32	35/29	63.18±3.21/ 63.24±3.35	CT/ CT+RATOT	6	①
Du Binhong 2022 [13]	China	30/30	37/23	58.33±11.90/ 60.77±11.27	CT/ CT+RATOT	4	①②
Su Lili 2022 [14]	China	30/30	29/31	65.53±5.46/ 64.97±2.26	CT/ CT+RATOT	4	②
Lei Yufeng 2021 [15]	China	47/47	53/41	61.03±7.36/ 60.82±7.54	CT/ RATOT	8	①
Fan Hong 2020 [16]	China	30/31	38/23	68.40±10.68/ 65.74±6.73	CT/ CT+RATOT	4	②
Ye Zhengmao 2019 [17]	China	18/17	24/11	60.11±5.64/ 62.06±7.39	CT/ RATOT	2	①②
Gao Hongliang 2023 [18]	China	18/17	22/13	52.33±11.44/ 53.11±11.43	CT/ CT+RATOT	4	①②
Pang Wenjun 2015 [19]	China	17/17	22/12	70.00±6.75/ 67.41±7.70	CT/ CT+RATOT	6	①②
Fu Zhen 2017 [20]	China	14/16	21/9	65.50±3.11/ 62.69±3.20	CT/ CT+RATOT	2	①②
Yang Qiang 2019 [21]	China	35/35	43/27	67.04±4.21/ 66.39±4.18	CT/ CT+RATOT	2	①
HE You-Ze 2023 [22]	China	16/16	22/10	56.75±9.54/ 58.38±10.81	CT/ CT+RATOT	4	①②
Yu-wei Hsieh 2017 [23]	China	15/16	18/13	52.87±10.40/ 49.28±10.90	CT/ CT+RATOT	4	①
Alexa B. Keeling 2021 [24]	Canada	10/9	17/2	57.70±15.10/ 54.33±15.60	CT/ RATOT	2	①
Gloria Perini 2021 [25]	Italy	9/9	9/9	61.4±9/ 58.7±20.6	CT/ RATOT	4	①

aC: Control group; T: Experimental group; M: Male; F: Female; CT: (Conventional therapy); RATOT: (Robot-assisted task-oriented training); ①:FMA-UE; ②: MBI

### Risk assessment for inclusion in the literature

**Fig 2:**
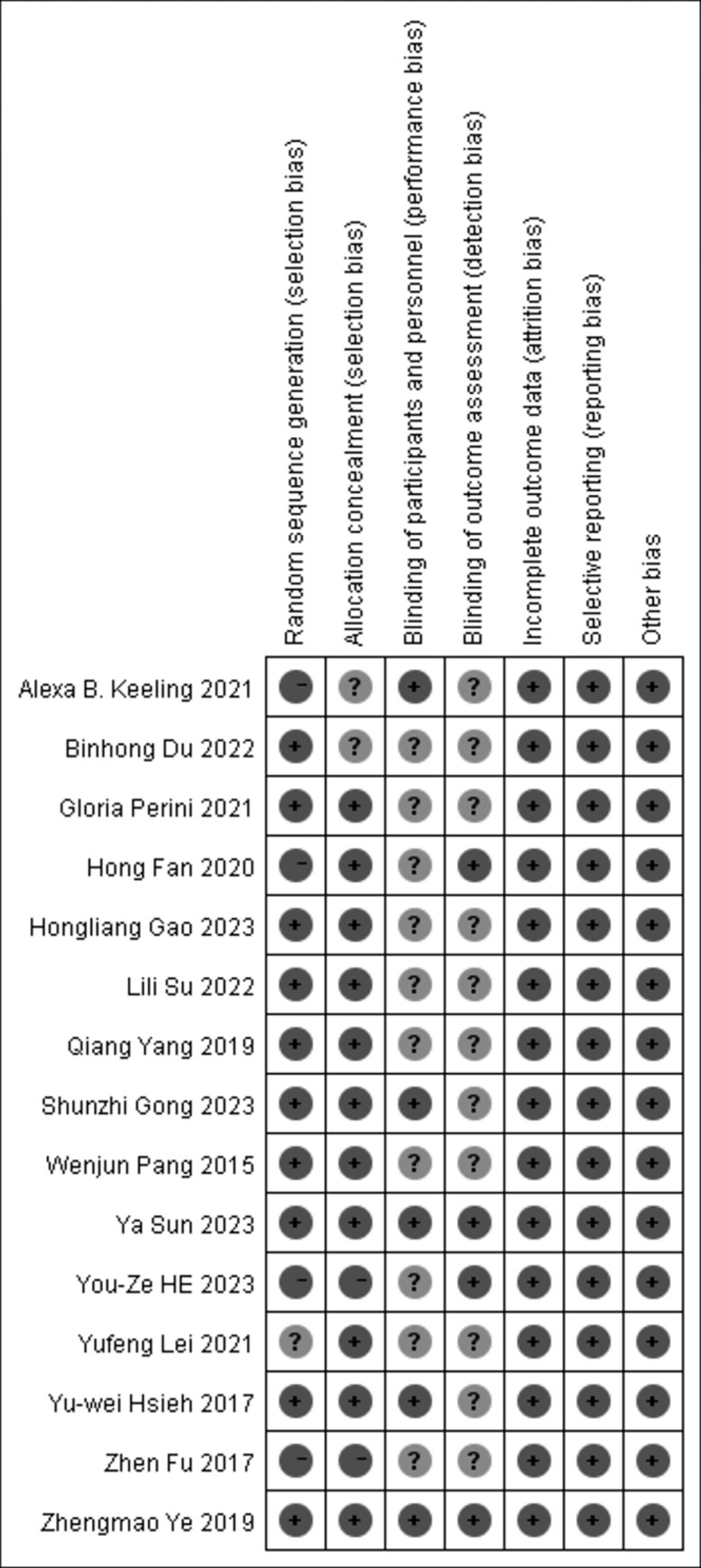
Risk of bias assessment.

The risk of bias assessment results are presented in (Fig 2). Among the 15 papers included in this study, 13 specified the use of random grouping for subjects; 14 described allocation concealment; 14 implemented blinding of subjects or intervention implementers; and 5 ensured blinding during outcome measurement and analysis. All included papers provided complete and non-selective reporting of results. However, the risk of bias for other domains was unclear for all 15 papers.

### Meta-analysis

**Fig 3:**
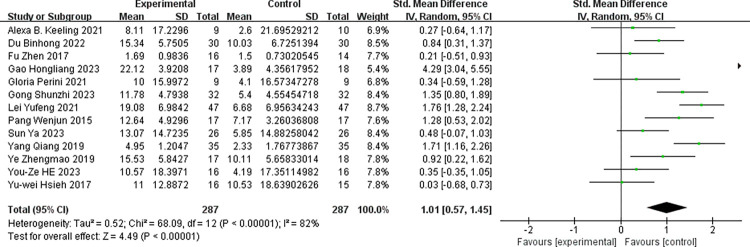
Effect of robot-assisted task-oriented training on FMA-UE.

**Fig 4:**
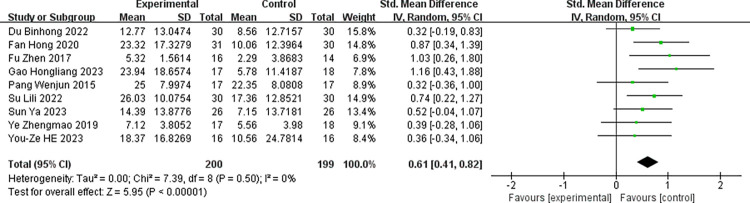
Effect of robot-assisted task-oriented training on MBI.

To assess the impact of robot-assisted task-oriented training on various outcome indicators, the studies featuring these different indicators were analyzed individually. Within the included literature, the FMA-UE served as an outcome measure in 13 articles, while the MBI was used in 9 articles. As depicted in (Fig 3), the experimental group demonstrated a significantly greater improvement in FMA-UE scores compared to the control group [SMD = 1.01, 95% CI (0.57, 1.45)]. The included studies exhibited a substantial degree of heterogeneity (I^2^ = 82%, p<0.001), warranting the use of a random-effects model. (Fig 4) illustrates the impact of robot-assisted task-oriented training on MBI scores, revealing that the experimental group had a significantly more pronounced effect on MBI compared to the control group [SMD = 0.61, 95% CI (0.41, 0.82)], with no significant heterogeneity observed (I^2^ = 0%, p = 0.50). Given the high heterogeneity observed in the meta-analysis of FMA-UE scores, subgroup and regression analyses were employed to explore potential sources of this heterogeneity.

### Subgroup analysis

**Fig 5:**
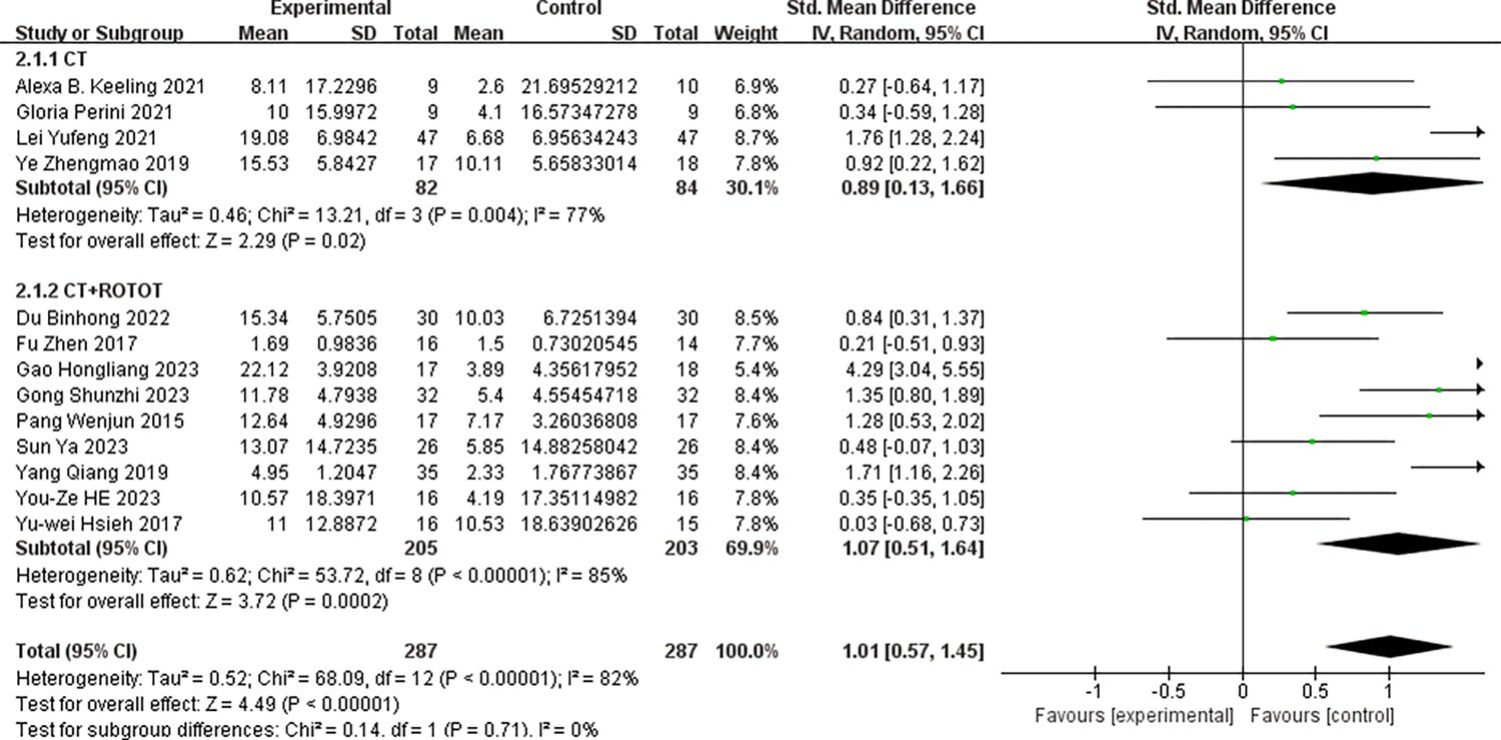
Subgroup analysis of the effect of the use of combined interventions on FMA-UE scores.

**Fig 6:**
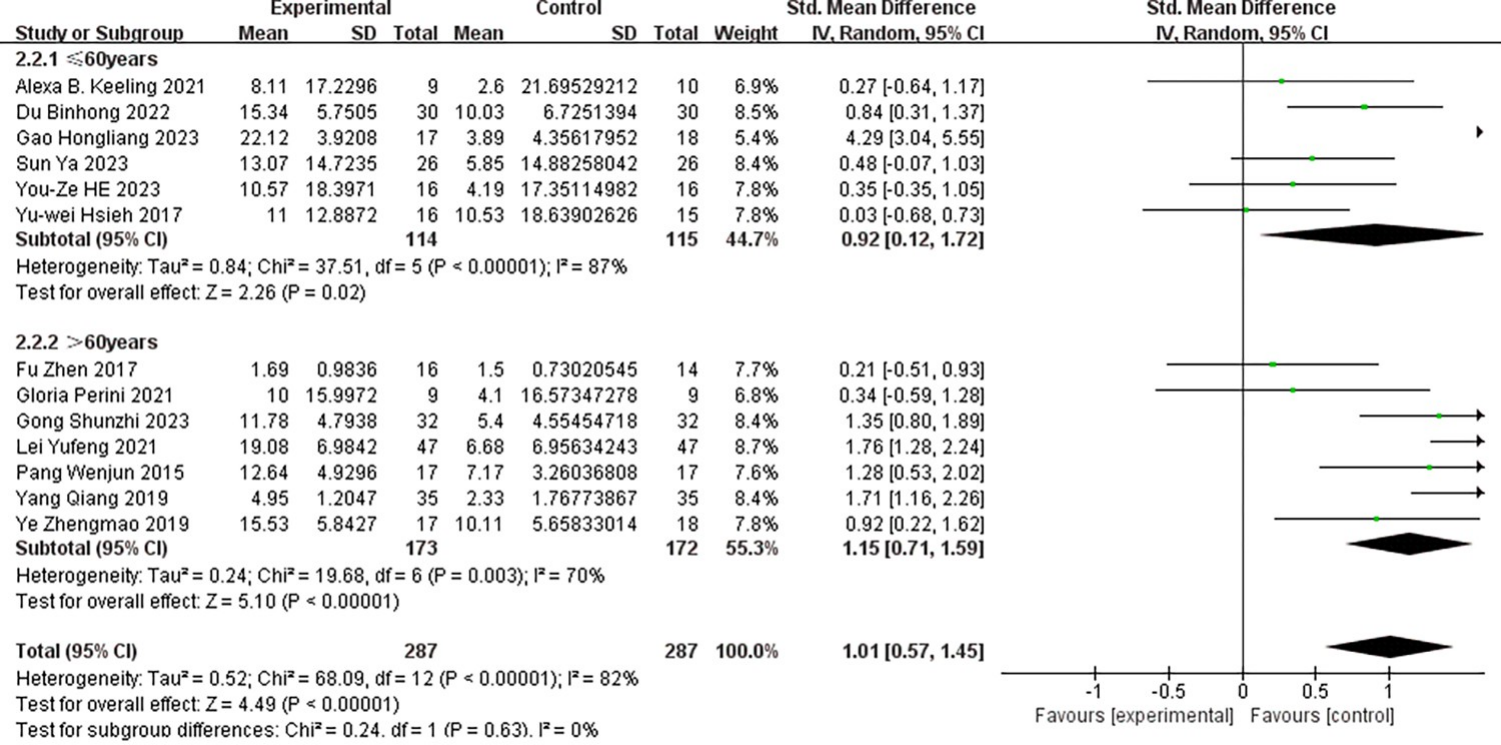
Subgroup analysis of the effect of different ages on FMA-UE scores.

**Fig 7:**
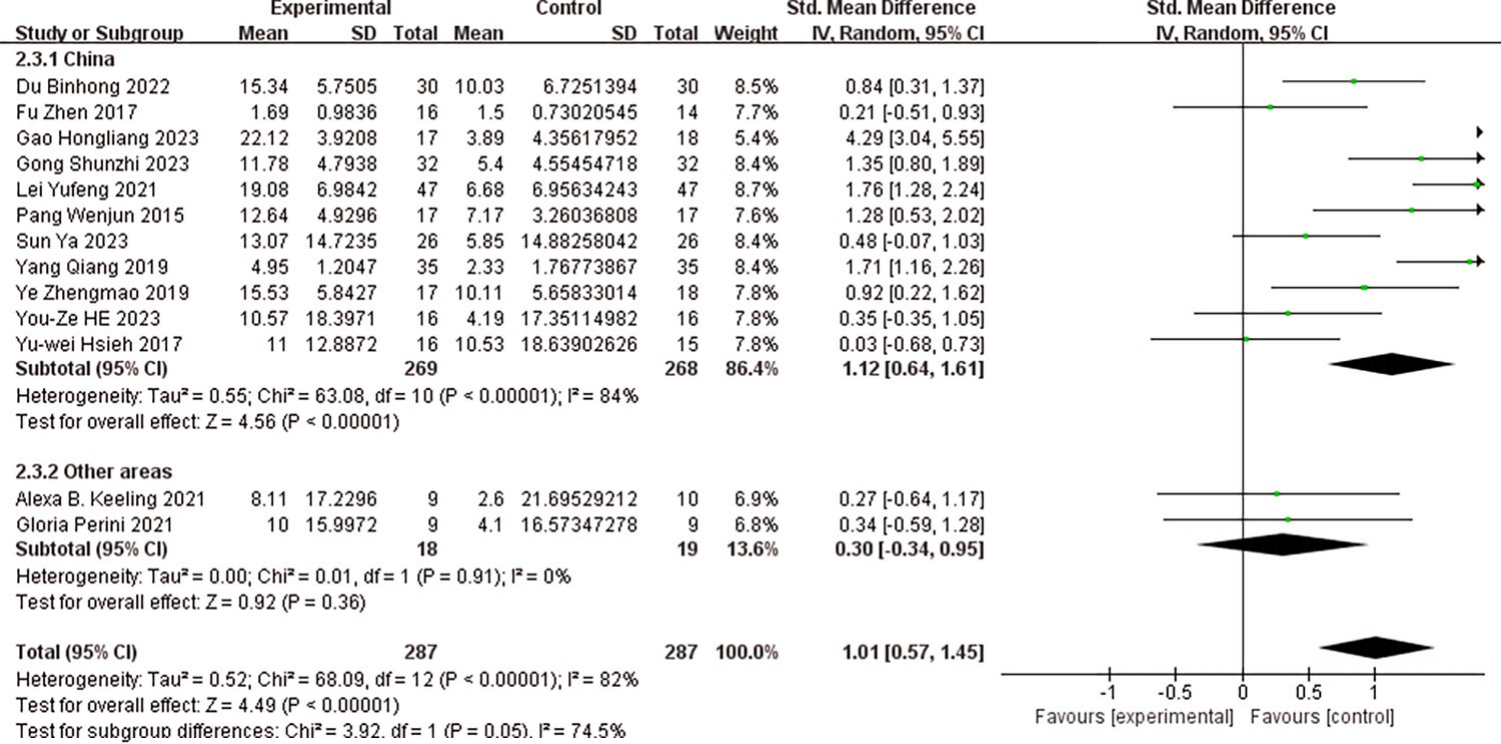
Subgroup analysis of the effect of different areas on FMA-UE scores.

Subgroup analyses were performed based on the following criteria: whether the experimental group received a combined intervention of conventional therapy (CT) and robot-assisted task-oriented training (ROTOT), the age of the subjects, and the geographical regions of the first authors. (Figs 5–7) display the results of these subgroup analyses for the FMA-UE scores. The findings indicated significant effect sizes within the subgroups, both with and without the use of combined interventions, as well as when stratified by age. However, high heterogeneity persisted across these subgroups. In the subgroup analysis examining the impact of different geographical regions on FMA-UE scores, the ’other areas’ subgroup exhibited low heterogeneity (I^2^ = 0%, p = 0.91) and a non-significant effect size [SMD = 0.30, 95% CI (-0.34, 0.95)]. Nevertheless, overall heterogeneity remained high (I^2^ = 82%, P<0.001), and the pooled effect size was significant [SMD = 1.01, 95% CI (0.57, 1.45)].

### Regression analysis

In the regression analyses, the implementation of a combined intervention, the geographical regions of the first authors, and the age of the subjects were each considered as covariates. Table 2 presents the outcomes of the regression analysis concerning the FMA-UE. The analysis yielded p-values exceeding 0.05, suggesting that these covariates did not significantly contribute to the observed high heterogeneity. Integrating the findings from both subgroup and regression analyses, it can be deduced that the use of combined interventions, the geographical regions of the studies, and the age of the participants were not driving factors of high heterogeneity in the literature utilizing FMA-UE as the primary outcome measure.

**Table 2:** Regression analysis of FMA-UE.

Covariate	Coefficient	Std. err.	p	95% CI
Interventions	0.2314	0.6154	0.707	-0.9749, 1.4376
Areas	-0.8330	0.7688	0.279	-2.3398, 0.6738
Age	0.1835	0.5609	0.744	-0.9158, 1.2828

### Publication bias and sensitivity analysis

**Fig 8:**
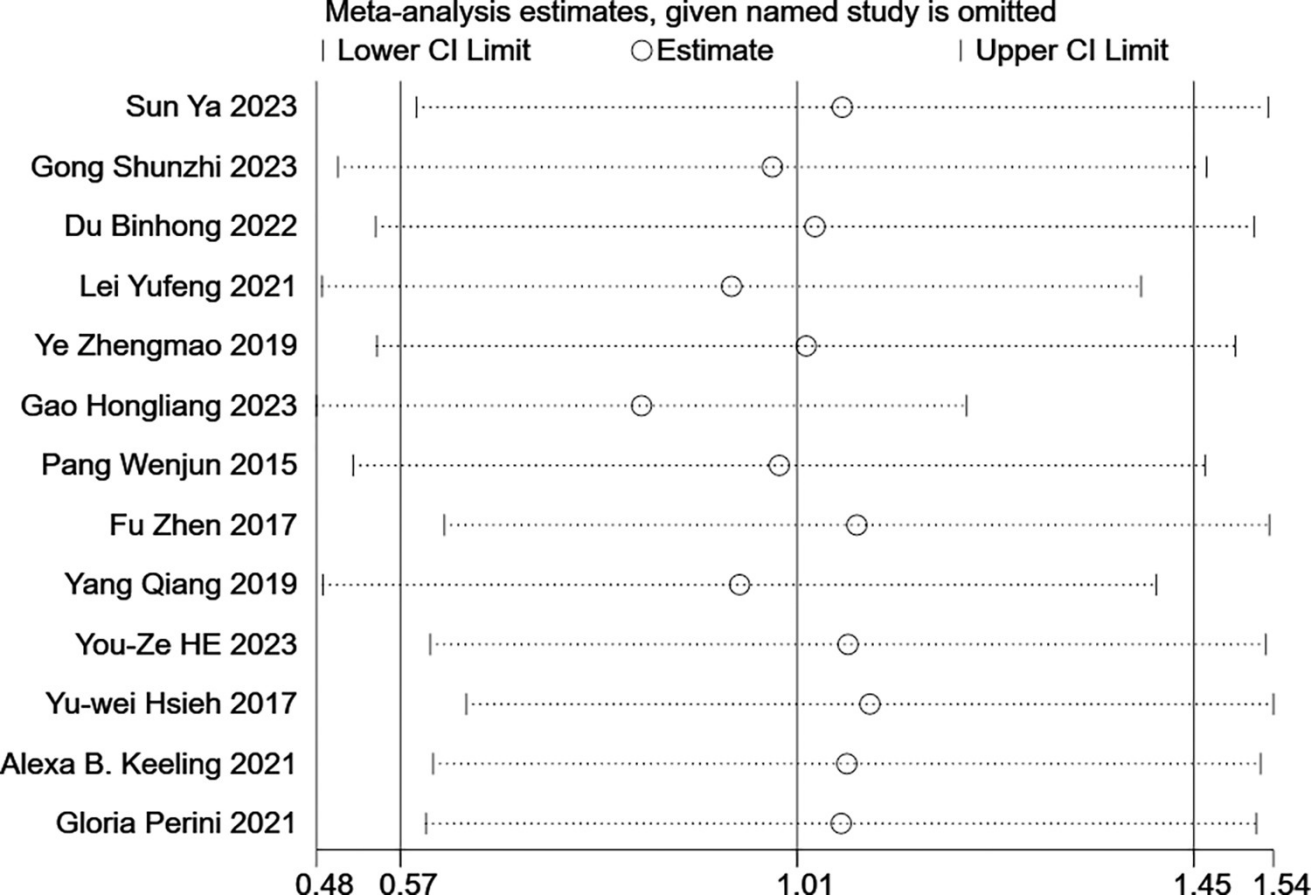
Sensitivity analysis using FMA-UE as an outcome indicator.

**Fig 9:**
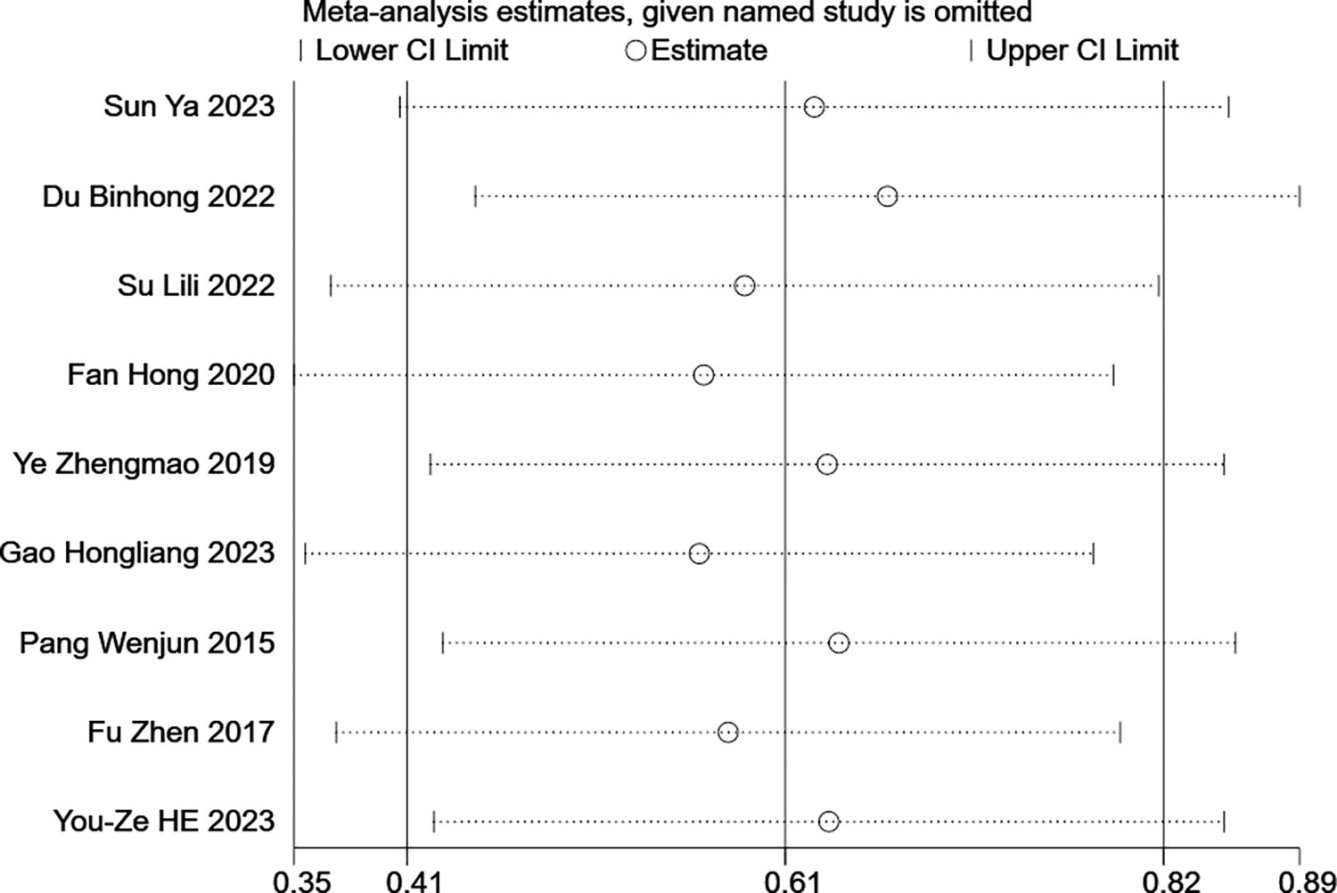
Sensitivity analysis using MBI as an outcome indicator.

In this study, publication bias analyses were conducted only on the 13 papers that included the FMA-UE as an outcome indicator. This is attributed to the fact that only 9 papers utilized the MBI as an outcome indicator, and it is generally deemed inappropriate to employ this method when the number of included studies falls below 10 [26]. The publication bias was assessed using Begg’s test (p = 0.76) and Egger’s test (p = 0.93), with both tests indicating no significant publication bias. The outcomes of the sensitivity analysis are depicted in (Figs 8 and 9). By sequentially removing each included study, the 95% confidence intervals remained relatively stable, suggesting that the findings of this study are robust.

### Discussion

The FMA-UE is a widely recognized and authoritative scale for assessing upper limb motor function in stroke patients [27]. This study’s findings on FMA-UE scores [SMD = 1.01, 95% CI (0.57, 1.45)] indicate that robot-assisted task-oriented training is significantly more efficacious than conventional therapy in improving upper limb motor function in stroke patients. Unlike traditional therapies that often involve repetitive, isolated movements, task-oriented training is designed to mimic real-life activities, providing a goal-directed approach that is particularly beneficial for stroke patients’ upper limb rehabilitation [28]. By integrating the advantages of both task-oriented training and robotic assistance, this intervention is increasingly acknowledged as an effective method for rehabilitation and training [29]. The MBI is a well-validated instrument for assessing a patient’s functional capabilities in daily living activities. However, it is important to note that the MBI primarily measures the patient’s physical functioning [30]. The meta-analysis conducted on the MBI data revealed [SMD = 0.61, 95% CI (0.41, 0.82)], suggesting that robot-assisted task-oriented training is significantly more effective than the control group in enhancing the daily living skills of stroke patients. Researchers have posited that robot-assisted task-oriented training, which incorporates motor and sensory stimulation along with social interaction, offers a diverse range of targeted training strategies [31]. This approach can facilitate the recovery of motor function by promoting neural plasticity and providing precise guidance for limb movements in patients [32].

The heterogeneity tests conducted on the included literature revealed a significant degree of variability among the studies. Consequently, subgroup and regression analyses were employed to pinpoint the potential sources of this heterogeneity. For the subgroup analyses, the following factors were examined as independent groupings: "performance of the combined conventional therapy and robot-assisted task-oriented training," "geographical region of the first author," and "age of the subjects." Similarly, in the regression analyses, these same factors were considered as covariates to assess their impact on heterogeneity. The outcomes of both the subgroup and regression analyses suggested that none of these variables were drivers of the observed high heterogeneity.

The motor relearning theory posits that the recovery of motor function in patients with neurological damage entails a relearning process, where the patient reacquires motor skills. This theory integrates insights from multiple disciplines and advocates for a task-oriented rehabilitation strategy that emphasizes the patient’s active involvement in their recovery process, namely, task-oriented training [33]. Task-oriented training is characterized by its flexibility in operating procedures. It situates the patient’s rehabilitation within a specific task environment, where goals are set and the difficulty of tasks is adjusted in real-time. This approach aims to provide the patient with neurological and kinesthetic feedback, ultimately leading to enhanced motor function [34]. The integration of robots in clinical practice for stroke patient rehabilitation is well-established, leveraging their unique advantages. For instance, exoskeleton robots have been utilized to aid in walking for patients with spinal cord injuries [35], while the combination of low-frequency repetitive transcranial magnetic stimulation with a rehabilitation robot has been shown to facilitate hand function recovery in stroke patients [36]. Additionally, wearable lower-limb rehabilitation robots have been applied to address posterior lower-limb mobility issues in stroke patients [37]. Scholarly research indicates that rehabilitation facilitated by rehabilitation robots has a more positive impact on patient recovery. Through meta-analyses, some researchers have compared the efficacy of robotic-assisted interventions with traditional methods for stroke patients. The findings suggest that rigid exoskeleton devices are more effective for enhancing hand control, increasing wrist muscle tone, and boosting muscle strength [38]. In contrast, soft robotic gloves have demonstrated greater efficacy in improving hand dexterity and daily living skills [39]. Researchers have investigated the impact of motor imagery-based brain-computer interface (BCI) training on the rehabilitation of hand function in stroke patients. Their findings suggest that this intervention is more effective in improving hand function and daily living activities than the control group [40]. Additionally, rehabilitation robots utilizing mirror therapy have demonstrated significant benefits for hand function recovery in stroke patients [41]. Despite these advancements, there has been a relative lack of focus on the effects of robot-assisted task-oriented training specifically on hand function and life skills in stroke patients. This gap in the literature underscores the rationale for the present study, which aims to conduct a meta-analysis examining the impact of robot-assisted task-oriented training interventions on upper limb mobility and daily living skills in stroke patients.

In summary, robot-assisted task-oriented training emerges as a superior intervention for enhancing upper limb mobility and daily living skills in stroke patients when compared to traditional therapies. Consequently, this form of training may be considered a viable intervention option. Nonetheless, this study acknowledges several limitations: 1. The included literature exhibited a significant degree of heterogeneity. Despite conducting subgroup and regression analyses, the origins of this heterogeneity remained unclear. 2. The studies incorporated in this review utilized a variety of robots with different types and models in the experimental group. This paper does not account for the potential differences in the impact of various robot models on the intervention’s effectiveness. 3. While the literature included in this study was published and fulfilled the inclusion criteria, the limited number of papers and the small cumulative sample size may have influenced the outcomes. This factor could potentially limit the generalizability of the results. 4. The included studies were at risk of bias with respect to the randomization process, allocation concealment, and blinding, among other aspects. As such, caution should be exercised when interpreting the findings. Consequently, there is a need for an increased number of well-conducted, high-quality randomized controlled trials (RCTs) in the future to bolster the credibility of the results and the conclusions drawn.

## Supporting information

S1 Checklist PRISMA 2020 checklist. (DOCX)

S1 Table List of raw analysis data. (DOCX)

S2 Table Assessment of quality of evidence using Cochrane. (DOCX)

S3 Table A numbered table of all studies identified in the literature search. (DOCX)

S1 File Search strategy. (DOCX)
