# Supplementary material for: Effectiveness of robot-assisted task-oriented training intervention for upper limb and daily living skills in stroke patients: A meta-analysis
Source: PLoS One. 2025 Jan 3;20(1):e0316633. doi: 10.1371/journal.pone.0316633 (PMC11698451; doi:10.1371/journal.pone.0316633)
Supplement: S1 Checklist — (DOCX) [file pone.0316633.s001.docx]

| **Section and Topic** | **Item #** | **Checklist item** | **Location where item is reported** |
| --- | --- | --- | --- |
| **TITLE** | | |  |
| Title | 1 | This report was identified as a meta-analysis. | 1 |
| **ABSTRACT** | | |  |
| Abstract | 2 | The list of abstracts for PRISMA 2020 has been consulted. | 2 |
| **INTRODUCTION** | | |  |
| Rationale | 3 | This is stated in the introduction. | 4 |
| Objectives | 4 | This is described in the introduction. | 5 |
| **METHODS** | | |  |
| Eligibility criteria | 5 | This is described in the Materials and methods section. | 6 |
| Information sources | 6 | Searches were performed on CNKI, WOS, Cochrane Library, EMbase, Scopus, and Pubmed databases. The search was conducted from the database establishment until March 1, 2024 Searches were conducted using a combination of subject terms and free text words. | 7 |
| Search strategy | 7 | Searches were performed on CNKI, WOS, Cochrane Library, EMbase, Scopus, and Pubmed databases. The search strategy will be uploaded as an attachment | 7 |
| Selection process | 8 | This is described in the Literature screening and data extraction section. | 7 |
| Data collection process | 9 | This is described in the Literature screening and data extraction section. | 7 |
| Data items | 10a | This is described in the Materials and methods section. | 6 |
|  | 10b | This is listed in Table 1 | 9 |
| Study risk of bias assessment | 11 | This is described in the Risk assessment for inclusion in the literature. | 11 |
| Effect measures | 12 | This is described in the Statistical analysis section. | 8 |
| Synthesis methods | 13a | This is listed in Table 1 | 9 |
|  | 13b | This is described in the Statistical analysis section. | 8 |
|  | 13c | This is described in the Statistical analysis section. | 8 |
|  | 13d | This is described in the Statistical analysis section. | 8 |
|  | 13e | If heterogeneity was large, subgroup analysis and meta-regression was used to explore sources of heterogeneity. | 8 |
|  | 13f | Sensitivity analysis showed that the study results were stable. | 14 |
| Reporting bias assessment | 14 | Egger's test and Begg's Test. | 14 |
| Certainty assessment | 15 | This is described in the Statistical analysis section. | 8 |
| **RESULTS** | | |  |
| Study selection | 16a | This is shown in Figure 1 | 9 |
|  | 16b | This is shown in Figure 1 | 9 |
| Study characteristics | 17 | This is listed in Table 1 | 9 |
| Risk of bias in studies | 18 | This is listed in Figure 2 | 11 |
| Results of individual studies | 19 | This is described in the Statistical analysis section. | 8 |
| Results of syntheses | 20a | This is described in the Risk assessment for inclusion in the literature. | 11 |
|  | 20b | This is described in the Meta-analysis section. | 11 |
|  | 20c | This is described in the Subgroup analysis. | 12 |
|  | 20d | This is described in the Publication bias and sensitivity analysis. | 14 |
| Reporting biases | 21 | This is described in the Risk assessment for inclusion in the literature. | 11 |
| Certainty of evidence | 22 | This will be described in the discussion section. | 14 |
| **DISCUSSION** | | |  |
| Discussion | 23a | This is described in the Discussion section. | 14 |
|  | 23b | This is described in the Discussion section. | 17 |
|  | 23c | This is described in the Discussion section. | 17 |
|  | 23d | This is described in the Discussion section. | 17 |
| **OTHER INFORMATION** | | |  |
| Registration and protocol | 24a | This study is registered with PROSPERO (No. CRD42024513483). | 2 |
|  | 24b | This study is registered with PROSPERO (No. CRD42024513483). | 2 |
|  | 24c | This study is registered with PROSPERO (No. CRD42024513483). | 2 |
| Support | 25 | There was no funding support for this research. | 18 |
| Competing interests | 26 | The authors declare that they have no conflict of interest. | 18 |
| Availability of data, code and other materials | 27 | All of the data included in this study are available in this paper and in supplemental documents. |  |

*From:*  Page MJ, McKenzie JE, Bossuyt PM, Boutron I, Hoffmann TC, Mulrow CD, et al. The PRISMA 2020 statement: an updated guideline for reporting systematic reviews. BMJ 2021;372:n71. doi: 10.1136/bmj.n71

For more information, visit: <http://www.prisma-statement.org/>
