# Supplementary material for: Effectiveness of robot-assisted task-oriented training intervention for upper limb and daily living skills in stroke patients: A meta-analysis
Source: PLoS One. 2025 Jan 3;20(1):e0316633. doi: 10.1371/journal.pone.0316633 (PMC11698451; doi:10.1371/journal.pone.0316633)
Supplement: S1 Table — (DOCX) [file pone.0316633.s002.docx]

**S1 List of raw analysis data.**

| FMA-UE | | | | | | | | | | | | |
| --- | --- | --- | --- | --- | --- | --- | --- | --- | --- | --- | --- | --- |
|  |  |  |  | E-Pre | | E-Post | |  | C-Pre | | C-Post | |
| Name of Data Extractors | Date | study | n1 | mean1 | sd1 | mean1 | sd1 | n2 | mean2 | sd2 | mean2 | sd2 |
| Yonghuan Chen | 2024/04/01 | Sun Ya 2023 | 26 | 24.85 | 15.18 | 37.92 | 14.22 | 26 | 23.5 | 15.04 | 29.35 | 14.72 |
| Yonghuan Chen | 2024/04/01 | Gong Shunzhi 2023 | 32 | 31.48 | 4.28 | 43.26 | 5.18 | 32 | 31.69 | 4.33 | 37.09 | 4.75 |
| Yonghuan Chen | 2024/04/01 | Du Binhong 2022 | 30 | 26.73 | 5.45 | 42.07 | 6.01 | 30 | 27.17 | 6.75 | 37.2 | 6.7 |
| Yonghuan Chen | 2024/04/01 | Lei Yufeng 2021 | 47 | 23.27 | 6.84 | 42.35 | 7.12 | 47 | 23.4 | 6.51 | 30.08 | 7.33 |
| Yonghuan Chen | 2024/04/01 | Ye Zhengmao 2019 | 17 | 18.12 | 4.31 | 33.65 | 6.65 | 18 | 19.06 | 4.82 | 29.17 | 6.23 |
| Yonghuan Chen | 2024/04/01 | Gao Hongliang 2023 | 17 | 21.7 | 4.22 | 43.82 | 3.53 | 18 | 20.77 | 4.66 | 24.66 | 3.97 |
| Yonghuan Chen | 2024/04/01 | Pang Wenjun 2015 | 17 | 44.24 | 5.25 | 56.88 | 4.53 | 17 | 43.59 | 3.1 | 50.76 | 3.4 |
| Yonghuan Chen | 2024/04/01 | Fu Zhen 2017 | 16 | 4.06 | 0.9 | 5.75 | 1.05 | 14 | 2.93 | 0.72 | 4.43 | 0.74 |
| Yonghuan Chen | 2024/04/01 | Yang Qiang 2019 | 35 | 5.41 | 1.32 | 10.36 | 1.04 | 35 | 5.36 | 1.2 | 7.69 | 2.03 |
| Yonghuan Chen | 2024/04/01 | You-Ze HE 2023 | 16 | 19.31 | 17.21 | 29.88 | 19.39 | 16 | 16.75 | 16.72 | 20.94 | 17.92 |
| Yonghuan Chen | 2024/04/01 | Yu-wei Hsieh 2017 | 16 | 26.81 | 12.13 | 37.81 | 13.53 | 15 | 29.07 | 16.12 | 39.6 | 20.41 |
| Yonghuan Chen | 2024/04/01 | Alexa B. Keeling 2021 | 9 | 32.11 | 16.47 | 40.22 | 17.9 | 10 | 32 | 21.63 | 34.6 | 21.76 |
| Yonghuan Chen | 2024/04/01 | Gloria Perini 2021 | 9 | 29.3 | 17.1 | 39.3 | 14.6 | 9 | 26.6 | 17.4 | 30.7 | 15.6 |

| MBI | | | | | | | | | | | | |
| --- | --- | --- | --- | --- | --- | --- | --- | --- | --- | --- | --- | --- |
|  |  |  |  | E-Pre | | E-Post | |  | C-Pre | | C-Post | |
| Name of Data Extractors | Date | study | n1 | mean1 | sd1 | mean1 | sd1 | n2 | mean2 | sd2 | mean2 | sd2 |
| Yonghuan Chen | 2024/04/02 | Sun Ya 2023 | 26 | 41.92 | 14.91 | 56.31 | 12.54 | 26 | 41.58 | 14.62 | 48.73 | 12.59 |
| Yonghuan Chen | 2024/04/02 | Du Binhong 2022 | 30 | 54.83 | 13.19 | 67.6 | 12.9 | 30 | 54.5 | 12.49 | 63.06 | 12.93 |
| Yonghuan Chen | 2024/04/02 | Su Lili 2022 | 30 | 47.50 | 9.88 | 73.53 | 10.26 | 30 | 45.47 | 12.21 | 62.83 | 13.41 |
| Yonghuan Chen | 2024/04/02 | Fan Hong 2020 | 31 | 32.13 | 12.34 | 55.45 | 19.81 | 30 | 28.47 | 10.73 | 38.53 | 13.57 |
| Yonghuan Chen | 2024/04/02 | Ye Zhengmao 2019 | 17 | 17.47 | 3.83 | 24.59 | 3.78 | 18 | 15.61 | 3.73 | 21.17 | 4.19 |
| Yonghuan Chen | 2024/04/02 | Gao Hongliang 2023 | 17 | 21.47 | 10.10 | 45.41 | 21.53 | 18 | 20.05 | 8.36 | 25.83 | 13.01 |
| Yonghuan Chen | 2024/04/02 | Pang Wenjun 2015 | 17 | 61.18 | 4.16 | 86.18 | 9.22 | 17 | 63.53 | 3.43 | 85.88 | 9.23 |
| Yonghuan Chen | 2024/04/02 | Fu Zhen 2017 | 16 | 21.31 | 1.37 | 26.63 | 1.7 | 14 | 19 | 3.67 | 21.29 | 4.04 |
| Yonghuan Chen | 2024/04/02 | You-Ze HE 2023 | 16 | 47.13 | 17.3 | 65.5 | 16.31 | 16 | 38.13 | 25.61 | 48.69 | 23.86 |
