# Supplementary material for: Effectiveness of robot-assisted task-oriented training intervention for upper limb and daily living skills in stroke patients: A meta-analysis
Source: PLoS One. 2025 Jan 3;20(1):e0316633. doi: 10.1371/journal.pone.0316633 (PMC11698451; doi:10.1371/journal.pone.0316633)
Supplement: S2 Table — (DOCX) [file pone.0316633.s003.docx]

**S2 Assessment of quality of evidence using Cochrane**

|  | Random sequence generation | Allocation concealment | Blinding of participants and personnel | Blinding of outcome assessment | Incomplete outcome data | Selective reporting | Other bias |
| --- | --- | --- | --- | --- | --- | --- | --- |
| Alexa B. Keeling 2021 | High risk | Unclear risk | Low risk | Unclear risk | Low risk | Low risk | Low risk |
| Du Binhong 2022 | Low risk | Unclear risk | Unclear risk | Unclear risk | Low risk | Low risk | Low risk |
| Gloria Perini 2021 | Low risk | Low risk | Unclear risk | Unclear risk | Low risk | Low risk | Low risk |
| Fan Hong 2020 | High risk | Low risk | Unclear risk | Low risk | Low risk | Low risk | Low risk |
| Gao Hongliang 2023 | Low risk | Low risk | Unclear risk | Unclear risk | Low risk | Low risk | Low risk |
| Su Lili 2022 | Low risk | Low risk | Unclear risk | Unclear risk | Low risk | Low risk | Low risk |
| Yang Qiang 2019 | Low risk | Low risk | Unclear risk | Unclear risk | Low risk | Low risk | Low risk |
| Gong Shunzhi 2023 | Low risk | Low risk | Low risk | Unclear risk | Low risk | Low risk | Low risk |
| Pang Wenjun 2015 | Low risk | Low risk | Unclear risk | Unclear risk | Low risk | Low risk | Low risk |
| Sun Ya 2023 | Low risk | Low risk | Low risk | Low risk | Low risk | Low risk | Low risk |
| You-Ze HE 2023 | High risk | High risk | Unclear risk | Low risk | Low risk | Low risk | Low risk |
| Lei Yufeng 2021 | Unclear risk | Low risk | Unclear risk | Unclear risk | Low risk | Low risk | Low risk |
| Yu-wei Hsieh 2017 | Low risk | Low risk | Low risk | Unclear risk | Low risk | Low risk | Low risk |
| Fu Zhen 2017 | High risk | High risk | Unclear risk | Unclear risk | Low risk | Low risk | Low risk |
| Ye Zhengmao 2019 | Low risk | Low risk | Low risk | Low risk | Low risk | Low risk | Low risk |
