# Supplementary material for: Effectiveness of robot-assisted task-oriented training intervention for upper limb and daily living skills in stroke patients: A meta-analysis
Source: PLoS One. 2025 Jan 3;20(1):e0316633. doi: 10.1371/journal.pone.0316633 (PMC11698451; doi:10.1371/journal.pone.0316633)
Supplement: S3 Table — (DOCX) [file pone.0316633.s004.docx]

**S3 Table.** A numbered table of all studies identified in the literature search.

1. Madhoun, H. Y., Tan, B., Feng, Y., Zhou, Y., Zhou, C., & Yu, L. (2020). Task-based mirror therapy enhances the upper limb motor function in subacute stroke patients: a randomized control trial. European journal of physical and rehabilitation medicine, 56(3), 265–271. <https://doi.org/10.23736/S1973-9087.20.06070-0> （The reason for exclusion: Non robot-assisted task-oriented training）
2. Ji EK, Wang HH, Jung SJ, et al. Graded motor imagery training as a home exercise program for upper limb motor function in patients with chronic stroke: A randomized controlled trial. Medicine (Baltimore). 2021;100(3):e24351. doi:10.1097/MD.0000000000024351 （The reason for exclusion: Non robot-assisted task-oriented training）
3. Ranzani R, Lambercy O, Metzger JC, et al. Neurocognitive robot-assisted rehabilitation of hand function: a randomized control trial on motor recovery in subacute stroke. J Neuroeng Rehabil. 2020;17(1):115. Published 2020 Aug 24. doi:10.1186/s12984-020-00746-7 （The reason for exclusion: duplicate record）
4. Lee HC, Kuo FL, Lin YN, Liou TH, Lin JC, Huang SW. Effects of Robot-Assisted Rehabilitation on Hand Function of People With Stroke: A Randomized, Crossover-Controlled, Assessor-Blinded Study. Am J Occup Ther. 2021;75(1):7501205020p1-7501205020p11. doi:10.5014/ajot.2021.038232 （The reason for exclusion: duplicate record）
5. Villafañe JH, Taveggia G, Galeri S, et al. Efficacy of Short-Term Robot-Assisted Rehabilitation in Patients With Hand Paralysis After Stroke: A Randomized Clinical Trial. Hand (N Y). 2018;13(1):95-102. doi:10.1177/1558944717692096 （The reason for exclusion: duplicate record）
6. Du J, Wang S, Cheng Y, et al. Effects of Neuromuscular Electrical Stimulation Combined with Repetitive Transcranial Magnetic Stimulation on Upper Limb Motor Function Rehabilitation in Stroke Patients with Hemiplegia [retracted in: Comput Math Methods Med. 2023 Nov 1;2023:9767295. doi: 10.1155/2023/9767295]. Comput Math Methods Med. 2022;2022:9455428. Published 2022 Jan 4. doi:10.1155/2022/9455428 （The reason for exclusion: Non robot-assisted task-oriented training）
7. Huang YC, Chen PC, Tso HH, Yang YC, Ho TL, Leong CP. Effects of kinesio taping on hemiplegic hand in patients with upper limb post-stroke spasticity: a randomized controlled pilot study. Eur J Phys Rehabil Med. 2019;55(5):551-557. doi:10.23736/S1973-9087.19.05684-3 （The reason for exclusion: Non robot-assisted task-oriented training）
8. Aguilera-Rubio Á, Alguacil-Diego IM, Mallo-López A, Jardón Huete A, Oña ED, Cuesta-Gómez A. Use of low-cost virtual reality in the treatment of the upper extremity in chronic stroke: a randomized clinical trial. J Neuroeng Rehabil. 2024;21(1):12. Published 2024 Jan 22. doi:10.1186/s12984-024-01303-2 （The reason for exclusion: Non robot-assisted task-oriented training）
9. Tang C, Zhou T, Zhang Y, et al. Bilateral upper limb robot-assisted rehabilitation improves upper limb motor function in stroke patients: a study based on quantitative EEG. Eur J Med Res. 2023;28(1):603. Published 2023 Dec 19. doi:10.1186/s40001-023-01565-x （The reason for exclusion: duplicate record）
10. Heineman JT, Forster GL, Stephens KL, Cottler PS, Timko MP, DeGeorge BR Jr. A Randomized Controlled Trial of Topical Cannabidiol for the Treatment of Thumb Basal Joint Arthritis. J Hand Surg Am. 2022;47(7):611-620. doi:10.1016/j.jhsa.2022.03.002 （The reason for exclusion: Non robot-assisted task-oriented training）
11. Xia, X., Dong, X., Huo, H., Zhang, Y., Song, J., & Wang, D. (2023). Clinical study of low-frequency acupoint electrical stimulation to improve thumb-to-finger movements after stroke: A randomized controlled trial. *Medicine*, *102*(47), e35755. <https://doi.org/10.1097/MD.0000000000035755> （The reason for exclusion: Non robot-assisted task-oriented training）
12. Vanoglio, F., Comini, L., Gaiani, M., Bonometti, G. P., Luisa, A., & Bernocchi, P. (2024). A Sensor-Based Upper Limb Treatment in Hemiplegic Patients: Results from a Randomized Pilot Study. *Sensors (Basel, Switzerland)*, *24*(8), 2574. <https://doi.org/10.3390/s24082574> （The reason for exclusion: Non robot-assisted task-oriented training）
13. Gentilucci M, Negrotti A, Gangitano M. Planning an action. Exp Brain Res. 1997;115(1):116-128. doi:10.1007/pl00005671Gentilucci M, Negrotti A, Gangitano M. Planning an action. Exp Brain Res. 1997;115(1):116-128. doi:10.1007/pl00005671 （The reason for exclusion: Non-RCTs）
14. Giuffre, A., Zewdie, E., Carlson, H. L., Wrightson, J. G., Kuo, H. C., Cole, L., & Kirton, A. (2021). Robotic transcranial magnetic stimulation motor maps and hand function in adolescents. *Physiological reports*, *9*(7), e14801. <https://doi.org/10.14814/phy2.14801> （The reason for exclusion: Irrelevant study outcome）
15. de Jong, T. R., Bonhof-Jansen, E. E. D. J., Brink, S. M., de Wildt, R. P., van Uchelen, J. H., & Werker, P. M. N. (2023). Total joint arthroplasty versus trapeziectomy in the treatment of trapeziometacarpal joint arthritis: a randomized controlled trial. The Journal of hand surgery, European volume, 48(9), 884–894. https://doi.org/10.1177/17531934231185245（The reason for exclusion: Non robot-assisted task-oriented training）
16. Barnard, A., Jansen, V., Swindells, M. G., Arundell, M., & Burke, F. D. (2020). A randomized controlled trial of real versus sham acupuncture for basal thumb joint arthritis. *The Journal of hand surgery, European volume*, *45*(5), 488–494. https://doi.org/10.1177/1753193420911326（The reason for exclusion: Non robot-assisted task-oriented training）
17. Klim, S. M., Glehr, R., Graef, A., Amerstorfer, F., Leithner, A., & Glehr, M. (2023). Total joint arthroplasty versus resection-interposition arthroplasty for thumb carpometacarpal arthritis: a randomized controlled trial. *Acta orthopaedica*, *94*, 224–229. https://doi.org/10.2340/17453674.2023.11919（The reason for exclusion: Non robot-assisted task-oriented training）
18. van Ravestyn, C., Gerardin, E., Térémetz, M., Hamdoun, S., Baron, J. C., Calvet, D., Vandermeeren, Y., Turc, G., Maier, M. A., Rosso, C., Mas, J. L., Dupin, L., & Lindberg, P. G. (2024). Post-Stroke Impairments of Manual Dexterity and Finger Proprioception: Their Contribution to Upper Limb Activity Capacity. Neurorehabilitation and neural repair, 38(5), 373–385. https://doi.org/10.1177/15459683241245416（The reason for exclusion: Non-RCTs）
19. Chen, L., Chen, Y., Fu, W. B., Huang, D. F., & Lo, W. L. A. (2022). The Effect of Virtual Reality on Motor Anticipation and Hand Function in Patients with Subacute Stroke: A Randomized Trial on Movement-Related Potential. *Neural plasticity*, *2022*, 7399995. https://doi.org/10.1155/2022/7399995（The reason for exclusion: Non robot-assisted task-oriented training）
20. Wong, Y., Li, C. J., Ada, L., Zhang, T., Månum, G., & Langhammer, B. (2022). Upper Limb Training with a Dynamic Hand Orthosis in Early Subacute Stroke: A Pilot Randomized Trial. *Journal of rehabilitation medicine*, *54*, jrm00279. https://doi.org/10.2340/jrm.v54.2231（The reason for exclusion: Non-RCTs）
21. Bian, L., Zhang, L., Huang, G., Song, D., Zheng, K., Xu, X., Dai, W., Ren, C., & Shen, Y. (2024). Effects of Priming Intermittent Theta Burst Stimulation With High-Definition tDCS on Upper Limb Function in Hemiparetic Patients With Stroke: A Randomized Controlled Study. *Neurorehabilitation and neural repair*, *38*(4), 268–278. https://doi.org/10.1177/15459683241233259（The reason for exclusion: Non robot-assisted task-oriented training）
22. Chen, Z. J., He, C., Guo, F., Xiong, C. H., & Huang, X. L. (2021). Exoskeleton-Assisted Anthropomorphic Movement Training (EAMT) for Poststroke Upper Limb Rehabilitation: A Pilot Randomized Controlled Trial. *Archives of physical medicine and rehabilitation*, *102*(11), 2074–2082. https://doi.org/10.1016/j.apmr.2021.06.001（The reason for exclusion: Control group using additional interventions)
23. Feng, T., Zhao, C., Dong, J., Xue, Z., Cai, F., Li, X., Hu, Z., & Xue, X. (2024). The effect of unaffected side resistance training on upper limb function reconstruction and prevention of sarcopenia in stroke patients: a randomized controlled trial. *Scientific reports*, *14*(1), 25330. <https://doi.org/10.1038/s41598-024-76810-2> （The reason for exclusion: duplicate record）
24. Wang, L., Wang, S., Zhang, S., Dou, Z., & Guo, T. (2023). Effectiveness and electrophysiological mechanisms of focal vibration on upper limb motor dysfunction in patients with subacute stroke: A randomized controlled trial. *Brain research*, *1809*, 148353. <https://doi.org/10.1016/j.brainres.2023.148353> （The reason for exclusion: Non robot-assisted task-oriented training）
25. Sentandreu-Mañó, T., Tomás, J. M., & Ricardo Salom Terrádez, J. (2021). A randomised clinical trial comparing 35 Hz versus 50 Hz frequency stimulation effects on hand motor recovery in older adults after stroke. *Scientific reports*, *11*(1), 9131. https://doi.org/10.1038/s41598-021-88607-8（The reason for exclusion: Non robot-assisted task-oriented training）
26. Hermann-Eriksen, M., Nilsen, T., Hove, Å., Eilertsen, L., Haugen, I. K., Sexton, J., & Kjeken, I. (2022). Comparison of 2 Postoperative Therapy Regimens After Trapeziectomy Due to Osteoarthritis: A Randomized, Controlled Trial. *The Journal of hand surgery*, *47*(2), 120–129.e4. <https://doi.org/10.1016/j.jhsa.2021.08.015> （The reason for exclusion: Non robot-assisted task-oriented training）
27. Pearse, J. E., Cadwgan, J. E., Wisher, V., Jesmont, C., Mason-Burton, P., Barry, M., Jona James, J., Kelly, S., & Basu, A. P. (2020). Feasibility Trial of Thumb Taping by Parents in Infants with Cerebral Palsy: Brief Report. *Developmental neurorehabilitation*, *23*(1), 50–58. <https://doi.org/10.1080/17518423.2019.1566280> （The reason for exclusion: Non robot-assisted task-oriented training）
28. Rowe, J. B., Chan, V., Ingemanson, M. L., Cramer, S. C., Wolbrecht, E. T., & Reinkensmeyer, D. J. (2017). Robotic Assistance for Training Finger Movement Using a Hebbian Model: A Randomized Controlled Trial. *Neurorehabilitation and neural repair*, *31*(8), 769–780. <https://doi.org/10.1177/1545968317721975> （The reason for exclusion: Control group using additional interventions)
29. Cantero-Téllez, R., Pérez-Cruzado, D., Villafañe, J. H., García-Orza, S., Naughton, N., & Valdes, K. (2022). The Effect of Proprioception Training on Pain Intensity in Thumb Basal Joint Osteoarthritis: A Randomized Controlled Trial. *International journal of environmental research and public health*, *19*(6), 3592. <https://doi.org/10.3390/ijerph19063592> （The reason for exclusion: Non robot-assisted task-oriented training）
30. Susanto, E. A., Tong, R. K., Ockenfeld, C., & Ho, N. S. (2015). Efficacy of robot-assisted fingers training in chronic stroke survivors: a pilot randomized-controlled trial. *Journal of neuroengineering and rehabilitation*, *12*, 42. <https://doi.org/10.1186/s12984-015-0033-5> （The reason for exclusion: Non robot-assisted task-oriented training）
31. Wang, L., Zhu, Q. X., Zhong, M. H., Zhou, R. Z., Liu, X. Q., Tang, N. S., Feng, X. C., & Gao, C. F. (2022). Effects of corticospinal tract integrity on upper limb motor function recovery in stroke patients treated with repetitive transcranial magnetic stimulation. *Journal of integrative neuroscience*, *21*(2), 50. （The reason for exclusion: Non robot-assisted task-oriented training）<https://doi.org/10.31083/j.jin2102050> （The reason for exclusion: Non robot-assisted task-oriented training）
32. Eyiis, E., Mathijssen, N. M. C., Kok, P., Sluijter, J., & Kraan, G. A. (2023). Three-dimensional printed customized versus conventional plaster brace for trapeziometacarpal osteoarthritis: a randomized controlled crossover trial. *The Journal of hand surgery, European volume*, *48*(5), 412–418. <https://doi.org/10.1177/17531934221146864> （The reason for exclusion: Non robot-assisted task-oriented training）
33. Brunner, I., Skouen, J. S., Hofstad, H., Aßmus, J., Becker, F., Sanders, A. M., Pallesen, H., Qvist Kristensen, L., Michielsen, M., Thijs, L., & Verheyden, G. (2017). Virtual Reality Training for Upper Extremity in Subacute Stroke (VIRTUES): A multicenter RCT. *Neurology*, *89*(24), 2413–2421. <https://doi.org/10.1212/WNL.0000000000004744> （The reason for exclusion: Non robot-assisted task-oriented training）
34. Tramontano, M., Morone, G., De Angelis, S., Casagrande Conti, L., Galeoto, G., & Grasso, M. G. (2020). Sensor-based technology for upper limb rehabilitation in patients with multiple sclerosis: A randomized controlled trial. *Restorative neurology and neuroscience*, *38*(4), 333–341. <https://doi.org/10.3233/RNN-201033> （The reason for exclusion: Irrelevant study outcome）
35. Seo, N. J., Ramakrishnan, V., Woodbury, M. L., Bonilha, L., Finetto, C., Schranz, C., Scronce, G., Coupland, K., Blaschke, J., Baker, A., Howard, K., Meinzer, C., Velozo, C. A., & Adams, R. J. (2022). Concomitant sensory stimulation during therapy to enhance hand functional recovery post stroke. *Trials*, *23*(1), 262. <https://doi.org/10.1186/s13063-022-06241-9> （The reason for exclusion: Non robot-assisted task-oriented training）
36. Sale, P., Mazzoleni, S., Lombardi, V., Galafate, D., Massimiani, M. P., Posteraro, F., Damiani, C., & Franceschini, M. (2014). Recovery of hand function with robot-assisted therapy in acute stroke patients: a randomized-controlled trial. *International journal of rehabilitation research. Internationale Zeitschrift fur Rehabilitationsforschung. Revue internationale de recherches de readaptation*, *37*(3), 236–242. <https://doi.org/10.1097/MRR.0000000000000059> （The reason for exclusion: Irrelevant study outcome）
37. Tveter, A. T., Østerås, N., Nossum, R., Eide, R. E. M., Klokkeide, Å., Matre, K. H., Olsen, M., & Kjeken, I. (2022). Short-Term Effects of Occupational Therapy on Hand Function and Pain in Patients With Carpometacarpal Osteoarthritis: Secondary Analyses From a Randomized Controlled Trial. *Arthritis care & research*, *74*(6), 955–964. <https://doi.org/10.1002/acr.24543> （The reason for exclusion: review）
38. Choi, Y. H., Ku, J., Lim, H., Kim, Y. H., & Paik, N. J. (2016). Mobile game-based virtual reality rehabilitation program for upper limb dysfunction after ischemic stroke. *Restorative neurology and neuroscience*, *34*(3), 455–463. <https://doi.org/10.3233/RNN-150626> （The reason for exclusion: Non robot-assisted task-oriented training）
39. Wang, T. N., Liang, K. J., Liu, Y. C., Shieh, J. Y., & Chen, H. L. (2023). Effects of Intensive Versus Distributed Constraint-Induced Movement Therapy for Children With Unilateral Cerebral Palsy: A Quasi-Randomized Trial. *Neurorehabilitation and neural repair*, *37*(2-3), 109–118. https://doi.org/10.1177/15459683231162330（The reason for exclusion: Non-RCTs）
40. Stinear, C. M., Petoe, M. A., Anwar, S., Barber, P. A., & Byblow, W. D. (2014). Bilateral priming accelerates recovery of upper limb function after stroke: a randomized controlled trial. *Stroke*, *45*(1), 205–210. <https://doi.org/10.1161/STROKEAHA.113.003537> （The reason for exclusion: Non robot-assisted task-oriented training）
41. Palomo-Carrión, R., Zuil-Escobar, J. C., Cabrera-Guerra, M., Barreda-Martínez, P., & Martínez-Cepa, C. B. (2022). Terapia en espejo y de observación de la acción en niños con parálisis cerebral espástica unilateral: estudio de viabilidad [Mirror and action observation therapy in children with unilateral spastic cerebral palsy: a feasibility study]. *Revista de neurologia*, *75*(11), 325–332. <https://doi.org/10.33588/rn.7511.2022343> （The reason for exclusion: Non robot-assisted task-oriented training）
42. Zhou, T., Zhu, D., Xu, X. Y., Luo, Z. H., Gu, X. L., Chen, J., Chen, Z. Q., Chen, B., Zhang, Y. L., Wang, K. X., Yan, P., & Chen, S. (2022). *Zhongguo zhen jiu = Chinese acupuncture & moxibustion*, *42*(10), 1120–1124. <https://doi.org/10.13703/j.0255-2930.20211030-0001> （The reason for exclusion: Non robot-assisted task-oriented training）
43. Liu, Y. F., Mao, H. L., Li, Y. J., Zhao, T., Wang, Z. M., Liu, Y. Y., An, J. M., & Huang, L. N. (2023). *Zhongguo zhen jiu = Chinese acupuncture & moxibustion*, *43*(10), 1109–1113. <https://doi.org/10.13703/j.0255-2930.20230131-0002> （The reason for exclusion: Non robot-assisted task-oriented training）
44. Radajewska, A., Opara, J. A., Kucio, C., Błaszczyszyn, M., Mehlich, K., & Szczygiel, J. (2013). The effects of mirror therapy on arm and hand function in subacute stroke in patients. *International journal of rehabilitation research. Internationale Zeitschrift fur Rehabilitationsforschung. Revue internationale de recherches de readaptation*, *36*(3), 268–274. <https://doi.org/10.1097/MRR.0b013e3283606218> （The reason for exclusion: duplicate record）
45. Xu, L., Zhang, Z., Xu, W., Liu, H., Han, X., & Wang, M. (2024). Interactive scalp acupuncture combined with suspension digital occupational therapy for upper limb motor dysfunction after stroke: a multi-center randomized controlled trial. 互动式头针结合悬吊下数字作业疗法治疗脑卒中后上肢运动功能障碍：多中心随机对照试验. *Zhongguo zhen jiu = Chinese acupuncture & moxibustion*, *44*(2), 129–133. https://doi.org/10.13703/j.0255-2930.20230507-k0001（The reason for exclusion: Non robot-assisted task-oriented training）
46. Jiang, W., Wang, S., Wu, Q., & Li, X. (2021). Effects of Self-Assisted Shoulder Elevation of the Affected Side Combined with Balance Training on Associated Reactions of Upper Limb and Walking Function in Chronic Stroke Patients: A Randomized Controlled Trial. *Medical science monitor : international medical journal of experimental and clinical research*, *27*, e928549. https://doi.org/10.12659/MSM.928549（The reason for exclusion: Control group using additional interventions)
47. Vanoglio, F., Bernocchi, P., Mulè, C., Garofali, F., Mora, C., Taveggia, G., Scalvini, S., & Luisa, A. (2017). Feasibility and efficacy of a robotic device for hand rehabilitation in hemiplegic stroke patients: a randomized pilot controlled study. *Clinical rehabilitation*, *31*(3), 351–360. https://doi.org/10.1177/0269215516642606（The reason for exclusion: Irrelevant study outcome）
48. Straudi, S., Baroni, A., Mele, S., Craighero, L., Manfredini, F., Lamberti, N., Maietti, E., & Basaglia, N. (2020). Effects of a Robot-Assisted Arm Training Plus Hand Functional Electrical Stimulation on Recovery After Stroke: A Randomized Clinical Trial. *Archives of physical medicine and rehabilitation*, *101*(2), 309–316. https://doi.org/10.1016/j.apmr.2019.09.016（The reason for exclusion: duplicate record）
49. Keeling, A. B., Piitz, M., Semrau, J. A., Hill, M. D., Scott, S. H., & Dukelow, S. P. (2021). Robot enhanced stroke therapy optimizes rehabilitation (RESTORE): a pilot study. *Journal of neuroengineering and rehabilitation*, *18*(1), 10. <https://doi.org/10.1186/s12984-021-00804-8>(This document meets the inclusion criteria)
50. Perini, G., Bertoni, R., Thorsen, R., Carpinella, I., Lencioni, T., Ferrarin, M., & Jonsdottir, J. (2021). Sequentially applied myoelectrically controlled FES in a task-oriented approach and robotic therapy for the recovery of upper limb in post-stroke patients: A randomized controlled pilot study. *Technology and health care : official journal of the European Society for Engineering and Medicine*, *29*(3), 419–429. <https://doi.org/10.3233/THC-202371>(This document meets the inclusion criteria)
51. He, Y. Z., Huang, Z. M., Deng, H. Y., Huang, J., Wu, J. H., & Wu, J. S. (2023). Feasibility, safety, and efficacy of task-oriented mirrored robotic training on upper-limb functions and activities of daily living in subacute poststroke patients: a pilot study. *European journal of physical and rehabilitation medicine*, *59*(6), 660–668. <https://doi.org/10.23736/S1973-9087.23.08018-8>(This document meets the inclusion criteria)
52. Hsieh, Y. W., Wu, C. Y., Wang, W. E., Lin, K. C., Chang, K. C., Chen, C. C., & Liu, C. T. (2017). Bilateral robotic priming before task-oriented approach in subacute stroke rehabilitation: a pilot randomized controlled trial. *Clinical rehabilitation*, *31*(2), 225–233. <https://doi.org/10.1177/0269215516633275>(This document meets the inclusion criteria)
53. Nakipoğlu Yuzer, G. F., Köse Dönmez, B., & Özgirgin, N. (2017). A Randomized Controlled Study: Effectiveness of Functional Electrical Stimulation on Wrist and Finger Flexor Spasticity in Hemiplegia. *Journal of stroke and cerebrovascular diseases : the official journal of National Stroke Association*, *26*(7), 1467–1471. https://doi.org/10.1016/j.jstrokecerebrovasdis.2017.03.011（The reason for exclusion: Non robot-assisted task-oriented training）
54. Renner, C. I. E., Brendel, C., & Hummelsheim, H. (2020). Bilateral Arm Training vs Unilateral Arm Training for Severely Affected Patients With Stroke: Exploratory Single-Blinded Randomized Controlled Trial. *Archives of physical medicine and rehabilitation*, *101*(7), 1120–1130. https://doi.org/10.1016/j.apmr.2020.02.007（The reason for exclusion: Non robot-assisted task-oriented training）
55. Cordo, P., Wolf, S., Lou, J. S., Bogey, R., Stevenson, M., Hayes, J., & Roth, E. (2013). Treatment of severe hand impairment following stroke by combining assisted movement, muscle vibration, and biofeedback. *Journal of neurologic physical therapy : JNPT*, *37*(4), 194–203. https://doi.org/10.1097/NPT.0000000000000023（The reason for exclusion: Non robot-assisted task-oriented training）
56. Sgandurra, G., Ferrari, A., Cossu, G., Guzzetta, A., Fogassi, L., & Cioni, G. (2013). Randomized trial of observation and execution of upper extremity actions versus action alone in children with unilateral cerebral palsy. *Neurorehabilitation and neural repair*, *27*(9), 808–815. https://doi.org/10.1177/1545968313497101（The reason for exclusion: Non robot-assisted task-oriented training）
57. Hsu, H. Y., Kuan, T. S., Tsai, C. L., Wu, P. T., Kuo, Y. L., Su, F. C., & Kuo, L. C. (2021). Effect of a Novel Perturbation-Based Pinch Task Training on Sensorimotor Performance of Upper Extremity for Patients With Chronic Stroke: A Pilot Randomized Controlled Trial. *Archives of physical medicine and rehabilitation*, *102*(5), 811–818. https://doi.org/10.1016/j.apmr.2020.11.004（The reason for exclusion: Irrelevant study outcome）
58. Calabrò, R. S., Accorinti, M., Porcari, B., Carioti, L., Ciatto, L., Billeri, L., Andronaco, V. A., Galletti, F., Filoni, S., & Naro, A. (2019). Does hand robotic rehabilitation improve motor function by rebalancing interhemispheric connectivity after chronic stroke? Encouraging data from a randomised-clinical-trial. *Clinical neurophysiology : official journal of the International Federation of Clinical Neurophysiology*, *130*(5), 767–780. https://doi.org/10.1016/j.clinph.2019.02.013（The reason for exclusion: Non robot-assisted task-oriented training）
59. Kwakkel, G., Winters, C., van Wegen, E. E., Nijland, R. H., van Kuijk, A. A., Visser-Meily, A., de Groot, J., de Vlugt, E., Arendzen, J. H., Geurts, A. C., Meskers, C. G., & EXPLICIT-Stroke Consortium (2016). Effects of Unilateral Upper Limb Training in Two Distinct Prognostic Groups Early After Stroke: The EXPLICIT-Stroke Randomized Clinical Trial. *Neurorehabilitation and neural repair*, *30*(9), 804–816. https://doi.org/10.1177/1545968315624784（The reason for exclusion: Non robot-assisted task-oriented training）
60. Lannin, N. A., Cusick, A., Hills, C., Kinnear, B., Vogel, K., Matthews, K., & Bowring, G. (2016). Upper limb motor training using a Saebo™ orthosis is feasible for increasing task-specific practice in hospital after stroke. *Australian occupational therapy journal*, *63*(6), 364–372. https://doi.org/10.1111/1440-1630.12330（The reason for exclusion: without relevant date）
61. Bang, D. H., Shin, W. S., & Choi, H. S. (2018). Effects of modified constraint-induced movement therapy with trunk restraint in early stroke patients: A single-blinded, randomized, controlled, pilot trial. *NeuroRehabilitation*, *42*(1), 29–35. https://doi.org/10.3233/NRE-172176（The reason for exclusion: Control group using additional interventions)
62. van Delden, A. L., Peper, C. L., Nienhuys, K. N., Zijp, N. I., Beek, P. J., & Kwakkel, G. (2013). Unilateral versus bilateral upper limb training after stroke: the Upper Limb Training After Stroke clinical trial. *Stroke*, *44*(9), 2613–2616. https://doi.org/10.1161/STROKEAHA.113.001969（The reason for exclusion: Non-RCTs）
63. Imms, C., Wallen, M., Elliott, C., Hoare, B., Randall, M., Greaves, S., Adair, B., Bradshaw, E., Carter, R., Orsini, F., Shih, S. T., & Reddihough, D. (2016). Minimising impairment: Protocol for a multicentre randomised controlled trial of upper limb orthoses for children with cerebral palsy. *BMC pediatrics*, *16*, 70. https://doi.org/10.1186/s12887-016-0608-8（The reason for exclusion: Non robot-assisted task-oriented training）
64. Fu, J., Zeng, M., Shen, F., Cui, Y., Zhu, M., Gu, X., & Sun, Y. (2017). Effects of action observation therapy on upper extremity function, daily activities and motion evoked potential in cerebral infarction patients. *Medicine*, *96*(42), e8080. https://doi.org/10.1097/MD.0000000000008080（The reason for exclusion: Non robot-assisted task-oriented training）
65. Zhou, Y. X., Xia, Y., Huang, J., Wang, H. P., Bao, X. L., Bi, Z. Y., Chen, X. B., Gao, Y. J., Lü, X. Y., & Wang, Z. G. (2017). Electromyographic bridge for promoting the recovery of hand movements in subacute stroke patients: A randomized controlled trial. *Journal of rehabilitation medicine*, *49*(8), 629–636. https://doi.org/10.2340/16501977-2256（The reason for exclusion: Non robot-assisted task-oriented training）
66. Zhou, Y. X., Xia, Y., Huang, J., Wang, H. P., Bao, X. L., Bi, Z. Y., Chen, X. B., Gao, Y. J., Lü, X. Y., & Wang, Z. G. (2017). Electromyographic bridge for promoting the recovery of hand movements in subacute stroke patients: A randomized controlled trial. *Journal of rehabilitation medicine*, *49*(8), 629–636. https://doi.org/10.2340/16501977-2256（The reason for exclusion: Non robot-assisted task-oriented training）
67. Kirac-Unal, Z., Gencay-Can, A., Karaca-Umay, E., & Cakci, F. A. (2019). The effect of task-oriented electromyography-triggered electrical stimulation of the paretic wrist extensors on upper limb motor function early after stroke: a pilot randomized controlled trial. *International journal of rehabilitation research. Internationale Zeitschrift fur Rehabilitationsforschung. Revue internationale de recherches de readaptation*, *42*(1), 74–81. https://doi.org/10.1097/MRR.0000000000000333（The reason for exclusion: Irrelevant study outcome）
68. Chae, J., Harley, M. Y., Hisel, T. Z., Corrigan, C. M., Demchak, J. A., Wong, Y. T., & Fang, Z. P. (2009). Intramuscular electrical stimulation for upper limb recovery in chronic hemiparesis: an exploratory randomized clinical trial. *Neurorehabilitation and neural repair*, *23*(6), 569–578. https://doi.org/10.1177/1545968308328729（The reason for exclusion: Non robot-assisted task-oriented training）
69. Prange-Lasonder, G. B., Radder, B., Kottink, A. I. R., Melendez-Calderon, A., Buurke, J. H., & Rietman, J. S. (2017). Applying a soft-robotic glove as assistive device and training tool with games to support hand function after stroke: Preliminary results on feasibility and potential clinical impact. *IEEE ... International Conference on Rehabilitation Robotics : [proceedings]*, *2017*, 1401–1406. https://doi.org/10.1109/ICORR.2017.8009444（The reason for exclusion: Irrelevant study outcome）
70. Chae, J., Bethoux, F., Bohine, T., Dobos, L., Davis, T., & Friedl, A. (1998). Neuromuscular stimulation for upper extremity motor and functional recovery in acute hemiplegia. *Stroke*, *29*(5), 975–979. <https://doi.org/10.1161/01.str.29.5.975> （The reason for exclusion: Non robot-assisted task-oriented training）
71. Bakker, C. D., Massa, M., Daffertshofer, A., Pasman, J. W., van Kuijk, A. A., Kwakkel, G., & Stegeman, D. F. (2019). The addition of the MEP amplitude of finger extension muscles to clinical predictors of hand function after stroke: A prospective cohort study. *Restorative neurology and neuroscience*, *37*(5), 445–456. https://doi.org/10.3233/RNN-180890（The reason for exclusion: Non robot-assisted task-oriented training）
72. Yıldızgören, M. T., Nakipoğlu Yüzer, G. F., Ekiz, T., & Özgirgin, N. (2014). Effects of neuromuscular electrical stimulation on the wrist and finger flexor spasticity and hand functions in cerebral palsy. *Pediatric neurology*, *51*(3), 360–364. https://doi.org/10.1016/j.pediatrneurol.2014.05.009（The reason for exclusion: Non robot-assisted task-oriented training）
73. Lin, C. H., Chou, L. W., Luo, H. J., Tsai, P. Y., Lieu, F. K., Chiang, S. L., & Sung, W. H. (2015). Effects of Computer-Aided Interlimb Force Coupling Training on Paretic Hand and Arm Motor Control following Chronic Stroke: A Randomized Controlled Trial. *PloS one*, *10*(7), e0131048. https://doi.org/10.1371/journal.pone.0131048（The reason for exclusion: duplicate record）
74. Kwon, T. G., Park, E., Kang, C., Chang, W. H., & Kim, Y. H. (2016). The effects of combined repetitive transcranial magnetic stimulation and transcranial direct current stimulation on motor function in patients with stroke. *Restorative neurology and neuroscience*, *34*(6), 915–923. https://doi.org/10.3233/RNN-160654（The reason for exclusion: duplicate record）
75. Fluet, G. G., Merians, A. S., Qiu, Q., Davidow, A., & Adamovich, S. V. (2014). Comparing integrated training of the hand and arm with isolated training of the same effectors in persons with stroke using haptically rendered virtual environments, a randomized clinical trial. *Journal of neuroengineering and rehabilitation*, *11*, 126. <https://doi.org/10.1186/1743-0003-11-126> （The reason for exclusion: duplicate record）
76. Hwang, C. H., Seong, J. W., & Son, D. S. (2012). Individual finger synchronized robot-assisted hand rehabilitation in subacute to chronic stroke: a prospective randomized clinical trial of efficacy. *Clinical rehabilitation*, *26*(8), 696–704. <https://doi.org/10.1177/0269215511431473> （The reason for exclusion: duplicate record）
77. Bai, Y. L., Hu, Y. S., Wu, Y., Zhu, Y. L., Zhang, B., Jiang, C. Y., Sun, L. M., & Fan, W. K. (2014). Long-term three-stage rehabilitation intervention alleviates spasticity of the elbows, fingers, and plantar flexors and improves activities of daily living in ischemic stroke patients: a randomized, controlled trial. *Neuroreport*, *25*(13), 998–1005. <https://doi.org/10.1097/WNR.0000000000000194> （The reason for exclusion: duplicate record）
78. Jackman, M., Novak, I., & Lannin, N. (2014). Effectiveness of functional hand splinting and the cognitive orientation to occupational performance (CO-OP) approach in children with cerebral palsy and brain injury: two randomised controlled trial protocols. *BMC neurology*, *14*, 144. https://doi.org/10.1186/1471-2377-14-144（The reason for exclusion: Non robot-assisted task-oriented training）
79. Choi, Y., Gordon, J., Park, H., & Schweighofer, N. (2011). Feasibility of the adaptive and automatic presentation of tasks (ADAPT) system for rehabilitation of upper extremity function post-stroke. *Journal of neuroengineering and rehabilitation*, *8*, 42. <https://doi.org/10.1186/1743-0003-8-42>（The reason for exclusion: duplicate record）
80. Thorsen, R., Cortesi, M., Jonsdottir, J., Carpinella, I., Morelli, D., Casiraghi, A., Puglia, M., Diverio, M., & Ferrarin, M. (2013). Myoelectrically driven functional electrical stimulation may increase motor recovery of upper limb in poststroke subjects: a randomized controlled pilot study. *Journal of rehabilitation research and development*, *50*(6), 785–794. <https://doi.org/10.1682/JRRD.2012.07.0123>（The reason for exclusion: duplicate record）
81. Iosa, M., Morone, G., Ragaglini, M. R., Fusco, A., & Paolucci, S. (2013). Motor strategies and bilateral transfer in sensorimotor learning of patients with subacute stroke and healthy subjects. A randomized controlled trial. *European journal of physical and rehabilitation medicine*, *49*(3), 291–299.（The reason for exclusion: duplicate record）
82. Winters, C., Kwakkel, G., Nijland, R., van Wegen, E., & EXPLICIT-stroke consortium (2016). When Does Return of Voluntary Finger Extension Occur Post-Stroke? A Prospective Cohort Study. *PloS one*, *11*(8), e0160528. https://doi.org/10.1371/journal.pone.0160528（The reason for exclusion: duplicate record）
83. Kutner, N. G., Zhang, R., Butler, A. J., Wolf, S. L., & Alberts, J. L. (2010). Quality-of-life change associated with robotic-assisted therapy to improve hand motor function in patients with subacute stroke: a randomized clinical trial. *Physical therapy*, *90*(4), 493–504. https://doi.org/10.2522/ptj.20090160（The reason for exclusion: Irrelevant study outcome）
84. Kilbreath, S. L., Crosbie, J., Canning, C. G., & Lee, M. J. (2006). Inter-limb coordination in bimanual reach-to-grasp following stroke. *Disability and rehabilitation*, *28*(23), 1435–1443. https://doi.org/10.1080/09638280600638307（The reason for exclusion: Irrelevant study outcome）
85. Germanotta, M., Gower, V., Papadopoulou, D., Cruciani, A., Pecchioli, C., Mosca, R., Speranza, G., Falsini, C., Cecchi, F., Vannetti, F., Montesano, A., Galeri, S., Gramatica, F., Aprile, I., & FDG Robotic Rehabilitation Group (2020). Reliability, validity and discriminant ability of a robotic device for finger training in patients with subacute stroke. *Journal of neuroengineering and rehabilitation*, *17*(1), 1. https://doi.org/10.1186/s12984-019-0634-5（The reason for exclusion: Non robot-assisted task-oriented training）
86. Hesse, S., Kuhlmann, H., Wilk, J., Tomelleri, C., & Kirker, S. G. (2008). A new electromechanical trainer for sensorimotor rehabilitation of paralysed fingers: a case series in chronic and acute stroke patients. *Journal of neuroengineering and rehabilitation*, *5*, 21. https://doi.org/10.1186/1743-0003-5-21（The reason for exclusion: Non robot-assisted task-oriented training）
87. Carey, J. R., Kimberley, T. J., Lewis, S. M., Auerbach, E. J., Dorsey, L., Rundquist, P., & Ugurbil, K. (2002). Analysis of fMRI and finger tracking training in subjects with chronic stroke. *Brain : a journal of neurology*, *125*(Pt 4), 773–788. <https://doi.org/10.1093/brain/awf091>（The reason for exclusion: Non robot-assisted task-oriented training）
88. Bhatt, E., Nagpal, A., Greer, K. H., Grunewald, T. K., Steele, J. L., Wiemiller, J. W., Lewis, S. M., & Carey, J. R. (2007). Effect of finger tracking combined with electrical stimulation on brain reorganization and hand function in subjects with stroke. *Experimental brain research*, *182*(4), 435–447. <https://doi.org/10.1007/s00221-007-1001-5>（The reason for exclusion: duplicate record）
89. Fischer, H. C., Stubblefield, K., Kline, T., Luo, X., Kenyon, R. V., & Kamper, D. G. (2007). Hand rehabilitation following stroke: a pilot study of assisted finger extension training in a virtual environment. *Topics in stroke rehabilitation*, *14*(1), 1–12. <https://doi.org/10.1310/tsr1401-1>（The reason for exclusion: duplicate record）
90. Jahangir, A. W., Tan, H. J., Norlinah, M. I., Nafisah, W. Y., Ramesh, S., Hamidon, B. B., & Raymond, A. A. (2007). Intramuscular injection of botulinum toxin for the treatment of wrist and finger spasticity after stroke. *The Medical journal of Malaysia*, *62*(4), 319–322.（The reason for exclusion: Non robot-assisted task-oriented training）
91. Cauraugh, J., Light, K., Kim, S., Thigpen, M., & Behrman, A. (2000). Chronic motor dysfunction after stroke: recovering wrist and finger extension by electromyography-triggered neuromuscular stimulation. *Stroke*, *31*(6), 1360–1364. <https://doi.org/10.1161/01.str.31.6.1360>（The reason for exclusion: Non robot-assisted task-oriented training）
92. Kim, D. G., Cho, Y. W., Hong, J. H., Song, J. C., Chung, H. A., Bai, D. S., Lee, C. H., & Jang, S. H. (2008). Effect of constraint-induced movement therapy with modified opposition restriction orthosis in chronic hemiparetic patients with stroke. NeuroRehabilitation, 23(3), 239–244.（The reason for exclusion: Non robot-assisted task-oriented training）
93. Seniów, J., Bilik, M., Leśniak, M., Waldowski, K., Iwański, S., & Członkowska, A. (2012). Transcranial magnetic stimulation combined with physiotherapy in rehabilitation of poststroke hemiparesis: a randomized, double-blind, placebo-controlled study. *Neurorehabilitation and neural repair*, *26*(9), 1072–1079. <https://doi.org/10.1177/1545968312445635>（The reason for exclusion: duplicate record）
94. Thielbar, K. O., Lord, T. J., Fischer, H. C., Lazzaro, E. C., Barth, K. C., Stoykov, M. E., Triandafilou, K. M., & Kamper, D. G. (2014). Training finger individuation with a mechatronic-virtual reality system leads to improved fine motor control post-stroke. *Journal of neuroengineering and rehabilitation*, *11*, 171. <https://doi.org/10.1186/1743-0003-11-171>（The reason for exclusion: duplicate record）
95. Singer, B. J., Vallence, A. M., Cleary, S., Cooper, I., & Loftus, A. M. (2013). The effect of EMG triggered electrical stimulation plus task practice on arm function in chronic stroke patients with moderate-severe arm deficits. *Restorative neurology and neuroscience*, *31*(6), 681–691. <https://doi.org/10.3233/RNN-130319>（The reason for exclusion: Non robot-assisted task-oriented training）
96. Dolganov, M. V., & Karpova, M. I. (2019). Virtual'naia real'nost' pri narushenii funktsii ruki: osobennosti primeneniia v ostrom periode insul'ta [Virtual reality in upper extremity dysfunction: specific features of usage in acute stroke]. *Voprosy kurortologii, fizioterapii, i lechebnoi fizicheskoi kultury*, *96*(5), 19–28. <https://doi.org/10.17116/kurort20199605119>（The reason for exclusion: Non robot-assisted task-oriented training）
97. Trombly, C. A., Thayer-Nason, L., Bliss, G., Girard, C. A., Lyrist, L. A., & Brexa-Hooson, A. (1986). The effectiveness of therapy in improving finger extension in stroke patients. *The American journal of occupational therapy : official publication of the American Occupational Therapy Association*, *40*(9), 612–617. https://doi.org/10.5014/ajot.40.9.612（The reason for exclusion: Non-RCTs）
98. Celnik, P., Webster, B., Glasser, D. M., & Cohen, L. G. (2008). Effects of action observation on physical training after stroke. *Stroke*, *39*(6), 1814–1820. https://doi.org/10.1161/STROKEAHA.107.508184（The reason for exclusion: Non robot-assisted task-oriented training）
99. Ro, T., Noser, E., Boake, C., Johnson, R., Gaber, M., Speroni, A., Bernstein, M., De Joya, A., Scott Burgin, W., Zhang, L., Taub, E., Grotta, J. C., & Levin, H. S. (2006). Functional reorganization and recovery after constraint-induced movement therapy in subacute stroke: case reports. *Neurocase*, *12*(1), 50–60. https://doi.org/10.1080/13554790500493415（The reason for exclusion: Non robot-assisted task-oriented training）
100. Madhoun, H. Y., Tan, B., Feng, Y., Zhou, Y., Zhou, C., & Yu, L. (2020). Task-based mirror therapy enhances the upper limb motor function in subacute stroke patients: a randomized control trial. *European journal of physical and rehabilitation medicine*, *56*(3), 265–271. <https://doi.org/10.23736/S1973-9087.20.06070-0>（The reason for exclusion: duplicate record）
101. Ranzani, R., Lambercy, O., Metzger, J. C., Califfi, A., Regazzi, S., Dinacci, D., Petrillo, C., Rossi, P., Conti, F. M., & Gassert, R. (2020). Neurocognitive robot-assisted rehabilitation of hand function: a randomized control trial on motor recovery in subacute stroke. *Journal of neuroengineering and rehabilitation*, *17*(1), 115. <https://doi.org/10.1186/s12984-020-00746-7>（The reason for exclusion: duplicate record）
102. Ji, E. K., Wang, H. H., Jung, S. J., Lee, K. B., Kim, J. S., Jo, L., Hong, B. Y., & Lim, S. H. (2021). Graded motor imagery training as a home exercise program for upper limb motor function in patients with chronic stroke: A randomized controlled trial. *Medicine*, *100*(3), e24351. <https://doi.org/10.1097/MD.0000000000024351>（The reason for exclusion: duplicate record）
103. Lee, H. C., Kuo, F. L., Lin, Y. N., Liou, T. H., Lin, J. C., & Huang, S. W. (2021). Effects of Robot-Assisted Rehabilitation on Hand Function of People With Stroke: A Randomized, Crossover-Controlled, Assessor-Blinded Study. *The American journal of occupational therapy : official publication of the American Occupational Therapy Association*, *75*(1), 7501205020p1–7501205020p11. <https://doi.org/10.5014/ajot.2021.038232>（The reason for exclusion: duplicate record）
104. Villafañe, J. H., Taveggia, G., Galeri, S., Bissolotti, L., Mullè, C., Imperio, G., Valdes, K., Borboni, A., & Negrini, S. (2018). Efficacy of Short-Term Robot-Assisted Rehabilitation in Patients With Hand Paralysis After Stroke: A Randomized Clinical Trial. *Hand (New York, N.Y.)*, *13*(1), 95–102. <https://doi.org/10.1177/1558944717692096>（The reason for exclusion: duplicate record）
105. Huang, Y. C., Chen, P. C., Tso, H. H., Yang, Y. C., Ho, T. L., & Leong, C. P. (2019). Effects of kinesio taping on hemiplegic hand in patients with upper limb post-stroke spasticity: a randomized controlled pilot study. *European journal of physical and rehabilitation medicine*, *55*(5), 551–557. （The reason for exclusion: duplicate record）<https://doi.org/10.23736/S1973-9087.19.05684-3>（The reason for exclusion: duplicate record）
106. Aguilera-Rubio, Á., Alguacil-Diego, I. M., Mallo-López, A., Jardón Huete, A., Oña, E. D., & Cuesta-Gómez, A. (2024). Use of low-cost virtual reality in the treatment of the upper extremity in chronic stroke: a randomized clinical trial. *Journal of neuroengineering and rehabilitation*, *21*(1), 12. <https://doi.org/10.1186/s12984-024-01303-2>（The reason for exclusion: duplicate record）
107. Tang, C., Zhou, T., Zhang, Y., Yuan, R., Zhao, X., Yin, R., Song, P., Liu, B., Song, R., Chen, W., & Wang, H. (2023). Bilateral upper limb robot-assisted rehabilitation improves upper limb motor function in stroke patients: a study based on quantitative EEG. *European journal of medical research*, *28*(1), 603. <https://doi.org/10.1186/s40001-023-01565-x>（The reason for exclusion: duplicate record）
108. Xia, X., Dong, X., Huo, H., Zhang, Y., Song, J., & Wang, D. (2023). Clinical study of low-frequency acupoint electrical stimulation to improve thumb-to-finger movements after stroke: A randomized controlled trial. *Medicine*, *102*(47), e35755. https://doi.org/10.1097/MD.0000000000035755（The reason for exclusion: Irrelevant study outcome）
109. Vanoglio, F., Comini, L., Gaiani, M., Bonometti, G. P., Luisa, A., & Bernocchi, P. (2024). A Sensor-Based Upper Limb Treatment in Hemiplegic Patients: Results from a Randomized Pilot Study. *Sensors (Basel, Switzerland)*, *24*(8), 2574. https://doi.org/10.3390/s24082574（The reason for exclusion: duplicate record）
110. Akgün, İ., Demirbüken, İ., Timurtaş, E., Pehlivan, M. K., Pehlivan, A. U., Polat, M. G., Francisco, G. E., & Yozbatiran, N. (2024). Exoskeleton-assisted upper limb rehabilitation after stroke: a randomized controlled trial. *Neurological research*, *46*(11), 1074–1082. https://doi.org/10.1080/01616412.2024.2381385（The reason for exclusion: duplicate record）
111. Bernal-Jiménez, J. J., Dileone, M., Mordillo-Mateos, L., Martín-Conty, J. L., Durantez-Fernández, C., Viñuela, A., Martín-Rodríguez, F., Lerin-Calvo, A., Alcántara-Porcuna, V., & Polonio-López, B. (2024). Combining Transcranial Direct Current Stimulation With Hand Robotic Rehabilitation in Chronic Stroke Patients: A Double-Blind Randomized Clinical Trial. *American journal of physical medicine & rehabilitation*, *103*(10), 875–882. https://doi.org/10.1097/PHM.0000000000002446（The reason for exclusion: duplicate record）
112. Li, Y., Lian, Y., Chen, X., Zhang, H., Xu, G., Duan, H., Xie, X., & Li, Z. (2024). Effect of task-oriented training assisted by force feedback hand rehabilitation robot on finger grasping function in stroke patients with hemiplegia: a randomised controlled trial. *Journal of neuroengineering and rehabilitation*, *21*(1), 77. https://doi.org/10.1186/s12984-024-01372-3（The reason for exclusion: duplicate record）
113. van Ravestyn, C., Gerardin, E., Térémetz, M., Hamdoun, S., Baron, J. C., Calvet, D., Vandermeeren, Y., Turc, G., Maier, M. A., Rosso, C., Mas, J. L., Dupin, L., & Lindberg, P. G. (2024). Post-Stroke Impairments of Manual Dexterity and Finger Proprioception: Their Contribution to Upper Limb Activity Capacity. *Neurorehabilitation and neural repair*, *38*(5), 373–385. https://doi.org/10.1177/15459683241245416（The reason for exclusion: Irrelevant study outcome）
114. Chen, L., Chen, Y., Fu, W. B., Huang, D. F., & Lo, W. L. A. (2022). The Effect of Virtual Reality on Motor Anticipation and Hand Function in Patients with Subacute Stroke: A Randomized Trial on Movement-Related Potential. *Neural plasticity*, *2022*, 7399995. https://doi.org/10.1155/2022/7399995（The reason for exclusion: Non robot-assisted task-oriented training）
115. Wong, Y., Li, C. J., Ada, L., Zhang, T., Månum, G., & Langhammer, B. (2022). Upper Limb Training with a Dynamic Hand Orthosis in Early Subacute Stroke: A Pilot Randomized Trial. *Journal of rehabilitation medicine*, *54*, jrm00279. https://doi.org/10.2340/jrm.v54.2231（The reason for exclusion: Non robot-assisted task-oriented training）
116. Bian, L., Zhang, L., Huang, G., Song, D., Zheng, K., Xu, X., Dai, W., Ren, C., & Shen, Y. (2024). Effects of Priming Intermittent Theta Burst Stimulation With High-Definition tDCS on Upper Limb Function in Hemiparetic Patients With Stroke: A Randomized Controlled Study. *Neurorehabilitation and neural repair*, *38*(4), 268–278. https://doi.org/10.1177/15459683241233259（The reason for exclusion: Non robot-assisted task-oriented training）
117. Chen, Z. J., He, C., Guo, F., Xiong, C. H., & Huang, X. L. (2021). Exoskeleton-Assisted Anthropomorphic Movement Training (EAMT) for Poststroke Upper Limb Rehabilitation: A Pilot Randomized Controlled Trial. *Archives of physical medicine and rehabilitation*, *102*(11), 2074–2082. https://doi.org/10.1016/j.apmr.2021.06.001（The reason for exclusion: duplicate record）
118. Feng, T., Zhao, C., Dong, J., Xue, Z., Cai, F., Li, X., Hu, Z., & Xue, X. (2024). The effect of unaffected side resistance training on upper limb function reconstruction and prevention of sarcopenia in stroke patients: a randomized controlled trial. *Scientific reports*, *14*(1), 25330. https://doi.org/10.1038/s41598-024-76810-2（The reason for exclusion: duplicate record）
119. Wang, L., Wang, S., Zhang, S., Dou, Z., & Guo, T. (2023). Effectiveness and electrophysiological mechanisms of focal vibration on upper limb motor dysfunction in patients with subacute stroke: A randomized controlled trial. *Brain research*, *1809*, 148353. https://doi.org/10.1016/j.brainres.2023.148353（The reason for exclusion: duplicate record）
120. Hu, C., Ti, C. H. E., Yuan, K., Chen, C., Khan, A., Shi, X., Chu, W. C., & Tong, R. K. (2024). Effects of high-definition tDCS targeting individual motor hotspot with EMG-driven robotic hand training on upper extremity motor function: a pilot randomized controlled trial. *Journal of neuroengineering and rehabilitation*, *21*(1), 169. https://doi.org/10.1186/s12984-024-01468-w（The reason for exclusion: duplicate record）
121. Sentandreu-Mañó, T., Tomás, J. M., & Ricardo Salom Terrádez, J. (2021). A randomised clinical trial comparing 35 Hz versus 50 Hz frequency stimulation effects on hand motor recovery in older adults after stroke. *Scientific reports*, *11*(1), 9131. https://doi.org/10.1038/s41598-021-88607-8（The reason for exclusion: Non robot-assisted task-oriented training）
122. Pearse, J. E., Cadwgan, J. E., Wisher, V., Jesmont, C., Mason-Burton, P., Barry, M., Jona James, J., Kelly, S., & Basu, A. P. (2020). Feasibility Trial of Thumb Taping by Parents in Infants with Cerebral Palsy: Brief Report. *Developmental neurorehabilitation*, *23*(1), 50–58. https://doi.org/10.1080/17518423.2019.1566280（The reason for exclusion: Irrelevant study outcome）
123. Shi, X. Q., Ti, C. E., Lu, H. Y., Hu, C. P., Xie, D. S., Yuan, K., Heung, H. L., Leung, T. W., Li, Z., & Tong, R. K. (2024). Task-Oriented Training by a Personalized Electromyography-Driven Soft Robotic Hand in Chronic Stroke: A Randomized Controlled Trial. *Neurorehabilitation and neural repair*, *38*(8), 595–606. https://doi.org/10.1177/15459683241257519（The reason for exclusion: Control group using additional interventions)
124. Rowe, J. B., Chan, V., Ingemanson, M. L., Cramer, S. C., Wolbrecht, E. T., & Reinkensmeyer, D. J. (2017). Robotic Assistance for Training Finger Movement Using a Hebbian Model: A Randomized Controlled Trial. *Neurorehabilitation and neural repair*, *31*(8), 769–780. https://doi.org/10.1177/1545968317721975（The reason for exclusion: duplicate record）
125. Susanto, E. A., Tong, R. K., Ockenfeld, C., & Ho, N. S. (2015). Efficacy of robot-assisted fingers training in chronic stroke survivors: a pilot randomized-controlled trial. *Journal of neuroengineering and rehabilitation*, *12*, 42. https://doi.org/10.1186/s12984-015-0033-5（The reason for exclusion: Non robot-assisted task-oriented training）
126. Wang, L., Zhu, Q. X., Zhong, M. H., Zhou, R. Z., Liu, X. Q., Tang, N. S., Feng, X. C., & Gao, C. F. (2022). Effects of corticospinal tract integrity on upper limb motor function recovery in stroke patients treated with repetitive transcranial magnetic stimulation. *Journal of integrative neuroscience*, *21*(2), 50. https://doi.org/10.31083/j.jin2102050（The reason for exclusion: Non robot-assisted task-oriented training）
127. Brunner, I., Skouen, J. S., Hofstad, H., Aßmus, J., Becker, F., Sanders, A. M., Pallesen, H., Qvist Kristensen, L., Michielsen, M., Thijs, L., & Verheyden, G. (2017). Virtual Reality Training for Upper Extremity in Subacute Stroke (VIRTUES): A multicenter RCT. *Neurology*, *89*(24), 2413–2421. https://doi.org/10.1212/WNL.0000000000004744（The reason for exclusion: Non robot-assisted task-oriented training）
128. Seo, N. J., Ramakrishnan, V., Woodbury, M. L., Bonilha, L., Finetto, C., Schranz, C., Scronce, G., Coupland, K., Blaschke, J., Baker, A., Howard, K., Meinzer, C., Velozo, C. A., & Adams, R. J. (2022). Concomitant sensory stimulation during therapy to enhance hand functional recovery post stroke. *Trials*, *23*(1), 262. https://doi.org/10.1186/s13063-022-06241-9（The reason for exclusion: Irrelevant study outcome）
129. Choi, Y. H., Ku, J., Lim, H., Kim, Y. H., & Paik, N. J. (2016). Mobile game-based virtual reality rehabilitation program for upper limb dysfunction after ischemic stroke. *Restorative neurology and neuroscience*, *34*(3), 455–463. https://doi.org/10.3233/RNN-150626（The reason for exclusion: Non robot-assisted task-oriented training）
130. Wang, T. N., Liang, K. J., Liu, Y. C., Shieh, J. Y., & Chen, H. L. (2023). Effects of Intensive Versus Distributed Constraint-Induced Movement Therapy for Children With Unilateral Cerebral Palsy: A Quasi-Randomized Trial. *Neurorehabilitation and neural repair*, *37*(2-3), 109–118. https://doi.org/10.1177/15459683231162330（The reason for exclusion: Irrelevant study outcome）
131. Zhang, J., Mao, W., Dai, F., Wu, M., Yang, K., Qin, X., He, C., Wang, L., Wang, L., Zhu, C., Han, W., & Wang, Y. (2024). *Tongdu Tiaoshen* acupuncture combined with Bobath rehabilitation training for upper limb spasm after stroke: a randomized controlled trial. 通督调神法针刺联合Bobath康复训练治疗卒中后上肢痉挛：随机对照试验. *Zhongguo zhen jiu = Chinese acupuncture & moxibustion*, *44*(1), 43–47. https://doi.org/10.13703/j.0255-2930.20230711-k0002（The reason for exclusion: duplicate record）
132. Stinear, C. M., Petoe, M. A., Anwar, S., Barber, P. A., & Byblow, W. D. (2014). Bilateral priming accelerates recovery of upper limb function after stroke: a randomized controlled trial. *Stroke*, *45*(1), 205–210. https://doi.org/10.1161/STROKEAHA.113.003537（The reason for exclusion: Irrelevant study outcome）
133. Palomo-Carrión, R., Zuil-Escobar, J. C., Cabrera-Guerra, M., Barreda-Martínez, P., & Martínez-Cepa, C. B. (2022). Terapia en espejo y de observación de la acción en niños con parálisis cerebral espástica unilateral: estudio de viabilidad [Mirror and action observation therapy in children with unilateral spastic cerebral palsy: a feasibility study]. *Revista de neurologia*, *75*(11), 325–332. https://doi.org/10.33588/rn.7511.2022343（The reason for exclusion: Non robot-assisted task-oriented training）
134. Chou, C. H., Lee, Y. Y., Chen, P. C., Leong, C. P., & Huang, Y. C. (2024). Effects of Kinesiotaping on upper limb function and daily activities in subacute or chronic stroke survivors: A randomized control study. *NeuroRehabilitation*, *54*(4), 629–637. https://doi.org/10.3233/NRE-240047（The reason for exclusion: Non robot-assisted task-oriented training）
135. Liu, Y. F., Mao, H. L., Li, Y. J., Zhao, T., Wang, Z. M., Liu, Y. Y., An, J. M., & Huang, L. N. (2023). *Zhongguo zhen jiu = Chinese acupuncture & moxibustion*, *43*(10), 1109–1113. https://doi.org/10.13703/j.0255-2930.20230131-0002（The reason for exclusion: Non robot-assisted task-oriented training）
136. Radajewska, A., Opara, J. A., Kucio, C., Błaszczyszyn, M., Mehlich, K., & Szczygiel, J. (2013). The effects of mirror therapy on arm and hand function in subacute stroke in patients. *International journal of rehabilitation research. Internationale Zeitschrift fur Rehabilitationsforschung. Revue internationale de recherches de readaptation*, *36*(3), 268–274. https://doi.org/10.1097/MRR.0b013e3283606218 reason for exclusion: Non robot-assisted task-oriented training）
137. Xu, L., Zhang, Z., Xu, W., Liu, H., Han, X., & Wang, M. (2024). Interactive scalp acupuncture combined with suspension digital occupational therapy for upper limb motor dysfunction after stroke: a multi-center randomized controlled trial. 互动式头针结合悬吊下数字作业疗法治疗脑卒中后上肢运动功能障碍：多中心随机对照试验. *Zhongguo zhen jiu = Chinese acupuncture & moxibustion*, *44*(2), 129–133. https://doi.org/10.13703/j.0255-2930.20230507-k0001（The reason for exclusion: duplicate record）
138. Jiang, W., Wang, S., Wu, Q., & Li, X. (2021). Effects of Self-Assisted Shoulder Elevation of the Affected Side Combined with Balance Training on Associated Reactions of Upper Limb and Walking Function in Chronic Stroke Patients: A Randomized Controlled Trial. *Medical science monitor : international medical journal of experimental and clinical research*, *27*, e928549. https://doi.org/10.12659/MSM.928549（The reason for exclusion: Non robot-assisted task-oriented training）
139. Vanoglio, F., Bernocchi, P., Mulè, C., Garofali, F., Mora, C., Taveggia, G., Scalvini, S., & Luisa, A. (2017). Feasibility and efficacy of a robotic device for hand rehabilitation in hemiplegic stroke patients: a randomized pilot controlled study. *Clinical rehabilitation*, *31*(3), 351–360. https://doi.org/10.1177/0269215516642606（The reason for exclusion: Control group using additional interventions)
140. Straudi, S., Baroni, A., Mele, S., Craighero, L., Manfredini, F., Lamberti, N., Maietti, E., & Basaglia, N. (2020). Effects of a Robot-Assisted Arm Training Plus Hand Functional Electrical Stimulation on Recovery After Stroke: A Randomized Clinical Trial. *Archives of physical medicine and rehabilitation*, *101*(2), 309–316. https://doi.org/10.1016/j.apmr.2019.09.016（The reason for exclusion: duplicate record）
141. Nakipoğlu Yuzer, G. F., Köse Dönmez, B., & Özgirgin, N. (2017). A Randomized Controlled Study: Effectiveness of Functional Electrical Stimulation on Wrist and Finger Flexor Spasticity in Hemiplegia. *Journal of stroke and cerebrovascular diseases : the official journal of National Stroke Association*, *26*(7), 1467–1471. https://doi.org/10.1016/j.jstrokecerebrovasdis.2017.03.011（The reason for exclusion: Non robot-assisted task-oriented training）
142. Renner, C. I. E., Brendel, C., & Hummelsheim, H. (2020). Bilateral Arm Training vs Unilateral Arm Training for Severely Affected Patients With Stroke: Exploratory Single-Blinded Randomized Controlled Trial. *Archives of physical medicine and rehabilitation*, *101*(7), 1120–1130. https://doi.org/10.1016/j.apmr.2020.02.007（The reason for exclusion: Irrelevant study outcome）
143. Cordo, P., Wolf, S., Lou, J. S., Bogey, R., Stevenson, M., Hayes, J., & Roth, E. (2013). Treatment of severe hand impairment following stroke by combining assisted movement, muscle vibration, and biofeedback. *Journal of neurologic physical therapy : JNPT*, *37*(4), 194–203. https://doi.org/10.1097/NPT.0000000000000023（The reason for exclusion: duplicate record）
144. Sgandurra, G., Ferrari, A., Cossu, G., Guzzetta, A., Fogassi, L., & Cioni, G. (2013). Randomized trial of observation and execution of upper extremity actions versus action alone in children with unilateral cerebral palsy. *Neurorehabilitation and neural repair*, *27*(9), 808–815. https://doi.org/10.1177/1545968313497101（The reason for exclusion: Non robot-assisted task-oriented training）
145. Hsu, H. Y., Kuan, T. S., Tsai, C. L., Wu, P. T., Kuo, Y. L., Su, F. C., & Kuo, L. C. (2021). Effect of a Novel Perturbation-Based Pinch Task Training on Sensorimotor Performance of Upper Extremity for Patients With Chronic Stroke: A Pilot Randomized Controlled Trial. *Archives of physical medicine and rehabilitation*, *102*(5), 811–818. https://doi.org/10.1016/j.apmr.2020.11.004（The reason for exclusion: Non robot-assisted task-oriented training）
146. Calabrò, R. S., Accorinti, M., Porcari, B., Carioti, L., Ciatto, L., Billeri, L., Andronaco, V. A., Galletti, F., Filoni, S., & Naro, A. (2019). Does hand robotic rehabilitation improve motor function by rebalancing interhemispheric connectivity after chronic stroke? Encouraging data from a randomised-clinical-trial. *Clinical neurophysiology : official journal of the International Federation of Clinical Neurophysiology*, *130*(5), 767–780. https://doi.org/10.1016/j.clinph.2019.02.013（The reason for exclusion: duplicate record）
147. Kwakkel, G., Winters, C., van Wegen, E. E., Nijland, R. H., van Kuijk, A. A., Visser-Meily, A., de Groot, J., de Vlugt, E., Arendzen, J. H., Geurts, A. C., Meskers, C. G., & EXPLICIT-Stroke Consortium (2016). Effects of Unilateral Upper Limb Training in Two Distinct Prognostic Groups Early After Stroke: The EXPLICIT-Stroke Randomized Clinical Trial. *Neurorehabilitation and neural repair*, *30*(9), 804–816. https://doi.org/10.1177/1545968315624784（The reason for exclusion: Non robot-assisted task-oriented training）
148. Giray, E., Gencer Atalay, K., Eren, N., Gündüz, O. H., & Karadag-Saygi, E. (2020). Effects of dynamic lycra orthosis as an adjunct to rehabilitation after botulinum toxin-A injection of the upper-limb in adults following stroke: A single-blinded randomized controlled pilot study. *Topics in stroke rehabilitation*, *27*(6), 473–481. https://doi.org/10.1080/10749357.2019.1704371（The reason for exclusion: Control group using additional interventions)

Sakzewski, L., Ziviani, J., Abbott, D. F., Macdonell, R. A., Jackson, G. D., & Boyd, R. N. (2011). Randomized trial of constraint-induced movement therapy and bimanual training on activity outcomes for children with congenital hemiplegia. *Developmental medicine and child neurology*, *53*(4), 313–320. https://doi.org/10.1111/j.1469-8749.2010.03859.x（The reason for exclusion: Irrelevant study outcome）

1. Lannin, N. A., Cusick, A., Hills, C., Kinnear, B., Vogel, K., Matthews, K., & Bowring, G. (2016). Upper limb motor training using a Saebo™ orthosis is feasible for increasing task-specific practice in hospital after stroke. *Australian occupational therapy journal*, *63*(6), 364–372. https://doi.org/10.1111/1440-1630.12330（The reason for exclusion: Non robot-assisted task-oriented training）
2. Bang, D. H., Shin, W. S., & Choi, H. S. (2018). Effects of modified constraint-induced movement therapy with trunk restraint in early stroke patients: A single-blinded, randomized, controlled, pilot trial. *NeuroRehabilitation*, *42*(1), 29–35. https://doi.org/10.3233/NRE-172176（The reason for exclusion: Control group using additional interventions)
3. van Delden, A. L., Peper, C. L., Nienhuys, K. N., Zijp, N. I., Beek, P. J., & Kwakkel, G. (2013). Unilateral versus bilateral upper limb training after stroke: the Upper Limb Training After Stroke clinical trial. *Stroke*, *44*(9), 2613–2616. https://doi.org/10.1161/STROKEAHA.113.001969（The reason for exclusion: Irrelevant study outcome）
4. Khallaf, M. E., Ameer, M. A., & Fayed, E. E. (2017). Effect of task specific training and wrist-fingers extension splint on hand joints range of motion and function after stroke. *NeuroRehabilitation*, *41*(2), 437–444. https://doi.org/10.3233/NRE-162128（The reason for exclusion: Non robot-assisted task-oriented training）
5. Imms, C., Wallen, M., Elliott, C., Hoare, B., Randall, M., Greaves, S., Adair, B., Bradshaw, E., Carter, R., Orsini, F., Shih, S. T., & Reddihough, D. (2016). Minimising impairment: Protocol for a multicentre randomised controlled trial of upper limb orthoses for children with cerebral palsy. *BMC pediatrics*, *16*, 70. https://doi.org/10.1186/s12887-016-0608-8（The reason for exclusion: duplicate record）
6. Fu, J., Zeng, M., Shen, F., Cui, Y., Zhu, M., Gu, X., & Sun, Y. (2017). Effects of action observation therapy on upper extremity function, daily activities and motion evoked potential in cerebral infarction patients. *Medicine*, *96*(42), e8080. https://doi.org/10.1097/MD.0000000000008080（The reason for exclusion: Non robot-assisted task-oriented training）
7. Zhou, Y. X., Xia, Y., Huang, J., Wang, H. P., Bao, X. L., Bi, Z. Y., Chen, X. B., Gao, Y. J., Lü, X. Y., & Wang, Z. G. (2017). Electromyographic bridge for promoting the recovery of hand movements in subacute stroke patients: A randomized controlled trial. *Journal of rehabilitation medicine*, *49*(8), 629–636. https://doi.org/10.2340/16501977-2256（The reason for exclusion: Non robot-assisted task-oriented training）
8. Nijland, R., van Wegen, E., van der Krogt, H., Bakker, C., Buma, F., Klomp, A., van Kordelaar, J., Kwakkel, G., & EXPLICIT-stroke consortium (2013). Characterizing the protocol for early modified constraint-induced movement therapy in the EXPLICIT-stroke trial. *Physiotherapy research international : the journal for researchers and clinicians in physical therapy*, *18*(1), 1–15. https://doi.org/10.1002/pri.1521（The reason for exclusion: Non robot-assisted task-oriented training）
9. Kirac-Unal, Z., Gencay-Can, A., Karaca-Umay, E., & Cakci, F. A. (2019). The effect of task-oriented electromyography-triggered electrical stimulation of the paretic wrist extensors on upper limb motor function early after stroke: a pilot randomized controlled trial. *International journal of rehabilitation research. Internationale Zeitschrift fur Rehabilitationsforschung. Revue internationale de recherches de readaptation*, *42*(1), 74–81. https://doi.org/10.1097/MRR.0000000000000333（The reason for exclusion: Irrelevant study outcome）
10. Chiu, H. C., Ada, L., & Lee, H. M. (2014). Upper limb training using Wii Sports Resort for children with hemiplegic cerebral palsy: a randomized, single-blind trial. *Clinical rehabilitation*, *28*(10), 1015–1024. https://doi.org/10.1177/0269215514533709（The reason for exclusion: Non robot-assisted task-oriented training）
11. Chae, J., Harley, M. Y., Hisel, T. Z., Corrigan, C. M., Demchak, J. A., Wong, Y. T., & Fang, Z. P. (2009). Intramuscular electrical stimulation for upper limb recovery in chronic hemiparesis: an exploratory randomized clinical trial. *Neurorehabilitation and neural repair*, *23*(6), 569–578. https://doi.org/10.1177/1545968308328729（The reason for exclusion: Non robot-assisted task-oriented training）
12. Lannin, N. A., Horsley, S. A., Herbert, R., McCluskey, A., & Cusick, A. (2003). Splinting the hand in the functional position after brain impairment: a randomized, controlled trial. *Archives of physical medicine and rehabilitation*, *84*(2), 297–302. https://doi.org/10.1053/apmr.2003.50031（The reason for exclusion: Non robot-assisted task-oriented training）
13. Prange-Lasonder, G. B., Radder, B., Kottink, A. I. R., Melendez-Calderon, A., Buurke, J. H., & Rietman, J. S. (2017). Applying a soft-robotic glove as assistive device and training tool with games to support hand function after stroke: Preliminary results on feasibility and potential clinical impact. *IEEE ... International Conference on Rehabilitation Robotics : [proceedings]*, *2017*, 1401–1406. https://doi.org/10.1109/ICORR.2017.8009444（The reason for exclusion: duplicate record）
14. Bakker, C. D., Massa, M., Daffertshofer, A., Pasman, J. W., van Kuijk, A. A., Kwakkel, G., & Stegeman, D. F. (2019). The addition of the MEP amplitude of finger extension muscles to clinical predictors of hand function after stroke: A prospective cohort study. *Restorative neurology and neuroscience*, *37*(5), 445–456. https://doi.org/10.3233/RNN-180890（The reason for exclusion: Non robot-assisted task-oriented training）
15. Bonzano, L., Pedullà, L., Tacchino, A., Brichetto, G., Battaglia, M. A., Mancardi, G. L., & Bove, M. (2019). Upper limb motor training based on task-oriented exercises induces functional brain reorganization in patients with multiple sclerosis. *Neuroscience*, *410*, 150–159. https://doi.org/10.1016/j.neuroscience.2019.05.004（The reason for exclusion: duplicate record）
16. Chae, J., Bethoux, F., Bohine, T., Dobos, L., Davis, T., & Friedl, A. (1998). Neuromuscular stimulation for upper extremity motor and functional recovery in acute hemiplegia. *Stroke*, *29*(5), 975–979. https://doi.org/10.1161/01.str.29.5.975（The reason for exclusion: Non robot-assisted task-oriented training）
17. Zhu, J. M., Zhuang, R., He, J., Wang, X. X., Wang, H., & Zhu, H. Y. (2020). *Zhongguo zhen jiu = Chinese acupuncture & moxibustion*, *40*(7), 697–701. <https://doi.org/10.13703/j.0255-2930.20190531-k0005>（The reason for exclusion: Non robot-assisted task-oriented training）
18. Vermeulen, G. M., Spekreijse, K. R., Slijper, H., Feitz, R., Hovius, S. E., & Selles, R. W. (2014). Comparison of arthroplasties with or without bone tunnel creation for thumb basal joint arthritis: a randomized controlled trial. *The Journal of hand surgery*, *39*(9), 1692–1698. <https://doi.org/10.1016/j.jhsa.2014.04.044>（The reason for exclusion: Non robot-assisted task-oriented training）
19. Sorinola, I. O., Bateman, R. W., & Mamy, K. (2012). Effect of somatosensory stimulation of two and three nerves on upper limb function in healthy individuals. *Physiotherapy research international : the journal for researchers and clinicians in physical therapy*, *17*(2), 74–79. <https://doi.org/10.1002/pri.515>（The reason for exclusion: Non robot-assisted task-oriented training）
20. Zhu, J. M., Zhuang, R., He, J., Wang, X. X., Wang, H., & Zhu, H. Y. (2020). *Zhongguo zhen jiu = Chinese acupuncture & moxibustion*, *40*(7), 697–701. <https://doi.org/10.13703/j.0255-2930.20190531-k0005>（The reason for exclusion: duplicate record）
21. Yıldızgören, M. T., Nakipoğlu Yüzer, G. F., Ekiz, T., & Özgirgin, N. (2014). Effects of neuromuscular electrical stimulation on the wrist and finger flexor spasticity and hand functions in cerebral palsy. *Pediatric neurology*, *51*(3), 360–364. <https://doi.org/10.1016/j.pediatrneurol.2014.05.009>（The reason for exclusion: duplicate record）
22. Lin, C. H., Chou, L. W., Luo, H. J., Tsai, P. Y., Lieu, F. K., Chiang, S. L., & Sung, W. H. (2015). Effects of Computer-Aided Interlimb Force Coupling Training on Paretic Hand and Arm Motor Control following Chronic Stroke: A Randomized Controlled Trial. *PloS one*, *10*(7), e0131048. <https://doi.org/10.1371/journal.pone.0131048>（The reason for exclusion: duplicate record）
23. Kwon, T. G., Park, E., Kang, C., Chang, W. H., & Kim, Y. H. (2016). The effects of combined repetitive transcranial magnetic stimulation and transcranial direct current stimulation on motor function in patients with stroke. *Restorative neurology and neuroscience*, *34*(6), 915–923. <https://doi.org/10.3233/RNN-160654>（The reason for exclusion: duplicate record）
24. Villafañe, J. H., Cleland, J. A., & Fernández-de-Las-Peñas, C. (2013). The effectiveness of a manual therapy and exercise protocol in patients with thumb carpometacarpal osteoarthritis: a randomized controlled trial. *The Journal of orthopaedic and sports physical therapy*, *43*(4), 204–213. <https://doi.org/10.2519/jospt.2013.4524>（The reason for exclusion: duplicate record）
25. Fluet, G. G., Merians, A. S., Qiu, Q., Davidow, A., & Adamovich, S. V. (2014). Comparing integrated training of the hand and arm with isolated training of the same effectors in persons with stroke using haptically rendered virtual environments, a randomized clinical trial. *Journal of neuroengineering and rehabilitation*, *11*, 126. <https://doi.org/10.1186/1743-0003-11-126>（The reason for exclusion: duplicate record）
26. Hwang, C. H., Seong, J. W., & Son, D. S. (2012). Individual finger synchronized robot-assisted hand rehabilitation in subacute to chronic stroke: a prospective randomized clinical trial of efficacy. *Clinical rehabilitation*, *26*(8), 696–704. <https://doi.org/10.1177/0269215511431473>（The reason for exclusion: duplicate record）
27. Bai, Y. L., Hu, Y. S., Wu, Y., Zhu, Y. L., Zhang, B., Jiang, C. Y., Sun, L. M., & Fan, W. K. (2014). Long-term three-stage rehabilitation intervention alleviates spasticity of the elbows, fingers, and plantar flexors and improves activities of daily living in ischemic stroke patients: a randomized, controlled trial. *Neuroreport*, *25*(13), 998–1005. <https://doi.org/10.1097/WNR.0000000000000194>（The reason for exclusion: duplicate record）
28. Ciatto, L., Dauccio, B., Tavilla, G., Bartolomeo, S., Lo Buono, V., De Cola, M. C., Quartarone, A., Pastura, C., Cellini, R., Bonanno, M., & Calabrò, R. S. (2024). Improving manual dexterity using ergonomic wearable glove in patients with multiple sclerosis: A quasi-randomized clinical trial. *Multiple sclerosis and related disorders*, *92*, 105938. <https://doi.org/10.1016/j.msard.2024.105938>（The reason for exclusion: Non robot-assisted task-oriented training）
29. Choi, Y., Gordon, J., Park, H., & Schweighofer, N. (2011). Feasibility of the adaptive and automatic presentation of tasks (ADAPT) system for rehabilitation of upper extremity function post-stroke. *Journal of neuroengineering and rehabilitation*, *8*, 42. <https://doi.org/10.1186/1743-0003-8-42>（The reason for exclusion: Non robot-assisted task-oriented training）
30. Huang, T. Y., Pan, L. H., Yang, W. W., Huang, L. Y., Sun, P. C., & Chen, C. S. (2019). Biomechanical Evaluation of Three-Dimensional Printed Dynamic Hand Device for Patients With Chronic Stroke. *IEEE transactions on neural systems and rehabilitation engineering : a publication of the IEEE Engineering in Medicine and Biology Society*, *27*(6), 1246–1252. <https://doi.org/10.1109/TNSRE.2019.2915260>（The reason for exclusion: Non robot-assisted task-oriented training）
31. Friedrich, D. T., Dürselen, L., Mayer, B., Hacker, S., Schall, F., Hahn, J., Hoffmann, T. K., Schuler, P. J., & Greve, J. (2018). Features of haptic and tactile feedback in TORS-a comparison of available surgical systems. *Journal of robotic surgery*, *12*(1), 103–108. https://doi.org/10.1007/s11701-017-0702-4（The reason for exclusion: Non robot-assisted task-oriented training）
32. Thorsen, R., Cortesi, M., Jonsdottir, J., Carpinella, I., Morelli, D., Casiraghi, A., Puglia, M., Diverio, M., & Ferrarin, M. (2013). Myoelectrically driven functional electrical stimulation may increase motor recovery of upper limb in poststroke subjects: a randomized controlled pilot study. *Journal of rehabilitation research and development*, *50*(6), 785–794. <https://doi.org/10.1682/JRRD.2012.07.0123>（The reason for exclusion: Non robot-assisted task-oriented training）
33. Iosa, M., Morone, G., Ragaglini, M. R., Fusco, A., & Paolucci, S. (2013). Motor strategies and bilateral transfer in sensorimotor learning of patients with subacute stroke and healthy subjects. A randomized controlled trial. *European journal of physical and rehabilitation medicine*, *49*(3), 291–299.（The reason for exclusion: Non robot-assisted task-oriented training）
34. Winters, C., Kwakkel, G., Nijland, R., van Wegen, E., & EXPLICIT-stroke consortium (2016). When Does Return of Voluntary Finger Extension Occur Post-Stroke? A Prospective Cohort Study. *PloS one*, *11*(8), e0160528. <https://doi.org/10.1371/journal.pone.0160528>（The reason for exclusion: Non robot-assisted task-oriented training）
35. Barrett, P. C., Hackley, D. T., Yu-Shan, A. A., Shumate, T. G., Larson, K. G., Deneault, C. R., Bravo, C. J., Peterman, N. J., & Apel, P. J. (2024). Provision of a Home-Based Video-Assisted Therapy Program Is Noninferior to In-Person Hand Therapy After Thumb Carpometacarpal Arthroplasty. *The Journal of bone and joint surgery. American volume*, *106*(8), 674–680. <https://doi.org/10.2106/JBJS.23.00597>（The reason for exclusion: Non robot-assisted task-oriented training）
36. Kutner, N. G., Zhang, R., Butler, A. J., Wolf, S. L., & Alberts, J. L. (2010). Quality-of-life change associated with robotic-assisted therapy to improve hand motor function in patients with subacute stroke: a randomized clinical trial. *Physical therapy*, *90*(4), 493–504. https://doi.org/10.2522/ptj.20090160（The reason for exclusion: duplicate record）
37. Tarkka, I. M., Pitkänen, K., Popovic, D. B., Vanninen, R., & Könönen, M. (2011). Functional electrical therapy for hemiparesis alleviates disability and enhances neuroplasticity. *The Tohoku journal of experimental medicine*, *225*(1), 71–76. https://doi.org/10.1620/tjem.225.71（The reason for exclusion: Non robot-assisted task-oriented training）
38. Germanotta, M., Gower, V., Papadopoulou, D., Cruciani, A., Pecchioli, C., Mosca, R., Speranza, G., Falsini, C., Cecchi, F., Vannetti, F., Montesano, A., Galeri, S., Gramatica, F., Aprile, I., & FDG Robotic Rehabilitation Group (2020). Reliability, validity and discriminant ability of a robotic device for finger training in patients with subacute stroke. *Journal of neuroengineering and rehabilitation*, *17*(1), 1. https://doi.org/10.1186/s12984-019-0634-5（The reason for exclusion: Irrelevant study outcome）
39. Silva, F. C., da Silva, R. V. T., Meireles, S. M., Fernandes, A. D. R. C., & Natour, J. (2024). Daytime Functional Usage Versus Night-Time Wearing: Identifying the Optimal Wearing Regimen for a Custom-Made Orthosis in the Treatment of Trapeziometacarpal Osteoarthritis. *Archives of physical medicine and rehabilitation*, *105*(10), 1837–1845. https://doi.org/10.1016/j.apmr.2024.06.013（The reason for exclusion: Non robot-assisted task-oriented training）
40. Carey, J. R., Durfee, W. K., Bhatt, E., Nagpal, A., Weinstein, S. A., Anderson, K. M., & Lewis, S. M. (2007). Comparison of finger tracking versus simple movement training via telerehabilitation to alter hand function and cortical reorganization after stroke. *Neurorehabilitation and neural repair*, *21*(3), 216–232. <https://doi.org/10.1177/1545968306292381>（The reason for exclusion: Non robot-assisted task-oriented training）
41. van den Noort, J. C., Verhagen, R., van Dijk, K. J., Veltink, P. H., Vos, M. C. P. M., de Bie, R. M. A., Bour, L. J., & Heida, C. T. (2017). Quantification of Hand Motor Symptoms in Parkinson's Disease: A Proof-of-Principle Study Using Inertial and Force Sensors. *Annals of biomedical engineering*, *45*(10), 2423–2436. <https://doi.org/10.1007/s10439-017-1881-x>（The reason for exclusion: Non robot-assisted task-oriented training）
42. Wei, Y., Chen, J., Fang, R., Liu, J., Feng, M., Du, H., Wang, M., Abulihaiti, R., Ling, H., & Huang, F. (2024). Investigating the Effect of Different Types of Exercise on Upper Limb Functional Recovery in Patients with Right Hemisphere Damage Based on fNIRS. *Journal of visualized experiments : JoVE*, (204), 10.3791/65996. <https://doi.org/10.3791/65996>（The reason for exclusion: Non robot-assisted task-oriented training）
43. Kilbreath, S. L., Crosbie, J., Canning, C. G., & Lee, M. J. (2006). Inter-limb coordination in bimanual reach-to-grasp following stroke. *Disability and rehabilitation*, *28*(23), 1435–1443. （The reason for exclusion: Non robot-assisted task-oriented training）https://doi.org/10.1080/09638280600638307（The reason for exclusion: Non robot-assisted task-oriented training）
44. Hesse, S., Kuhlmann, H., Wilk, J., Tomelleri, C., & Kirker, S. G. (2008). A new electromechanical trainer for sensorimotor rehabilitation of paralysed fingers: a case series in chronic and acute stroke patients. *Journal of neuroengineering and rehabilitation*, *5*, 21. https://doi.org/10.1186/1743-0003-5-21（The reason for exclusion: duplicate record）
45. Carey, J. R., Kimberley, T. J., Lewis, S. M., Auerbach, E. J., Dorsey, L., Rundquist, P., & Ugurbil, K. (2002). Analysis of fMRI and finger tracking training in subjects with chronic stroke. *Brain : a journal of neurology*, *125*(Pt 4), 773–788. <https://doi.org/10.1093/brain/awf091>（The reason for exclusion: Non robot-assisted task-oriented training）
46. Muller, C. O., Metais, A., Boublay, N., Breuil, C., Daligault, S., Di Rienzo, F., Guillot, A., Collet, C., Krolak-Salmon, P., & Saimpont, A. (2024). Anodal transcranial direct current stimulation does not enhance the effects of motor imagery training of a sequential finger-tapping task in young adults. *Journal of sports sciences*, *42*(5), 392–403. <https://doi.org/10.1080/02640414.2024.2328418>（The reason for exclusion: Non robot-assisted task-oriented training）
47. Bhatt, E., Nagpal, A., Greer, K. H., Grunewald, T. K., Steele, J. L., Wiemiller, J. W., Lewis, S. M., & Carey, J. R. (2007). Effect of finger tracking combined with electrical stimulation on brain reorganization and hand function in subjects with stroke. *Experimental brain research*, *182*(4), 435–447. <https://doi.org/10.1007/s00221-007-1001-5>（The reason for exclusion: Non robot-assisted task-oriented training）
48. Fischer, H. C., Stubblefield, K., Kline, T., Luo, X., Kenyon, R. V., & Kamper, D. G. (2007). Hand rehabilitation following stroke: a pilot study of assisted finger extension training in a virtual environment. *Topics in stroke rehabilitation*, *14*(1), 1–12. https://doi.org/10.1310/tsr1401-1（The reason for exclusion: duplicate record）
49. Jahangir, A. W., Tan, H. J., Norlinah, M. I., Nafisah, W. Y., Ramesh, S., Hamidon, B. B., & Raymond, A. A. (2007). Intramuscular injection of botulinum toxin for the treatment of wrist and finger spasticity after stroke. *The Medical journal of Malaysia*, *62*(4), 319–322.（The reason for exclusion: Non robot-assisted task-oriented training）
50. Cauraugh, J., Light, K., Kim, S., Thigpen, M., & Behrman, A. (2000). Chronic motor dysfunction after stroke: recovering wrist and finger extension by electromyography-triggered neuromuscular stimulation. *Stroke*, *31*(6), 1360–1364. <https://doi.org/10.1161/01.str.31.6.1360>（The reason for exclusion: Non robot-assisted task-oriented training）
51. Kim, D. G., Cho, Y. W., Hong, J. H., Song, J. C., Chung, H. A., Bai, D. S., Lee, C. H., & Jang, S. H. (2008). Effect of constraint-induced movement therapy with modified opposition restriction orthosis in chronic hemiparetic patients with stroke. *NeuroRehabilitation*, *23*(3), 239–244.（The reason for exclusion: duplicate record）
52. Seniów, J., Bilik, M., Leśniak, M., Waldowski, K., Iwański, S., & Członkowska, A. (2012). Transcranial magnetic stimulation combined with physiotherapy in rehabilitation of poststroke hemiparesis: a randomized, double-blind, placebo-controlled study. *Neurorehabilitation and neural repair*, *26*(9), 1072–1079. <https://doi.org/10.1177/1545968312445635>（The reason for exclusion: duplicate record）
53. Thielbar, K. O., Lord, T. J., Fischer, H. C., Lazzaro, E. C., Barth, K. C., Stoykov, M. E., Triandafilou, K. M., & Kamper, D. G. (2014). Training finger individuation with a mechatronic-virtual reality system leads to improved fine motor control post-stroke. *Journal of neuroengineering and rehabilitation*, *11*, 171. <https://doi.org/10.1186/1743-0003-11-171>（The reason for exclusion: duplicate record）
54. Singer, B. J., Vallence, A. M., Cleary, S., Cooper, I., & Loftus, A. M. (2013). The effect of EMG triggered electrical stimulation plus task practice on arm function in chronic stroke patients with moderate-severe arm deficits. *Restorative neurology and neuroscience*, *31*(6), 681–691. <https://doi.org/10.3233/RNN-130319>（The reason for exclusion: duplicate record）
55. Dolganov, M. V., & Karpova, M. I. (2019). Virtual'naia real'nost' pri narushenii funktsii ruki: osobennosti primeneniia v ostrom periode insul'ta [Virtual reality in upper extremity dysfunction: specific features of usage in acute stroke]. *Voprosy kurortologii, fizioterapii, i lechebnoi fizicheskoi kultury*, *96*(5), 19–28. <https://doi.org/10.17116/kurort20199605119>（The reason for exclusion: duplicate record）
56. Trombly, C. A., Thayer-Nason, L., Bliss, G., Girard, C. A., Lyrist, L. A., & Brexa-Hooson, A. (1986). The effectiveness of therapy in improving finger extension in stroke patients. *The American journal of occupational therapy : official publication of the American Occupational Therapy Association*, *40*(9), 612–617. <https://doi.org/10.5014/ajot.40.9.612>（The reason for exclusion: duplicate record）

Klamroth-Marganska V. (2018). Stroke Rehabilitation: Therapy Robots and Assistive Devices. *Advances in experimental medicine and biology*, *1065*, 579–587. https://doi.org/10.1007/978-3-319-77932-4_35（The reason for exclusion: review）

1. Takebayashi, T., Takahashi, K., Amano, S., Gosho, M., Sakai, M., Hashimoto, K., Hachisuka, K., Uchiyama, Y., & Domen, K. (2022). Robot-Assisted Training as Self-Training for Upper-Limb Hemiplegia in Chronic Stroke: A Randomized Controlled Trial. *Stroke*, *53*(7), 2182–2191. https://doi.org/10.1161/STROKEAHA.121.037260（The reason for exclusion: duplicate record）
2. Moucheboeuf, G., Griffier, R., Gasq, D., Glize, B., Bouyer, L., Dehail, P., & Cassoudesalle, H. (2020). Effects of robotic gait training after stroke: A meta-analysis. *Annals of physical and rehabilitation medicine*, *63*(6), 518–534. <https://doi.org/10.1016/j.rehab.2020.02.008>（The reason for exclusion: review）

Chien, W. T., Chong, Y. Y., Tse, M. K., Chien, C. W., & Cheng, H. Y. (2020). Robot-assisted therapy for upper-limb rehabilitation in subacute stroke patients: A systematic review and meta-analysis. *Brain and behavior*, *10*(8), e01742. https://doi.org/10.1002/brb3.1742（The reason for exclusion: review）

1. Lee, J., Kim, D. Y., Lee, S. H., Kim, J. H., Kim, D. Y., Lim, K. B., & Yoo, J. (2023). End-effector lower limb robot-assisted gait training effects in subacute stroke patients: A randomized controlled pilot trial. *Medicine*, *102*(42), e35568. https://doi.org/10.1097/MD.0000000000035568（The reason for exclusion: duplicate record）
2. Chen, Y. W., Li, K. Y., Lin, C. H., Hung, P. H., Lai, H. T., & Wu, C. Y. (2023). The effect of sequential combination of mirror therapy and robot-assisted therapy on motor function, daily function, and self-efficacy after stroke. *Scientific reports*, *13*(1), 16841. https://doi.org/10.1038/s41598-023-43981-3（The reason for exclusion: duplicate record）
3. Zhang, B., Wong, K. P., Kang, R., Fu, S., Qin, J., & Xiao, Q. (2023). Efficacy of Robot-Assisted and Virtual Reality Interventions on Balance, Gait, and Daily Function in Patients With Stroke: A Systematic Review and Network Meta-analysis. *Archives of physical medicine and rehabilitation*, *104*(10), 1711–1719. <https://doi.org/10.1016/j.apmr.2023.04.005>（The reason for exclusion: review）
4. Yang, X., Shi, X., Xue, X., & Deng, Z. (2023). Efficacy of Robot-Assisted Training on Rehabilitation of Upper Limb Function in Patients With Stroke: A Systematic Review and Meta-analysis. *Archives of physical medicine and rehabilitation*, *104*(9), 1498–1513. <https://doi.org/10.1016/j.apmr.2023.02.004>（The reason for exclusion: review）
5. Iwamoto, Y., Imura, T., Suzukawa, T., Fukuyama, H., Ishii, T., Taki, S., Imada, N., Shibukawa, M., Inagawa, T., Araki, H., & Araki, O. (2019). Combination of Exoskeletal Upper Limb Robot and Occupational Therapy Improve Activities of Daily Living Function in Acute Stroke Patients. *Journal of stroke and cerebrovascular diseases : the official journal of National Stroke Association*, *28*(7), 2018–2025. <https://doi.org/10.1016/j.jstrokecerebrovasdis.2019.03.006>（The reason for exclusion: duplicate record）
6. Akıncı, M., Burak, M., Yaşar, E., & Kılıç, R. T. (2023). The effects of Robot-assisted gait training and virtual reality on balance and gait in stroke survivors: A randomized controlled trial. *Gait & posture*, *103*, 215–222. <https://doi.org/10.1016/j.gaitpost.2023.05.013>（The reason for exclusion: duplicate record）
7. Sale, P., Franceschini, M., Mazzoleni, S., Palma, E., Agosti, M., & Posteraro, F. (2014). Effects of upper limb robot-assisted therapy on motor recovery in subacute stroke patients. *Journal of neuroengineering and rehabilitation*, *11*, 104. <https://doi.org/10.1186/1743-0003-11-104>（The reason for exclusion: duplicate record）
8. Schrader, M., Sterr, A., Kettlitz, R., Wohlmeiner, A., Buschfort, R., Dohle, C., & Bamborschke, S. (2022). The effect of mirror therapy can be improved by simultaneous robotic assistance. *Restorative neurology and neuroscience*, *40*(3), 185–194. <https://doi.org/10.3233/RNN-221263>（The reason for exclusion: duplicate record）
9. Inoue, S., Otaka, Y., Kumagai, M., Sugasawa, M., Mori, N., & Kondo, K. (2022). Effects of Balance Exercise Assist Robot training for patients with hemiparetic stroke: a randomized controlled trial. *Journal of neuroengineering and rehabilitation*, *19*(1), 12. <https://doi.org/10.1186/s12984-022-00989-6>（The reason for exclusion: duplicate record）
10. Ranzani, R., Lambercy, O., Metzger, J. C., Califfi, A., Regazzi, S., Dinacci, D., Petrillo, C., Rossi, P., Conti, F. M., & Gassert, R. (2020). Neurocognitive robot-assisted rehabilitation of hand function: a randomized control trial on motor recovery in subacute stroke. *Journal of neuroengineering and rehabilitation*, *17*(1), 115. <https://doi.org/10.1186/s12984-020-00746-7>（The reason for exclusion: duplicate record）
11. Lee, H. C., Kuo, F. L., Lin, Y. N., Liou, T. H., Lin, J. C., & Huang, S. W. (2021). Effects of Robot-Assisted Rehabilitation on Hand Function of People With Stroke: A Randomized, Crossover-Controlled, Assessor-Blinded Study. *The American journal of occupational therapy : official publication of the American Occupational Therapy Association*, *75*(1), 7501205020p1–7501205020p11. <https://doi.org/10.5014/ajot.2021.038232>（The reason for exclusion: duplicate record）
12. Novak, D., & Riener, R. (2020). Sensor Fusion in Assistive and Rehabilitation Robotics. *Sensors (Basel, Switzerland)*, *20*(18), 5235. <https://doi.org/10.3390/s20185235>（The reason for exclusion: Non robot-assisted task-oriented training）
13. Dehem, S., Gilliaux, M., Stoquart, G., Detrembleur, C., Jacquemin, G., Palumbo, S., Frederick, A., & Lejeune, T. (2019). Effectiveness of upper-limb robotic-assisted therapy in the early rehabilitation phase after stroke: A single-blind, randomised, controlled trial. *Annals of physical and rehabilitation medicine*, *62*(5), 313–320. <https://doi.org/10.1016/j.rehab.2019.04.002>（The reason for exclusion: duplicate record）
14. Castelli E. (2023). Robotic Rehabilitation in Children. *Psychiatria Danubina*, *35*(Suppl 3), 93–94.（The reason for exclusion: Non robot-assisted task-oriented training）
15. Wang, J., Li, Y., Qi, L., Mamtilahun, M., Liu, C., Liu, Z., Shi, R., Wu, S., & Yang, G. Y. (2024). Advanced rehabilitation in ischaemic stroke research. *Stroke and vascular neurology*, *9*(4), 328–343. https://doi.org/10.1136/svn-2022-002285（The reason for exclusion: review）
16. Pignolo L. (2009). Robotics in neuro-rehabilitation. *Journal of rehabilitation medicine*, *41*(12), 955–960. https://doi.org/10.2340/16501977-0434（The reason for exclusion: Non-RCTs）
17. Bui, K. D., & Johnson, M. J. (2018). Designing robot-assisted neurorehabilitation strategies for people with both HIV and stroke. *Journal of neuroengineering and rehabilitation*, *15*(1), 75. https://doi.org/10.1186/s12984-018-0418-3（The reason for exclusion: review）
18. Carrillo, C., Tilley, D., Horn, K., Gonzalez, M., Coffman, C., Hilton, C., & Mani, K. (2023). Effectiveness of Robotics in Stroke Rehabilitation to Accelerate Upper Extremity Function: Systematic Review. *Occupational therapy international*, *2023*, 7991765. https://doi.org/10.1155/2023/7991765（The reason for exclusion: review）
19. Cinnera, A. M., Bonnì, S., D'Acunto, A., Maiella, M., Ferraresi, M., Casula, E. P., Pezzopane, V., Tramontano, M., Iosa, M., Paolucci, S., Morone, G., Vannozzi, G., & Koch, G. (2023). Cortico-cortical stimulation and robot-assisted therapy (CCS and RAT) for upper limb recovery after stroke: study protocol for a randomised controlled trial. *Trials*, *24*(1), 823. https://doi.org/10.1186/s13063-023-07849-1（The reason for exclusion: Control group using additional interventions)
20. Kim, E., Lee, G., Lee, J., & Kim, Y. H. (2024). Simultaneous high-definition transcranial direct current stimulation and robot-assisted gait training in stroke patients. *Scientific reports*, *14*(1), 4483. https://doi.org/10.1038/s41598-024-53482-6（The reason for exclusion: Control group using additional interventions)
21. Stockbridge, M. D., Bunker, L. D., & Hillis, A. E. (2022). Reversing the Ruin: Rehabilitation, Recovery, and Restoration After Stroke. *Current neurology and neuroscience reports*, *22*(11), 745–755. <https://doi.org/10.1007/s11910-022-01231-5>（The reason for exclusion: review）
22. Pinheiro, C., Figueiredo, J., Cerqueira, J., & Santos, C. P. (2022). Robotic Biofeedback for Post-Stroke Gait Rehabilitation: A Scoping Review. *Sensors (Basel, Switzerland)*, *22*(19), 7197. <https://doi.org/10.3390/s22197197>（The reason for exclusion: review）
23. Tseng, K. C., Wang, L., Hsieh, C., & Wong, A. M. (2024). Portable robots for upper-limb rehabilitation after stroke: a systematic review and meta-analysis. *Annals of medicine*, *56*(1), 2337735. <https://doi.org/10.1080/07853890.2024.2337735>（The reason for exclusion: review）
24. Chen, Z., Wang, C., Fan, W., Gu, M., Yasin, G., Xiao, S., Huang, J., & Huang, X. (2020). Robot-Assisted Arm Training versus Therapist-Mediated Training after Stroke: A Systematic Review and Meta-Analysis. *Journal of healthcare engineering*, *2020*, 8810867. <https://doi.org/10.1155/2020/8810867>（The reason for exclusion: review）
25. Zhao, M., Wang, G., Wang, A., Cheng, L. J., & Lau, Y. (2022). Robot-assisted distal training improves upper limb dexterity and function after stroke: a systematic review and meta-regression. *Neurological sciences : official journal of the Italian Neurological Society and of the Italian Society of Clinical Neurophysiology*, *43*(3), 1641–1657. <https://doi.org/10.1007/s10072-022-05913-3>（The reason for exclusion: review）
26. Moggio, L., de Sire, A., Marotta, N., Demeco, A., & Ammendolia, A. (2022). Exoskeleton *versus* end-effector robot-assisted therapy for finger-hand motor recovery in stroke survivors: systematic review and meta-analysis. *Topics in stroke rehabilitation*, *29*(8), 539–550. <https://doi.org/10.1080/10749357.2021.1967657>（The reason for exclusion: review）
27. Tran, D. A., Pajaro-Blazquez, M., Daneault, J. F., Gallegos, J. G., Pons, J., Fregni, F., Bonato, P., & Zafonte, R. (2016). Combining Dopaminergic Facilitation with Robot-Assisted Upper Limb Therapy in Stroke Survivors: A Focused Review. *American journal of physical medicine & rehabilitation*, *95*(6), 459–474. <https://doi.org/10.1097/PHM.0000000000000438>（The reason for exclusion: review）
28. Maranesi, E., Riccardi, G. R., Di Donna, V., Di Rosa, M., Fabbietti, P., Luzi, R., Pranno, L., Lattanzio, F., & Bevilacqua, R. (2020). Effectiveness of Intervention Based on End-effector Gait Trainer in Older Patients With Stroke: A Systematic Review. *Journal of the American Medical Directors Association*, *21*(8), 1036–1044. <https://doi.org/10.1016/j.jamda.2019.10.010>（The reason for exclusion: review）
29. Hachisuka K. (2010). *Brain and nerve = Shinkei kenkyu no shinpo*, *62*(2), 133–140.（The reason for exclusion: review）
30. Choi, J. B., & Cho, K. I. (2024). Effects of virtual reality-based robot therapy combined with task-oriented therapy on upper limb function and cerebral cortex activation in patients with stroke. *Medicine*, *103*(27), e38723. <https://doi.org/10.1097/MD.0000000000038723>（The reason for exclusion: duplicate record）
31. Zhang, L., Jia, G., Ma, J., Wang, S., & Cheng, L. (2022). Short and long-term effects of robot-assisted therapy on upper limb motor function and activity of daily living in patients post-stroke: a meta-analysis of randomized controlled trials. *Journal of neuroengineering and rehabilitation*, *19*(1), 76. <https://doi.org/10.1186/s12984-022-01058-8>（The reason for exclusion: review）
32. Khan, M. A., Saibene, M., Das, R., Brunner, I., & Puthusserypady, S. (2021). Emergence of flexible technology in developing advanced systems for post-stroke rehabilitation: a comprehensive review. *Journal of neural engineering*, *18*(6), 10.1088/1741-2552/ac36aa. <https://doi.org/10.1088/1741-2552/ac36aa>（The reason for exclusion: review）
33. Tang, C., Zhou, T., Zhang, Y., Yuan, R., Zhao, X., Yin, R., Song, P., Liu, B., Song, R., Chen, W., & Wang, H. (2023). Bilateral upper limb robot-assisted rehabilitation improves upper limb motor function in stroke patients: a study based on quantitative EEG. *European journal of medical research*, *28*(1), 603. <https://doi.org/10.1186/s40001-023-01565-x>（The reason for exclusion: duplicate record）
34. Yue, Z., Zhang, X., & Wang, J. (2017). Hand Rehabilitation Robotics on Poststroke Motor Recovery. *Behavioural neurology*, *2017*, 3908135. <https://doi.org/10.1155/2017/3908135>（The reason for exclusion: review）
35. Hu, Y., Meng, J., Li, G., Zhao, D., Feng, G., Zuo, G., Liu, Y., Zhang, J., & Shi, C. (2023). Fuzzy Adaptive Passive Control Strategy Design for Upper-Limb End-Effector Rehabilitation Robot. *Sensors (Basel, Switzerland)*, *23*(8), 4042. https://doi.org/10.3390/s23084042（The reason for exclusion: Non-RCTs）
36. Devittori, G., Dinacci, D., Romiti, D., Califfi, A., Petrillo, C., Rossi, P., Ranzani, R., Gassert, R., & Lambercy, O. (2024). Unsupervised robot-assisted rehabilitation after stroke: feasibility, effect on therapy dose, and user experience. *Journal of neuroengineering and rehabilitation*, *21*(1), 52. https://doi.org/10.1186/s12984-024-01347-4（The reason for exclusion: Non robot-assisted task-oriented training）
37. O'Malley, M. K., Ro, T., & Levin, H. S. (2006). Assessing and inducing neuroplasticity with transcranial magnetic stimulation and robotics for motor function. *Archives of physical medicine and rehabilitation*, *87*(12 Suppl 2), S59–S66. https://doi.org/10.1016/j.apmr.2006.08.332（The reason for exclusion: review）
38. Balasubramanian, S., Klein, J., & Burdet, E. (2010). Robot-assisted rehabilitation of hand function. *Current opinion in neurology*, *23*(6), 661–670. <https://doi.org/10.1097/WCO.0b013e32833e99a4>（The reason for exclusion: review）
39. Sun, Z., Mu, A., Wang, C., Liu, Q., Hao, F., Wei, J., & Li, W. (2023). Research on an ankle rehabilitation robot for hemiplegic patients after stroke. *Proceedings of the Institution of Mechanical Engineers. Part H, Journal of engineering in medicine*, *237*(10), 1177–1189. https://doi.org/10.1177/09544119231197082（The reason for exclusion: Non-RCTs）
40. Saragih, I. D., Everard, G., Tzeng, H. M., Saragih, I. S., & Lee, B. O. (2023). Efficacy of Robots-Assisted Therapy in Patients With Stroke: A Meta-analysis Update. *The Journal of cardiovascular nursing*, *38*(6), E192–E217. <https://doi.org/10.1097/JCN.0000000000000945>（The reason for exclusion: review）
41. Tran, V. D., Dario, P., & Mazzoleni, S. (2018). Kinematic measures for upper limb robot-assisted therapy following stroke and correlations with clinical outcome measures: A review. *Medical engineering & physics*, *53*, 13–31. <https://doi.org/10.1016/j.medengphy.2017.12.005>（The reason for exclusion: review）
42. Shin, S. Y., Hohl, K., Giffhorn, M., Awad, L. N., Walsh, C. J., & Jayaraman, A. (2022). Soft robotic exosuit augmented high intensity gait training on stroke survivors: a pilot study. *Journal of neuroengineering and rehabilitation*, *19*(1), 51. https://doi.org/10.1186/s12984-022-01034-2（The reason for exclusion: Irrelevant study outcome）
43. Kuczynski, A. M., Carlson, H. L., Lebel, C., Hodge, J. A., Dukelow, S. P., Semrau, J. A., & Kirton, A. (2017). Sensory tractography and robot-quantified proprioception in hemiparetic children with perinatal stroke. *Human brain mapping*, *38*(5), 2424–2440. https://doi.org/10.1002/hbm.23530（The reason for exclusion: Non robot-assisted task-oriented training）
44. Li, Y., Lian, Y., Chen, X., Zhang, H., Xu, G., Duan, H., Xie, X., & Li, Z. (2024). Effect of task-oriented training assisted by force feedback hand rehabilitation robot on finger grasping function in stroke patients with hemiplegia: a randomised controlled trial. *Journal of neuroengineering and rehabilitation*, *21*(1), 77. https://doi.org/10.1186/s12984-024-01372-3（The reason for exclusion: duplicate record）
45. Grosmaire, A. G., Pila, O., Breuckmann, P., & Duret, C. (2022). Robot-assisted therapy for upper limb paresis after stroke: Use of robotic algorithms in advanced practice. *NeuroRehabilitation*, *51*(4), 577–593. <https://doi.org/10.3233/NRE-220025>（The reason for exclusion: review）
46. Zanatta, F., Farhane-Medina, N. Z., Adorni, R., Steca, P., Giardini, A., D'Addario, M., & Pierobon, A. (2023). Combining robot-assisted therapy with virtual reality or using it alone? A systematic review on health-related quality of life in neurological patients. *Health and quality of life outcomes*, *21*(1), 18. <https://doi.org/10.1186/s12955-023-02097-y>（The reason for exclusion: review）
47. Cho, J. E., Yoo, J. S., Kim, K. E., Cho, S. T., Jang, W. S., Cho, K. H., & Lee, W. H. (2018). Systematic Review of Appropriate Robotic Intervention for Gait Function in Subacute Stroke Patients. *BioMed research international*, *2018*, 4085298. <https://doi.org/10.1155/2018/4085298>（The reason for exclusion: review）
48. Hogan, N., & Krebs, H. I. (2011). Physically interactive robotic technology for neuromotor rehabilitation. *Progress in brain research*, *192*, 59–68. <https://doi.org/10.1016/B978-0-444-53355-5.00004-X>（The reason for exclusion: review）
49. Bertani, R., Melegari, C., De Cola, M. C., Bramanti, A., Bramanti, P., & Calabrò, R. S. (2017). Effects of robot-assisted upper limb rehabilitation in stroke patients: a systematic review with meta-analysis. *Neurological sciences : official journal of the Italian Neurological Society and of the Italian Society of Clinical Neurophysiology*, *38*(9), 1561–1569. <https://doi.org/10.1007/s10072-017-2995-5>（The reason for exclusion: review）
50. Palmcrantz, S., Plantin, J., & Borg, J. (2020). Factors affecting the usability of an assistive soft robotic glove after stroke or multiple sclerosis. *Journal of rehabilitation medicine*, *52*(3), jrm00027. <https://doi.org/10.2340/16501977-2650>（The reason for exclusion: Non robot-assisted task-oriented training）
51. Lefeber, N., De Keersmaecker, E., Henderix, S., Michielsen, M., Tamburella, F., Tagliamonte, N. L., Molinari, M., de Geus, B., Kerckhofs, E., & Swinnen, E. (2021). Physiological responses and perceived exertion during robot-assisted treadmill walking in non-ambulatory stroke survivors. *Disability and rehabilitation*, *43*(11), 1576–1584. <https://doi.org/10.1080/09638288.2019.1671502>（The reason for exclusion: Non robot-assisted task-oriented training）
52. Cazenave, L., Einenkel, M., Yurkewich, A., Endo, S., Hirche, S., & Burdet, E. (2023). Hybrid Robotic and Electrical Stimulation Assistance Can Enhance Performance and Reduce Mental Demand. *IEEE transactions on neural systems and rehabilitation engineering : a publication of the IEEE Engineering in Medicine and Biology Society*, *31*, 4063–4072. <https://doi.org/10.1109/TNSRE.2023.3323370>（The reason for exclusion: Non robot-assisted task-oriented training）
53. Feingold-Polak, R., Barzel, O., & Levy-Tzedek, S. (2024). Socially Assistive Robot for Stroke Rehabilitation: A Long-Term in-the-Wild Pilot Randomized Controlled Trial. *IEEE transactions on neural systems and rehabilitation engineering : a publication of the IEEE Engineering in Medicine and Biology Society*, *32*, 1616–1626. <https://doi.org/10.1109/TNSRE.2024.3387320>（The reason for exclusion: Non robot-assisted task-oriented training）
54. Guler, M. A., Erhan, B., & Yilmaz Yalcinkaya, E. (2021). Caregiver burden in stroke inpatients: a randomized study comparing robot-assisted gait training and conventional therapy. *Acta neurologica Belgica*, *121*(3), 729–736. <https://doi.org/10.1007/s13760-020-01465-5>（The reason for exclusion: Non robot-assisted task-oriented training）
55. Grosmaire, A. G., Pila, O., Breuckmann, P., & Duret, C. (2022). Robot-assisted therapy for upper limb paresis after stroke: Use of robotic algorithms in advanced practice. *NeuroRehabilitation*, *51*(4), 577–593. <https://doi.org/10.3233/NRE-220025>（The reason for exclusion: review）
56. Palmcrantz, S., Plantin, J., & Borg, J. (2020). Factors affecting the usability of an assistive soft robotic glove after stroke or multiple sclerosis. *Journal of rehabilitation medicine*, *52*(3), jrm00027. <https://doi.org/10.2340/16501977-2650>（The reason for exclusion: duplicate record）
57. Lefeber, N., De Keersmaecker, E., Henderix, S., Michielsen, M., Tamburella, F., Tagliamonte, N. L., Molinari, M., de Geus, B., Kerckhofs, E., & Swinnen, E. (2021). Physiological responses and perceived exertion during robot-assisted treadmill walking in non-ambulatory stroke survivors. *Disability and rehabilitation*, *43*(11), 1576–1584. <https://doi.org/10.1080/09638288.2019.1671502>（The reason for exclusion: duplicate record）
58. Cazenave, L., Einenkel, M., Yurkewich, A., Endo, S., Hirche, S., & Burdet, E. (2023). Hybrid Robotic and Electrical Stimulation Assistance Can Enhance Performance and Reduce Mental Demand. *IEEE transactions on neural systems and rehabilitation engineering : a publication of the IEEE Engineering in Medicine and Biology Society*, *31*, 4063–4072. <https://doi.org/10.1109/TNSRE.2023.3323370>（The reason for exclusion: duplicate record）
59. Feingold-Polak, R., Barzel, O., & Levy-Tzedek, S. (2024). Socially Assistive Robot for Stroke Rehabilitation: A Long-Term in-the-Wild Pilot Randomized Controlled Trial. *IEEE transactions on neural systems and rehabilitation engineering : a publication of the IEEE Engineering in Medicine and Biology Society*, *32*, 1616–1626. <https://doi.org/10.1109/TNSRE.2024.3387320>（The reason for exclusion: duplicate record）
60. Guler, M. A., Erhan, B., & Yilmaz Yalcinkaya, E. (2021). Caregiver burden in stroke inpatients: a randomized study comparing robot-assisted gait training and conventional therapy. *Acta neurologica Belgica*, *121*(3), 729–736. <https://doi.org/10.1007/s13760-020-01465-5>（The reason for exclusion: duplicate record）
61. Zanatta, F., Farhane-Medina, N. Z., Adorni, R., Steca, P., Giardini, A., D'Addario, M., & Pierobon, A. (2023). Combining robot-assisted therapy with virtual reality or using it alone? A systematic review on health-related quality of life in neurological patients. *Health and quality of life outcomes*, *21*(1), 18. <https://doi.org/10.1186/s12955-023-02097-y>（The reason for exclusion: review）
62. Cho, J. E., Yoo, J. S., Kim, K. E., Cho, S. T., Jang, W. S., Cho, K. H., & Lee, W. H. (2018). Systematic Review of Appropriate Robotic Intervention for Gait Function in Subacute Stroke Patients. *BioMed research international*, *2018*, 4085298. <https://doi.org/10.1155/2018/4085298>（The reason for exclusion: review）
63. Hogan, N., & Krebs, H. I. (2011). Physically interactive robotic technology for neuromotor rehabilitation. *Progress in brain research*, *192*, 59–68. <https://doi.org/10.1016/B978-0-444-53355-5.00004-X>（The reason for exclusion: review）
64. Bertani, R., Melegari, C., De Cola, M. C., Bramanti, A., Bramanti, P., & Calabrò, R. S. (2017). Effects of robot-assisted upper limb rehabilitation in stroke patients: a systematic review with meta-analysis. *Neurological sciences : official journal of the Italian Neurological Society and of the Italian Society of Clinical Neurophysiology*, *38*(9), 1561–1569. <https://doi.org/10.1007/s10072-017-2995-5>（The reason for exclusion: review）
65. Krebs, H. I., Saitoh, E., & Hogan, N. (2015). Robotic Therapy and the Paradox of the Diminishing Number of Degrees of Freedom. *Physical medicine and rehabilitation clinics of North America*, *26*(4), 691–702. <https://doi.org/10.1016/j.pmr.2015.06.003>（The reason for exclusion: Non-RCTs）
66. Berczeli, M., Chinnadurai, P., Legeza, P. T., Britz, G. W., & Lumsden, A. B. (2022). Transcarotid access for remote robotic endovascular neurointerventions: a cadaveric proof-of-concept study. *Neurosurgical focus*, *52*(1), E18. <https://doi.org/10.3171/2021.10.FOCUS21511>（The reason for exclusion: Non-RCTs）
67. Wade, E., & Winstein, C. J. (2011). Virtual reality and robotics for stroke rehabilitation: where do we go from here?. *Topics in stroke rehabilitation*, *18*(6), 685–700. <https://doi.org/10.1310/tsr1806-685>（The reason for exclusion: Non-RCTs）
68. Yokota, C., Tanaka, K., Omae, K., Kamada, M., Nishikawa, H., Koga, M., Ihara, M., Fujimoto, Y., Sankai, Y., Nakajima, T., & Minami, M. (2023). Effect of cyborg-type robot Hybrid Assistive Limb on patients with severe walking disability in acute stroke: A randomized controlled study. *Journal of stroke and cerebrovascular diseases : the official journal of National Stroke Association*, *32*(4), 107020. <https://doi.org/10.1016/j.jstrokecerebrovasdis.2023.107020>（The reason for exclusion: duplicate record）
69. Elangovan, N., Yeh, I. L., Holst-Wolf, J., & Konczak, J. (2019). A robot-assisted sensorimotor training program can improve proprioception and motor function in stroke survivors. *IEEE ... International Conference on Rehabilitation Robotics : [proceedings]*, *2019*, 660–664. https://doi.org/10.1109/ICORR.2019.8779409（The reason for exclusion: meeting abstract）
70. Yokota, C., Tanaka, K., Omae, K., Kamada, M., Nishikawa, H., Koga, M., Ihara, M., Fujimoto, Y., Sankai, Y., Nakajima, T., & Minami, M. (2023). Effect of cyborg-type robot Hybrid Assistive Limb on patients with severe walking disability in acute stroke: A randomized controlled study. *Journal of stroke and cerebrovascular diseases : the official journal of National Stroke Association*, *32*(4), 107020. https://doi.org/10.1016/j.jstrokecerebrovasdis.2023.107020（The reason for exclusion: duplicate record）
71. Alashram A. R. (2024). Combined robot-assisted therapy virtual reality for upper limb rehabilitation in stroke survivors: a systematic review of randomized controlled trials. *Neurological sciences : official journal of the Italian Neurological Society and of the Italian Society of Clinical Neurophysiology*, *45*(11), 5141–5155. <https://doi.org/10.1007/s10072-024-07628-z>（The reason for exclusion: review）
72. Nam, C., Rong, W., Li, W., Cheung, C., Ngai, W., Cheung, T., Pang, M., Li, L., Hu, J., Wai, H., & Hu, X. (2022). An Exoneuromusculoskeleton for Self-Help Upper Limb Rehabilitation After Stroke. *Soft robotics*, *9*(1), 14–35. https://doi.org/10.1089/soro.2020.0090（The reason for exclusion: Non-RCTs）
73. Lin, Y. N., Huang, S. W., Kuan, Y. C., Chen, H. C., Jian, W. S., & Lin, L. F. (2022). Hybrid robot-assisted gait training for motor function in subacute stroke: a single-blind randomized controlled trial. *Journal of neuroengineering and rehabilitation*, *19*(1), 99. <https://doi.org/10.1186/s12984-022-01076-6>（The reason for exclusion: duplicate record）
74. Hussain, S., Jamwal, P. K., Vliet, P. V., & Brown, N. A. T. (2021). Robot Assisted Ankle Neuro-Rehabilitation: State of the art and Future Challenges. *Expert review of neurotherapeutics*, *21*(1), 111–121. <https://doi.org/10.1080/14737175.2021.1847646>（The reason for exclusion: review）
75. Yokota, C., Yamamoto, Y., Kamada, M., Nakai, M., Nishimura, K., Ando, D., Sato, T., Koga, M., Ihara, M., Toyoda, K., Fujimoto, Y., Odani, H., Minematsu, K., & Nakajima, T. (2019). Acute stroke rehabilitation for gait training with cyborg type robot Hybrid Assistive Limb: A pilot study. *Journal of the neurological sciences*, *404*, 11–15. https://doi.org/10.1016/j.jns.2019.07.012（The reason for exclusion: Non robot-assisted task-oriented training）
76. Sale, P., Franceschini, M., Waldner, A., & Hesse, S. (2012). Use of the robot assisted gait therapy in rehabilitation of patients with stroke and spinal cord injury. *European journal of physical and rehabilitation medicine*, *48*(1), 111–121.（The reason for exclusion: review）
77. Chen, X., Yin, L., Hou, Y., Wang, J., Li, Y., Yan, J., Tao, J., & Ma, S. (2024). Effect of robot-assisted gait training on improving cardiopulmonary function in stroke patients: a meta-analysis. *Journal of neuroengineering and rehabilitation*, *21*(1), 92. <https://doi.org/10.1186/s12984-024-01388-9>（The reason for exclusion: review）
78. Yang, R., Shen, Z., Lyu, Y., Zhuang, Y., Li, L., & Song, R. (2023). Voluntary Assist-as-Needed Controller for an Ankle Power-Assist Rehabilitation Robot. *IEEE transactions on bio-medical engineering*, *70*(6), 1795–1803. <https://doi.org/10.1109/TBME.2022.3228070>（The reason for exclusion: duplicate record）
79. Zbytniewska-Megret, M., Salzmann, C., Ranzani, R., Kanzler, C. M., Gassert, R., Liepert, J., & Lambercy, O. (2022). Design and Preliminary Evaluation of a Robot-assisted Assessment-driven Finger Proprioception Therapy. *IEEE ... International Conference on Rehabilitation Robotics : [proceedings]*, *2022*, 1–6. <https://doi.org/10.1109/ICORR55369.2022.9896602>（The reason for exclusion: Non-RCTs）
80. Choi W. (2022). Effects of Robot-Assisted Gait Training with Body Weight Support on Gait and Balance in Stroke Patients. *International journal of environmental research and public health*, *19*(10), 5814. <https://doi.org/10.3390/ijerph19105814>（The reason for exclusion: duplicate record）
81. Hung, J. W., Yen, C. L., Chang, K. C., Chiang, W. C., Chuang, I. C., Pong, Y. P., Wu, W. C., & Wu, C. Y. (2022). A Pilot Randomized Controlled Trial of Botulinum Toxin Treatment Combined with Robot-Assisted Therapy, Mirror Therapy, or Active Control Treatment in Patients with Spasticity Following Stroke. *Toxins*, *14*(6), 415. <https://doi.org/10.3390/toxins14060415>（The reason for exclusion: duplicate record）
82. Bui, K. D., Lyn, B., Roland, M., Wamsley, C. A., Mendonca, R., & Johnson, M. J. (2022). The Impact of Cognitive Impairment on Robot-Based Upper-Limb Motor Assessment in Chronic Stroke. *Neurorehabilitation and neural repair*, *36*(9), 587–595. <https://doi.org/10.1177/15459683221110892>（The reason for exclusion: duplicate record）
83. Costa, M., Tataryn, Z., Alobaid, A., Pierre, C., Basamh, M., Somji, M., Loh, Y., Patel, A., & Monteith, S. (2023). Robotically-assisted neuro-endovascular procedures: Single-Center Experience and a Review of the Literature. *Interventional neuroradiology : journal of peritherapeutic neuroradiology, surgical procedures and related neurosciences*, *29*(2), 201–210. <https://doi.org/10.1177/15910199221082475>（The reason for exclusion: review）
84. Fonte, C., Varalta, V., Rocco, A., Munari, D., Filippetti, M., Evangelista, E., Modenese, A., Smania, N., & Picelli, A. (2021). Combined transcranial Direct Current Stimulation and robot-assisted arm training in patients with stroke: a systematic review. *Restorative neurology and neuroscience*, *39*(6), 435–446. <https://doi.org/10.3233/RNN-211218>（The reason for exclusion: duplicate record）
85. Leem, M. J., Kim, G. S., Kim, K. H., Yi, T. I., & Moon, H. I. (2019). Predictors of functional and motor outcomes following upper limb robot-assisted therapy after stroke. *International journal of rehabilitation research. Internationale Zeitschrift fur Rehabilitationsforschung. Revue internationale de recherches de readaptation*, *42*(3), 223–228. <https://doi.org/10.1097/MRR.0000000000000349>（The reason for exclusion: duplicate record）
86. Pérez, P. J., Garcia-Zapirain, B., & Mendez-Zorrilla, A. (2015). Caregiver and social assistant robot for rehabilitation and coaching for the elderly. *Technology and health care : official journal of the European Society for Engineering and Medicine*, *23*(3), 351–357. <https://doi.org/10.3233/THC-150896>（The reason for exclusion: duplicate record）
87. Nizamis, K., Athanasiou, A., Almpani, S., Dimitrousis, C., & Astaras, A. (2021). Converging Robotic Technologies in Targeted Neural Rehabilitation: A Review of Emerging Solutions and Challenges. *Sensors (Basel, Switzerland)*, *21*(6), 2084. <https://doi.org/10.3390/s21062084>（The reason for exclusion: review）
88. Khalid, S., Alnajjar, F., Gochoo, M., Renawi, A., & Shimoda, S. (2023). Robotic assistive and rehabilitation devices leading to motor recovery in upper limb: a systematic review. *Disability and rehabilitation. Assistive technology*, *18*(5), 658–672. <https://doi.org/10.1080/17483107.2021.1906960>（The reason for exclusion: review）
89. Chen, S. C., Kang, J. H., Peng, C. W., Hsu, C. C., Lin, Y. N., & Lai, C. H. (2022). Adjustable Parameters and the Effectiveness of Adjunct Robot-Assisted Gait Training in Individuals with Chronic Stroke. *International journal of environmental research and public health*, *19*(13), 8186. <https://doi.org/10.3390/ijerph19138186>（The reason for exclusion: duplicate record）
90. Yang, X., Fengyi, W., Yi, C., Lin, Q., Yang, L., Xize, L., Shaxin, L., & Yonghong, Y. (2024). Effects of robot-assisted upper limb training combined with functional electrical stimulation in stroke patients: study protocol for a randomized controlled trial. *Trials*, *25*(1), 355. <https://doi.org/10.1186/s13063-024-08199-2>（The reason for exclusion: duplicate record）
91. Hu, M. M., Wang, S., Wu, C. Q., Li, K. P., Geng, Z. H., Xu, G. H., & Dong, L. (2024). Efficacy of robot-assisted gait training on lower extremity function in subacute stroke patients: a systematic review and meta-analysis. *Journal of neuroengineering and rehabilitation*, *21*(1), 165. <https://doi.org/10.1186/s12984-024-01463-1>（The reason for exclusion: review）
92. Hesse, S., Tomelleri, C., Bardeleben, A., Werner, C., & Waldner, A. (2012). Robot-assisted practice of gait and stair climbing in nonambulatory stroke patients. *Journal of rehabilitation research and development*, *49*(4), 613–622. <https://doi.org/10.1682/jrrd.2011.08.0142>（The reason for exclusion: duplicate record）
93. Mazzoleni, S., Focacci, A., Franceschini, M., Waldner, A., Spagnuolo, C., Battini, E., & Bonaiuti, D. (2017). Robot-assisted end-effector-based gait training in chronic stroke patients: A multicentric uncontrolled observational retrospective clinical study. *NeuroRehabilitation*, *40*(4), 483–492. <https://doi.org/10.3233/NRE-161435>（The reason for exclusion: duplicate record）
94. Klinkwan, P., Kongmaroeng, C., Muengtaweepongsa, S., & Limtrakarn, W. (2023). Prototype development of bilateral arm mirror-like-robotic rehabilitation device for acute stroke patients. *Biomedical physics & engineering express*, *9*(4), 10.1088/2057-1976/acd11d. <https://doi.org/10.1088/2057-1976/acd11d>（The reason for exclusion: Non robot-assisted task-oriented training）
95. Li, N., Yang, T., Yu, P., Chang, J., Zhao, L., Zhao, X., Elhajj, I. H., Xi, N., & Liu, L. (2018). Bio-inspired upper limb soft exoskeleton to reduce stroke-induced complications. *Bioinspiration & biomimetics*, *13*(6), 066001. <https://doi.org/10.1088/1748-3190/aad8d4>（The reason for exclusion: Non robot-assisted task-oriented training）
96. Yakşi, E., Bahadır, E. S., Yaşar, M. F., Alışık, T., Kurul, R., & Demirel, A. (2023). The effect of robot-assisted gait training frequency on walking, functional recovery, and quality of life in patients with stroke. *Acta neurologica Belgica*, *123*(2), 583–590. <https://doi.org/10.1007/s13760-023-02194-1>（The reason for exclusion: duplicate record）
97. Lim, D. Y., Lai, H. S., & Yeow, R. C. (2023). A bidirectional fabric-based soft robotic glove for hand function assistance in patients with chronic stroke. *Journal of neuroengineering and rehabilitation*, *20*(1), 120. https://doi.org/10.1186/s12984-023-01250-4（The reason for exclusion: duplicate record）
98. Neo, J. R. E., Visperas, C. A., Tan, M. P. H., & Tay, S. S. (2023). Novel use of robot-assisted gait rehabilitation in a patient with stroke and blindness. *BMJ case reports*, *16*(7), e255457. <https://doi.org/10.1136/bcr-2023-255457>（The reason for exclusion: Non-RCTs）
99. Sayın, A. M., Duruturk, N., Balaban, B., & Korkusuz, S. (2023). The effect of robot-assisted walking in different modalities on cardiorespiratory responses and energy consumption in patients with subacute stroke. *Neurological research*, *45*(7), 688–694. <https://doi.org/10.1080/01616412.2023.2188520>（The reason for exclusion: Non-RCTs）
100. Uehara, S., Yuasa, A., Ushizawa, K., Kitamura, S., Yamazaki, K., Otaka, E., & Otaka, Y. (2023). Direction-dependent differences in the quality and quantity of horizontal reaching in people after stroke. *Journal of neurophysiology*, *130*(4), 861–870. <https://doi.org/10.1152/jn.00455.2022>（The reason for exclusion: duplicate record）
101. Tomida, K., Sonoda, S., Hirano, S., Suzuki, A., Tanino, G., Kawakami, K., Saitoh, E., & Kagaya, H. (2019). Randomized Controlled Trial of Gait Training Using Gait Exercise Assist Robot (GEAR) in Stroke Patients with Hemiplegia. *Journal of stroke and cerebrovascular diseases : the official journal of National Stroke Association*, *28*(9), 2421–2428. <https://doi.org/10.1016/j.jstrokecerebrovasdis.2019.06.030>（The reason for exclusion: duplicate record）
102. Volpe, B. T., Ferraro, M., Lynch, D., Christos, P., Krol, J., Trudell, C., Krebs, H. I., & Hogan, N. (2005). Robotics and other devices in the treatment of patients recovering from stroke. *Current neurology and neuroscience reports*, *5*(6), 465–470. <https://doi.org/10.1007/s11910-005-0035-y>（The reason for exclusion: duplicate record）
103. Shin, J., An, H., Yang, S., Park, C., Lee, Y., & You, S. J. H. (2022). Comparative effects of passive and active mode robot-assisted gait training on brain and muscular activities in sub-acute and chronic stroke. *NeuroRehabilitation*, *51*(1), 51–63. <https://doi.org/10.3233/NRE-210304>（The reason for exclusion: duplicate record）
104. Rikhof, C. J. H., Feenstra, Y., Fleuren, J. F. M., Buurke, J. H., Prinsen, E. C., Rietman, J. S., & Prange-Lasonder, G. B. (2024). Robot-assisted support combined with electrical stimulation for the lower extremity in stroke patients: a systematic review. *Journal of neural engineering*, *21*(2), 10.1088/1741-2552/ad377c. <https://doi.org/10.1088/1741-2552/ad377c>（The reason for exclusion: review）
105. Mehrholz J. (2019). Is Electromechanical and Robot-Assisted Arm Training Effective for Improving Arm Function in People Who Have Had a Stroke?: A Cochrane Review Summary With Commentary. *American journal of physical medicine & rehabilitation*, *98*(4), 339–340. <https://doi.org/10.1097/PHM.0000000000001133>（The reason for exclusion: review）
106. Semrau, J. A., Herter, T. M., Scott, S. H., & Dukelow, S. P. (2015). Examining Differences in Patterns of Sensory and Motor Recovery After Stroke With Robotics. *Stroke*, *46*(12), 3459–3469. <https://doi.org/10.1161/STROKEAHA.115.010750>（The reason for exclusion: duplicate record）
107. Chen, Y. W., Chiang, W. C., Chang, C. L., Lo, S. M., & Wu, C. Y. (2022). Comparative effects of EMG-driven robot-assisted therapy versus task-oriented training on motor and daily function in patients with stroke: a randomized cross-over trial. *Journal of neuroengineering and rehabilitation*, *19*(1), 6. <https://doi.org/10.1186/s12984-021-00961-w>（The reason for exclusion: duplicate record）
108. Yurkewich, A., Hebert, D., Wang, R. H., & Mihailidis, A. (2019). Hand Extension Robot Orthosis (HERO) Glove: Development and Testing With Stroke Survivors With Severe Hand Impairment. *IEEE transactions on neural systems and rehabilitation engineering : a publication of the IEEE Engineering in Medicine and Biology Society*, *27*(5), 916–926. <https://doi.org/10.1109/TNSRE.2019.2910011>（The reason for exclusion: duplicate record）
109. Joo, M. C., Jung, K. M., Kim, J. H., Jung, Y. J., Chang, W. N., & Shin, H. J. (2022). Robot-Assisted Therapy Combined with Trunk Restraint in Acute Stroke Patients: A Randomized Controlled Study. *Journal of stroke and cerebrovascular diseases : the official journal of National Stroke Association*, *31*(5), 106330. <https://doi.org/10.1016/j.jstrokecerebrovasdis.2022.106330>（The reason for exclusion: duplicate record）
110. Yeung, L. F., Lau, C. C. Y., Lai, C. W. K., Soo, Y. O. Y., Chan, M. L., & Tong, R. K. Y. (2021). Effects of wearable ankle robotics for stair and over-ground training on sub-acute stroke: a randomized controlled trial. *Journal of neuroengineering and rehabilitation*, *18*(1), 19. <https://doi.org/10.1186/s12984-021-00814-6>（The reason for exclusion: duplicate record）
111. Thimabut, N., Yotnuengnit, P., Charoenlimprasert, J., Sillapachai, T., Hirano, S., Saitoh, E., & Piravej, K. (2022). Effects of the Robot-Assisted Gait Training Device Plus Physiotherapy in Improving Ambulatory Functions in Patients With Subacute Stroke With Hemiplegia: An Assessor-Blinded, Randomized Controlled Trial. *Archives of physical medicine and rehabilitation*, *103*(5), 843–850. <https://doi.org/10.1016/j.apmr.2022.01.146>（The reason for exclusion: duplicate record）
112. Bernal-Jiménez, J. J., Dileone, M., Mordillo-Mateos, L., Martín-Conty, J. L., Durantez-Fernández, C., Viñuela, A., Martín-Rodríguez, F., Lerin-Calvo, A., Alcántara-Porcuna, V., & Polonio-López, B. (2024). Combining Transcranial Direct Current Stimulation With Hand Robotic Rehabilitation in Chronic Stroke Patients: A Double-Blind Randomized Clinical Trial. *American journal of physical medicine & rehabilitation*, *103*(10), 875–882. <https://doi.org/10.1097/PHM.0000000000002446>（The reason for exclusion: duplicate record）
113. Proulx, C. E., Higgins, J., & Gagnon, D. H. (2023). Occupational therapists' evaluation of the perceived usability and utility of wearable soft robotic exoskeleton gloves for hand function rehabilitation following a stroke. *Disability and rehabilitation. Assistive technology*, *18*(6), 953–962. <https://doi.org/10.1080/17483107.2021.1938710>（The reason for exclusion: duplicate record）
114. Rikhof, C. J. H., Leerskov, K. S., Prange-Lasonder, G. B., Prinsen, E. C., Spaich, E. G., Dosen, S., Struijk, L. N. S. A., Buurke, J. H., & Rietman, J. S. (2024). Combining robotics and functional electrical stimulation for assist-as-needed support of leg movements in stroke patients: A feasibility study. *Medical engineering & physics*, *130*, 104216. <https://doi.org/10.1016/j.medengphy.2024.104216>（The reason for exclusion: duplicate record）
115. Skovgaard Jensen, J., Sørensen, A. S., Kruuse, C., Nielsen, H. H., Skov, C. D., Jensen, H. B., Buckwalter, M. S., Bojsen-Møller, J., Lambertsen, K. L., & Holsgaard-Larsen, A. (2024). The effect of robot-assisted versus standard training on motor function following subacute rehabilitation after ischemic stroke - protocol for a randomised controlled trial nested in a prospective cohort (RoboRehab). *BMC neurology*, *24*(1), 233. <https://doi.org/10.1186/s12883-024-03734-9>（The reason for exclusion: duplicate record）
116. Lin, Y., Qu, Q., Lin, Y., He, J., Zhang, Q., Wang, C., Jiang, Z., Guo, F., & Jia, J. (2021). Customizing Robot-Assisted Passive Neurorehabilitation Exercise Based on Teaching Training Mechanism. *BioMed research international*, *2021*, 9972560. <https://doi.org/10.1155/2021/9972560>（The reason for exclusion: duplicate record）
117. Chan, H. L., Hung, J. W., Chang, K. C., & Wu, C. Y. (2021). Myoelectric analysis of upper-extremity muscles during robot-assisted bilateral wrist flexion-extension in subjects with poststroke hemiplegia. *Clinical biomechanics (Bristol, Avon)*, *87*, 105412. <https://doi.org/10.1016/j.clinbiomech.2021.105412>（The reason for exclusion: duplicate record）
118. Ru, H., Gao, W., Ou, W., Yang, X., Li, A., Fu, Z., Huo, J., Yang, B., Zhang, Y., Xiao, X., Yang, Z., & Huang, J. (2023). A Flexible Wearable Supernumerary Robotic Limb for Chronic Stroke Patients. *Journal of visualized experiments : JoVE*, (200), 10.3791/65917. <https://doi.org/10.3791/65917>（The reason for exclusion: duplicate record）
119. Hirano, S., Saitoh, E., Imoto, D., Ii, T., Tsunoda, T., & Otaka, Y. (2024). Effects of robot-assisted gait training using the Welwalk on gait independence for individuals with hemiparetic stroke: an assessor-blinded, multicenter randomized controlled trial. *Journal of neuroengineering and rehabilitation*, *21*(1), 76. <https://doi.org/10.1186/s12984-024-01370-5>（The reason for exclusion: duplicate record）
120. Wang, L., Zheng, Y., Dang, Y., Teng, M., Zhang, X., Cheng, Y., Zhang, X., Yu, Q., Yin, A., & Lu, X. (2021). Effects of robot-assisted training on balance function in patients with stroke: A systematic review and meta-analysis. *Journal of rehabilitation medicine*, *53*(4), jrm00174. <https://doi.org/10.2340/16501977-2815>（The reason for exclusion: review）
121. Zheng, Q. X., Ge, L., Wang, C. C., Ma, Q. S., Liao, Y. T., Huang, P. P., Wang, G. D., Xie, Q. L., & Rask, M. (2019). Robot-assisted therapy for balance function rehabilitation after stroke: A systematic review and meta-analysis. *International journal of nursing studies*, *95*, 7–18. <https://doi.org/10.1016/j.ijnurstu.2019.03.015>（The reason for exclusion: review）
122. Leconte, P., & Ronsse, R. (2016). Performance-based robotic assistance during rhythmic arm exercises. *Journal of neuroengineering and rehabilitation*, *13*(1), 82. <https://doi.org/10.1186/s12984-016-0189-7>（The reason for exclusion: duplicate record）
123. Behidj, A., Achiche, S., & Mohebbi, A. (2023). Upper-Limb Rehabilitation of Patients with Neuromotor Deficits Using Impedance-Based Control of a 6-DOF Robot. *Annual International Conference of the IEEE Engineering in Medicine and Biology Society. IEEE Engineering in Medicine and Biology Society. Annual International Conference*, *2023*, 1–4. <https://doi.org/10.1109/EMBC40787.2023.10340328>（The reason for exclusion: duplicate record）
124. Cho, K. H., & Song, W. K. (2021). Effects of two different robot-assisted arm training on upper limb motor function and kinematics in chronic stroke survivors: A randomized controlled trial. *Topics in stroke rehabilitation*, *28*(4), 241–250. <https://doi.org/10.1080/10749357.2020.1804699>（The reason for exclusion: duplicate record）
125. Qu, Q., Lin, Y., He, Z., Fu, J., Zou, F., Jiang, Z., Guo, F., & Jia, J. (2021). The Effect of Applying Robot-Assisted Task-Oriented Training Using Human-Robot Collaborative Interaction Force Control Technology on Upper Limb Function in Stroke Patients: Preliminary Findings. *BioMed research international*, *2021*, 9916492. <https://doi.org/10.1155/2021/9916492>（The reason for exclusion: duplicate record）
126. Wright, Z. A., Patton, J. L., & Huang, F. C. (2018). Energetics during robot-assisted training predicts recovery in stroke. *Annual International Conference of the IEEE Engineering in Medicine and Biology Society. IEEE Engineering in Medicine and Biology Society. Annual International Conference*, *2018*, 2507–2510. <https://doi.org/10.1109/EMBC.2018.8512737>（The reason for exclusion: duplicate record）
127. Campagnini, S., Liuzzi, P., Mannini, A., Riener, R., & Carrozza, M. C. (2022). Effects of control strategies on gait in robot-assisted post-stroke lower limb rehabilitation: a systematic review. *Journal of neuroengineering and rehabilitation*, *19*(1), 52. <https://doi.org/10.1186/s12984-022-01031-5>（The reason for exclusion: duplicate record）
128. Hsieh, Y. W., Lin, K. C., Horng, Y. S., Wu, C. Y., Wu, T. C., & Ku, F. L. (2014). Sequential combination of robot-assisted therapy and constraint-induced therapy in stroke rehabilitation: a randomized controlled trial. *Journal of neurology*, *261*(5), 1037–1045. <https://doi.org/10.1007/s00415-014-7345-4>（The reason for exclusion: duplicate record）
129. Kim, D. H., In, T. S., & Jung, K. S. (2022). Effects of robot-assisted trunk control training on trunk control ability and balance in patients with stroke: A randomized controlled trial. *Technology and health care : official journal of the European Society for Engineering and Medicine*, *30*(2), 413–422. <https://doi.org/10.3233/THC-202720>（The reason for exclusion: duplicate record）
130. Campagnini, S., Liuzzi, P., Mannini, A., Riener, R., & Carrozza, M. C. (2022). Effects of control strategies on gait in robot-assisted post-stroke lower limb rehabilitation: a systematic review. *Journal of neuroengineering and rehabilitation*, *19*(1), 52. <https://doi.org/10.1186/s12984-022-01031-5>（The reason for exclusion: duplicate record）
131. Hsieh, Y. W., Lin, K. C., Horng, Y. S., Wu, C. Y., Wu, T. C., & Ku, F. L. (2014). Sequential combination of robot-assisted therapy and constraint-induced therapy in stroke rehabilitation: a randomized controlled trial. *Journal of neurology*, *261*(5), 1037–1045. <https://doi.org/10.1007/s00415-014-7345-4>（The reason for exclusion: duplicate record）
132. Kim, D. H., In, T. S., & Jung, K. S. (2022). Effects of robot-assisted trunk control training on trunk control ability and balance in patients with stroke: A randomized controlled trial. *Technology and health care : official journal of the European Society for Engineering and Medicine*, *30*(2), 413–422. <https://doi.org/10.3233/THC-202720>（The reason for exclusion: duplicate record）
133. Lum, P. S., Godfrey, S. B., Brokaw, E. B., Holley, R. J., & Nichols, D. (2012). Robotic approaches for rehabilitation of hand function after stroke. *American journal of physical medicine & rehabilitation*, *91*(11 Suppl 3), S242–S254. <https://doi.org/10.1097/PHM.0b013e31826bcedb>（The reason for exclusion: duplicate record）
134. Maki, Y., Ii, T., Yamada, M., & Tanabe, S. (2024). Factors affecting the efficiency of walking independence in patients with subacute stroke following robot-assisted gait training with conventional rehabilitation. *International journal of rehabilitation research. Internationale Zeitschrift fur Rehabilitationsforschung. Revue internationale de recherches de readaptation*, *47*(1), 26–33. <https://doi.org/10.1097/MRR.0000000000000609>（The reason for exclusion: duplicate record）
135. Akinci, M., Burak, M., Kasal, F. Z., Özaslan, E. A., Huri, M., & Kurtaran, Z. A. (2024). The Effects of Combined Virtual Reality Exercises and Robot Assisted Gait Training on Cognitive Functions, Daily Living Activities, and Quality of Life in High Functioning Individuals With Subacute Stroke. *Perceptual and motor skills*, *131*(3), 756–769. <https://doi.org/10.1177/00315125241235420>（The reason for exclusion: duplicate record）
136. Valero-Cuevas, F. J., Klamroth-Marganska, V., Winstein, C. J., & Riener, R. (2016). Robot-assisted and conventional therapies produce distinct rehabilitative trends in stroke survivors. *Journal of neuroengineering and rehabilitation*, *13*(1), 92. <https://doi.org/10.1186/s12984-016-0199-5>（The reason for exclusion: duplicate record）
137. Liang, S., Hong, Z. Q., Cai, Q., Gao, H. G., Ren, Y. J., Zheng, H. Q., Chen, X., & Hu, X. Q. (2024). Effects of robot-assisted gait training on motor performance of lower limb in poststroke survivors: a systematic review with meta-analysis. *European review for medical and pharmacological sciences*, *28*(3), 879–898. <https://doi.org/10.26355/eurrev_202402_35325>（The reason for exclusion: review）
138. Ghasemi, A., Sadedel, M., & Moghaddam, M. M. (2024). A wearable system to assist impaired-neck patients: Design and evaluation. *Proceedings of the Institution of Mechanical Engineers. Part H, Journal of engineering in medicine*, *238*(1), 63–77. <https://doi.org/10.1177/09544119231211362>（The reason for exclusion: duplicate record）
139. Cindy J H, R., Prange-Lasonder, G. B., Prinsen, E. C., Buurke, J. H., & Rietman, J. S. (2022). Detection thresholds for electrostimulation combined with robotic leg support in sub-acute stroke patients. *IEEE ... International Conference on Rehabilitation Robotics : [proceedings]*, *2022*, 1–5. <https://doi.org/10.1109/ICORR55369.2022.9896576>（The reason for exclusion: duplicate record）
140. Schicketmueller, A., Lamprecht, J., Hofmann, M., Sailer, M., & Rose, G. (2020). Gait Event Detection for Stroke Patients during Robot-Assisted Gait Training. *Sensors (Basel, Switzerland)*, *20*(12), 3399. <https://doi.org/10.3390/s20123399>（The reason for exclusion: duplicate record）
141. Ueba, T., Hamada, O., Ogata, T., Inoue, T., Shiota, E., & Sankai, Y. (2013). Feasibility and safety of acute phase rehabilitation after stroke using the hybrid assistive limb robot suit. *Neurologia medico-chirurgica*, *53*(5), 287–290. <https://doi.org/10.2176/nmc.53.287>（The reason for exclusion: duplicate record）
142. Fernandez-Garcia, C., Ternent, L., Homer, T. M., Rodgers, H., Bosomworth, H., Shaw, L., Aird, L., Andole, S., Cohen, D., Dawson, J., Finch, T., Ford, G., Francis, R., Hogg, S., Hughes, N., Krebs, H. I., Price, C., Turner, D., Van Wijck, F., Wilkes, S., … Vale, L. (2021). Economic evaluation of robot-assisted training versus an enhanced upper limb therapy programme or usual care for patients with moderate or severe upper limb functional limitation due to stroke: results from the RATULS randomised controlled trial. *BMJ open*, *11*(5), e042081. <https://doi.org/10.1136/bmjopen-2020-042081>（The reason for exclusion: duplicate record）
143. Guo, Z., Zhou, S., Ji, K., Zhuang, Y., Song, J., Nam, C., Hu, X., & Zheng, Y. (2022). Corticomuscular integrated representation of voluntary motor effort in robotic control for wrist-hand rehabilitation after stroke. *Journal of neural engineering*, *19*(2), 10.1088/1741-2552/ac5757. <https://doi.org/10.1088/1741-2552/ac5757>（The reason for exclusion: duplicate record）
144. Lin, Y., Li, Q. Y., Qu, Q., Ding, L., Chen, Z., Huang, F., Hu, S., Deng, W., Guo, F., Wang, C., Deng, P., Li, L., Jin, H., Gao, C., Shu, B., & Jia, J. (2022). Comparative Effectiveness of Robot-Assisted Training Versus Enhanced Upper Extremity Therapy on Upper and Lower Extremity for Stroke Survivors: A Multicentre Randomized Controlled Trial. *Journal of rehabilitation medicine*, *54*, jrm00314. <https://doi.org/10.2340/jrm.v54.882>（The reason for exclusion: duplicate record）
145. Vales, Y., Catalan, J. M., Bertomeu-Motos, A., Garcia-Perez, J. V., Lledo, L. D., Blanco-Ivorra, A., Marzo, C. A., Mas, G., & Garcia-Aracil, N. (2023). Influence of Robotic Therapy on Severe Stroke Patients. *IEEE ... International Conference on Rehabilitation Robotics : [proceedings]*, *2023*, 1–6. <https://doi.org/10.1109/ICORR58425.2023.10304780>（The reason for exclusion: duplicate record）
146. Gupta, A., Prakash, N. B., Sannyasi, G., Mohamad, F., Honavar, P., Jotheeswaran, S., Khanna, M., & Ramakrishnan, S. (2023). Effect of overground gait training with 'Mobility Assisted Robotic System-MARS' on gait parameters in patients with stroke: a pre-post study. *BMC neurology*, *23*(1), 296. <https://doi.org/10.1186/s12883-023-03357-6>（The reason for exclusion: Non-RCTs）
147. Park, J. H., Park, G., Kim, H. Y., Lee, J. Y., Ham, Y., Hwang, D., Kwon, S., & Shin, J. H. (2020). A comparison of the effects and usability of two exoskeletal robots with and without robotic actuation for upper extremity rehabilitation among patients with stroke: a single-blinded randomised controlled pilot study. *Journal of neuroengineering and rehabilitation*, *17*(1), 137. <https://doi.org/10.1186/s12984-020-00763-6>（The reason for exclusion: duplicate record）
148. Bazan, R., Fonseca, B. H. S., Miranda, J. M. A., Nunes, H. R. C., Bazan, S. G. Z., & Luvizutto, G. J. (2022). Effect of Robot-Assisted Training on Unilateral Spatial Neglect After Stroke: Systematic Review and Meta-Analysis of Randomized Controlled Trials. *Neurorehabilitation and neural repair*, *36*(8), 545–556. <https://doi.org/10.1177/15459683221110894>（The reason for exclusion: review）
149. Norouzi-Gheidari, N., Archambault, P. S., & Fung, J. (2012). Effects of robot-assisted therapy on stroke rehabilitation in upper limbs: systematic review and meta-analysis of the literature. *Journal of rehabilitation research and development*, *49*(4), 479–496. <https://doi.org/10.1682/jrrd.2010.10.0210>（The reason for exclusion: review）
150. Bosomworth, H., Rodgers, H., Shaw, L., Smith, L., Aird, L., Howel, D., Wilson, N., Alvarado, N., Andole, S., Cohen, D. L., Dawson, J., Fernandez-Garcia, C., Finch, T., Ford, G. A., Francis, R., Hogg, S., Hughes, N., Price, C. I., Ternent, L., Turner, D. L., … van Wijck, F. (2021). Evaluation of the enhanced upper limb therapy programme within the Robot-Assisted Training for the Upper Limb after Stroke trial: descriptive analysis of intervention fidelity, goal selection and goal achievement. *Clinical rehabilitation*, *35*(1), 119–134. <https://doi.org/10.1177/0269215520953833>（The reason for exclusion: duplicate record）
151. Hidler, J., Nichols, D., Pelliccio, M., & Brady, K. (2005). Advances in the understanding and treatment of stroke impairment using robotic devices. *Topics in stroke rehabilitation*, *12*(2), 22–35. <https://doi.org/10.1310/RYT5-62N4-CTVX-8JTE>（The reason for exclusion: review）
152. Hsu, C. Y., Cheng, Y. H., Lai, C. H., & Lin, Y. N. (2020). Clinical non-superiority of technology-assisted gait training with body weight support in patients with subacute stroke: A meta-analysis. *Annals of physical and rehabilitation medicine*, *63*(6), 535–542. <https://doi.org/10.1016/j.rehab.2019.09.009>（The reason for exclusion: review）
153. Morizio, C., Compagnat, M., Boujut, A., Labbani-Igbida, O., Billot, M., & Perrochon, A. (2022). Immersive Virtual Reality during Robot-Assisted Gait Training: Validation of a New Device in Stroke Rehabilitation. *Medicina (Kaunas, Lithuania)*, *58*(12), 1805. <https://doi.org/10.3390/medicina58121805>（The reason for exclusion: duplicate record）
154. Arantes, A. P., Bressan, N., Borges, L. R., & McGibbon, C. A. (2023). Evaluation of a novel real-time adaptive assist-as-needed controller for robot-assisted upper extremity rehabilitation following stroke. *PloS one*, *18*(10), e0292627. <https://doi.org/10.1371/journal.pone.0292627>（The reason for exclusion: duplicate record）
155. Hsieh, Y. W., Lin, K. C., Wu, C. Y., Lien, H. Y., Chen, J. L., Chen, C. C., & Chang, W. H. (2014). Predicting clinically significant changes in motor and functional outcomes after robot-assisted stroke rehabilitation. *Archives of physical medicine and rehabilitation*, *95*(2), 316–321. <https://doi.org/10.1016/j.apmr.2013.09.018>（The reason for exclusion: duplicate record）
156. Lo, K., Stephenson, M., & Lockwood, C. (2017). Effectiveness of robotic assisted rehabilitation for mobility and functional ability in adult stroke patients: a systematic review. *JBI database of systematic reviews and implementation reports*, *15*(12), 3049–3091. <https://doi.org/10.11124/JBISRIR-2017-003456>（The reason for exclusion: duplicate record）
157. Tanaka, H., Nankaku, M., Kikuchi, T., Nishi, H., Nishikawa, T., Yonezawa, H., Kitamura, G., Takagi, Y., Miyamoto, S., Ikeguchi, R., & Matsuda, S. (2021). Effects of periodic robot rehabilitation using the Hybrid Assistive Limb for a year on gait function in chronic stroke patients. *Journal of clinical neuroscience : official journal of the Neurosurgical Society of Australasia*, *92*, 17–21. <https://doi.org/10.1016/j.jocn.2021.07.040>（The reason for exclusion: duplicate record）
158. Rinderknecht, M. D., Lambercy, O., Raible, V., Büsching, I., Sehle, A., Liepert, J., & Gassert, R. (2018). Reliability, validity, and clinical feasibility of a rapid and objective assessment of post-stroke deficits in hand proprioception. *Journal of neuroengineering and rehabilitation*, *15*(1), 47. <https://doi.org/10.1186/s12984-018-0387-6>（The reason for exclusion: duplicate record）
159. Montedoro, V., Alsamour, M., Dehem, S., Lejeune, T., Dehez, B., & Edwards, M. G. (2019). Robot Diagnosis Test for Egocentric and Allocentric Hemineglect. *Archives of clinical neuropsychology : the official journal of the National Academy of Neuropsychologists*, *34*(4), 481–494. <https://doi.org/10.1093/arclin/acy062>（The reason for exclusion: duplicate record）
160. Hsu, H. Y., Koh, C. L., Yang, K. C., Lin, Y. C., Hsu, C. H., Su, F. C., & Kuo, L. C. (2024). Effects of an assist-as-needed equipped Tenodesis-Induced-Grip Exoskeleton Robot (TIGER) on upper limb function in patients with chronic stroke. *Journal of neuroengineering and rehabilitation*, *21*(1), 5. <https://doi.org/10.1186/s12984-023-01298-2>（The reason for exclusion: duplicate record）
161. Huo, C., Shao, G., Chen, T., Li, W., Wang, J., Xie, H., Wang, Y., Li, Z., Zheng, P., Li, L., & Li, L. (2024). Effectiveness of unilateral lower-limb exoskeleton robot on balance and gait recovery and neuroplasticity in patients with subacute stroke: a randomized controlled trial. *Journal of neuroengineering and rehabilitation*, *21*(1), 213. <https://doi.org/10.1186/s12984-024-01493-9>（The reason for exclusion: duplicate record）
162. Norouzi-Gheidari, N., Archambault, P. S., & Fung, J. (2019). Robot-Assisted Reaching Performance of Chronic Stroke and Healthy Individuals in a Virtual Versus a Physical Environment: A Pilot Study. *IEEE transactions on neural systems and rehabilitation engineering : a publication of the IEEE Engineering in Medicine and Biology Society*, *27*(6), 1273–1281. <https://doi.org/10.1109/TNSRE.2019.2914015>（The reason for exclusion: duplicate record）
163. Tedla, J. S., Dixit, S., Gular, K., & Abohashrh, M. (2019). Robotic-Assisted Gait Training Effect on Function and Gait Speed in Subacute and Chronic Stroke Population: A Systematic Review and Meta-Analysis of Randomized Controlled Trials. *European neurology*, *81*(3-4), 103–111. <https://doi.org/10.1159/000500747>（The reason for exclusion: review）
164. Mazzoleni, S., Sale, P., Tiboni, M., Franceschini, M., Carrozza, M. C., & Posteraro, F. (2013). Upper limb robot-assisted therapy in chronic and subacute stroke patients: a kinematic analysis. *American journal of physical medicine & rehabilitation*, *92*(10 Suppl 2), e26–e37. <https://doi.org/10.1097/PHM.0b013e3182a1e852>（The reason for exclusion: duplicate record）
165. Maeshima, S., Osawa, A., Nishio, D., Hirano, Y., Takeda, K., Kigawa, H., & Sankai, Y. (2011). Efficacy of a hybrid assistive limb in post-stroke hemiplegic patients: a preliminary report. *BMC neurology*, *11*, 116. <https://doi.org/10.1186/1471-2377-11-116>（The reason for exclusion: duplicate record）
166. Koyama, S., Tanabe, S., Otaka, Y., Kato, T., Furuzawa, S., Tatemoto, T., Kumazawa, N., Yoshimuta, H., Torii, K., Tsukada, S., & Saitoh, E. (2022). Novel lateral transfer assist robot decreases the difficulty of transfer in post-stroke hemiparesis patients: a pilot study. *Disability and rehabilitation. Assistive technology*, *17*(7), 828–832. <https://doi.org/10.1080/17483107.2020.1818136>（The reason for exclusion: duplicate record）
167. Pennycott, A., Wyss, D., Vallery, H., Klamroth-Marganska, V., & Riener, R. (2012). Towards more effective robotic gait training for stroke rehabilitation: a review. *Journal of neuroengineering and rehabilitation*, *9*, 65. <https://doi.org/10.1186/1743-0003-9-65>（The reason for exclusion: duplicate record）
168. Liu, Q., Liu, L., Liu, Z., Xu, Y., Wang, F., Cheng, H., & Hu, X. (2024). Reminiscent music therapy combined with robot-assisted rehabilitation for elderly stroke patients: a pilot study. *Journal of neuroengineering and rehabilitation*, *21*(1), 16. <https://doi.org/10.1186/s12984-024-01315-y>（The reason for exclusion: duplicate record）
169. Flynn, N., Froude, E., Cooke, D., & Kuys, S. (2022). Repetitions, duration and intensity of upper limb practice following the implementation of robot assisted therapy with sub-acute stroke survivors: an observational study. *Disability and rehabilitation. Assistive technology*, *17*(6), 675–680. <https://doi.org/10.1080/17483107.2020.1807621>（The reason for exclusion: duplicate record）
170. Balch, M. H. H., Harris, H., Chugh, D., Gnyawali, S., Rink, C., Nimjee, S. M., & Arnold, W. D. (2021). Ischemic stroke-induced polyaxonal innervation at the neuromuscular junction is attenuated by robot-assisted mechanical therapy. *Experimental neurology*, *343*, 113767. <https://doi.org/10.1016/j.expneurol.2021.113767>（The reason for exclusion: duplicate record）
171. Dai, L., Zhang, W., Zhang, H., Fang, L., Chen, J., Li, X., Yu, H., Song, J., Chen, S., Zheng, B., Zhang, Y., & Li, Z. (2024). Effects of robot-assisted upper limb training combined with intermittent theta burst stimulation (iTBS) on cortical activation in stroke patients: A functional near-infrared spectroscopy study. *NeuroRehabilitation*, *54*(3), 421–434. <https://doi.org/10.3233/NRE-230355>（The reason for exclusion: duplicate record）
172. Torrisi, M., De Cola, M. C., Buda, A., Carioti, L., Scaltrito, M. V., Bramanti, P., Manuli, A., De Luca, R., & Calabrò, R. S. (2018). Self-Efficacy, Poststroke Depression, and Rehabilitation Outcomes: Is There a Correlation?. *Journal of stroke and cerebrovascular diseases : the official journal of National Stroke Association*, *27*(11), 3208–3211. <https://doi.org/10.1016/j.jstrokecerebrovasdis.2018.07.021>（The reason for exclusion: duplicate record）
173. Hu, X. L., Tong, K. Y., Li, R., Xue, J. J., Ho, S. K., & Chen, P. (2012). The effects of electromechanical wrist robot assistive system with neuromuscular electrical stimulation for stroke rehabilitation. *Journal of electromyography and kinesiology : official journal of the International Society of Electrophysiological Kinesiology*, *22*(3), 431–439. <https://doi.org/10.1016/j.jelekin.2011.12.010>（The reason for exclusion: duplicate record）
174. Hamedani, M., Prada, V., Tognetti, P., Leoni, V., & Schenone, A. (2022). Robot-assisted and traditional intensive rehabilitation therapy in the treatment of post-acute stroke patient: the experience of a standard rehabilitation ward. *Neurological sciences : official journal of the Italian Neurological Society and of the Italian Society of Clinical Neurophysiology*, *43*(6), 3999–4001. <https://doi.org/10.1007/s10072-022-06041-8>（The reason for exclusion: duplicate record）
175. Kuczynski, A. M., Dukelow, S. P., Semrau, J. A., & Kirton, A. (2016). Robotic Quantification of Position Sense in Children With Perinatal Stroke. *Neurorehabilitation and neural repair*, *30*(8), 762–772. <https://doi.org/10.1177/1545968315624781>（The reason for exclusion: duplicate record）
176. Varas-Diaz, G., Cordo, P., Dusane, S., & Bhatt, T. (2022). Effect of robotic-assisted ankle training on gait in stroke participants: A case series study. *Physiotherapy theory and practice*, *38*(13), 2973–2982. <https://doi.org/10.1080/09593985.2021.1964658>（The reason for exclusion: duplicate record）
177. Forrester, L. W., Roy, A., Hafer-Macko, C., Krebs, H. I., & Macko, R. F. (2016). Task-specific ankle robotics gait training after stroke: a randomized pilot study. *Journal of neuroengineering and rehabilitation*, *13*(1), 51. <https://doi.org/10.1186/s12984-016-0158-1>（The reason for exclusion: duplicate record）
178. Zengin-Metli, D., Özbudak-Demir, S., Eraktaş, İ., Binay-Safer, V., & Ekiz, T. (2018). Effects of robot assistive upper extremity rehabilitation on motor and cognitive recovery, the quality of life, and activities of daily living in stroke patients. *Journal of back and musculoskeletal rehabilitation*, *31*(6), 1059–1064. <https://doi.org/10.3233/BMR-171015>（The reason for exclusion: duplicate record）
179. Chockalingam, M., Vasanthan, L. T., Balasubramanian, S., & Sriram, V. (2022). Experiences of patients who had a stroke and rehabilitation professionals with upper limb rehabilitation robots: a qualitative systematic review protocol. *BMJ open*, *12*(9), e065177. <https://doi.org/10.1136/bmjopen-2022-065177>（The reason for exclusion: duplicate record）
180. Wei, D., Hua, X. Y., Zheng, M. X., Wu, J. J., & Xu, J. G. (2022). Effectiveness of robot-assisted virtual reality mirror therapy for upper limb motor dysfunction after stroke: study protocol for a single-center randomized controlled clinical trial. *BMC neurology*, *22*(1), 307. <https://doi.org/10.1186/s12883-022-02836-6>（The reason for exclusion: Non robot-assisted task-oriented training）
181. Bishop, L., Omofuma, I., Stein, J., Agrawal, S., & Quinn, L. (2020). Treadmill-Based Locomotor Training With Robotic Pelvic Assist and Visual Feedback: A Feasibility Study. *Journal of neurologic physical therapy : JNPT*, *44*(3), 205–213. <https://doi.org/10.1097/NPT.0000000000000317>（The reason for exclusion: Non robot-assisted task-oriented training）
182. Bennett, T., Kumar, P., & Garate, V. R. (2022). A Machine Learning Model for Predicting Sit-to-Stand Trajectories of People with and without Stroke: Towards Adaptive Robotic Assistance. *Sensors (Basel, Switzerland)*, *22*(13), 4789. <https://doi.org/10.3390/s22134789>（The reason for exclusion: Non robot-assisted task-oriented training）
183. Lamberti, N., Manfredini, F., Lissom, L. O., Lavezzi, S., Basaglia, N., & Straudi, S. (2021). Beneficial Effects of Robot-Assisted Gait Training on Functional Recovery in Women after Stroke: A Cohort Study. *Medicina (Kaunas, Lithuania)*, *57*(11), 1200. <https://doi.org/10.3390/medicina57111200>（The reason for exclusion: Non robot-assisted task-oriented training）
184. He, Y. Z., Huang, Z. M., Deng, H. Y., Huang, J., Wu, J. H., & Wu, J. S. (2023). Feasibility, safety, and efficacy of task-oriented mirrored robotic training on upper-limb functions and activities of daily living in subacute poststroke patients: a pilot study. *European journal of physical and rehabilitation medicine*, *59*(6), 660–668. <https://doi.org/10.23736/S1973-9087.23.08018-8>（The reason for exclusion: duplicate record）
185. Morone, G., Masiero, S., Coiro, P., De Angelis, D., Venturiero, V., Paolucci, S., & Iosa, M. (2018). Clinical features of patients who might benefit more from walking robotic training. *Restorative neurology and neuroscience*, *36*(2), 293–299. <https://doi.org/10.3233/RNN-170799>（The reason for exclusion: duplicate record）
186. Elmanowski, J., Kleynen, M., Geers, R. P. J., Rovelo-Ruiz, G., Geurts, E., Coninx, K., Verbunt, J. A., & Seelen, H. A. M. (2023). Task-oriented arm training for stroke patients based on remote handling technology concepts: A feasibility study. *Technology and health care : official journal of the European Society for Engineering and Medicine*, *31*(5), 1593–1605. <https://doi.org/10.3233/THC-220465>（The reason for exclusion: duplicate record）
187. Kuo, L. C., Yang, K. C., Lin, Y. C., Lin, Y. C., Yeh, C. H., Su, F. C., & Hsu, H. Y. (2023). Internet of Things (IoT) Enables Robot-Assisted Therapy as a Home Program for Training Upper Limb Functions in Chronic Stroke: A Randomized Control Crossover Study. *Archives of physical medicine and rehabilitation*, *104*(3), 363–371. <https://doi.org/10.1016/j.apmr.2022.08.976>（The reason for exclusion: duplicate record）
188. Serrano-López Terradas, P. A., Criado Ferrer, T., Jakob, I., & Calvo-Arenillas, J. I. (2022). Quo Vadis, Amadeo Hand Robot? A Randomized Study with a Hand Recovery Predictive Model in Subacute Stroke. *International journal of environmental research and public health*, *20*(1), 690. <https://doi.org/10.3390/ijerph20010690>（The reason for exclusion: duplicate record）
189. Abedi, M., Moghaddam, M. M., & Fallah, D. (2018). A Poincare map based analysis of stroke patients' walking after a rehabilitation by a robot. *Mathematical biosciences*, *299*, 73–84. <https://doi.org/10.1016/j.mbs.2018.03.001>（The reason for exclusion: Non robot-assisted task-oriented training）
190. Pignolo, L., Servidio, R., Basta, G., Carozzo, S., Tonin, P., Calabrò, R. S., & Cerasa, A. (2021). The Route of Motor Recovery in Stroke Patients Driven by Exoskeleton-Robot-Assisted Therapy: A Path-Analysis. *Medical sciences (Basel, Switzerland)*, *9*(4), 64. <https://doi.org/10.3390/medsci9040064>（The reason for exclusion: Non robot-assisted task-oriented training）
191. Zhou, Z. Q., Hua, X. Y., Wu, J. J., Xu, J. J., Ren, M., Shan, C. L., & Xu, J. G. (2022). Combined robot motor assistance with neural circuit-based virtual reality (NeuCir-VR) lower extremity rehabilitation training in patients after stroke: a study protocol for a single-centre randomised controlled trial. *BMJ open*, *12*(12), e064926. <https://doi.org/10.1136/bmjopen-2022-064926>（The reason for exclusion: Non robot-assisted task-oriented training）
192. Aisen, M. L., Krebs, H. I., Hogan, N., McDowell, F., & Volpe, B. T. (1997). The effect of robot-assisted therapy and rehabilitative training on motor recovery following stroke. *Archives of neurology*, *54*(4), 443–446. <https://doi.org/10.1001/archneur.1997.00550160075019>（The reason for exclusion: Non robot-assisted task-oriented training）
193. Amano, Y., Noma, T., Etoh, S., Miyata, R., Kawamura, K., & Shimodozono, M. (2020). Reaching exercise for chronic paretic upper extremity after stroke using a novel rehabilitation robot with arm-weight support and concomitant electrical stimulation and vibration: before-and-after feasibility trial. *Biomedical engineering online*, *19*(1), 28. <https://doi.org/10.1186/s12938-020-00774-3>（The reason for exclusion: Non robot-assisted task-oriented training）
194. Tanaka, N., Matsushita, S., Sonoda, Y., Maruta, Y., Fujitaka, Y., Sato, M., Simomori, M., Onaka, R., Harada, K., Hirata, T., Kinoshita, S., Okamoto, T., & Okamura, H. (2019). Effect of Stride Management Assist Gait Training for Poststroke Hemiplegia: A Single Center, Open-Label, Randomized Controlled Trial. *Journal of stroke and cerebrovascular diseases : the official journal of National Stroke Association*, *28*(2), 477–486. <https://doi.org/10.1016/j.jstrokecerebrovasdis.2018.10.025>（The reason for exclusion: Non robot-assisted task-oriented training）
195. Jamin, P., Duret, C., Hutin, E., Bayle, N., Koeppel, T., Gracies, J. M., & Pila, O. (2022). Using Robot-Based Variables during Upper Limb Robot-Assisted Training in Subacute Stroke Patients to Quantify Treatment Dose. *Sensors (Basel, Switzerland)*, *22*(8), 2989. <https://doi.org/10.3390/s22082989>（The reason for exclusion: Non robot-assisted task-oriented training）
196. Iwamoto, Y., Tanaka, R., Imura, T., Mitsutake, T., Jung, H., Suzukawa, T., Taki, S., Imada, N., Inagawa, T., Araki, H., & Araki, O. (2023). Does frequent use of an exoskeletal upper limb robot improve motor function in stroke patients?. *Disability and rehabilitation*, *45*(7), 1185–1191. <https://doi.org/10.1080/09638288.2022.2055163>（The reason for exclusion: Non robot-assisted task-oriented training）
197. Chinembiri, B., Ming, Z., Kai, S., Xiu Fang, Z., & Wei, C. (2021). The fourier M2 robotic machine combined with occupational therapy on post-stroke upper limb function and independence-related quality of life: A randomized clinical trial. *Topics in stroke rehabilitation*, *28*(1), 1–18. <https://doi.org/10.1080/10749357.2020.1755815>（The reason for exclusion: Non robot-assisted task-oriented training）
198. Rosati G. (2010). The place of robotics in post-stroke rehabilitation. *Expert review of medical devices*, *7*(6), 753–758. <https://doi.org/10.1586/erd.10.49>（The reason for exclusion: Non robot-assisted task-oriented training）
199. Li, D. X., Zha, F. B., Long, J. J., Liu, F., Cao, J., & Wang, Y. L. (2021). Effect of Robot Assisted Gait Training on Motor and Walking Function in Patients with Subacute Stroke: A Random Controlled Study. *Journal of stroke and cerebrovascular diseases : the official journal of National Stroke Association*, *30*(7), 105807. <https://doi.org/10.1016/j.jstrokecerebrovasdis.2021.105807>（The reason for exclusion: duplicate record）
200. Bourke, T. C., Lowrey, C. R., Dukelow, S. P., Bagg, S. D., Norman, K. E., & Scott, S. H. (2016). A robot-based behavioural task to quantify impairments in rapid motor decisions and actions after stroke. *Journal of neuroengineering and rehabilitation*, *13*(1), 91. <https://doi.org/10.1186/s12984-016-0201-2>（The reason for exclusion: duplicate record）
201. Su, T., Wang, M., Chen, Z., & Feng, L. (2024). Effect of Upper Robot-Assisted Training on Upper Limb Motor, Daily Life Activities, and Muscular Tone in Patients With Stroke: A Systematic Review and Meta-Analysis. *Brain and behavior*, *14*(11), e70117. <https://doi.org/10.1002/brb3.70117>（The reason for exclusion: review）
202. Baldan, F., Turolla, A., Rimini, D., Pregnolato, G., Maistrello, L., Agostini, M., & Jakob, I. (2021). Robot-assisted rehabilitation of hand function after stroke: Development of prediction models for reference to therapy. *Journal of electromyography and kinesiology : official journal of the International Society of Electrophysiological Kinesiology*, *57*, 102534. <https://doi.org/10.1016/j.jelekin.2021.102534>（The reason for exclusion: duplicate record）
203. Awad, L. N., Esquenazi, A., Francisco, G. E., Nolan, K. J., & Jayaraman, A. (2020). The ReWalk ReStore™ soft robotic exosuit: a multi-site clinical trial of the safety, reliability, and feasibility of exosuit-augmented post-stroke gait rehabilitation. *Journal of neuroengineering and rehabilitation*, *17*(1), 80. <https://doi.org/10.1186/s12984-020-00702-5>（The reason for exclusion: duplicate record）
204. Gasperina, S. D., Longatelli, V., Panzenbeck, M., Luciani, B., Morosini, A., Piantoni, A., Tropea, P., Braghin, F., Pedrocchi, A., & Gandolla, M. (2022). AGREE: an upper-limb robotic platform for personalized rehabilitation, concept and clinical study design. *IEEE ... International Conference on Rehabilitation Robotics : [proceedings]*, *2022*, 1–6. <https://doi.org/10.1109/ICORR55369.2022.9896569>（The reason for exclusion: duplicate record）
205. Krebs, H. I., Ladenheim, B., Hippolyte, C., Monterroso, L., & Mast, J. (2009). Robot-assisted task-specific training in cerebral palsy. *Developmental medicine and child neurology*, *51 Suppl 4*, 140–145. <https://doi.org/10.1111/j.1469-8749.2009.03416.x>（The reason for exclusion: duplicate record）
206. Pila, O., Duret, C., Koeppel, T., & Jamin, P. (2023). Performance-Based Robotic Training in Individuals with Subacute Stroke: Differences between Responders and Non-Responders. *Sensors (Basel, Switzerland)*, *23*(9), 4304. <https://doi.org/10.3390/s23094304>（The reason for exclusion: duplicate record）
207. Nedergård, H., Sandlund, M., Häger, C. K., & Palmcrantz, S. (2023). Users' experiences of intensive robotic-assisted gait training post-stroke - "a push forward or feeling pushed around?". *Disability and rehabilitation*, *45*(23), 3861–3868. <https://doi.org/10.1080/09638288.2022.2140848>（The reason for exclusion: duplicate record）
208. Hawe, R. L., Kuczynski, A. M., Kirton, A., & Dukelow, S. P. (2020). Robotic assessment of rapid motor decision making in children with perinatal stroke. *Journal of neuroengineering and rehabilitation*, *17*(1), 94. <https://doi.org/10.1186/s12984-020-00714-1>（The reason for exclusion: duplicate record）
209. Reis, S. B., Bernardo, W. M., Oshiro, C. A., Krebs, H. I., & Conforto, A. B. (2021). Effects of Robotic Therapy Associated With Noninvasive Brain Stimulation on Upper-Limb Rehabilitation After Stroke: Systematic Review and Meta-analysis of Randomized Clinical Trials. *Neurorehabilitation and neural repair*, *35*(3), 256–266. https://doi.org/10.1177/1545968321989353（The reason for exclusion: review）
210. Park, S., Fraser, M., Weber, L. M., Meeker, C., Bishop, L., Geller, D., Stein, J., & Ciocarlie, M. (2020). User-Driven Functional Movement Training With a Wearable Hand Robot After Stroke. *IEEE transactions on neural systems and rehabilitation engineering : a publication of the IEEE Engineering in Medicine and Biology Society*, *28*(10), 2265–2275. <https://doi.org/10.1109/TNSRE.2020.3021691>（The reason for exclusion: duplicate record）
211. Scano, A., Chiavenna, A., Caimmi, M., Malosio, M., Tosatti, L. M., & Molteni, F. (2017). Effect of human-robot interaction on muscular synergies on healthy people and post-stroke chronic patients. *IEEE ... International Conference on Rehabilitation Robotics : [proceedings]*, *2017*, 527–532. <https://doi.org/10.1109/ICORR.2017.8009302>（The reason for exclusion: duplicate record）
212. Lee, M. J., Lee, J. H., & Lee, S. M. (2018). Effects of robot-assisted therapy on upper extremity function and activities of daily living in hemiplegic patients: A single-blinded, randomized, controlled trial. *Technology and health care : official journal of the European Society for Engineering and Medicine*, *26*(4), 659–666. <https://doi.org/10.3233/THC-181336>（The reason for exclusion: duplicate record）
213. Hsu, H. Y., Yang, K. C., Yeh, C. H., Lin, Y. C., Lin, K. R., Su, F. C., & Kuo, L. C. (2022). A Tenodesis-Induced-Grip exoskeleton robot (TIGER) for assisting upper extremity functions in stroke patients: a randomized control study. *Disability and rehabilitation*, *44*(23), 7078–7086. <https://doi.org/10.1080/09638288.2021.1980915>（The reason for exclusion: duplicate record）
214. Ahmed, T., Islam, M. R., Brahmi, B., & Rahman, M. H. (2022). Robustness and Tracking Performance Evaluation of PID Motion Control of 7 DoF Anthropomorphic Exoskeleton Robot Assisted Upper Limb Rehabilitation. *Sensors (Basel, Switzerland)*, *22*(10), 3747. <https://doi.org/10.3390/s22103747>（The reason for exclusion: duplicate record）
215. Caimmi, M., Chiavenna, A., Scano, A., Gasperini, G., Giovanzana, C., Molinari Tosatti, L., & Molteni, F. (2017). Using robot fully assisted functional movements in upper-limb rehabilitation of chronic stroke patients: preliminary results. *European journal of physical and rehabilitation medicine*, *53*(3), 390–399. <https://doi.org/10.23736/S1973-9087.16.04407-5>（The reason for exclusion: duplicate record）
216. Kim, G. J., Taub, M., Creelman, C., Cahalan, C., O'Dell, M. W., & Stein, J. (2019). Feasibility of an Electromyography-Triggered Hand Robot for People After Chronic Stroke. *The American journal of occupational therapy : official publication of the American Occupational Therapy Association*, *73*(4), 7304345040p1–7304345040p9. <https://doi.org/10.5014/ajot.2019.030908>（The reason for exclusion: duplicate record）
217. Zhou, J., Peng, H., Zheng, M., Wei, Z., Fan, T., & Song, R. (2024). Trajectory Deformation-Based Multi-Modal Adaptive Compliance Control for a Wearable Lower Limb Rehabilitation Robot. *IEEE transactions on neural systems and rehabilitation engineering : a publication of the IEEE Engineering in Medicine and Biology Society*, *32*, 314–324. <https://doi.org/10.1109/TNSRE.2023.3348332>（The reason for exclusion: duplicate record）
218. Pila, O., Duret, C., Gracies, J. M., Francisco, G. E., Bayle, N., & Hutin, É. (2018). Evolution of upper limb kinematics four years after subacute robot-assisted rehabilitation in stroke patients. *The International journal of neuroscience*, *128*(11), 1030–1039. <https://doi.org/10.1080/00207454.2018.1461626>（The reason for exclusion: duplicate record）
219. Lo, A. C., Guarino, P., Krebs, H. I., Volpe, B. T., Bever, C. T., Duncan, P. W., Ringer, R. J., Wagner, T. H., Richards, L. G., Bravata, D. M., Haselkorn, J. K., Wittenberg, G. F., Federman, D. G., Corn, B. H., Maffucci, A. D., & Peduzzi, P. (2009). Multicenter randomized trial of robot-assisted rehabilitation for chronic stroke: methods and entry characteristics for VA ROBOTICS. *Neurorehabilitation and neural repair*, *23*(8), 775–783. <https://doi.org/10.1177/1545968309338195>（The reason for exclusion: duplicate record）
220. Hung, C. S., Hsieh, Y. W., Wu, C. Y., Lin, Y. T., Lin, K. C., & Chen, C. L. (2016). The Effects of Combination of Robot-Assisted Therapy With Task-Specific or Impairment-Oriented Training on Motor Function and Quality of Life in Chronic Stroke. *PM & R : the journal of injury, function, and rehabilitation*, *8*(8), 721–729. <https://doi.org/10.1016/j.pmrj.2016.01.008>（The reason for exclusion: duplicate record）
221. Lee, S. Y., Choi, Y. S., Kim, M. H., & Chang, W. N. (2024). Effects of robot-assisted walking training on balance, motor function, and ADL depending on severity levels in stroke patients. *Technology and health care : official journal of the European Society for Engineering and Medicine*, *32*(5), 3293–3307. <https://doi.org/10.3233/THC-232015>（The reason for exclusion: duplicate record）
222. Bay, B., Kiwus, L. M., Goßling, A., Koester, L., Blaum, C., Schrage, B., Clemmensen, P., Blankenberg, S., Waldeyer, C., Seiffert, M., & Brunner, F. J. (2024). Procedural and one-year outcomes of robotic-assisted versus manual percutaneous coronary intervention. *EuroIntervention : journal of EuroPCR in collaboration with the Working Group on Interventional Cardiology of the European Society of Cardiology*, *20*(1), 56–65. <https://doi.org/10.4244/EIJ-D-23-00375>（The reason for exclusion: duplicate record）
223. Devittori, G., Ranzani, R., Dinacci, D., Romiti, D., Califfi, A., Petrillo, C., Rossi, P., Gassert, R., & Lambercy, O. (2022). Automatic and Personalized Adaptation of Therapy Parameters for Unsupervised Robot-Assisted Rehabilitation: a Pilot Evaluation. *IEEE ... International Conference on Rehabilitation Robotics : [proceedings]*, *2022*, 1–6. <https://doi.org/10.1109/ICORR55369.2022.9896527>（The reason for exclusion: duplicate record）
224. Leonardis, D., Barsotti, M., Loconsole, C., Solazzi, M., Troncossi, M., Mazzotti, C., Castelli, V. P., Procopio, C., Lamola, G., Chisari, C., Bergamasco, M., & Frisoli, A. (2015). An EMG-Controlled Robotic Hand Exoskeleton for Bilateral Rehabilitation. *IEEE transactions on haptics*, *8*(2), 140–151. <https://doi.org/10.1109/TOH.2015.2417570>（The reason for exclusion: duplicate record）
225. Vahdat, S., Darainy, M., Thiel, A., & Ostry, D. J. (2019). A Single Session of Robot-Controlled Proprioceptive Training Modulates Functional Connectivity of Sensory Motor Networks and Improves Reaching Accuracy in Chronic Stroke. *Neurorehabilitation and neural repair*, *33*(1), 70–81. <https://doi.org/10.1177/1545968318818902>（The reason for exclusion: duplicate record）
226. Park J. H. (2021). The effects of robot-assisted left-hand training on hemispatial neglect in older patients with chronic stroke: A pilot and randomized controlled trial. *Medicine*, *100*(9), e24781. <https://doi.org/10.1097/MD.0000000000024781>（The reason for exclusion: duplicate record）
227. Pai, M. Y. B., Terranova, T. T., Simis, M., Fregni, F., & Battistella, L. R. (2018). The Combined Use of Transcranial Direct Current Stimulation and Robotic Therapy for the Upper Limb. *Journal of visualized experiments : JoVE*, (139), 58495. <https://doi.org/10.3791/58495>（The reason for exclusion: duplicate record）
228. Park, C., Oh-Park, M., Bialek, A., Friel, K., Edwards, D., & You, J. S. H. (2021). Abnormal synergistic gait mitigation in acute stroke using an innovative ankle-knee-hip interlimb humanoid robot: a preliminary randomized controlled trial. *Scientific reports*, *11*(1), 22823. <https://doi.org/10.1038/s41598-021-01959-z>（The reason for exclusion: duplicate record）
229. Daunoraviciene, K., Adomaviciene, A., Grigonyte, A., Griškevičius, J., & Juocevicius, A. (2018). Effects of robot-assisted training on upper limb functional recovery during the rehabilitation of poststroke patients. *Technology and health care : official journal of the European Society for Engineering and Medicine*, *26*(S2), 533–542. <https://doi.org/10.3233/THC-182500>（The reason for exclusion: duplicate record）
230. Camardella, C., Germanotta, M., Aprile, I., Cappiello, G., Curto, Z., Scoglio, A., Mazzoleni, S., & Frisoli, A. (2023). A Decision Support System to Provide an Ongoing Prediction of Robot-Assisted Rehabilitation Outcome in Stroke Survivors. *IEEE ... International Conference on Rehabilitation Robotics : [proceedings]*, *2023*, 1–6. <https://doi.org/10.1109/ICORR58425.2023.10304700>（The reason for exclusion: duplicate record）
231. Sloot, L. H., Baker, L. M., Bae, J., Porciuncula, F., Clément, B. F., Siviy, C., Nuckols, R. W., Baker, T., Sloutsky, R., Choe, D. K., O'Donnell, K., Ellis, T. D., Awad, L. N., & Walsh, C. J. (2023). Effects of a soft robotic exosuit on the quality and speed of overground walking depends on walking ability after stroke. *Journal of neuroengineering and rehabilitation*, *20*(1), 113. <https://doi.org/10.1186/s12984-023-01231-7>（The reason for exclusion: duplicate record）
232. Cho, K. H., & Song, W. K. (2019). Robot-Assisted Reach Training With an Active Assistant Protocol for Long-Term Upper Extremity Impairment Poststroke: A Randomized Controlled Trial. *Archives of physical medicine and rehabilitation*, *100*(2), 213–219. <https://doi.org/10.1016/j.apmr.2018.10.002>（The reason for exclusion: duplicate record）
233. Sloot, L., Bae, J., Baker, L., O'Donnell, K., Menard, N., Porciuncula, F., Choe, D., Ellis, T., Awad, L., & Walsh, C. (2022). O 089 - A soft robotic exosuit assisting the paretic ankle in patients post-stroke: Effect on muscle activation during overground walking. *Gait & posture*, *95*, 217–218. <https://doi.org/10.1016/j.gaitpost.2018.06.124>（The reason for exclusion: duplicate record）
234. Hyakutake, K., Morishita, T., Saita, K., Fukuda, H., Abe, H., Ogata, T., Kamada, S., & Inoue, T. (2022). Effect of Robot-assisted Rehabilitation to Botulinum Toxin A Injection for Upper Limb Disability in Patients with Chronic Stroke: A Case Series and Systematic Review. *Neurologia medico-chirurgica*, *62*(1), 35–44. <https://doi.org/10.2176/nmc.oa.2020-0408>（The reason for exclusion: duplicate record）
235. McCabe, J., Monkiewicz, M., Holcomb, J., Pundik, S., & Daly, J. J. (2015). Comparison of robotics, functional electrical stimulation, and motor learning methods for treatment of persistent upper extremity dysfunction after stroke: a randomized controlled trial. *Archives of physical medicine and rehabilitation*, *96*(6), 981–990. <https://doi.org/10.1016/j.apmr.2014.10.022>（The reason for exclusion: duplicate record）
236. Rodgers, H., Shaw, L., Bosomworth, H., Aird, L., Alvarado, N., Andole, S., Cohen, D. L., Dawson, J., Eyre, J., Finch, T., Ford, G. A., Hislop, J., Hogg, S., Howel, D., Hughes, N., Krebs, H. I., Price, C., Rochester, L., Stamp, E., Ternent, L., … Wilkes, S. (2017). Robot Assisted Training for the Upper Limb after Stroke (RATULS): study protocol for a randomised controlled trial. *Trials*, *18*(1), 340. <https://doi.org/10.1186/s13063-017-2083-4>（The reason for exclusion: duplicate record）
237. Hogan, N., Krebs, H. I., Rohrer, B., Palazzolo, J. J., Dipietro, L., Fasoli, S. E., Stein, J., Hughes, R., Frontera, W. R., Lynch, D., & Volpe, B. T. (2006). Motions or muscles? Some behavioral factors underlying robotic assistance of motor recovery. *Journal of rehabilitation research and development*, *43*(5), 605–618. <https://doi.org/10.1682/jrrd.2005.06.0103>（The reason for exclusion: duplicate record）
238. Calabrò, R. S., De Cola, M. C., Leo, A., Reitano, S., Balletta, T., Trombetta, G., Naro, A., Russo, M., Bertè, F., De Luca, R., & Bramanti, P. (2015). Robotic neurorehabilitation in patients with chronic stroke: psychological well-being beyond motor improvement. *International journal of rehabilitation research. Internationale Zeitschrift fur Rehabilitationsforschung. Revue internationale de recherches de readaptation*, *38*(3), 219–225. <https://doi.org/10.1097/MRR.0000000000000114>（The reason for exclusion: duplicate record）
239. Cai, S., Li, G., Su, E., Wei, X., Huang, S., Ma, K., Zheng, H., & Xie, L. (2020). Real-Time Detection of Compensatory Patterns in Patients With Stroke to Reduce Compensation During Robotic Rehabilitation Therapy. *IEEE journal of biomedical and health informatics*, *24*(9), 2630–2638. <https://doi.org/10.1109/JBHI.2019.2963365>（The reason for exclusion: duplicate record）
240. Hennes, M., Bollue, K., Arenbeck, H., & Disselhorst-Klug, C. (2015). A proposal for patient-tailored supervision of movement performance during end-effector-based robot-assisted rehabilitation of the upper extremities. *Biomedizinische Technik. Biomedical engineering*, *60*(3), 193–197. <https://doi.org/10.1515/bmt-2014-0021>（The reason for exclusion: duplicate record）
241. Kang, C. J., Chun, M. H., Lee, J., & Lee, J. Y. (2021). Effects of robot (SUBAR)-assisted gait training in patients with chronic stroke: Randomized controlled trial. *Medicine*, *100*(48), e27974. <https://doi.org/10.1097/MD.0000000000027974>（The reason for exclusion: duplicate record）
242. Maddalena, M., & Saadat, M. (2021). Simulated muscle activity in locomotion: implications of co-occurrence between effort minimisation and gait modularity for robot-assisted rehabilitation therapy. *Computer methods in biomechanics and biomedical engineering*, *24*(12), 1380–1392. <https://doi.org/10.1080/10255842.2021.1890046>（The reason for exclusion: duplicate record）
243. Seo, J. S., Yang, H. S., Jung, S., Kang, C. S., Jang, S., & Kim, D. H. (2018). Effect of reducing assistance during robot-assisted gait training on step length asymmetry in patients with hemiplegic stroke: A randomized controlled pilot trial. *Medicine*, *97*(33), e11792. <https://doi.org/10.1097/MD.0000000000011792>（The reason for exclusion: duplicate record）
244. Zhao, H., Cui, W., Wang, S., & Wang, L. (2023). Comment on: Efficacy of Robot-Assisted Training on Rehabilitation of Upper Limb Function in Patients With Stroke: A Systematic Review and Meta-analysis. *Archives of physical medicine and rehabilitation*, *104*(11), 1970–1971. https://doi.org/10.1016/j.apmr.2023.07.015（The reason for exclusion: review）
245. Jiang, S., You, H., Zhao, W., & Zhang, M. (2021). Effects of short-term upper limb robot-assisted therapy on the rehabilitation of sub-acute stroke patients. *Technology and health care : official journal of the European Society for Engineering and Medicine*, *29*(2), 295–303. <https://doi.org/10.3233/THC-202127>
246. Wagner, T. H., Lo, A. C., Peduzzi, P., Bravata, D. M., Huang, G. D., Krebs, H. I., Ringer, R. J., Federman, D. G., Richards, L. G., Haselkorn, J. K., Wittenberg, G. F., Volpe, B. T., Bever, C. T., Duncan, P. W., Siroka, A., & Guarino, P. D. (2011). An economic analysis of robot-assisted therapy for long-term upper-limb impairment after stroke. *Stroke*, *42*(9), 2630–2632. <https://doi.org/10.1161/STROKEAHA.110.606442>（The reason for exclusion: duplicate record
247. Song, R., Tong, K. Y., Hu, X., & Zhou, W. (2013). Myoelectrically controlled wrist robot for stroke rehabilitation. *Journal of neuroengineering and rehabilitation*, *10*, 52. <https://doi.org/10.1186/1743-0003-10-52>（The reason for exclusion: duplicate record
248. Secoli, R., Milot, M. H., Rosati, G., & Reinkensmeyer, D. J. (2011). Effect of visual distraction and auditory feedback on patient effort during robot-assisted movement training after stroke. *Journal of neuroengineering and rehabilitation*, *8*, 21. <https://doi.org/10.1186/1743-0003-8-21>（The reason for exclusion: duplicate record
249. Zhang, R., Feng, S., Hu, N., Low, S., Li, M., Chen, X., & Cui, H. (2024). Hybrid Brain-Computer Interface Controlled Soft Robotic Glove for Stroke Rehabilitation. *IEEE journal of biomedical and health informatics*, *28*(7), 4194–4203. <https://doi.org/10.1109/JBHI.2024.3392412>（The reason for exclusion: duplicate record
250. Shi, X. Q., Ti, C. E., Lu, H. Y., Hu, C. P., Xie, D. S., Yuan, K., Heung, H. L., Leung, T. W., Li, Z., & Tong, R. K. (2024). Task-Oriented Training by a Personalized Electromyography-Driven Soft Robotic Hand in Chronic Stroke: A Randomized Controlled Trial. *Neurorehabilitation and neural repair*, *38*(8), 595–606. <https://doi.org/10.1177/15459683241257519>（The reason for exclusion: duplicate record
251. Volpe, B. T., Krebs, H. I., Hogan, N., Edelstein OTR, L., Diels, C., & Aisen, M. (2000). A novel approach to stroke rehabilitation: robot-aided sensorimotor stimulation. *Neurology*, *54*(10), 1938–1944. <https://doi.org/10.1212/wnl.54.10.1938>（The reason for exclusion: duplicate record
252. Duret, C., Courtial, O., Grosmaire, A. G., & Hutin, E. (2015). Use of a robotic device for the rehabilitation of severe upper limb paresis in subacute stroke: exploration of patient/robot interactions and the motor recovery process. *BioMed research international*, *2015*, 482389. <https://doi.org/10.1155/2015/482389>（The reason for exclusion: Non-RCTs）
253. Colombo, R., Pisano, F., Delconte, C., Mazzone, A., Grioni, G., Castagna, M., Bazzini, G., Imarisio, C., Maggioni, G., & Pistarini, C. (2017). Comparison of exercise training effect with different robotic devices for upper limb rehabilitation: a retrospective study. *European journal of physical and rehabilitation medicine*, *53*(2), 240–248. https://doi.org/10.23736/S1973-9087.16.04297-0（The reason for exclusion: Non-RCTs）
254. Rowe, J. B., Chan, V., Ingemanson, M. L., Cramer, S. C., Wolbrecht, E. T., & Reinkensmeyer, D. J. (2017). Robotic Assistance for Training Finger Movement Using a Hebbian Model: A Randomized Controlled Trial. *Neurorehabilitation and neural repair*, *31*(8), 769–780. <https://doi.org/10.1177/1545968317721975>（The reason for exclusion: duplicate record）
255. Lo, A. C., Guarino, P. D., Richards, L. G., Haselkorn, J. K., Wittenberg, G. F., Federman, D. G., Ringer, R. J., Wagner, T. H., Krebs, H. I., Volpe, B. T., Bever, C. T., Jr, Bravata, D. M., Duncan, P. W., Corn, B. H., Maffucci, A. D., Nadeau, S. E., Conroy, S. S., Powell, J. M., Huang, G. D., & Peduzzi, P. (2010). Robot-assisted therapy for long-term upper-limb impairment after stroke. *The New England journal of medicine*, *362*(19), 1772–1783. <https://doi.org/10.1056/NEJMoa0911341>（The reason for exclusion: duplicate record）
256. Taheri, H., Rowe, J. B., Gardner, D., Chan, V., Reinkensmeyer, D. J., & Wolbrecht, E. T. (2012). Robot-assisted Guitar Hero for finger rehabilitation after stroke. *Annual International Conference of the IEEE Engineering in Medicine and Biology Society. IEEE Engineering in Medicine and Biology Society. Annual International Conference*, *2012*, 3911–3917. <https://doi.org/10.1109/EMBC.2012.6346822>（The reason for exclusion: meeting abstract）
257. Alingh, J. F., Fleerkotte, B. M., Groen, B. E., Rietman, J. S., Weerdesteyn, V., van Asseldonk, E. H. F., Geurts, A. C. H., & Buurke, J. H. (2021). Effect of assist-as-needed robotic gait training on the gait pattern post stroke: a randomized controlled trial. *Journal of neuroengineering and rehabilitation*, *18*(1), 26. <https://doi.org/10.1186/s12984-020-00800-4>（The reason for exclusion: duplicate record）
258. Zhou, H. X., Hu, J., Yun, R. S., Zhao, Z. Z., Lai, M. H., Sun, L. H., & Luo, K. L. (2023). Synergy-based functional electrical stimulation and robotic-assisted for retraining reach-to-grasp in stroke: a study protocol for a randomized controlled trial. *BMC neurology*, *23*(1), 324. <https://doi.org/10.1186/s12883-023-03369-2>（The reason for exclusion: duplicate record）
259. Yun, N., Joo, M. C., Kim, S. C., & Kim, M. S. (2018). Robot-assisted gait training effectively improved lateropulsion in subacute stroke patients: a single-blinded randomized controlled trial. *European journal of physical and rehabilitation medicine*, *54*(6), 827–836. <https://doi.org/10.23736/S1973-9087.18.05077-3>（The reason for exclusion: duplicate record）
260. Ii, T., Hirano, S., Tanabe, S., Saitoh, E., Yamada, J., Mukaino, M., Watanabe, M., Sonoda, S., & Otaka, Y. (2020). Robot-assisted Gait Training Using Welwalk in Hemiparetic Stroke Patients: An Effectiveness Study with Matched Control. *Journal of stroke and cerebrovascular diseases : the official journal of National Stroke Association*, *29*(12), 105377. <https://doi.org/10.1016/j.jstrokecerebrovasdis.2020.105377>（The reason for exclusion: duplicate record）
261. Leerskov, K. S., Rikhof, C. J. H., Spaich, E. G., Dosen, S., Prange-Lasonder, G. B., Prinsen, E. C., Rietman, J. S., & Struijk, L. N. S. A. (2024). A robot-based hybrid lower limb system for Assist-As-Needed rehabilitation of stroke patients: Technical evaluation and clinical feasibility. *Computers in biology and medicine*, *179*, 108839. <https://doi.org/10.1016/j.compbiomed.2024.108839>（The reason for exclusion: duplicate record）
262. Hu, X. L., Tong, K. Y., Wei, X. J., Rong, W., Susanto, E. A., & Ho, S. K. (2013). The effects of post-stroke upper-limb training with an electromyography (EMG)-driven hand robot. *Journal of electromyography and kinesiology : official journal of the International Society of Electrophysiological Kinesiology*, *23*(5), 1065–1074. https://doi.org/10.1016/j.jelekin.2013.07.007（The reason for exclusion: Non-RCTs）
263. Duret, C., Hutin, E., Lehenaff, L., & Gracies, J. M. (2015). Do all sub acute stroke patients benefit from robot-assisted therapy? A retrospective study. *Restorative neurology and neuroscience*, *33*(1), 57–65. <https://doi.org/10.3233/RNN-140418>（The reason for exclusion: Non robot-assisted task-oriented training）
264. Sale, P., Mazzoleni, S., Lombardi, V., Galafate, D., Massimiani, M. P., Posteraro, F., Damiani, C., & Franceschini, M. (2014). Recovery of hand function with robot-assisted therapy in acute stroke patients: a randomized-controlled trial. *International journal of rehabilitation research. Internationale Zeitschrift fur Rehabilitationsforschung. Revue internationale de recherches de readaptation*, *37*(3), 236–242. <https://doi.org/10.1097/MRR.0000000000000059>（The reason for exclusion: Non robot-assisted task-oriented training）
265. Hesse, S., Schulte-Tigges, G., Konrad, M., Bardeleben, A., & Werner, C. (2003). Robot-assisted arm trainer for the passive and active practice of bilateral forearm and wrist movements in hemiparetic subjects. *Archives of physical medicine and rehabilitation*, *84*(6), 915–920. <https://doi.org/10.1016/s0003-9993(02)04954-7>（The reason for exclusion: Non robot-assisted task-oriented training）
266. Jeon, S. Y., Ki, M., & Shin, J. H. (2024). Resistive versus active assisted robotic training for the upper limb after a stroke: A randomized controlled study. *Annals of physical and rehabilitation medicine*, *67*(1), 101789. <https://doi.org/10.1016/j.rehab.2023.101789>（The reason for exclusion: Non robot-assisted task-oriented training）
267. Gandolfi, M., Geroin, C., Tomelleri, C., Maddalena, I., Kirilova Dimitrova, E., Picelli, A., Smania, N., & Waldner, A. (2017). Feasibility and safety of early lower limb robot-assisted training in sub-acute stroke patients: a pilot study. *European journal of physical and rehabilitation medicine*, *53*(6), 870–882. <https://doi.org/10.23736/S1973-9087.17.04468-9>（The reason for exclusion: Non robot-assisted task-oriented training）
268. Lee, S. H., Lee, H. J., Shim, Y., Chang, W. H., Choi, B. O., Ryu, G. H., & Kim, Y. H. (2020). Wearable hip-assist robot modulates cortical activation during gait in stroke patients: a functional near-infrared spectroscopy study. *Journal of neuroengineering and rehabilitation*, *17*(1), 145. <https://doi.org/10.1186/s12984-020-00777-0>（The reason for exclusion: Non-RCTs）
269. Hesse, S., Heß, A., Werner C, C., Kabbert, N., & Buschfort, R. (2014). Effect on arm function and cost of robot-assisted group therapy in subacute patients with stroke and a moderately to severely affected arm: a randomized controlled trial. *Clinical rehabilitation*, *28*(7), 637–647. <https://doi.org/10.1177/0269215513516967>（The reason for exclusion: duplicate record）
270. Hsu, C. Y., Wu, C. M., Huang, C. C., Shie, H. H., & Tsai, Y. S. (2022). Feasibility and Potential Effects of Robot-Assisted Passive Range of Motion Training in Combination with Conventional Rehabilitation on Hand Function in Patients with Chronic Stroke. *Journal of rehabilitation medicine*, *54*, jrm00323. <https://doi.org/10.2340/jrm.v54.1407>（The reason for exclusion: duplicate record）
271. Nam, C., Zhang, B., Chow, T., Ye, F., Huang, Y., Guo, Z., Li, W., Rong, W., Hu, X., & Poon, W. (2021). Home-based self-help telerehabilitation of the upper limb assisted by an electromyography-driven wrist/hand exoneuromusculoskeleton after stroke. *Journal of neuroengineering and rehabilitation*, *18*(1), 137. <https://doi.org/10.1186/s12984-021-00930-3>（The reason for exclusion: duplicate record）
272. Lee, S. H., & Song, W. K. (2024). Mitigating Trunk Compensatory Movements in Post-Stroke Survivors through Visual Feedback during Robotic-Assisted Arm Reaching Exercises. *Sensors (Basel, Switzerland)*, *24*(11), 3331. <https://doi.org/10.3390/s24113331>（The reason for exclusion: duplicate record）
273. Finley, M. A., Fasoli, S. E., Dipietro, L., Ohlhoff, J., Macclellan, L., Meister, C., Whitall, J., Macko, R., Bever, C. T., Jr, Krebs, H. I., & Hogan, N. (2005). Short-duration robotic therapy in stroke patients with severe upper-limb motor impairment. *Journal of rehabilitation research and development*, *42*(5), 683–692. <https://doi.org/10.1682/jrrd.2004.12.0153>（The reason for exclusion: duplicate record）
274. Colombo, R., Pisano, F., Micera, S., Mazzone, A., Delconte, C., Carrozza, M. C., Dario, P., & Minuco, G. (2008). Assessing mechanisms of recovery during robot-aided neurorehabilitation of the upper limb. *Neurorehabilitation and neural repair*, *22*(1), 50–63. <https://doi.org/10.1177/1545968307303401>（The reason for exclusion: duplicate record）
275. Geroin, C., Picelli, A., Munari, D., Waldner, A., Tomelleri, C., & Smania, N. (2011). Combined transcranial direct current stimulation and robot-assisted gait training in patients with chronic stroke: a preliminary comparison. *Clinical rehabilitation*, *25*(6), 537–548. <https://doi.org/10.1177/0269215510389497>（The reason for exclusion: duplicate record）
276. Lutokhin, G. M., Kashezhev, A. G., Rassulova, M. A., Pogonchenkova, I. V., Turova, E. A., Shulkina, A. V., & Samokhvalov, R. I. (2022). Primenenie robotizirovannoi mekhanoterapii dlya vosstanovleniya dvizheniya u patsientov posle insul'ta [Implementation of robotic mechanotherapy for movement recovery in patients after stroke]. *Voprosy kurortologii, fizioterapii, i lechebnoi fizicheskoi kultury*, *99*(5), 60–67. <https://doi.org/10.17116/kurort20229905160>（The reason for exclusion: duplicate record）
277. Liao, W. W., Wu, C. Y., Hsieh, Y. W., Lin, K. C., & Chang, W. Y. (2012). Effects of robot-assisted upper limb rehabilitation on daily function and real-world arm activity in patients with chronic stroke: a randomized controlled trial. *Clinical rehabilitation*, *26*(2), 111–120. <https://doi.org/10.1177/0269215511416383>（The reason for exclusion: duplicate record）
278. Łajczak, P. M., Jóźwik, K., Nowakowski, P., & Nawrat, Z. (2024). Machine Meets Brain: A Systematic Review of Effectiveness of Robotically Performed Cerebral Angiography Interventions. *World neurosurgery*, *186*, 235–241.e1. <https://doi.org/10.1016/j.wneu.2024.04.012>（The reason for exclusion: duplicate record）
279. Astrakas, L. G., Naqvi, S. H., Kateb, B., & Tzika, A. A. (2012). Functional MRI using robotic MRI compatible devices for monitoring rehabilitation from chronic stroke in the molecular medicine era (Review). *International journal of molecular medicine*, *29*(6), 963–973. <https://doi.org/10.3892/ijmm.2012.942>（The reason for exclusion: review）
280. Bernhardt, J., & Mehrholz, J. (2019). Robotic-assisted training after stroke: RATULS advances science. *Lancet (London, England)*, *394*(10192), 6–8. <https://doi.org/10.1016/S0140-6736(19)31156-0>（The reason for exclusion: review）
281. Flynn, N., Froude, E., Cooke, D., Dennis, J., & Kuys, S. (2022). The sustainability of upper limb robotic therapy for stroke survivors in an inpatient rehabilitation setting. *Disability and rehabilitation*, *44*(24), 7522–7527. <https://doi.org/10.1080/09638288.2021.1998664>（The reason for exclusion: duplicate record）
282. Sharma, A., Kumari, S., & Saxena, A. (2023). Commentary on "Comparative Effectiveness of Robot-Assisted Training Versus Enhanced Upper Extremity Therapy on Upper and Lower Extremity for Stroke Survivors: A Multicentre Randomized Controlled Trial". *Journal of rehabilitation medicine*, *55*, jrm5588. <https://doi.org/10.2340/jrm.v55.5588>（The reason for exclusion: duplicate record）
283. Raghavan, P., Bilaloglu, S., Ali, S. Z., Jin, X., Aluru, V., Buckley, M. C., Tang, A., Yousefi, A., Stone, J., Agrawal, S. K., & Lu, Y. (2020). The Role of Robotic Path Assistance and Weight Support in Facilitating 3D Movements in Individuals With Poststroke Hemiparesis. *Neurorehabilitation and neural repair*, *34*(2), 134–147. <https://doi.org/10.1177/1545968319887685>（The reason for exclusion: duplicate record）
284. Masiero, S., Armani, M., & Rosati, G. (2011). Upper-limb robot-assisted therapy in rehabilitation of acute stroke patients: focused review and results of new randomized controlled trial. *Journal of rehabilitation research and development*, *48*(4), 355–366. <https://doi.org/10.1682/jrrd.2010.04.0063>（The reason for exclusion: duplicate record）
285. Sale, P., Infarinato, F., Del Percio, C., Lizio, R., Babiloni, C., Foti, C., & Franceschini, M. (2015). Electroencephalographic markers of robot-aided therapy in stroke patients for the evaluation of upper limb rehabilitation. *International journal of rehabilitation research. Internationale Zeitschrift fur Rehabilitationsforschung. Revue internationale de recherches de readaptation*, *38*(4), 294–305. <https://doi.org/10.1097/MRR.0000000000000125>（The reason for exclusion: duplicate record）
286. Mayr, A., Quirbach, E., Picelli, A., Kofler, M., Smania, N., & Saltuari, L. (2018). Early robot-assisted gait retraining in non-ambulatory patients with stroke: a single blind randomized controlled trial. *European journal of physical and rehabilitation medicine*, *54*(6), 819–826. <https://doi.org/10.23736/S1973-9087.18.04832-3>（The reason for exclusion: duplicate record）
287. Brockmann, L., Saengsuwan, J., Schuster-Amft, C., & Hunt, K. J. (2024). Feedback control of heart rate during robotics-assisted tilt table exercise in patients after stroke: a clinical feasibility study. *Journal of neuroengineering and rehabilitation*, *21*(1), 141. <https://doi.org/10.1186/s12984-024-01440-8>（The reason for exclusion: duplicate record）
288. Brokaw, E. B., Murray, T., Nef, T., & Lum, P. S. (2011). Retraining of interjoint arm coordination after stroke using robot-assisted time-independent functional training. *Journal of rehabilitation research and development*, *48*(4), 299–316. <https://doi.org/10.1682/jrrd.2010.04.0064>（The reason for exclusion: duplicate record）
289. Orihuela-Espina, F., Roldán, G. F., Sánchez-Villavicencio, I., Palafox, L., Leder, R., Sucar, L. E., & Hernández-Franco, J. (2016). Robot training for hand motor recovery in subacute stroke patients: A randomized controlled trial. *Journal of hand therapy : official journal of the American Society of Hand Therapists*, *29*(1), 51–57. <https://doi.org/10.1016/j.jht.2015.11.006>（The reason for exclusion: duplicate record）
290. Takahashi, C. D., Der-Yeghiaian, L., Le, V., Motiwala, R. R., & Cramer, S. C. (2008). Robot-based hand motor therapy after stroke. *Brain : a journal of neurology*, *131*(Pt 2), 425–437. <https://doi.org/10.1093/brain/awm311>（The reason for exclusion: duplicate record）
291. Tanaka, H., Nankaku, M., Nishikawa, T., Hosoe, T., Yonezawa, H., Mori, H., Kikuchi, T., Nishi, H., Takagi, Y., Miyamoto, S., Ikeguchi, R., & Matsuda, S. (2019). Spatiotemporal gait characteristic changes with gait training using the hybrid assistive limb for chronic stroke patients. *Gait & posture*, *71*, 205–210. <https://doi.org/10.1016/j.gaitpost.2019.05.003>（The reason for exclusion: duplicate record）
292. Taki, S., Imura, T., Iwamoto, Y., Imada, N., Tanaka, R., Araki, H., & Araki, O. (2020). Effects of Exoskeletal Lower Limb Robot Training on the Activities of Daily Living in Stroke Patients: Retrospective Pre-Post Comparison Using Propensity Score Matched Analysis. *Journal of stroke and cerebrovascular diseases : the official journal of National Stroke Association*, *29*(10), 105176. <https://doi.org/10.1016/j.jstrokecerebrovasdis.2020.105176>（The reason for exclusion: duplicate record）
293. Cazenave, L., Yurkewich, A., Hohler, C., Keller, T., Krewer, C., Jahn, K., Hirche, S., Endo, S., & Burdet, E. (2023). Hybrid Functional Electrical Stimulation and Robotic Assistance for Wrist Motion Training After Stroke: Preliminary Results. *IEEE ... International Conference on Rehabilitation Robotics : [proceedings]*, *2023*, 1–6. <https://doi.org/10.1109/ICORR58425.2023.10304736>（The reason for exclusion: duplicate record）
294. Vanoglio, F., Bernocchi, P., Mulè, C., Garofali, F., Mora, C., Taveggia, G., Scalvini, S., & Luisa, A. (2017). Feasibility and efficacy of a robotic device for hand rehabilitation in hemiplegic stroke patients: a randomized pilot controlled study. *Clinical rehabilitation*, *31*(3), 351–360. <https://doi.org/10.1177/0269215516642606>（The reason for exclusion: duplicate record）
295. Trujillo, P., Mastropietro, A., Scano, A., Chiavenna, A., Mrakic-Sposta, S., Caimmi, M., Molteni, F., & Rizzo, G. (2017). Quantitative EEG for Predicting Upper Limb Motor Recovery in Chronic Stroke Robot-Assisted Rehabilitation. *IEEE transactions on neural systems and rehabilitation engineering : a publication of the IEEE Engineering in Medicine and Biology Society*, *25*(7), 1058–1067. <https://doi.org/10.1109/TNSRE.2017.2678161>（The reason for exclusion: Non robot-assisted task-oriented training）
296. Huang, X., Naghdy, F., Naghdy, G., Du, H., & Todd, C. (2018). The Combined Effects of Adaptive Control and Virtual Reality on Robot-Assisted Fine Hand Motion Rehabilitation in Chronic Stroke Patients: A Case Study. *Journal of stroke and cerebrovascular diseases : the official journal of National Stroke Association*, *27*(1), 221–228. <https://doi.org/10.1016/j.jstrokecerebrovasdis.2017.08.027>（The reason for exclusion: Non robot-assisted task-oriented training）
297. Nankaku, M., Tanaka, H., Ikeguchi, R., Kikuchi, T., Miyamoto, S., & Matsuda, S. (2020). Effects of walking distance over robot-assisted training on walking ability in chronic stroke patients. *Journal of clinical neuroscience : official journal of the Neurosurgical Society of Australasia*, *81*, 279–283. <https://doi.org/10.1016/j.jocn.2020.09.067>（The reason for exclusion: duplicate record）
298. Christopher, S. M., & Johnson, M. J. (2014). Task-oriented robot-assisted stroke therapy of paretic limb improves control in a unilateral and bilateral functional drink task: a case study. *Annual International Conference of the IEEE Engineering in Medicine and Biology Society. IEEE Engineering in Medicine and Biology Society. Annual International Conference*, *2014*, 1194–1197. <https://doi.org/10.1109/EMBC.2014.6943810>（The reason for exclusion: Non robot-assisted task-oriented training）
299. Colombo, R., Pisano, F., Micera, S., Mazzone, A., Delconte, C., Carrozza, M. C., Dario, P., & Minuco, G. (2005). Robotic techniques for upper limb evaluation and rehabilitation of stroke patients. *IEEE transactions on neural systems and rehabilitation engineering : a publication of the IEEE Engineering in Medicine and Biology Society*, *13*(3), 311–324. <https://doi.org/10.1109/TNSRE.2005.848352>（The reason for exclusion: Non robot-assisted task-oriented training）
300. Noronha, B., Ng, C. Y., Little, K., Xiloyannis, M., Kuah, C. W. K., Wee, S. K., Kulkarni, S. R., Masia, L., Chua, K. S. G., & Accoto, D. (2022). Soft, Lightweight Wearable Robots to Support the Upper Limb in Activities of Daily Living: A Feasibility Study on Chronic Stroke Patients. *IEEE transactions on neural systems and rehabilitation engineering : a publication of the IEEE Engineering in Medicine and Biology Society*, *30*, 1401–1411. <https://doi.org/10.1109/TNSRE.2022.3175224>（The reason for exclusion: duplicate record）
301. Massie, C. L., Du, Y., Conroy, S. S., Krebs, H. I., Wittenberg, G. F., Bever, C. T., & Whitall, J. (2016). A Clinically Relevant Method of Analyzing Continuous Change in Robotic Upper Extremity Chronic Stroke Rehabilitation. *Neurorehabilitation and neural repair*, *30*(8), 703–712. <https://doi.org/10.1177/1545968315620301>（The reason for exclusion: Non robot-assisted task-oriented training）
302. Zhang, B., Wong, K. P., Kang, R., Fu, S., Qin, J., & Xiao, Q. (2023). Efficacy of Robot-Assisted and Virtual Reality Interventions on Balance, Gait, and Daily Function in Patients With Stroke: A Systematic Review and Network Meta-analysis. *Archives of physical medicine and rehabilitation*, *104*(10), 1711–1719. <https://doi.org/10.1016/j.apmr.2023.04.005>（The reason for exclusion: review）
303. Yang, X., Shi, X., Xue, X., & Deng, Z. (2023). Efficacy of Robot-Assisted Training on Rehabilitation of Upper Limb Function in Patients With Stroke: A Systematic Review and Meta-analysis. *Archives of physical medicine and rehabilitation*, *104*(9), 1498–1513. <https://doi.org/10.1016/j.apmr.2023.02.004>（The reason for exclusion: review）
304. Iwamoto, Y., Imura, T., Suzukawa, T., Fukuyama, H., Ishii, T., Taki, S., Imada, N., Shibukawa, M., Inagawa, T., Araki, H., & Araki, O. (2019). Combination of Exoskeletal Upper Limb Robot and Occupational Therapy Improve Activities of Daily Living Function in Acute Stroke Patients. *Journal of stroke and cerebrovascular diseases : the official journal of National Stroke Association*, *28*(7), 2018–2025. <https://doi.org/10.1016/j.jstrokecerebrovasdis.2019.03.006>（The reason for exclusion: duplicate record）
305. Akıncı, M., Burak, M., Yaşar, E., & Kılıç, R. T. (2023). The effects of Robot-assisted gait training and virtual reality on balance and gait in stroke survivors: A randomized controlled trial. *Gait & posture*, *103*, 215–222. <https://doi.org/10.1016/j.gaitpost.2023.05.013>（The reason for exclusion: duplicate record）
306. Wong, Y., Li, C. J., Ada, L., Zhang, T., Månum, G., & Langhammer, B. (2022). Upper Limb Training with a Dynamic Hand Orthosis in Early Subacute Stroke: A Pilot Randomized Trial. *Journal of rehabilitation medicine*, *54*, jrm00279. https://doi.org/10.2340/jrm.v54.2231（The reason for exclusion: Non robot-assisted task-oriented training）
307. Bian, L., Zhang, L., Huang, G., Song, D., Zheng, K., Xu, X., Dai, W., Ren, C., & Shen, Y. (2024). Effects of Priming Intermittent Theta Burst Stimulation With High-Definition tDCS on Upper Limb Function in Hemiparetic Patients With Stroke: A Randomized Controlled Study. *Neurorehabilitation and neural repair*, *38*(4), 268–278. https://doi.org/10.1177/15459683241233259（The reason for exclusion: Non robot-assisted task-oriented training）
308. Chen, Z. J., He, C., Guo, F., Xiong, C. H., & Huang, X. L. (2021). Exoskeleton-Assisted Anthropomorphic Movement Training (EAMT) for Poststroke Upper Limb Rehabilitation: A Pilot Randomized Controlled Trial. *Archives of physical medicine and rehabilitation*, *102*(11), 2074–2082. https://doi.org/10.1016/j.apmr.2021.06.001（The reason for exclusion: duplicate record）
309. Feng, T., Zhao, C., Dong, J., Xue, Z., Cai, F., Li, X., Hu, Z., & Xue, X. (2024). The effect of unaffected side resistance training on upper limb function reconstruction and prevention of sarcopenia in stroke patients: a randomized controlled trial. *Scientific reports*, *14*(1), 25330. https://doi.org/10.1038/s41598-024-76810-2（The reason for exclusion: duplicate record）
310. Wang, L., Wang, S., Zhang, S., Dou, Z., & Guo, T. (2023). Effectiveness and electrophysiological mechanisms of focal vibration on upper limb motor dysfunction in patients with subacute stroke: A randomized controlled trial. *Brain research*, *1809*, 148353. https://doi.org/10.1016/j.brainres.2023.148353（The reason for exclusion: duplicate record）
311. Akinci, M., Burak, M., Kasal, F. Z., Özaslan, E. A., Huri, M., & Kurtaran, Z. A. (2024). The Effects of Combined Virtual Reality Exercises and Robot Assisted Gait Training on Cognitive Functions, Daily Living Activities, and Quality of Life in High Functioning Individuals With Subacute Stroke. *Perceptual and motor skills*, *131*(3), 756–769. <https://doi.org/10.1177/00315125241235420>（The reason for exclusion: duplicate record）
312. Valero-Cuevas, F. J., Klamroth-Marganska, V., Winstein, C. J., & Riener, R. (2016). Robot-assisted and conventional therapies produce distinct rehabilitative trends in stroke survivors. *Journal of neuroengineering and rehabilitation*, *13*(1), 92. <https://doi.org/10.1186/s12984-016-0199-5>（The reason for exclusion: duplicate record）
313. Liang, S., Hong, Z. Q., Cai, Q., Gao, H. G., Ren, Y. J., Zheng, H. Q., Chen, X., & Hu, X. Q. (2024). Effects of robot-assisted gait training on motor performance of lower limb in poststroke survivors: a systematic review with meta-analysis. *European review for medical and pharmacological sciences*, *28*(3), 879–898. <https://doi.org/10.26355/eurrev_202402_35325>（The reason for exclusion: review）
314. Ghasemi, A., Sadedel, M., & Moghaddam, M. M. (2024). A wearable system to assist impaired-neck patients: Design and evaluation. *Proceedings of the Institution of Mechanical Engineers. Part H, Journal of engineering in medicine*, *238*(1), 63–77. <https://doi.org/10.1177/09544119231211362>（The reason for exclusion: duplicate record）
315. Cindy J H, R., Prange-Lasonder, G. B., Prinsen, E. C., Buurke, J. H., & Rietman, J. S. (2022). Detection thresholds for electrostimulation combined with robotic leg support in sub-acute stroke patients. *IEEE ... International Conference on Rehabilitation Robotics : [proceedings]*, *2022*, 1–5. <https://doi.org/10.1109/ICORR55369.2022.9896576>（The reason for exclusion: duplicate record）
316. Schicketmueller, A., Lamprecht, J., Hofmann, M., Sailer, M., & Rose, G. (2020). Gait Event Detection for Stroke Patients during Robot-Assisted Gait Training. *Sensors (Basel, Switzerland)*, *20*(12), 3399. <https://doi.org/10.3390/s20123399>（The reason for exclusion: duplicate record）
317. Villafañe JH, Taveggia G, Galeri S, et al. Efficacy of Short-Term Robot-Assisted Rehabilitation in Patients With Hand Paralysis After Stroke: A Randomized Clinical Trial. Hand (N Y). 2018;13(1):95-102. doi:10.1177/1558944717692096 （The reason for exclusion: duplicate record）
318. Du J, Wang S, Cheng Y, et al. Effects of Neuromuscular Electrical Stimulation Combined with Repetitive Transcranial Magnetic Stimulation on Upper Limb Motor Function Rehabilitation in Stroke Patients with Hemiplegia [retracted in: Comput Math Methods Med. 2023 Nov 1;2023:9767295. doi: 10.1155/2023/9767295]. Comput Math Methods Med. 2022;2022:9455428. Published 2022 Jan 4. doi:10.1155/2022/9455428 （The reason for exclusion: Non robot-assisted task-oriented training）
319. Huang YC, Chen PC, Tso HH, Yang YC, Ho TL, Leong CP. Effects of kinesio taping on hemiplegic hand in patients with upper limb post-stroke spasticity: a randomized controlled pilot study. Eur J Phys Rehabil Med. 2019;55(5):551-557. doi:10.23736/S1973-9087.19.05684-3 （The reason for exclusion: Non robot-assisted task-oriented training）
320. Imms, C., Wallen, M., Elliott, C., Hoare, B., Randall, M., Greaves, S., Adair, B., Bradshaw, E., Carter, R., Orsini, F., Shih, S. T., & Reddihough, D. (2016). Minimising impairment: Protocol for a multicentre randomised controlled trial of upper limb orthoses for children with cerebral palsy. *BMC pediatrics*, *16*, 70. https://doi.org/10.1186/s12887-016-0608-8（The reason for exclusion: Non robot-assisted task-oriented training）
321. Fu, J., Zeng, M., Shen, F., Cui, Y., Zhu, M., Gu, X., & Sun, Y. (2017). Effects of action observation therapy on upper extremity function, daily activities and motion evoked potential in cerebral infarction patients. *Medicine*, *96*(42), e8080. https://doi.org/10.1097/MD.0000000000008080（The reason for exclusion: Non robot-assisted task-oriented training）
322. Zhou, Y. X., Xia, Y., Huang, J., Wang, H. P., Bao, X. L., Bi, Z. Y., Chen, X. B., Gao, Y. J., Lü, X. Y., & Wang, Z. G. (2017). Electromyographic bridge for promoting the recovery of hand movements in subacute stroke patients: A randomized controlled trial. *Journal of rehabilitation medicine*, *49*(8), 629–636. https://doi.org/10.2340/16501977-2256（The reason for exclusion: Non robot-assisted task-oriented training）
323. Zhou, Y. X., Xia, Y., Huang, J., Wang, H. P., Bao, X. L., Bi, Z. Y., Chen, X. B., Gao, Y. J., Lü, X. Y., & Wang, Z. G. (2017). Electromyographic bridge for promoting the recovery of hand movements in subacute stroke patients: A randomized controlled trial. *Journal of rehabilitation medicine*, *49*(8), 629–636. https://doi.org/10.2340/16501977-2256（The reason for exclusion: Non robot-assisted task-oriented training）
324. Kirac-Unal, Z., Gencay-Can, A., Karaca-Umay, E., & Cakci, F. A. (2019). The effect of task-oriented electromyography-triggered electrical stimulation of the paretic wrist extensors on upper limb motor function early after stroke: a pilot randomized controlled trial. *International journal of rehabilitation research. Internationale Zeitschrift fur Rehabilitationsforschung. Revue internationale de recherches de readaptation*, *42*(1), 74–81. https://doi.org/10.1097/MRR.0000000000000333（The reason for exclusion: Irrelevant study outcome）
325. Chae, J., Harley, M. Y., Hisel, T. Z., Corrigan, C. M., Demchak, J. A., Wong, Y. T., & Fang, Z. P. (2009). Intramuscular electrical stimulation for upper limb recovery in chronic hemiparesis: an exploratory randomized clinical trial. *Neurorehabilitation and neural repair*, *23*(6), 569–578. https://doi.org/10.1177/1545968308328729（The reason for exclusion: Non robot-assisted task-oriented training）
326. Zhou, Y. X., Xia, Y., Huang, J., Wang, H. P., Bao, X. L., Bi, Z. Y., Chen, X. B., Gao, Y. J., Lü, X. Y., & Wang, Z. G. (2017). Electromyographic bridge for promoting the recovery of hand movements in subacute stroke patients: A randomized controlled trial. *Journal of rehabilitation medicine*, *49*(8), 629–636. https://doi.org/10.2340/16501977-2256（The reason for exclusion: Non robot-assisted task-oriented training）
327. Nijland, R., van Wegen, E., van der Krogt, H., Bakker, C., Buma, F., Klomp, A., van Kordelaar, J., Kwakkel, G., & EXPLICIT-stroke consortium (2013). Characterizing the protocol for early modified constraint-induced movement therapy in the EXPLICIT-stroke trial. *Physiotherapy research international : the journal for researchers and clinicians in physical therapy*, *18*(1), 1–15. https://doi.org/10.1002/pri.1521（The reason for exclusion: Non robot-assisted task-oriented training）
328. Kirac-Unal, Z., Gencay-Can, A., Karaca-Umay, E., & Cakci, F. A. (2019). The effect of task-oriented electromyography-triggered electrical stimulation of the paretic wrist extensors on upper limb motor function early after stroke: a pilot randomized controlled trial. *International journal of rehabilitation research. Internationale Zeitschrift fur Rehabilitationsforschung. Revue internationale de recherches de readaptation*, *42*(1), 74–81. https://doi.org/10.1097/MRR.0000000000000333（The reason for exclusion: Irrelevant study outcome）
329. Chiu, H. C., Ada, L., & Lee, H. M. (2014). Upper limb training using Wii Sports Resort for children with hemiplegic cerebral palsy: a randomized, single-blind trial. *Clinical rehabilitation*, *28*(10), 1015–1024. https://doi.org/10.1177/0269215514533709（The reason for exclusion: Non robot-assisted task-oriented training）
330. Chae, J., Harley, M. Y., Hisel, T. Z., Corrigan, C. M., Demchak, J. A., Wong, Y. T., & Fang, Z. P. (2009). Intramuscular electrical stimulation for upper limb recovery in chronic hemiparesis: an exploratory randomized clinical trial. *Neurorehabilitation and neural repair*, *23*(6), 569–578. https://doi.org/10.1177/1545968308328729（The reason for exclusion: Non robot-assisted task-oriented training）
331. Lannin, N. A., Horsley, S. A., Herbert, R., McCluskey, A., & Cusick, A. (2003). Splinting the hand in the functional position after brain impairment: a randomized, controlled trial. *Archives of physical medicine and rehabilitation*, *84*(2), 297–302. https://doi.org/10.1053/apmr.2003.50031（The reason for exclusion: Non robot-assisted task-oriented training）
332. Prange-Lasonder, G. B., Radder, B., Kottink, A. I. R., Melendez-Calderon, A., Buurke, J. H., & Rietman, J. S. (2017). Applying a soft-robotic glove as assistive device and training tool with games to support hand function after stroke: Preliminary results on feasibility and potential clinical impact. *IEEE ... International Conference on Rehabilitation Robotics : [proceedings]*, *2017*, 1401–1406. https://doi.org/10.1109/ICORR.2017.8009444（The reason for exclusion: duplicate record）
333. Bakker, C. D., Massa, M., Daffertshofer, A., Pasman, J. W., van Kuijk, A. A., Kwakkel, G., & Stegeman, D. F. (2019). The addition of the MEP amplitude of finger extension muscles to clinical predictors of hand function after stroke: A prospective cohort study. *Restorative neurology and neuroscience*, *37*(5), 445–456. https://doi.org/10.3233/RNN-180890（The reason for exclusion: Non robot-assisted task-oriented training）
334. Bonzano, L., Pedullà, L., Tacchino, A., Brichetto, G., Battaglia, M. A., Mancardi, G. L., & Bove, M. (2019). Upper limb motor training based on task-oriented exercises induces functional brain reorganization in patients with multiple sclerosis. *Neuroscience*, *410*, 150–159. https://doi.org/10.1016/j.neuroscience.2019.05.004（The reason for exclusion: duplicate record）
335. Chae, J., Bethoux, F., Bohine, T., Dobos, L., Davis, T., & Friedl, A. (1998). Neuromuscular stimulation for upper extremity motor and functional recovery in acute hemiplegia. *Stroke*, *29*(5), 975–979. https://doi.org/10.1161/01.str.29.5.975（The reason for exclusion: Non robot-assisted task-oriented training）
336. Zhu, J. M., Zhuang, R., He, J., Wang, X. X., Wang, H., & Zhu, H. Y. (2020). *Zhongguo zhen jiu = Chinese acupuncture & moxibustion*, *40*(7), 697–701. <https://doi.org/10.13703/j.0255-2930.20190531-k0005>（The reason for exclusion: Non robot-assisted task-oriented training）
337. Semrau, J. A., Herter, T. M., Scott, S. H., & Dukelow, S. P. (2015). Examining Differences in Patterns of Sensory and Motor Recovery After Stroke With Robotics. *Stroke*, *46*(12), 3459–3469. <https://doi.org/10.1161/STROKEAHA.115.010750>（The reason for exclusion: duplicate record）
338. Chen, Y. W., Chiang, W. C., Chang, C. L., Lo, S. M., & Wu, C. Y. (2022). Comparative effects of EMG-driven robot-assisted therapy versus task-oriented training on motor and daily function in patients with stroke: a randomized cross-over trial. *Journal of neuroengineering and rehabilitation*, *19*(1), 6. <https://doi.org/10.1186/s12984-021-00961-w>（The reason for exclusion: duplicate record）
339. Yurkewich, A., Hebert, D., Wang, R. H., & Mihailidis, A. (2019). Hand Extension Robot Orthosis (HERO) Glove: Development and Testing With Stroke Survivors With Severe Hand Impairment. *IEEE transactions on neural systems and rehabilitation engineering : a publication of the IEEE Engineering in Medicine and Biology Society*, *27*(5), 916–926. <https://doi.org/10.1109/TNSRE.2019.2910011>（The reason for exclusion: duplicate record）
340. Joo, M. C., Jung, K. M., Kim, J. H., Jung, Y. J., Chang, W. N., & Shin, H. J. (2022). Robot-Assisted Therapy Combined with Trunk Restraint in Acute Stroke Patients: A Randomized Controlled Study. *Journal of stroke and cerebrovascular diseases : the official journal of National Stroke Association*, *31*(5), 106330. <https://doi.org/10.1016/j.jstrokecerebrovasdis.2022.106330>（The reason for exclusion: duplicate record）
341. Yeung, L. F., Lau, C. C. Y., Lai, C. W. K., Soo, Y. O. Y., Chan, M. L., & Tong, R. K. Y. (2021). Effects of wearable ankle robotics for stair and over-ground training on sub-acute stroke: a randomized controlled trial. *Journal of neuroengineering and rehabilitation*, *18*(1), 19. <https://doi.org/10.1186/s12984-021-00814-6>（The reason for exclusion: duplicate record）
342. Thimabut, N., Yotnuengnit, P., Charoenlimprasert, J., Sillapachai, T., Hirano, S., Saitoh, E., & Piravej, K. (2022). Effects of the Robot-Assisted Gait Training Device Plus Physiotherapy in Improving Ambulatory Functions in Patients With Subacute Stroke With Hemiplegia: An Assessor-Blinded, Randomized Controlled Trial. *Archives of physical medicine and rehabilitation*, *103*(5), 843–850. <https://doi.org/10.1016/j.apmr.2022.01.146>（The reason for exclusion: duplicate record）
343. Bernal-Jiménez, J. J., Dileone, M., Mordillo-Mateos, L., Martín-Conty, J. L., Durantez-Fernández, C., Viñuela, A., Martín-Rodríguez, F., Lerin-Calvo, A., Alcántara-Porcuna, V., & Polonio-López, B. (2024). Combining Transcranial Direct Current Stimulation With Hand Robotic Rehabilitation in Chronic Stroke Patients: A Double-Blind Randomized Clinical Trial. *American journal of physical medicine & rehabilitation*, *103*(10), 875–882. <https://doi.org/10.1097/PHM.0000000000002446>（The reason for exclusion: duplicate record）
344. Proulx, C. E., Higgins, J., & Gagnon, D. H. (2023). Occupational therapists' evaluation of the perceived usability and utility of wearable soft robotic exoskeleton gloves for hand function rehabilitation following a stroke. *Disability and rehabilitation. Assistive technology*, *18*(6), 953–962. <https://doi.org/10.1080/17483107.2021.1938710>（The reason for exclusion: duplicate record）
345. Akıncı, M., Burak, M., Yaşar, E., & Kılıç, R. T. (2023). The effects of Robot-assisted gait training and virtual reality on balance and gait in stroke survivors: A randomized controlled trial. *Gait & posture*, *103*, 215–222. <https://doi.org/10.1016/j.gaitpost.2023.05.013>（The reason for exclusion: duplicate record）
346. Sale, P., Franceschini, M., Mazzoleni, S., Palma, E., Agosti, M., & Posteraro, F. (2014). Effects of upper limb robot-assisted therapy on motor recovery in subacute stroke patients. *Journal of neuroengineering and rehabilitation*, *11*, 104. <https://doi.org/10.1186/1743-0003-11-104>（The reason for exclusion: duplicate record）
347. Schrader, M., Sterr, A., Kettlitz, R., Wohlmeiner, A., Buschfort, R., Dohle, C., & Bamborschke, S. (2022). The effect of mirror therapy can be improved by simultaneous robotic assistance. *Restorative neurology and neuroscience*, *40*(3), 185–194. <https://doi.org/10.3233/RNN-221263>（The reason for exclusion: duplicate record）
348. Inoue, S., Otaka, Y., Kumagai, M., Sugasawa, M., Mori, N., & Kondo, K. (2022). Effects of Balance Exercise Assist Robot training for patients with hemiparetic stroke: a randomized controlled trial. *Journal of neuroengineering and rehabilitation*, *19*(1), 12. <https://doi.org/10.1186/s12984-022-00989-6>（The reason for exclusion: duplicate record）
349. Ranzani, R., Lambercy, O., Metzger, J. C., Califfi, A., Regazzi, S., Dinacci, D., Petrillo, C., Rossi, P., Conti, F. M., & Gassert, R. (2020). Neurocognitive robot-assisted rehabilitation of hand function: a randomized control trial on motor recovery in subacute stroke. *Journal of neuroengineering and rehabilitation*, *17*(1), 115. <https://doi.org/10.1186/s12984-020-00746-7>（The reason for exclusion: duplicate record）
350. Lee, H. C., Kuo, F. L., Lin, Y. N., Liou, T. H., Lin, J. C., & Huang, S. W. (2021). Effects of Robot-Assisted Rehabilitation on Hand Function of People With Stroke: A Randomized, Crossover-Controlled, Assessor-Blinded Study. *The American journal of occupational therapy : official publication of the American Occupational Therapy Association*, *75*(1), 7501205020p1–7501205020p11. <https://doi.org/10.5014/ajot.2021.038232>（The reason for exclusion: duplicate record）
351. Novak, D., & Riener, R. (2020). Sensor Fusion in Assistive and Rehabilitation Robotics. *Sensors (Basel, Switzerland)*, *20*(18), 5235. <https://doi.org/10.3390/s20185235>（The reason for exclusion: Non robot-assisted task-oriented training）
352. Kilbreath, S. L., Crosbie, J., Canning, C. G., & Lee, M. J. (2006). Inter-limb coordination in bimanual reach-to-grasp following stroke. *Disability and rehabilitation*, *28*(23), 1435–1443. （The reason for exclusion: Non robot-assisted task-oriented training）https://doi.org/10.1080/09638280600638307（The reason for exclusion: Non robot-assisted task-oriented training）
353. Hesse, S., Kuhlmann, H., Wilk, J., Tomelleri, C., & Kirker, S. G. (2008). A new electromechanical trainer for sensorimotor rehabilitation of paralysed fingers: a case series in chronic and acute stroke patients. *Journal of neuroengineering and rehabilitation*, *5*, 21. https://doi.org/10.1186/1743-0003-5-21（The reason for exclusion: duplicate record）
354. Carey, J. R., Kimberley, T. J., Lewis, S. M., Auerbach, E. J., Dorsey, L., Rundquist, P., & Ugurbil, K. (2002). Analysis of fMRI and finger tracking training in subjects with chronic stroke. *Brain : a journal of neurology*, *125*(Pt 4), 773–788. <https://doi.org/10.1093/brain/awf091>（The reason for exclusion: Non robot-assisted task-oriented training）
355. Muller, C. O., Metais, A., Boublay, N., Breuil, C., Daligault, S., Di Rienzo, F., Guillot, A., Collet, C., Krolak-Salmon, P., & Saimpont, A. (2024). Anodal transcranial direct current stimulation does not enhance the effects of motor imagery training of a sequential finger-tapping task in young adults. *Journal of sports sciences*, *42*(5), 392–403. <https://doi.org/10.1080/02640414.2024.2328418>（The reason for exclusion: Non robot-assisted task-oriented training）
356. Bhatt, E., Nagpal, A., Greer, K. H., Grunewald, T. K., Steele, J. L., Wiemiller, J. W., Lewis, S. M., & Carey, J. R. (2007). Effect of finger tracking combined with electrical stimulation on brain reorganization and hand function in subjects with stroke. *Experimental brain research*, *182*(4), 435–447. <https://doi.org/10.1007/s00221-007-1001-5>（The reason for exclusion: Non robot-assisted task-oriented training）
357. Fischer, H. C., Stubblefield, K., Kline, T., Luo, X., Kenyon, R. V., & Kamper, D. G. (2007). Hand rehabilitation following stroke: a pilot study of assisted finger extension training in a virtual environment. *Topics in stroke rehabilitation*, *14*(1), 1–12. https://doi.org/10.1310/tsr1401-1（The reason for exclusion: duplicate record）
358. Jahangir, A. W., Tan, H. J., Norlinah, M. I., Nafisah, W. Y., Ramesh, S., Hamidon, B. B., & Raymond, A. A. (2007). Intramuscular injection of botulinum toxin for the treatment of wrist and finger spasticity after stroke. *The Medical journal of Malaysia*, *62*(4), 319–322.（The reason for exclusion: Non robot-assisted task-oriented training）
359. Cauraugh, J., Light, K., Kim, S., Thigpen, M., & Behrman, A. (2000). Chronic motor dysfunction after stroke: recovering wrist and finger extension by electromyography-triggered neuromuscular stimulation. *Stroke*, *31*(6), 1360–1364. <https://doi.org/10.1161/01.str.31.6.1360>（The reason for exclusion: Non robot-assisted task-oriented training）
360. Kim, D. G., Cho, Y. W., Hong, J. H., Song, J. C., Chung, H. A., Bai, D. S., Lee, C. H., & Jang, S. H. (2008). Effect of constraint-induced movement therapy with modified opposition restriction orthosis in chronic hemiparetic patients with stroke. *NeuroRehabilitation*, *23*(3), 239–244.（The reason for exclusion: duplicate record）
361. Seniów, J., Bilik, M., Leśniak, M., Waldowski, K., Iwański, S., & Członkowska, A. (2012). Transcranial magnetic stimulation combined with physiotherapy in rehabilitation of poststroke hemiparesis: a randomized, double-blind, placebo-controlled study. *Neurorehabilitation and neural repair*, *26*(9), 1072–1079. <https://doi.org/10.1177/1545968312445635>（The reason for exclusion: duplicate record）
362. Thielbar, K. O., Lord, T. J., Fischer, H. C., Lazzaro, E. C., Barth, K. C., Stoykov, M. E., Triandafilou, K. M., & Kamper, D. G. (2014). Training finger individuation with a mechatronic-virtual reality system leads to improved fine motor control post-stroke. *Journal of neuroengineering and rehabilitation*, *11*, 171. <https://doi.org/10.1186/1743-0003-11-171>（The reason for exclusion: duplicate record）
363. Singer, B. J., Vallence, A. M., Cleary, S., Cooper, I., & Loftus, A. M. (2013). The effect of EMG triggered electrical stimulation plus task practice on arm function in chronic stroke patients with moderate-severe arm deficits. *Restorative neurology and neuroscience*, *31*(6), 681–691. <https://doi.org/10.3233/RNN-130319>（The reason for exclusion: duplicate record）
364. Bakker, C. D., Massa, M., Daffertshofer, A., Pasman, J. W., van Kuijk, A. A., Kwakkel, G., & Stegeman, D. F. (2019). The addition of the MEP amplitude of finger extension muscles to clinical predictors of hand function after stroke: A prospective cohort study. *Restorative neurology and neuroscience*, *37*(5), 445–456. https://doi.org/10.3233/RNN-180890（The reason for exclusion: Non robot-assisted task-oriented training）
365. Yıldızgören, M. T., Nakipoğlu Yüzer, G. F., Ekiz, T., & Özgirgin, N. (2014). Effects of neuromuscular electrical stimulation on the wrist and finger flexor spasticity and hand functions in cerebral palsy. *Pediatric neurology*, *51*(3), 360–364. https://doi.org/10.1016/j.pediatrneurol.2014.05.009（The reason for exclusion: Non robot-assisted task-oriented training）
366. Lin, C. H., Chou, L. W., Luo, H. J., Tsai, P. Y., Lieu, F. K., Chiang, S. L., & Sung, W. H. (2015). Effects of Computer-Aided Interlimb Force Coupling Training on Paretic Hand and Arm Motor Control following Chronic Stroke: A Randomized Controlled Trial. *PloS one*, *10*(7), e0131048. https://doi.org/10.1371/journal.pone.0131048（The reason for exclusion: duplicate record）
367. Kwon, T. G., Park, E., Kang, C., Chang, W. H., & Kim, Y. H. (2016). The effects of combined repetitive transcranial magnetic stimulation and transcranial direct current stimulation on motor function in patients with stroke. *Restorative neurology and neuroscience*, *34*(6), 915–923. https://doi.org/10.3233/RNN-160654（The reason for exclusion: duplicate record）
368. Fluet, G. G., Merians, A. S., Qiu, Q., Davidow, A., & Adamovich, S. V. (2014). Comparing integrated training of the hand and arm with isolated training of the same effectors in persons with stroke using haptically rendered virtual environments, a randomized clinical trial. *Journal of neuroengineering and rehabilitation*, *11*, 126. <https://doi.org/10.1186/1743-0003-11-126> （The reason for exclusion: duplicate record）
369. Hwang, C. H., Seong, J. W., & Son, D. S. (2012). Individual finger synchronized robot-assisted hand rehabilitation in subacute to chronic stroke: a prospective randomized clinical trial of efficacy. *Clinical rehabilitation*, *26*(8), 696–704. <https://doi.org/10.1177/0269215511431473> （The reason for exclusion: duplicate record）
370. Bai, Y. L., Hu, Y. S., Wu, Y., Zhu, Y. L., Zhang, B., Jiang, C. Y., Sun, L. M., & Fan, W. K. (2014). Long-term three-stage rehabilitation intervention alleviates spasticity of the elbows, fingers, and plantar flexors and improves activities of daily living in ischemic stroke patients: a randomized, controlled trial. *Neuroreport*, *25*(13), 998–1005. <https://doi.org/10.1097/WNR.0000000000000194> （The reason for exclusion: duplicate record）
371. Jackman, M., Novak, I., & Lannin, N. (2014). Effectiveness of functional hand splinting and the cognitive orientation to occupational performance (CO-OP) approach in children with cerebral palsy and brain injury: two randomised controlled trial protocols. *BMC neurology*, *14*, 144. https://doi.org/10.1186/1471-2377-14-144（The reason for exclusion: Non robot-assisted task-oriented training）
372. Choi, Y., Gordon, J., Park, H., & Schweighofer, N. (2011). Feasibility of the adaptive and automatic presentation of tasks (ADAPT) system for rehabilitation of upper extremity function post-stroke. *Journal of neuroengineering and rehabilitation*, *8*, 42. <https://doi.org/10.1186/1743-0003-8-42>（The reason for exclusion: duplicate record）
373. Thorsen, R., Cortesi, M., Jonsdottir, J., Carpinella, I., Morelli, D., Casiraghi, A., Puglia, M., Diverio, M., & Ferrarin, M. (2013). Myoelectrically driven functional electrical stimulation may increase motor recovery of upper limb in poststroke subjects: a randomized controlled pilot study. *Journal of rehabilitation research and development*, *50*(6), 785–794. <https://doi.org/10.1682/JRRD.2012.07.0123>（The reason for exclusion: duplicate record）
374. Iosa, M., Morone, G., Ragaglini, M. R., Fusco, A., & Paolucci, S. (2013). Motor strategies and bilateral transfer in sensorimotor learning of patients with subacute stroke and healthy subjects. A randomized controlled trial. *European journal of physical and rehabilitation medicine*, *49*(3), 291–299.（The reason for exclusion: duplicate record）
375. Wang, L., Wang, S., Zhang, S., Dou, Z., & Guo, T. (2023). Effectiveness and electrophysiological mechanisms of focal vibration on upper limb motor dysfunction in patients with subacute stroke: A randomized controlled trial. *Brain research*, *1809*, 148353. https://doi.org/10.1016/j.brainres.2023.148353（The reason for exclusion: duplicate record）
376. Hu, C., Ti, C. H. E., Yuan, K., Chen, C., Khan, A., Shi, X., Chu, W. C., & Tong, R. K. (2024). Effects of high-definition tDCS targeting individual motor hotspot with EMG-driven robotic hand training on upper extremity motor function: a pilot randomized controlled trial. *Journal of neuroengineering and rehabilitation*, *21*(1), 169. https://doi.org/10.1186/s12984-024-01468-w（The reason for exclusion: duplicate record）
377. Sentandreu-Mañó, T., Tomás, J. M., & Ricardo Salom Terrádez, J. (2021). A randomised clinical trial comparing 35 Hz versus 50 Hz frequency stimulation effects on hand motor recovery in older adults after stroke. *Scientific reports*, *11*(1), 9131. https://doi.org/10.1038/s41598-021-88607-8（The reason for exclusion: Non robot-assisted task-oriented training）
378. Pearse, J. E., Cadwgan, J. E., Wisher, V., Jesmont, C., Mason-Burton, P., Barry, M., Jona James, J., Kelly, S., & Basu, A. P. (2020). Feasibility Trial of Thumb Taping by Parents in Infants with Cerebral Palsy: Brief Report. *Developmental neurorehabilitation*, *23*(1), 50–58. https://doi.org/10.1080/17518423.2019.1566280（The reason for exclusion: Irrelevant study outcome）
379. Shi, X. Q., Ti, C. E., Lu, H. Y., Hu, C. P., Xie, D. S., Yuan, K., Heung, H. L., Leung, T. W., Li, Z., & Tong, R. K. (2024). Task-Oriented Training by a Personalized Electromyography-Driven Soft Robotic Hand in Chronic Stroke: A Randomized Controlled Trial. *Neurorehabilitation and neural repair*, *38*(8), 595–606. https://doi.org/10.1177/15459683241257519（The reason for exclusion: Control group using additional interventions)
380. Rowe, J. B., Chan, V., Ingemanson, M. L., Cramer, S. C., Wolbrecht, E. T., & Reinkensmeyer, D. J. (2017). Robotic Assistance for Training Finger Movement Using a Hebbian Model: A Randomized Controlled Trial. *Neurorehabilitation and neural repair*, *31*(8), 769–780. https://doi.org/10.1177/1545968317721975（The reason for exclusion: duplicate record）
381. Susanto, E. A., Tong, R. K., Ockenfeld, C., & Ho, N. S. (2015). Efficacy of robot-assisted fingers training in chronic stroke survivors: a pilot randomized-controlled trial. *Journal of neuroengineering and rehabilitation*, *12*, 42. https://doi.org/10.1186/s12984-015-0033-5（The reason for exclusion: Non robot-assisted task-oriented training）
382. Wang, L., Zhu, Q. X., Zhong, M. H., Zhou, R. Z., Liu, X. Q., Tang, N. S., Feng, X. C., & Gao, C. F. (2022). Effects of corticospinal tract integrity on upper limb motor function recovery in stroke patients treated with repetitive transcranial magnetic stimulation. *Journal of integrative neuroscience*, *21*(2), 50. https://doi.org/10.31083/j.jin2102050（The reason for exclusion: Non robot-assisted task-oriented training）
383. Brunner, I., Skouen, J. S., Hofstad, H., Aßmus, J., Becker, F., Sanders, A. M., Pallesen, H., Qvist Kristensen, L., Michielsen, M., Thijs, L., & Verheyden, G. (2017). Virtual Reality Training for Upper Extremity in Subacute Stroke (VIRTUES): A multicenter RCT. *Neurology*, *89*(24), 2413–2421. https://doi.org/10.1212/WNL.0000000000004744（The reason for exclusion: Non robot-assisted task-oriented training）
384. Hermann-Eriksen, M., Nilsen, T., Hove, Å., Eilertsen, L., Haugen, I. K., Sexton, J., & Kjeken, I. (2022). Comparison of 2 Postoperative Therapy Regimens After Trapeziectomy Due to Osteoarthritis: A Randomized, Controlled Trial. *The Journal of hand surgery*, *47*(2), 120–129.e4. <https://doi.org/10.1016/j.jhsa.2021.08.015> （The reason for exclusion: Non robot-assisted task-oriented training）
385. Pearse, J. E., Cadwgan, J. E., Wisher, V., Jesmont, C., Mason-Burton, P., Barry, M., Jona James, J., Kelly, S., & Basu, A. P. (2020). Feasibility Trial of Thumb Taping by Parents in Infants with Cerebral Palsy: Brief Report. *Developmental neurorehabilitation*, *23*(1), 50–58. <https://doi.org/10.1080/17518423.2019.1566280> （The reason for exclusion: Non robot-assisted task-oriented training）
386. Rowe, J. B., Chan, V., Ingemanson, M. L., Cramer, S. C., Wolbrecht, E. T., & Reinkensmeyer, D. J. (2017). Robotic Assistance for Training Finger Movement Using a Hebbian Model: A Randomized Controlled Trial. *Neurorehabilitation and neural repair*, *31*(8), 769–780. <https://doi.org/10.1177/1545968317721975> （The reason for exclusion: Control group using additional interventions)
387. Cantero-Téllez, R., Pérez-Cruzado, D., Villafañe, J. H., García-Orza, S., Naughton, N., & Valdes, K. (2022). The Effect of Proprioception Training on Pain Intensity in Thumb Basal Joint Osteoarthritis: A Randomized Controlled Trial. *International journal of environmental research and public health*, *19*(6), 3592. <https://doi.org/10.3390/ijerph19063592> （The reason for exclusion: Non robot-assisted task-oriented training）
388. Susanto, E. A., Tong, R. K., Ockenfeld, C., & Ho, N. S. (2015). Efficacy of robot-assisted fingers training in chronic stroke survivors: a pilot randomized-controlled trial. *Journal of neuroengineering and rehabilitation*, *12*, 42. <https://doi.org/10.1186/s12984-015-0033-5> （The reason for exclusion: Non robot-assisted task-oriented training）
389. Wang, L., Zhu, Q. X., Zhong, M. H., Zhou, R. Z., Liu, X. Q., Tang, N. S., Feng, X. C., & Gao, C. F. (2022). Effects of corticospinal tract integrity on upper limb motor function recovery in stroke patients treated with repetitive transcranial magnetic stimulation. *Journal of integrative neuroscience*, *21*(2), 50. （The reason for exclusion: Non robot-assisted task-oriented training）<https://doi.org/10.31083/j.jin2102050> （The reason for exclusion: Non robot-assisted task-oriented training）
390. Eyiis, E., Mathijssen, N. M. C., Kok, P., Sluijter, J., & Kraan, G. A. (2023). Three-dimensional printed customized versus conventional plaster brace for trapeziometacarpal osteoarthritis: a randomized controlled crossover trial. *The Journal of hand surgery, European volume*, *48*(5), 412–418. <https://doi.org/10.1177/17531934221146864> （The reason for exclusion: Non robot-assisted task-oriented training）
391. Brunner, I., Skouen, J. S., Hofstad, H., Aßmus, J., Becker, F., Sanders, A. M., Pallesen, H., Qvist Kristensen, L., Michielsen, M., Thijs, L., & Verheyden, G. (2017). Virtual Reality Training for Upper Extremity in Subacute Stroke (VIRTUES): A multicenter RCT. *Neurology*, *89*(24), 2413–2421. <https://doi.org/10.1212/WNL.0000000000004744> （The reason for exclusion: Non robot-assisted task-oriented training）
392. Tramontano, M., Morone, G., De Angelis, S., Casagrande Conti, L., Galeoto, G., & Grasso, M. G. (2020). Sensor-based technology for upper limb rehabilitation in patients with multiple sclerosis: A randomized controlled trial. *Restorative neurology and neuroscience*, *38*(4), 333–341. <https://doi.org/10.3233/RNN-201033> （The reason for exclusion: Irrelevant study outcome）
393. Ji, E. K., Wang, H. H., Jung, S. J., Lee, K. B., Kim, J. S., Jo, L., Hong, B. Y., & Lim, S. H. (2021). Graded motor imagery training as a home exercise program for upper limb motor function in patients with chronic stroke: A randomized controlled trial. *Medicine*, *100*(3), e24351. <https://doi.org/10.1097/MD.0000000000024351>（The reason for exclusion: duplicate record）
394. Lee, H. C., Kuo, F. L., Lin, Y. N., Liou, T. H., Lin, J. C., & Huang, S. W. (2021). Effects of Robot-Assisted Rehabilitation on Hand Function of People With Stroke: A Randomized, Crossover-Controlled, Assessor-Blinded Study. *The American journal of occupational therapy : official publication of the American Occupational Therapy Association*, *75*(1), 7501205020p1–7501205020p11. <https://doi.org/10.5014/ajot.2021.038232>（The reason for exclusion: duplicate record）
395. Villafañe, J. H., Taveggia, G., Galeri, S., Bissolotti, L., Mullè, C., Imperio, G., Valdes, K., Borboni, A., & Negrini, S. (2018). Efficacy of Short-Term Robot-Assisted Rehabilitation in Patients With Hand Paralysis After Stroke: A Randomized Clinical Trial. *Hand (New York, N.Y.)*, *13*(1), 95–102. <https://doi.org/10.1177/1558944717692096>（The reason for exclusion: duplicate record）
396. Huang, Y. C., Chen, P. C., Tso, H. H., Yang, Y. C., Ho, T. L., & Leong, C. P. (2019). Effects of kinesio taping on hemiplegic hand in patients with upper limb post-stroke spasticity: a randomized controlled pilot study. *European journal of physical and rehabilitation medicine*, *55*(5), 551–557. （The reason for exclusion: duplicate record）<https://doi.org/10.23736/S1973-9087.19.05684-3>（The reason for exclusion: duplicate record）
397. Aguilera-Rubio, Á., Alguacil-Diego, I. M., Mallo-López, A., Jardón Huete, A., Oña, E. D., & Cuesta-Gómez, A. (2024). Use of low-cost virtual reality in the treatment of the upper extremity in chronic stroke: a randomized clinical trial. *Journal of neuroengineering and rehabilitation*, *21*(1), 12. <https://doi.org/10.1186/s12984-024-01303-2>（The reason for exclusion: duplicate record）
398. Tang, C., Zhou, T., Zhang, Y., Yuan, R., Zhao, X., Yin, R., Song, P., Liu, B., Song, R., Chen, W., & Wang, H. (2023). Bilateral upper limb robot-assisted rehabilitation improves upper limb motor function in stroke patients: a study based on quantitative EEG. *European journal of medical research*, *28*(1), 603. <https://doi.org/10.1186/s40001-023-01565-x>（The reason for exclusion: duplicate record）
399. Xia, X., Dong, X., Huo, H., Zhang, Y., Song, J., & Wang, D. (2023). Clinical study of low-frequency acupoint electrical stimulation to improve thumb-to-finger movements after stroke: A randomized controlled trial. *Medicine*, *102*(47), e35755. https://doi.org/10.1097/MD.0000000000035755（The reason for exclusion: Irrelevant study outcome）
400. Vanoglio, F., Comini, L., Gaiani, M., Bonometti, G. P., Luisa, A., & Bernocchi, P. (2024). A Sensor-Based Upper Limb Treatment in Hemiplegic Patients: Results from a Randomized Pilot Study. *Sensors (Basel, Switzerland)*, *24*(8), 2574. https://doi.org/10.3390/s24082574（The reason for exclusion: duplicate record）
401. Akgün, İ., Demirbüken, İ., Timurtaş, E., Pehlivan, M. K., Pehlivan, A. U., Polat, M. G., Francisco, G. E., & Yozbatiran, N. (2024). Exoskeleton-assisted upper limb rehabilitation after stroke: a randomized controlled trial. *Neurological research*, *46*(11), 1074–1082. https://doi.org/10.1080/01616412.2024.2381385（The reason for exclusion: duplicate record）
402. Bernal-Jiménez, J. J., Dileone, M., Mordillo-Mateos, L., Martín-Conty, J. L., Durantez-Fernández, C., Viñuela, A., Martín-Rodríguez, F., Lerin-Calvo, A., Alcántara-Porcuna, V., & Polonio-López, B. (2024). Combining Transcranial Direct Current Stimulation With Hand Robotic Rehabilitation in Chronic Stroke Patients: A Double-Blind Randomized Clinical Trial. *American journal of physical medicine & rehabilitation*, *103*(10), 875–882. https://doi.org/10.1097/PHM.0000000000002446（The reason for exclusion: duplicate record）
403. Li, Y., Lian, Y., Chen, X., Zhang, H., Xu, G., Duan, H., Xie, X., & Li, Z. (2024). Effect of task-oriented training assisted by force feedback hand rehabilitation robot on finger grasping function in stroke patients with hemiplegia: a randomised controlled trial. *Journal of neuroengineering and rehabilitation*, *21*(1), 77. https://doi.org/10.1186/s12984-024-01372-3（The reason for exclusion: duplicate record）
404. Calabrò, R. S., Accorinti, M., Porcari, B., Carioti, L., Ciatto, L., Billeri, L., Andronaco, V. A., Galletti, F., Filoni, S., & Naro, A. (2019). Does hand robotic rehabilitation improve motor function by rebalancing interhemispheric connectivity after chronic stroke? Encouraging data from a randomised-clinical-trial. *Clinical neurophysiology : official journal of the International Federation of Clinical Neurophysiology*, *130*(5), 767–780. https://doi.org/10.1016/j.clinph.2019.02.013（The reason for exclusion: duplicate record）
405. Kwakkel, G., Winters, C., van Wegen, E. E., Nijland, R. H., van Kuijk, A. A., Visser-Meily, A., de Groot, J., de Vlugt, E., Arendzen, J. H., Geurts, A. C., Meskers, C. G., & EXPLICIT-Stroke Consortium (2016). Effects of Unilateral Upper Limb Training in Two Distinct Prognostic Groups Early After Stroke: The EXPLICIT-Stroke Randomized Clinical Trial. *Neurorehabilitation and neural repair*, *30*(9), 804–816. https://doi.org/10.1177/1545968315624784（The reason for exclusion: Non robot-assisted task-oriented training）
406. Giray, E., Gencer Atalay, K., Eren, N., Gündüz, O. H., & Karadag-Saygi, E. (2020). Effects of dynamic lycra orthosis as an adjunct to rehabilitation after botulinum toxin-A injection of the upper-limb in adults following stroke: A single-blinded randomized controlled pilot study. *Topics in stroke rehabilitation*, *27*(6), 473–481. https://doi.org/10.1080/10749357.2019.1704371（The reason for exclusion: Control group using additional interventions)

Sakzewski, L., Ziviani, J., Abbott, D. F., Macdonell, R. A., Jackson, G. D., & Boyd, R. N. (2011). Randomized trial of constraint-induced movement therapy and bimanual training on activity outcomes for children with congenital hemiplegia. *Developmental medicine and child neurology*, *53*(4), 313–320. https://doi.org/10.1111/j.1469-8749.2010.03859.x（The reason for exclusion: Irrelevant study outcome）

1. Lannin, N. A., Cusick, A., Hills, C., Kinnear, B., Vogel, K., Matthews, K., & Bowring, G. (2016). Upper limb motor training using a Saebo™ orthosis is feasible for increasing task-specific practice in hospital after stroke. *Australian occupational therapy journal*, *63*(6), 364–372. https://doi.org/10.1111/1440-1630.12330（The reason for exclusion: Non robot-assisted task-oriented training）
2. Bang, D. H., Shin, W. S., & Choi, H. S. (2018). Effects of modified constraint-induced movement therapy with trunk restraint in early stroke patients: A single-blinded, randomized, controlled, pilot trial. *NeuroRehabilitation*, *42*(1), 29–35. https://doi.org/10.3233/NRE-172176（The reason for exclusion: Control group using additional interventions)
3. van Delden, A. L., Peper, C. L., Nienhuys, K. N., Zijp, N. I., Beek, P. J., & Kwakkel, G. (2013). Unilateral versus bilateral upper limb training after stroke: the Upper Limb Training After Stroke clinical trial. *Stroke*, *44*(9), 2613–2616. https://doi.org/10.1161/STROKEAHA.113.001969（The reason for exclusion: Irrelevant study outcome）
4. Khallaf, M. E., Ameer, M. A., & Fayed, E. E. (2017). Effect of task specific training and wrist-fingers extension splint on hand joints range of motion and function after stroke. *NeuroRehabilitation*, *41*(2), 437–444. https://doi.org/10.3233/NRE-162128（The reason for exclusion: Non robot-assisted task-oriented training）
5. Imms, C., Wallen, M., Elliott, C., Hoare, B., Randall, M., Greaves, S., Adair, B., Bradshaw, E., Carter, R., Orsini, F., Shih, S. T., & Reddihough, D. (2016). Minimising impairment: Protocol for a multicentre randomised controlled trial of upper limb orthoses for children with cerebral palsy. *BMC pediatrics*, *16*, 70. https://doi.org/10.1186/s12887-016-0608-8（The reason for exclusion: duplicate record）
6. Fu, J., Zeng, M., Shen, F., Cui, Y., Zhu, M., Gu, X., & Sun, Y. (2017). Effects of action observation therapy on upper extremity function, daily activities and motion evoked potential in cerebral infarction patients. *Medicine*, *96*(42), e8080. https://doi.org/10.1097/MD.0000000000008080（The reason for exclusion: Non robot-assisted task-oriented training）
7. Zhou, Y. X., Xia, Y., Huang, J., Wang, H. P., Bao, X. L., Bi, Z. Y., Chen, X. B., Gao, Y. J., Lü, X. Y., & Wang, Z. G. (2017). Electromyographic bridge for promoting the recovery of hand movements in subacute stroke patients: A randomized controlled trial. *Journal of rehabilitation medicine*, *49*(8), 629–636. https://doi.org/10.2340/16501977-2256（The reason for exclusion: Non robot-assisted task-oriented training）
8. Nijland, R., van Wegen, E., van der Krogt, H., Bakker, C., Buma, F., Klomp, A., van Kordelaar, J., Kwakkel, G., & EXPLICIT-stroke consortium (2013). Characterizing the protocol for early modified constraint-induced movement therapy in the EXPLICIT-stroke trial. *Physiotherapy research international : the journal for researchers and clinicians in physical therapy*, *18*(1), 1–15. https://doi.org/10.1002/pri.1521（The reason for exclusion: Non robot-assisted task-oriented training）
9. Kirac-Unal, Z., Gencay-Can, A., Karaca-Umay, E., & Cakci, F. A. (2019). The effect of task-oriented electromyography-triggered electrical stimulation of the paretic wrist extensors on upper limb motor function early after stroke: a pilot randomized controlled trial. *International journal of rehabilitation research. Internationale Zeitschrift fur Rehabilitationsforschung. Revue internationale de recherches de readaptation*, *42*(1), 74–81. https://doi.org/10.1097/MRR.0000000000000333（The reason for exclusion: Irrelevant study outcome）
10. Chiu, H. C., Ada, L., & Lee, H. M. (2014). Upper limb training using Wii Sports Resort for children with hemiplegic cerebral palsy: a randomized, single-blind trial. *Clinical rehabilitation*, *28*(10), 1015–1024. https://doi.org/10.1177/0269215514533709（The reason for exclusion: Non robot-assisted task-oriented training）
11. Chae, J., Harley, M. Y., Hisel, T. Z., Corrigan, C. M., Demchak, J. A., Wong, Y. T., & Fang, Z. P. (2009). Intramuscular electrical stimulation for upper limb recovery in chronic hemiparesis: an exploratory randomized clinical trial. *Neurorehabilitation and neural repair*, *23*(6), 569–578. https://doi.org/10.1177/1545968308328729（The reason for exclusion: Non robot-assisted task-oriented training）
12. Lannin, N. A., Horsley, S. A., Herbert, R., McCluskey, A., & Cusick, A. (2003). Splinting the hand in the functional position after brain impairment: a randomized, controlled trial. *Archives of physical medicine and rehabilitation*, *84*(2), 297–302. https://doi.org/10.1053/apmr.2003.50031（The reason for exclusion: Non robot-assisted task-oriented training）
13. Prange-Lasonder, G. B., Radder, B., Kottink, A. I. R., Melendez-Calderon, A., Buurke, J. H., & Rietman, J. S. (2017). Applying a soft-robotic glove as assistive device and training tool with games to support hand function after stroke: Preliminary results on feasibility and potential clinical impact. *IEEE ... International Conference on Rehabilitation Robotics : [proceedings]*, *2017*, 1401–1406. https://doi.org/10.1109/ICORR.2017.8009444（The reason for exclusion: duplicate record）
14. Inoue, S., Otaka, Y., Kumagai, M., Sugasawa, M., Mori, N., & Kondo, K. (2022). Effects of Balance Exercise Assist Robot training for patients with hemiparetic stroke: a randomized controlled trial. *Journal of neuroengineering and rehabilitation*, *19*(1), 12. <https://doi.org/10.1186/s12984-022-00989-6>（The reason for exclusion: duplicate record）
15. Ranzani, R., Lambercy, O., Metzger, J. C., Califfi, A., Regazzi, S., Dinacci, D., Petrillo, C., Rossi, P., Conti, F. M., & Gassert, R. (2020). Neurocognitive robot-assisted rehabilitation of hand function: a randomized control trial on motor recovery in subacute stroke. *Journal of neuroengineering and rehabilitation*, *17*(1), 115. <https://doi.org/10.1186/s12984-020-00746-7>（The reason for exclusion: duplicate record）
16. Lee, H. C., Kuo, F. L., Lin, Y. N., Liou, T. H., Lin, J. C., & Huang, S. W. (2021). Effects of Robot-Assisted Rehabilitation on Hand Function of People With Stroke: A Randomized, Crossover-Controlled, Assessor-Blinded Study. *The American journal of occupational therapy : official publication of the American Occupational Therapy Association*, *75*(1), 7501205020p1–7501205020p11. <https://doi.org/10.5014/ajot.2021.038232>（The reason for exclusion: duplicate record）
17. Novak, D., & Riener, R. (2020). Sensor Fusion in Assistive and Rehabilitation Robotics. *Sensors (Basel, Switzerland)*, *20*(18), 5235. <https://doi.org/10.3390/s20185235>（The reason for exclusion: Non robot-assisted task-oriented training）
18. Dehem, S., Gilliaux, M., Stoquart, G., Detrembleur, C., Jacquemin, G., Palumbo, S., Frederick, A., & Lejeune, T. (2019). Effectiveness of upper-limb robotic-assisted therapy in the early rehabilitation phase after stroke: A single-blind, randomised, controlled trial. *Annals of physical and rehabilitation medicine*, *62*(5), 313–320. <https://doi.org/10.1016/j.rehab.2019.04.002>（The reason for exclusion: duplicate record）
19. Castelli E. (2023). Robotic Rehabilitation in Children. *Psychiatria Danubina*, *35*(Suppl 3), 93–94.（The reason for exclusion: Non robot-assisted task-oriented training）
20. Wang, J., Li, Y., Qi, L., Mamtilahun, M., Liu, C., Liu, Z., Shi, R., Wu, S., & Yang, G. Y. (2024). Advanced rehabilitation in ischaemic stroke research. *Stroke and vascular neurology*, *9*(4), 328–343. https://doi.org/10.1136/svn-2022-002285（The reason for exclusion: review）
21. Pignolo L. (2009). Robotics in neuro-rehabilitation. *Journal of rehabilitation medicine*, *41*(12), 955–960. https://doi.org/10.2340/16501977-0434（The reason for exclusion: Non-RCTs）
22. Bui, K. D., & Johnson, M. J. (2018). Designing robot-assisted neurorehabilitation strategies for people with both HIV and stroke. *Journal of neuroengineering and rehabilitation*, *15*(1), 75. https://doi.org/10.1186/s12984-018-0418-3（The reason for exclusion: review）
23. Carrillo, C., Tilley, D., Horn, K., Gonzalez, M., Coffman, C., Hilton, C., & Mani, K. (2023). Effectiveness of Robotics in Stroke Rehabilitation to Accelerate Upper Extremity Function: Systematic Review. *Occupational therapy international*, *2023*, 7991765. https://doi.org/10.1155/2023/7991765（The reason for exclusion: review）
24. Cinnera, A. M., Bonnì, S., D'Acunto, A., Maiella, M., Ferraresi, M., Casula, E. P., Pezzopane, V., Tramontano, M., Iosa, M., Paolucci, S., Morone, G., Vannozzi, G., & Koch, G. (2023). Cortico-cortical stimulation and robot-assisted therapy (CCS and RAT) for upper limb recovery after stroke: study protocol for a randomised controlled trial. *Trials*, *24*(1), 823. https://doi.org/10.1186/s13063-023-07849-1（The reason for exclusion: Control group using additional interventions)
25. Kim, E., Lee, G., Lee, J., & Kim, Y. H. (2024). Simultaneous high-definition transcranial direct current stimulation and robot-assisted gait training in stroke patients. *Scientific reports*, *14*(1), 4483. https://doi.org/10.1038/s41598-024-53482-6（The reason for exclusion: Control group using additional interventions)
26. Stockbridge, M. D., Bunker, L. D., & Hillis, A. E. (2022). Reversing the Ruin: Rehabilitation, Recovery, and Restoration After Stroke. *Current neurology and neuroscience reports*, *22*(11), 745–755. <https://doi.org/10.1007/s11910-022-01231-5>（The reason for exclusion: review）
27. Pinheiro, C., Figueiredo, J., Cerqueira, J., & Santos, C. P. (2022). Robotic Biofeedback for Post-Stroke Gait Rehabilitation: A Scoping Review. *Sensors (Basel, Switzerland)*, *22*(19), 7197. <https://doi.org/10.3390/s22197197>（The reason for exclusion: review）
28. Tseng, K. C., Wang, L., Hsieh, C., & Wong, A. M. (2024). Portable robots for upper-limb rehabilitation after stroke: a systematic review and meta-analysis. *Annals of medicine*, *56*(1), 2337735. <https://doi.org/10.1080/07853890.2024.2337735>（The reason for exclusion: review）
29. Singer, B. J., Vallence, A. M., Cleary, S., Cooper, I., & Loftus, A. M. (2013). The effect of EMG triggered electrical stimulation plus task practice on arm function in chronic stroke patients with moderate-severe arm deficits. *Restorative neurology and neuroscience*, *31*(6), 681–691. <https://doi.org/10.3233/RNN-130319>（The reason for exclusion: duplicate record）
30. Dolganov, M. V., & Karpova, M. I. (2019). Virtual'naia real'nost' pri narushenii funktsii ruki: osobennosti primeneniia v ostrom periode insul'ta [Virtual reality in upper extremity dysfunction: specific features of usage in acute stroke]. *Voprosy kurortologii, fizioterapii, i lechebnoi fizicheskoi kultury*, *96*(5), 19–28. <https://doi.org/10.17116/kurort20199605119>（The reason for exclusion: duplicate record）
31. Trombly, C. A., Thayer-Nason, L., Bliss, G., Girard, C. A., Lyrist, L. A., & Brexa-Hooson, A. (1986). The effectiveness of therapy in improving finger extension in stroke patients. *The American journal of occupational therapy : official publication of the American Occupational Therapy Association*, *40*(9), 612–617. <https://doi.org/10.5014/ajot.40.9.612>（The reason for exclusion: duplicate record）

Klamroth-Marganska V. (2018). Stroke Rehabilitation: Therapy Robots and Assistive Devices. *Advances in experimental medicine and biology*, *1065*, 579–587. https://doi.org/10.1007/978-3-319-77932-4_35（The reason for exclusion: review）

1. Takebayashi, T., Takahashi, K., Amano, S., Gosho, M., Sakai, M., Hashimoto, K., Hachisuka, K., Uchiyama, Y., & Domen, K. (2022). Robot-Assisted Training as Self-Training for Upper-Limb Hemiplegia in Chronic Stroke: A Randomized Controlled Trial. *Stroke*, *53*(7), 2182–2191. https://doi.org/10.1161/STROKEAHA.121.037260（The reason for exclusion: duplicate record）
2. Moucheboeuf, G., Griffier, R., Gasq, D., Glize, B., Bouyer, L., Dehail, P., & Cassoudesalle, H. (2020). Effects of robotic gait training after stroke: A meta-analysis. *Annals of physical and rehabilitation medicine*, *63*(6), 518–534. <https://doi.org/10.1016/j.rehab.2020.02.008>（The reason for exclusion: review）

Chien, W. T., Chong, Y. Y., Tse, M. K., Chien, C. W., & Cheng, H. Y. (2020). Robot-assisted therapy for upper-limb rehabilitation in subacute stroke patients: A systematic review and meta-analysis. *Brain and behavior*, *10*(8), e01742. https://doi.org/10.1002/brb3.1742（The reason for exclusion: review）

1. Lee, J., Kim, D. Y., Lee, S. H., Kim, J. H., Kim, D. Y., Lim, K. B., & Yoo, J. (2023). End-effector lower limb robot-assisted gait training effects in subacute stroke patients: A randomized controlled pilot trial. *Medicine*, *102*(42), e35568. https://doi.org/10.1097/MD.0000000000035568（The reason for exclusion: duplicate record）
2. Chen, Y. W., Li, K. Y., Lin, C. H., Hung, P. H., Lai, H. T., & Wu, C. Y. (2023). The effect of sequential combination of mirror therapy and robot-assisted therapy on motor function, daily function, and self-efficacy after stroke. *Scientific reports*, *13*(1), 16841. https://doi.org/10.1038/s41598-023-43981-3（The reason for exclusion: duplicate record）
3. Zhang, B., Wong, K. P., Kang, R., Fu, S., Qin, J., & Xiao, Q. (2023). Efficacy of Robot-Assisted and Virtual Reality Interventions on Balance, Gait, and Daily Function in Patients With Stroke: A Systematic Review and Network Meta-analysis. *Archives of physical medicine and rehabilitation*, *104*(10), 1711–1719. <https://doi.org/10.1016/j.apmr.2023.04.005>（The reason for exclusion: review）
4. Yang, X., Shi, X., Xue, X., & Deng, Z. (2023). Efficacy of Robot-Assisted Training on Rehabilitation of Upper Limb Function in Patients With Stroke: A Systematic Review and Meta-analysis. *Archives of physical medicine and rehabilitation*, *104*(9), 1498–1513. <https://doi.org/10.1016/j.apmr.2023.02.004>（The reason for exclusion: review）
5. Iwamoto, Y., Imura, T., Suzukawa, T., Fukuyama, H., Ishii, T., Taki, S., Imada, N., Shibukawa, M., Inagawa, T., Araki, H., & Araki, O. (2019). Combination of Exoskeletal Upper Limb Robot and Occupational Therapy Improve Activities of Daily Living Function in Acute Stroke Patients. *Journal of stroke and cerebrovascular diseases : the official journal of National Stroke Association*, *28*(7), 2018–2025. <https://doi.org/10.1016/j.jstrokecerebrovasdis.2019.03.006>（The reason for exclusion: duplicate record）
6. Akıncı, M., Burak, M., Yaşar, E., & Kılıç, R. T. (2023). The effects of Robot-assisted gait training and virtual reality on balance and gait in stroke survivors: A randomized controlled trial. *Gait & posture*, *103*, 215–222. <https://doi.org/10.1016/j.gaitpost.2023.05.013>（The reason for exclusion: duplicate record）
7. Sale, P., Franceschini, M., Mazzoleni, S., Palma, E., Agosti, M., & Posteraro, F. (2014). Effects of upper limb robot-assisted therapy on motor recovery in subacute stroke patients. *Journal of neuroengineering and rehabilitation*, *11*, 104. <https://doi.org/10.1186/1743-0003-11-104>（The reason for exclusion: duplicate record）
8. Schrader, M., Sterr, A., Kettlitz, R., Wohlmeiner, A., Buschfort, R., Dohle, C., & Bamborschke, S. (2022). The effect of mirror therapy can be improved by simultaneous robotic assistance. *Restorative neurology and neuroscience*, *40*(3), 185–194. <https://doi.org/10.3233/RNN-221263>（The reason for exclusion: duplicate record）
9. Inoue, S., Otaka, Y., Kumagai, M., Sugasawa, M., Mori, N., & Kondo, K. (2022). Effects of Balance Exercise Assist Robot training for patients with hemiparetic stroke: a randomized controlled trial. *Journal of neuroengineering and rehabilitation*, *19*(1), 12. <https://doi.org/10.1186/s12984-022-00989-6>（The reason for exclusion: duplicate record）
10. Ranzani, R., Lambercy, O., Metzger, J. C., Califfi, A., Regazzi, S., Dinacci, D., Petrillo, C., Rossi, P., Conti, F. M., & Gassert, R. (2020). Neurocognitive robot-assisted rehabilitation of hand function: a randomized control trial on motor recovery in subacute stroke. *Journal of neuroengineering and rehabilitation*, *17*(1), 115. <https://doi.org/10.1186/s12984-020-00746-7>（The reason for exclusion: duplicate record）
11. Lee, H. C., Kuo, F. L., Lin, Y. N., Liou, T. H., Lin, J. C., & Huang, S. W. (2021). Effects of Robot-Assisted Rehabilitation on Hand Function of People With Stroke: A Randomized, Crossover-Controlled, Assessor-Blinded Study. *The American journal of occupational therapy : official publication of the American Occupational Therapy Association*, *75*(1), 7501205020p1–7501205020p11. <https://doi.org/10.5014/ajot.2021.038232>（The reason for exclusion: duplicate record）
12. Novak, D., & Riener, R. (2020). Sensor Fusion in Assistive and Rehabilitation Robotics. *Sensors (Basel, Switzerland)*, *20*(18), 5235. <https://doi.org/10.3390/s20185235>（The reason for exclusion: Non robot-assisted task-oriented training）
13. Reis, S. B., Bernardo, W. M., Oshiro, C. A., Krebs, H. I., & Conforto, A. B. (2021). Effects of Robotic Therapy Associated With Noninvasive Brain Stimulation on Upper-Limb Rehabilitation After Stroke: Systematic Review and Meta-analysis of Randomized Clinical Trials. *Neurorehabilitation and neural repair*, *35*(3), 256–266. https://doi.org/10.1177/1545968321989353（The reason for exclusion: review）
14. Park, S., Fraser, M., Weber, L. M., Meeker, C., Bishop, L., Geller, D., Stein, J., & Ciocarlie, M. (2020). User-Driven Functional Movement Training With a Wearable Hand Robot After Stroke. *IEEE transactions on neural systems and rehabilitation engineering : a publication of the IEEE Engineering in Medicine and Biology Society*, *28*(10), 2265–2275. <https://doi.org/10.1109/TNSRE.2020.3021691>（The reason for exclusion: duplicate record）
15. Scano, A., Chiavenna, A., Caimmi, M., Malosio, M., Tosatti, L. M., & Molteni, F. (2017). Effect of human-robot interaction on muscular synergies on healthy people and post-stroke chronic patients. *IEEE ... International Conference on Rehabilitation Robotics : [proceedings]*, *2017*, 527–532. <https://doi.org/10.1109/ICORR.2017.8009302>（The reason for exclusion: duplicate record）
16. Lee, M. J., Lee, J. H., & Lee, S. M. (2018). Effects of robot-assisted therapy on upper extremity function and activities of daily living in hemiplegic patients: A single-blinded, randomized, controlled trial. *Technology and health care : official journal of the European Society for Engineering and Medicine*, *26*(4), 659–666. <https://doi.org/10.3233/THC-181336>（The reason for exclusion: duplicate record）
17. Hsu, H. Y., Yang, K. C., Yeh, C. H., Lin, Y. C., Lin, K. R., Su, F. C., & Kuo, L. C. (2022). A Tenodesis-Induced-Grip exoskeleton robot (TIGER) for assisting upper extremity functions in stroke patients: a randomized control study. *Disability and rehabilitation*, *44*(23), 7078–7086. <https://doi.org/10.1080/09638288.2021.1980915>（The reason for exclusion: duplicate record）
18. Ahmed, T., Islam, M. R., Brahmi, B., & Rahman, M. H. (2022). Robustness and Tracking Performance Evaluation of PID Motion Control of 7 DoF Anthropomorphic Exoskeleton Robot Assisted Upper Limb Rehabilitation. *Sensors (Basel, Switzerland)*, *22*(10), 3747. <https://doi.org/10.3390/s22103747>（The reason for exclusion: duplicate record）
19. Yue, Z., Zhang, X., & Wang, J. (2017). Hand Rehabilitation Robotics on Poststroke Motor Recovery. *Behavioural neurology*, *2017*, 3908135. <https://doi.org/10.1155/2017/3908135>（The reason for exclusion: review）
20. Hu, Y., Meng, J., Li, G., Zhao, D., Feng, G., Zuo, G., Liu, Y., Zhang, J., & Shi, C. (2023). Fuzzy Adaptive Passive Control Strategy Design for Upper-Limb End-Effector Rehabilitation Robot. *Sensors (Basel, Switzerland)*, *23*(8), 4042. https://doi.org/10.3390/s23084042（The reason for exclusion: Non-RCTs）
21. Devittori, G., Dinacci, D., Romiti, D., Califfi, A., Petrillo, C., Rossi, P., Ranzani, R., Gassert, R., & Lambercy, O. (2024). Unsupervised robot-assisted rehabilitation after stroke: feasibility, effect on therapy dose, and user experience. *Journal of neuroengineering and rehabilitation*, *21*(1), 52. https://doi.org/10.1186/s12984-024-01347-4（The reason for exclusion: Non robot-assisted task-oriented training）
22. O'Malley, M. K., Ro, T., & Levin, H. S. (2006). Assessing and inducing neuroplasticity with transcranial magnetic stimulation and robotics for motor function. *Archives of physical medicine and rehabilitation*, *87*(12 Suppl 2), S59–S66. https://doi.org/10.1016/j.apmr.2006.08.332（The reason for exclusion: review）
23. Balasubramanian, S., Klein, J., & Burdet, E. (2010). Robot-assisted rehabilitation of hand function. *Current opinion in neurology*, *23*(6), 661–670. <https://doi.org/10.1097/WCO.0b013e32833e99a4>（The reason for exclusion: review）
24. Sun, Z., Mu, A., Wang, C., Liu, Q., Hao, F., Wei, J., & Li, W. (2023). Research on an ankle rehabilitation robot for hemiplegic patients after stroke. *Proceedings of the Institution of Mechanical Engineers. Part H, Journal of engineering in medicine*, *237*(10), 1177–1189. https://doi.org/10.1177/09544119231197082（The reason for exclusion: Non-RCTs）
25. Saragih, I. D., Everard, G., Tzeng, H. M., Saragih, I. S., & Lee, B. O. (2023). Efficacy of Robots-Assisted Therapy in Patients With Stroke: A Meta-analysis Update. *The Journal of cardiovascular nursing*, *38*(6), E192–E217. <https://doi.org/10.1097/JCN.0000000000000945>（The reason for exclusion: review）
26. Tran, V. D., Dario, P., & Mazzoleni, S. (2018). Kinematic measures for upper limb robot-assisted therapy following stroke and correlations with clinical outcome measures: A review. *Medical engineering & physics*, *53*, 13–31. <https://doi.org/10.1016/j.medengphy.2017.12.005>（The reason for exclusion: review）
27. Shin, S. Y., Hohl, K., Giffhorn, M., Awad, L. N., Walsh, C. J., & Jayaraman, A. (2022). Soft robotic exosuit augmented high intensity gait training on stroke survivors: a pilot study. *Journal of neuroengineering and rehabilitation*, *19*(1), 51. https://doi.org/10.1186/s12984-022-01034-2（The reason for exclusion: Irrelevant study outcome）
28. Dehem, S., Gilliaux, M., Stoquart, G., Detrembleur, C., Jacquemin, G., Palumbo, S., Frederick, A., & Lejeune, T. (2019). Effectiveness of upper-limb robotic-assisted therapy in the early rehabilitation phase after stroke: A single-blind, randomised, controlled trial. *Annals of physical and rehabilitation medicine*, *62*(5), 313–320. <https://doi.org/10.1016/j.rehab.2019.04.002>（The reason for exclusion: duplicate record）
29. Castelli E. (2023). Robotic Rehabilitation in Children. *Psychiatria Danubina*, *35*(Suppl 3), 93–94.（The reason for exclusion: Non robot-assisted task-oriented training）
30. Wang, J., Li, Y., Qi, L., Mamtilahun, M., Liu, C., Liu, Z., Shi, R., Wu, S., & Yang, G. Y. (2024). Advanced rehabilitation in ischaemic stroke research. *Stroke and vascular neurology*, *9*(4), 328–343. https://doi.org/10.1136/svn-2022-002285（The reason for exclusion: review）
31. Pignolo L. (2009). Robotics in neuro-rehabilitation. *Journal of rehabilitation medicine*, *41*(12), 955–960. https://doi.org/10.2340/16501977-0434（The reason for exclusion: Non-RCTs）
32. Bui, K. D., & Johnson, M. J. (2018). Designing robot-assisted neurorehabilitation strategies for people with both HIV and stroke. *Journal of neuroengineering and rehabilitation*, *15*(1), 75. https://doi.org/10.1186/s12984-018-0418-3（The reason for exclusion: review）
33. Carrillo, C., Tilley, D., Horn, K., Gonzalez, M., Coffman, C., Hilton, C., & Mani, K. (2023). Effectiveness of Robotics in Stroke Rehabilitation to Accelerate Upper Extremity Function: Systematic Review. *Occupational therapy international*, *2023*, 7991765. https://doi.org/10.1155/2023/7991765（The reason for exclusion: review）
34. Cinnera, A. M., Bonnì, S., D'Acunto, A., Maiella, M., Ferraresi, M., Casula, E. P., Pezzopane, V., Tramontano, M., Iosa, M., Paolucci, S., Morone, G., Vannozzi, G., & Koch, G. (2023). Cortico-cortical stimulation and robot-assisted therapy (CCS and RAT) for upper limb recovery after stroke: study protocol for a randomised controlled trial. *Trials*, *24*(1), 823. https://doi.org/10.1186/s13063-023-07849-1（The reason for exclusion: Control group using additional interventions)
35. Kim, E., Lee, G., Lee, J., & Kim, Y. H. (2024). Simultaneous high-definition transcranial direct current stimulation and robot-assisted gait training in stroke patients. *Scientific reports*, *14*(1), 4483. https://doi.org/10.1038/s41598-024-53482-6（The reason for exclusion: Control group using additional interventions)
36. Stockbridge, M. D., Bunker, L. D., & Hillis, A. E. (2022). Reversing the Ruin: Rehabilitation, Recovery, and Restoration After Stroke. *Current neurology and neuroscience reports*, *22*(11), 745–755. <https://doi.org/10.1007/s11910-022-01231-5>（The reason for exclusion: review）
37. Pinheiro, C., Figueiredo, J., Cerqueira, J., & Santos, C. P. (2022). Robotic Biofeedback for Post-Stroke Gait Rehabilitation: A Scoping Review. *Sensors (Basel, Switzerland)*, *22*(19), 7197. <https://doi.org/10.3390/s22197197>（The reason for exclusion: review）
38. Tseng, K. C., Wang, L., Hsieh, C., & Wong, A. M. (2024). Portable robots for upper-limb rehabilitation after stroke: a systematic review and meta-analysis. *Annals of medicine*, *56*(1), 2337735. <https://doi.org/10.1080/07853890.2024.2337735>（The reason for exclusion: review）
39. Chen, Z., Wang, C., Fan, W., Gu, M., Yasin, G., Xiao, S., Huang, J., & Huang, X. (2020). Robot-Assisted Arm Training versus Therapist-Mediated Training after Stroke: A Systematic Review and Meta-Analysis. *Journal of healthcare engineering*, *2020*, 8810867. <https://doi.org/10.1155/2020/8810867>（The reason for exclusion: review）
40. Zhao, M., Wang, G., Wang, A., Cheng, L. J., & Lau, Y. (2022). Robot-assisted distal training improves upper limb dexterity and function after stroke: a systematic review and meta-regression. *Neurological sciences : official journal of the Italian Neurological Society and of the Italian Society of Clinical Neurophysiology*, *43*(3), 1641–1657. <https://doi.org/10.1007/s10072-022-05913-3>（The reason for exclusion: review）
41. Ji EK, Wang HH, Jung SJ, et al. Graded motor imagery training as a home exercise program for upper limb motor function in patients with chronic stroke: A randomized controlled trial. Medicine (Baltimore). 2021;100(3):e24351. doi:10.1097/MD.0000000000024351 （The reason for exclusion: Non robot-assisted task-oriented training）
42. Ranzani R, Lambercy O, Metzger JC, et al. Neurocognitive robot-assisted rehabilitation of hand function: a randomized control trial on motor recovery in subacute stroke. J Neuroeng Rehabil. 2020;17(1):115. Published 2020 Aug 24. doi:10.1186/s12984-020-00746-7 （The reason for exclusion: duplicate record）
43. Lee HC, Kuo FL, Lin YN, Liou TH, Lin JC, Huang SW. Effects of Robot-Assisted Rehabilitation on Hand Function of People With Stroke: A Randomized, Crossover-Controlled, Assessor-Blinded Study. Am J Occup Ther. 2021;75(1):7501205020p1-7501205020p11. doi:10.5014/ajot.2021.038232 （The reason for exclusion: duplicate record）
44. Villafañe JH, Taveggia G, Galeri S, et al. Efficacy of Short-Term Robot-Assisted Rehabilitation in Patients With Hand Paralysis After Stroke: A Randomized Clinical Trial. Hand (N Y). 2018;13(1):95-102. doi:10.1177/1558944717692096 （The reason for exclusion: duplicate record）
45. Du J, Wang S, Cheng Y, et al. Effects of Neuromuscular Electrical Stimulation Combined with Repetitive Transcranial Magnetic Stimulation on Upper Limb Motor Function Rehabilitation in Stroke Patients with Hemiplegia [retracted in: Comput Math Methods Med. 2023 Nov 1;2023:9767295. doi: 10.1155/2023/9767295]. Comput Math Methods Med. 2022;2022:9455428. Published 2022 Jan 4. doi:10.1155/2022/9455428 （The reason for exclusion: Non robot-assisted task-oriented training）
46. Huang YC, Chen PC, Tso HH, Yang YC, Ho TL, Leong CP. Effects of kinesio taping on hemiplegic hand in patients with upper limb post-stroke spasticity: a randomized controlled pilot study. Eur J Phys Rehabil Med. 2019;55(5):551-557. doi:10.23736/S1973-9087.19.05684-3 （The reason for exclusion: Non robot-assisted task-oriented training）
47. Aguilera-Rubio Á, Alguacil-Diego IM, Mallo-López A, Jardón Huete A, Oña ED, Cuesta-Gómez A. Use of low-cost virtual reality in the treatment of the upper extremity in chronic stroke: a randomized clinical trial. J Neuroeng Rehabil. 2024;21(1):12. Published 2024 Jan 22. doi:10.1186/s12984-024-01303-2 （The reason for exclusion: Non robot-assisted task-oriented training）
48. Tang C, Zhou T, Zhang Y, et al. Bilateral upper limb robot-assisted rehabilitation improves upper limb motor function in stroke patients: a study based on quantitative EEG. Eur J Med Res. 2023;28(1):603. Published 2023 Dec 19. doi:10.1186/s40001-023-01565-x （The reason for exclusion: duplicate record）
49. Heineman JT, Forster GL, Stephens KL, Cottler PS, Timko MP, DeGeorge BR Jr. A Randomized Controlled Trial of Topical Cannabidiol for the Treatment of Thumb Basal Joint Arthritis. J Hand Surg Am. 2022;47(7):611-620. doi:10.1016/j.jhsa.2022.03.002 （The reason for exclusion: Non robot-assisted task-oriented training）
50. Xia, X., Dong, X., Huo, H., Zhang, Y., Song, J., & Wang, D. (2023). Clinical study of low-frequency acupoint electrical stimulation to improve thumb-to-finger movements after stroke: A randomized controlled trial. *Medicine*, *102*(47), e35755. <https://doi.org/10.1097/MD.0000000000035755> （The reason for exclusion: Non robot-assisted task-oriented training）
51. Vanoglio, F., Comini, L., Gaiani, M., Bonometti, G. P., Luisa, A., & Bernocchi, P. (2024). A Sensor-Based Upper Limb Treatment in Hemiplegic Patients: Results from a Randomized Pilot Study. *Sensors (Basel, Switzerland)*, *24*(8), 2574. <https://doi.org/10.3390/s24082574> （The reason for exclusion: Non robot-assisted task-oriented training）
52. Gentilucci M, Negrotti A, Gangitano M. Planning an action. Exp Brain Res. 1997;115(1):116-128. doi:10.1007/pl00005671Gentilucci M, Negrotti A, Gangitano M. Planning an action. Exp Brain Res. 1997;115(1):116-128. doi:10.1007/pl00005671 （The reason for exclusion: Non-RCTs）
53. Giuffre, A., Zewdie, E., Carlson, H. L., Wrightson, J. G., Kuo, H. C., Cole, L., & Kirton, A. (2021). Robotic transcranial magnetic stimulation motor maps and hand function in adolescents. *Physiological reports*, *9*(7), e14801. <https://doi.org/10.14814/phy2.14801> （The reason for exclusion: Irrelevant study outcome）
54. de Jong, T. R., Bonhof-Jansen, E. E. D. J., Brink, S. M., de Wildt, R. P., van Uchelen, J. H., & Werker, P. M. N. (2023). Total joint arthroplasty versus trapeziectomy in the treatment of trapeziometacarpal joint arthritis: a randomized controlled trial. The Journal of hand surgery, European volume, 48(9), 884–894. https://doi.org/10.1177/17531934231185245（The reason for exclusion: Non robot-assisted task-oriented training）
55. Barnard, A., Jansen, V., Swindells, M. G., Arundell, M., & Burke, F. D. (2020). A randomized controlled trial of real versus sham acupuncture for basal thumb joint arthritis. *The Journal of hand surgery, European volume*, *45*(5), 488–494. https://doi.org/10.1177/1753193420911326（The reason for exclusion: Non robot-assisted task-oriented training）
56. Klim, S. M., Glehr, R., Graef, A., Amerstorfer, F., Leithner, A., & Glehr, M. (2023). Total joint arthroplasty versus resection-interposition arthroplasty for thumb carpometacarpal arthritis: a randomized controlled trial. *Acta orthopaedica*, *94*, 224–229. https://doi.org/10.2340/17453674.2023.11919（The reason for exclusion: Non robot-assisted task-oriented training）
57. van Ravestyn, C., Gerardin, E., Térémetz, M., Hamdoun, S., Baron, J. C., Calvet, D., Vandermeeren, Y., Turc, G., Maier, M. A., Rosso, C., Mas, J. L., Dupin, L., & Lindberg, P. G. (2024). Post-Stroke Impairments of Manual Dexterity and Finger Proprioception: Their Contribution to Upper Limb Activity Capacity. Neurorehabilitation and neural repair, 38(5), 373–385. https://doi.org/10.1177/15459683241245416（The reason for exclusion: Non-RCTs）
58. Chen, L., Chen, Y., Fu, W. B., Huang, D. F., & Lo, W. L. A. (2022). The Effect of Virtual Reality on Motor Anticipation and Hand Function in Patients with Subacute Stroke: A Randomized Trial on Movement-Related Potential. *Neural plasticity*, *2022*, 7399995. https://doi.org/10.1155/2022/7399995（The reason for exclusion: Non robot-assisted task-oriented training）
59. Hesse, S., Tomelleri, C., Bardeleben, A., Werner, C., & Waldner, A. (2012). Robot-assisted practice of gait and stair climbing in nonambulatory stroke patients. *Journal of rehabilitation research and development*, *49*(4), 613–622. <https://doi.org/10.1682/jrrd.2011.08.0142>（The reason for exclusion: duplicate record）
60. Mazzoleni, S., Focacci, A., Franceschini, M., Waldner, A., Spagnuolo, C., Battini, E., & Bonaiuti, D. (2017). Robot-assisted end-effector-based gait training in chronic stroke patients: A multicentric uncontrolled observational retrospective clinical study. *NeuroRehabilitation*, *40*(4), 483–492. <https://doi.org/10.3233/NRE-161435>（The reason for exclusion: duplicate record）
61. Klinkwan, P., Kongmaroeng, C., Muengtaweepongsa, S., & Limtrakarn, W. (2023). Prototype development of bilateral arm mirror-like-robotic rehabilitation device for acute stroke patients. *Biomedical physics & engineering express*, *9*(4), 10.1088/2057-1976/acd11d. <https://doi.org/10.1088/2057-1976/acd11d>（The reason for exclusion: Non robot-assisted task-oriented training）
62. Li, N., Yang, T., Yu, P., Chang, J., Zhao, L., Zhao, X., Elhajj, I. H., Xi, N., & Liu, L. (2018). Bio-inspired upper limb soft exoskeleton to reduce stroke-induced complications. *Bioinspiration & biomimetics*, *13*(6), 066001. <https://doi.org/10.1088/1748-3190/aad8d4>（The reason for exclusion: Non robot-assisted task-oriented training）
63. Yakşi, E., Bahadır, E. S., Yaşar, M. F., Alışık, T., Kurul, R., & Demirel, A. (2023). The effect of robot-assisted gait training frequency on walking, functional recovery, and quality of life in patients with stroke. *Acta neurologica Belgica*, *123*(2), 583–590. <https://doi.org/10.1007/s13760-023-02194-1>（The reason for exclusion: duplicate record）
64. Lim, D. Y., Lai, H. S., & Yeow, R. C. (2023). A bidirectional fabric-based soft robotic glove for hand function assistance in patients with chronic stroke. *Journal of neuroengineering and rehabilitation*, *20*(1), 120. https://doi.org/10.1186/s12984-023-01250-4（The reason for exclusion: duplicate record）
65. Neo, J. R. E., Visperas, C. A., Tan, M. P. H., & Tay, S. S. (2023). Novel use of robot-assisted gait rehabilitation in a patient with stroke and blindness. *BMJ case reports*, *16*(7), e255457. <https://doi.org/10.1136/bcr-2023-255457>（The reason for exclusion: Non-RCTs）
66. Sayın, A. M., Duruturk, N., Balaban, B., & Korkusuz, S. (2023). The effect of robot-assisted walking in different modalities on cardiorespiratory responses and energy consumption in patients with subacute stroke. *Neurological research*, *45*(7), 688–694. <https://doi.org/10.1080/01616412.2023.2188520>（The reason for exclusion: Non-RCTs）
67. Uehara, S., Yuasa, A., Ushizawa, K., Kitamura, S., Yamazaki, K., Otaka, E., & Otaka, Y. (2023). Direction-dependent differences in the quality and quantity of horizontal reaching in people after stroke. *Journal of neurophysiology*, *130*(4), 861–870. <https://doi.org/10.1152/jn.00455.2022>（The reason for exclusion: duplicate record）
68. Tomida, K., Sonoda, S., Hirano, S., Suzuki, A., Tanino, G., Kawakami, K., Saitoh, E., & Kagaya, H. (2019). Randomized Controlled Trial of Gait Training Using Gait Exercise Assist Robot (GEAR) in Stroke Patients with Hemiplegia. *Journal of stroke and cerebrovascular diseases : the official journal of National Stroke Association*, *28*(9), 2421–2428. <https://doi.org/10.1016/j.jstrokecerebrovasdis.2019.06.030>（The reason for exclusion: duplicate record）
69. Volpe, B. T., Ferraro, M., Lynch, D., Christos, P., Krol, J., Trudell, C., Krebs, H. I., & Hogan, N. (2005). Robotics and other devices in the treatment of patients recovering from stroke. *Current neurology and neuroscience reports*, *5*(6), 465–470. <https://doi.org/10.1007/s11910-005-0035-y>（The reason for exclusion: duplicate record）
70. Shin, J., An, H., Yang, S., Park, C., Lee, Y., & You, S. J. H. (2022). Comparative effects of passive and active mode robot-assisted gait training on brain and muscular activities in sub-acute and chronic stroke. *NeuroRehabilitation*, *51*(1), 51–63. <https://doi.org/10.3233/NRE-210304>（The reason for exclusion: duplicate record）
71. Rikhof, C. J. H., Feenstra, Y., Fleuren, J. F. M., Buurke, J. H., Prinsen, E. C., Rietman, J. S., & Prange-Lasonder, G. B. (2024). Robot-assisted support combined with electrical stimulation for the lower extremity in stroke patients: a systematic review. *Journal of neural engineering*, *21*(2), 10.1088/1741-2552/ad377c. <https://doi.org/10.1088/1741-2552/ad377c>（The reason for exclusion: review）
72. Mehrholz J. (2019). Is Electromechanical and Robot-Assisted Arm Training Effective for Improving Arm Function in People Who Have Had a Stroke?: A Cochrane Review Summary With Commentary. *American journal of physical medicine & rehabilitation*, *98*(4), 339–340. <https://doi.org/10.1097/PHM.0000000000001133>（The reason for exclusion: review）
73. Semrau, J. A., Herter, T. M., Scott, S. H., & Dukelow, S. P. (2015). Examining Differences in Patterns of Sensory and Motor Recovery After Stroke With Robotics. *Stroke*, *46*(12), 3459–3469. <https://doi.org/10.1161/STROKEAHA.115.010750>（The reason for exclusion: duplicate record）
74. Chen, Y. W., Chiang, W. C., Chang, C. L., Lo, S. M., & Wu, C. Y. (2022). Comparative effects of EMG-driven robot-assisted therapy versus task-oriented training on motor and daily function in patients with stroke: a randomized cross-over trial. *Journal of neuroengineering and rehabilitation*, *19*(1), 6. <https://doi.org/10.1186/s12984-021-00961-w>（The reason for exclusion: duplicate record）
75. Yurkewich, A., Hebert, D., Wang, R. H., & Mihailidis, A. (2019). Hand Extension Robot Orthosis (HERO) Glove: Development and Testing With Stroke Survivors With Severe Hand Impairment. *IEEE transactions on neural systems and rehabilitation engineering : a publication of the IEEE Engineering in Medicine and Biology Society*, *27*(5), 916–926. <https://doi.org/10.1109/TNSRE.2019.2910011>（The reason for exclusion: duplicate record）
76. Joo, M. C., Jung, K. M., Kim, J. H., Jung, Y. J., Chang, W. N., & Shin, H. J. (2022). Robot-Assisted Therapy Combined with Trunk Restraint in Acute Stroke Patients: A Randomized Controlled Study. *Journal of stroke and cerebrovascular diseases : the official journal of National Stroke Association*, *31*(5), 106330. <https://doi.org/10.1016/j.jstrokecerebrovasdis.2022.106330>（The reason for exclusion: duplicate record）
77. Yeung, L. F., Lau, C. C. Y., Lai, C. W. K., Soo, Y. O. Y., Chan, M. L., & Tong, R. K. Y. (2021). Effects of wearable ankle robotics for stair and over-ground training on sub-acute stroke: a randomized controlled trial. *Journal of neuroengineering and rehabilitation*, *18*(1), 19. <https://doi.org/10.1186/s12984-021-00814-6>（The reason for exclusion: duplicate record）
78. Thimabut, N., Yotnuengnit, P., Charoenlimprasert, J., Sillapachai, T., Hirano, S., Saitoh, E., & Piravej, K. (2022). Effects of the Robot-Assisted Gait Training Device Plus Physiotherapy in Improving Ambulatory Functions in Patients With Subacute Stroke With Hemiplegia: An Assessor-Blinded, Randomized Controlled Trial. *Archives of physical medicine and rehabilitation*, *103*(5), 843–850. <https://doi.org/10.1016/j.apmr.2022.01.146>（The reason for exclusion: duplicate record）
79. Volpe, B. T., Krebs, H. I., Hogan, N., Edelstein OTR, L., Diels, C., & Aisen, M. (2000). A novel approach to stroke rehabilitation: robot-aided sensorimotor stimulation. *Neurology*, *54*(10), 1938–1944. <https://doi.org/10.1212/wnl.54.10.1938>（The reason for exclusion: duplicate record
80. Duret, C., Courtial, O., Grosmaire, A. G., & Hutin, E. (2015). Use of a robotic device for the rehabilitation of severe upper limb paresis in subacute stroke: exploration of patient/robot interactions and the motor recovery process. *BioMed research international*, *2015*, 482389. <https://doi.org/10.1155/2015/482389>（The reason for exclusion: Non-RCTs）
81. Colombo, R., Pisano, F., Delconte, C., Mazzone, A., Grioni, G., Castagna, M., Bazzini, G., Imarisio, C., Maggioni, G., & Pistarini, C. (2017). Comparison of exercise training effect with different robotic devices for upper limb rehabilitation: a retrospective study. *European journal of physical and rehabilitation medicine*, *53*(2), 240–248. https://doi.org/10.23736/S1973-9087.16.04297-0（The reason for exclusion: Non-RCTs）
82. Rowe, J. B., Chan, V., Ingemanson, M. L., Cramer, S. C., Wolbrecht, E. T., & Reinkensmeyer, D. J. (2017). Robotic Assistance for Training Finger Movement Using a Hebbian Model: A Randomized Controlled Trial. *Neurorehabilitation and neural repair*, *31*(8), 769–780. <https://doi.org/10.1177/1545968317721975>（The reason for exclusion: duplicate record）
83. Lo, A. C., Guarino, P. D., Richards, L. G., Haselkorn, J. K., Wittenberg, G. F., Federman, D. G., Ringer, R. J., Wagner, T. H., Krebs, H. I., Volpe, B. T., Bever, C. T., Jr, Bravata, D. M., Duncan, P. W., Corn, B. H., Maffucci, A. D., Nadeau, S. E., Conroy, S. S., Powell, J. M., Huang, G. D., & Peduzzi, P. (2010). Robot-assisted therapy for long-term upper-limb impairment after stroke. *The New England journal of medicine*, *362*(19), 1772–1783. <https://doi.org/10.1056/NEJMoa0911341>（The reason for exclusion: duplicate record）
84. Taheri, H., Rowe, J. B., Gardner, D., Chan, V., Reinkensmeyer, D. J., & Wolbrecht, E. T. (2012). Robot-assisted Guitar Hero for finger rehabilitation after stroke. *Annual International Conference of the IEEE Engineering in Medicine and Biology Society. IEEE Engineering in Medicine and Biology Society. Annual International Conference*, *2012*, 3911–3917. <https://doi.org/10.1109/EMBC.2012.6346822>（The reason for exclusion: meeting abstract）
85. Lin, Y., Qu, Q., Lin, Y., He, J., Zhang, Q., Wang, C., Jiang, Z., Guo, F., & Jia, J. (2021). Customizing Robot-Assisted Passive Neurorehabilitation Exercise Based on Teaching Training Mechanism. *BioMed research international*, *2021*, 9972560. <https://doi.org/10.1155/2021/9972560>（The reason for exclusion: duplicate record）
86. Chan, H. L., Hung, J. W., Chang, K. C., & Wu, C. Y. (2021). Myoelectric analysis of upper-extremity muscles during robot-assisted bilateral wrist flexion-extension in subjects with poststroke hemiplegia. *Clinical biomechanics (Bristol, Avon)*, *87*, 105412. <https://doi.org/10.1016/j.clinbiomech.2021.105412>（The reason for exclusion: duplicate record）
87. Ru, H., Gao, W., Ou, W., Yang, X., Li, A., Fu, Z., Huo, J., Yang, B., Zhang, Y., Xiao, X., Yang, Z., & Huang, J. (2023). A Flexible Wearable Supernumerary Robotic Limb for Chronic Stroke Patients. *Journal of visualized experiments : JoVE*, (200), 10.3791/65917. <https://doi.org/10.3791/65917>（The reason for exclusion: duplicate record）
88. Hirano, S., Saitoh, E., Imoto, D., Ii, T., Tsunoda, T., & Otaka, Y. (2024). Effects of robot-assisted gait training using the Welwalk on gait independence for individuals with hemiparetic stroke: an assessor-blinded, multicenter randomized controlled trial. *Journal of neuroengineering and rehabilitation*, *21*(1), 76. <https://doi.org/10.1186/s12984-024-01370-5>（The reason for exclusion: duplicate record）
89. Wang, L., Zheng, Y., Dang, Y., Teng, M., Zhang, X., Cheng, Y., Zhang, X., Yu, Q., Yin, A., & Lu, X. (2021). Effects of robot-assisted training on balance function in patients with stroke: A systematic review and meta-analysis. *Journal of rehabilitation medicine*, *53*(4), jrm00174. <https://doi.org/10.2340/16501977-2815>（The reason for exclusion: review）
90. Zheng, Q. X., Ge, L., Wang, C. C., Ma, Q. S., Liao, Y. T., Huang, P. P., Wang, G. D., Xie, Q. L., & Rask, M. (2019). Robot-assisted therapy for balance function rehabilitation after stroke: A systematic review and meta-analysis. *International journal of nursing studies*, *95*, 7–18. <https://doi.org/10.1016/j.ijnurstu.2019.03.015>（The reason for exclusion: review）
91. Leconte, P., & Ronsse, R. (2016). Performance-based robotic assistance during rhythmic arm exercises. *Journal of neuroengineering and rehabilitation*, *13*(1), 82. <https://doi.org/10.1186/s12984-016-0189-7>（The reason for exclusion: duplicate record）
92. Lo, A. C., Guarino, P., Krebs, H. I., Volpe, B. T., Bever, C. T., Duncan, P. W., Ringer, R. J., Wagner, T. H., Richards, L. G., Bravata, D. M., Haselkorn, J. K., Wittenberg, G. F., Federman, D. G., Corn, B. H., Maffucci, A. D., & Peduzzi, P. (2009). Multicenter randomized trial of robot-assisted rehabilitation for chronic stroke: methods and entry characteristics for VA ROBOTICS. *Neurorehabilitation and neural repair*, *23*(8), 775–783. <https://doi.org/10.1177/1545968309338195>（The reason for exclusion: duplicate record）
93. Hung, C. S., Hsieh, Y. W., Wu, C. Y., Lin, Y. T., Lin, K. C., & Chen, C. L. (2016). The Effects of Combination of Robot-Assisted Therapy With Task-Specific or Impairment-Oriented Training on Motor Function and Quality of Life in Chronic Stroke. *PM & R : the journal of injury, function, and rehabilitation*, *8*(8), 721–729. <https://doi.org/10.1016/j.pmrj.2016.01.008>（The reason for exclusion: duplicate record）
94. Lee, S. Y., Choi, Y. S., Kim, M. H., & Chang, W. N. (2024). Effects of robot-assisted walking training on balance, motor function, and ADL depending on severity levels in stroke patients. *Technology and health care : official journal of the European Society for Engineering and Medicine*, *32*(5), 3293–3307. <https://doi.org/10.3233/THC-232015>（The reason for exclusion: duplicate record）
95. Bay, B., Kiwus, L. M., Goßling, A., Koester, L., Blaum, C., Schrage, B., Clemmensen, P., Blankenberg, S., Waldeyer, C., Seiffert, M., & Brunner, F. J. (2024). Procedural and one-year outcomes of robotic-assisted versus manual percutaneous coronary intervention. *EuroIntervention : journal of EuroPCR in collaboration with the Working Group on Interventional Cardiology of the European Society of Cardiology*, *20*(1), 56–65. <https://doi.org/10.4244/EIJ-D-23-00375>（The reason for exclusion: duplicate record）
96. Devittori, G., Ranzani, R., Dinacci, D., Romiti, D., Califfi, A., Petrillo, C., Rossi, P., Gassert, R., & Lambercy, O. (2022). Automatic and Personalized Adaptation of Therapy Parameters for Unsupervised Robot-Assisted Rehabilitation: a Pilot Evaluation. *IEEE ... International Conference on Rehabilitation Robotics : [proceedings]*, *2022*, 1–6. <https://doi.org/10.1109/ICORR55369.2022.9896527>（The reason for exclusion: duplicate record）
97. Leonardis, D., Barsotti, M., Loconsole, C., Solazzi, M., Troncossi, M., Mazzotti, C., Castelli, V. P., Procopio, C., Lamola, G., Chisari, C., Bergamasco, M., & Frisoli, A. (2015). An EMG-Controlled Robotic Hand Exoskeleton for Bilateral Rehabilitation. *IEEE transactions on haptics*, *8*(2), 140–151. <https://doi.org/10.1109/TOH.2015.2417570>（The reason for exclusion: duplicate record）
98. Vahdat, S., Darainy, M., Thiel, A., & Ostry, D. J. (2019). A Single Session of Robot-Controlled Proprioceptive Training Modulates Functional Connectivity of Sensory Motor Networks and Improves Reaching Accuracy in Chronic Stroke. *Neurorehabilitation and neural repair*, *33*(1), 70–81. <https://doi.org/10.1177/1545968318818902>（The reason for exclusion: duplicate record）
99. Park J. H. (2021). The effects of robot-assisted left-hand training on hemispatial neglect in older patients with chronic stroke: A pilot and randomized controlled trial. *Medicine*, *100*(9), e24781. <https://doi.org/10.1097/MD.0000000000024781>（The reason for exclusion: duplicate record）
100. Pai, M. Y. B., Terranova, T. T., Simis, M., Fregni, F., & Battistella, L. R. (2018). The Combined Use of Transcranial Direct Current Stimulation and Robotic Therapy for the Upper Limb. *Journal of visualized experiments : JoVE*, (139), 58495. <https://doi.org/10.3791/58495>（The reason for exclusion: duplicate record）
101. Park, C., Oh-Park, M., Bialek, A., Friel, K., Edwards, D., & You, J. S. H. (2021). Abnormal synergistic gait mitigation in acute stroke using an innovative ankle-knee-hip interlimb humanoid robot: a preliminary randomized controlled trial. *Scientific reports*, *11*(1), 22823. <https://doi.org/10.1038/s41598-021-01959-z>（The reason for exclusion: duplicate record）
102. Thimabut, N., Yotnuengnit, P., Charoenlimprasert, J., Sillapachai, T., Hirano, S., Saitoh, E., & Piravej, K. (2022). Effects of the Robot-Assisted Gait Training Device Plus Physiotherapy in Improving Ambulatory Functions in Patients With Subacute Stroke With Hemiplegia: An Assessor-Blinded, Randomized Controlled Trial. *Archives of physical medicine and rehabilitation*, *103*(5), 843–850. <https://doi.org/10.1016/j.apmr.2022.01.146>（The reason for exclusion: duplicate record）
103. Bernal-Jiménez, J. J., Dileone, M., Mordillo-Mateos, L., Martín-Conty, J. L., Durantez-Fernández, C., Viñuela, A., Martín-Rodríguez, F., Lerin-Calvo, A., Alcántara-Porcuna, V., & Polonio-López, B. (2024). Combining Transcranial Direct Current Stimulation With Hand Robotic Rehabilitation in Chronic Stroke Patients: A Double-Blind Randomized Clinical Trial. *American journal of physical medicine & rehabilitation*, *103*(10), 875–882. <https://doi.org/10.1097/PHM.0000000000002446>（The reason for exclusion: duplicate record）
104. Proulx, C. E., Higgins, J., & Gagnon, D. H. (2023). Occupational therapists' evaluation of the perceived usability and utility of wearable soft robotic exoskeleton gloves for hand function rehabilitation following a stroke. *Disability and rehabilitation. Assistive technology*, *18*(6), 953–962. <https://doi.org/10.1080/17483107.2021.1938710>（The reason for exclusion: duplicate record）
105. Rikhof, C. J. H., Leerskov, K. S., Prange-Lasonder, G. B., Prinsen, E. C., Spaich, E. G., Dosen, S., Struijk, L. N. S. A., Buurke, J. H., & Rietman, J. S. (2024). Combining robotics and functional electrical stimulation for assist-as-needed support of leg movements in stroke patients: A feasibility study. *Medical engineering & physics*, *130*, 104216. <https://doi.org/10.1016/j.medengphy.2024.104216>（The reason for exclusion: duplicate record）
106. Skovgaard Jensen, J., Sørensen, A. S., Kruuse, C., Nielsen, H. H., Skov, C. D., Jensen, H. B., Buckwalter, M. S., Bojsen-Møller, J., Lambertsen, K. L., & Holsgaard-Larsen, A. (2024). The effect of robot-assisted versus standard training on motor function following subacute rehabilitation after ischemic stroke - protocol for a randomised controlled trial nested in a prospective cohort (RoboRehab). *BMC neurology*, *24*(1), 233. <https://doi.org/10.1186/s12883-024-03734-9>（The reason for exclusion: duplicate record）
107. Lin, Y., Qu, Q., Lin, Y., He, J., Zhang, Q., Wang, C., Jiang, Z., Guo, F., & Jia, J. (2021). Customizing Robot-Assisted Passive Neurorehabilitation Exercise Based on Teaching Training Mechanism. *BioMed research international*, *2021*, 9972560. <https://doi.org/10.1155/2021/9972560>（The reason for exclusion: duplicate record）
108. Chan, H. L., Hung, J. W., Chang, K. C., & Wu, C. Y. (2021). Myoelectric analysis of upper-extremity muscles during robot-assisted bilateral wrist flexion-extension in subjects with poststroke hemiplegia. *Clinical biomechanics (Bristol, Avon)*, *87*, 105412. <https://doi.org/10.1016/j.clinbiomech.2021.105412>（The reason for exclusion: duplicate record）
109. Ru, H., Gao, W., Ou, W., Yang, X., Li, A., Fu, Z., Huo, J., Yang, B., Zhang, Y., Xiao, X., Yang, Z., & Huang, J. (2023). A Flexible Wearable Supernumerary Robotic Limb for Chronic Stroke Patients. *Journal of visualized experiments : JoVE*, (200), 10.3791/65917. <https://doi.org/10.3791/65917>（The reason for exclusion: duplicate record）
110. Hirano, S., Saitoh, E., Imoto, D., Ii, T., Tsunoda, T., & Otaka, Y. (2024). Effects of robot-assisted gait training using the Welwalk on gait independence for individuals with hemiparetic stroke: an assessor-blinded, multicenter randomized controlled trial. *Journal of neuroengineering and rehabilitation*, *21*(1), 76. <https://doi.org/10.1186/s12984-024-01370-5>（The reason for exclusion: duplicate record）
111. Wang, L., Zheng, Y., Dang, Y., Teng, M., Zhang, X., Cheng, Y., Zhang, X., Yu, Q., Yin, A., & Lu, X. (2021). Effects of robot-assisted training on balance function in patients with stroke: A systematic review and meta-analysis. *Journal of rehabilitation medicine*, *53*(4), jrm00174. <https://doi.org/10.2340/16501977-2815>（The reason for exclusion: review）
112. Amano, Y., Noma, T., Etoh, S., Miyata, R., Kawamura, K., & Shimodozono, M. (2020). Reaching exercise for chronic paretic upper extremity after stroke using a novel rehabilitation robot with arm-weight support and concomitant electrical stimulation and vibration: before-and-after feasibility trial. *Biomedical engineering online*, *19*(1), 28. <https://doi.org/10.1186/s12938-020-00774-3>（The reason for exclusion: Non robot-assisted task-oriented training）
113. Tanaka, N., Matsushita, S., Sonoda, Y., Maruta, Y., Fujitaka, Y., Sato, M., Simomori, M., Onaka, R., Harada, K., Hirata, T., Kinoshita, S., Okamoto, T., & Okamura, H. (2019). Effect of Stride Management Assist Gait Training for Poststroke Hemiplegia: A Single Center, Open-Label, Randomized Controlled Trial. *Journal of stroke and cerebrovascular diseases : the official journal of National Stroke Association*, *28*(2), 477–486. <https://doi.org/10.1016/j.jstrokecerebrovasdis.2018.10.025>（The reason for exclusion: Non robot-assisted task-oriented training）
114. Jamin, P., Duret, C., Hutin, E., Bayle, N., Koeppel, T., Gracies, J. M., & Pila, O. (2022). Using Robot-Based Variables during Upper Limb Robot-Assisted Training in Subacute Stroke Patients to Quantify Treatment Dose. *Sensors (Basel, Switzerland)*, *22*(8), 2989. <https://doi.org/10.3390/s22082989>（The reason for exclusion: Non robot-assisted task-oriented training）
115. Iwamoto, Y., Tanaka, R., Imura, T., Mitsutake, T., Jung, H., Suzukawa, T., Taki, S., Imada, N., Inagawa, T., Araki, H., & Araki, O. (2023). Does frequent use of an exoskeletal upper limb robot improve motor function in stroke patients?. *Disability and rehabilitation*, *45*(7), 1185–1191. <https://doi.org/10.1080/09638288.2022.2055163>（The reason for exclusion: Non robot-assisted task-oriented training）
116. Chinembiri, B., Ming, Z., Kai, S., Xiu Fang, Z., & Wei, C. (2021). The fourier M2 robotic machine combined with occupational therapy on post-stroke upper limb function and independence-related quality of life: A randomized clinical trial. *Topics in stroke rehabilitation*, *28*(1), 1–18. <https://doi.org/10.1080/10749357.2020.1755815>（The reason for exclusion: Non robot-assisted task-oriented training）
117. Rosati G. (2010). The place of robotics in post-stroke rehabilitation. *Expert review of medical devices*, *7*(6), 753–758. <https://doi.org/10.1586/erd.10.49>（The reason for exclusion: Non robot-assisted task-oriented training）
118. Li, D. X., Zha, F. B., Long, J. J., Liu, F., Cao, J., & Wang, Y. L. (2021). Effect of Robot Assisted Gait Training on Motor and Walking Function in Patients with Subacute Stroke: A Random Controlled Study. *Journal of stroke and cerebrovascular diseases : the official journal of National Stroke Association*, *30*(7), 105807. <https://doi.org/10.1016/j.jstrokecerebrovasdis.2021.105807>（The reason for exclusion: duplicate record）
119. Bourke, T. C., Lowrey, C. R., Dukelow, S. P., Bagg, S. D., Norman, K. E., & Scott, S. H. (2016). A robot-based behavioural task to quantify impairments in rapid motor decisions and actions after stroke. *Journal of neuroengineering and rehabilitation*, *13*(1), 91. <https://doi.org/10.1186/s12984-016-0201-2>（The reason for exclusion: duplicate record）
120. Su, T., Wang, M., Chen, Z., & Feng, L. (2024). Effect of Upper Robot-Assisted Training on Upper Limb Motor, Daily Life Activities, and Muscular Tone in Patients With Stroke: A Systematic Review and Meta-Analysis. *Brain and behavior*, *14*(11), e70117. <https://doi.org/10.1002/brb3.70117>（The reason for exclusion: review）
121. Baldan, F., Turolla, A., Rimini, D., Pregnolato, G., Maistrello, L., Agostini, M., & Jakob, I. (2021). Robot-assisted rehabilitation of hand function after stroke: Development of prediction models for reference to therapy. *Journal of electromyography and kinesiology : official journal of the International Society of Electrophysiological Kinesiology*, *57*, 102534. <https://doi.org/10.1016/j.jelekin.2021.102534>（The reason for exclusion: duplicate record）
122. Awad, L. N., Esquenazi, A., Francisco, G. E., Nolan, K. J., & Jayaraman, A. (2020). The ReWalk ReStore™ soft robotic exosuit: a multi-site clinical trial of the safety, reliability, and feasibility of exosuit-augmented post-stroke gait rehabilitation. *Journal of neuroengineering and rehabilitation*, *17*(1), 80. <https://doi.org/10.1186/s12984-020-00702-5>（The reason for exclusion: duplicate record）
123. Gasperina, S. D., Longatelli, V., Panzenbeck, M., Luciani, B., Morosini, A., Piantoni, A., Tropea, P., Braghin, F., Pedrocchi, A., & Gandolla, M. (2022). AGREE: an upper-limb robotic platform for personalized rehabilitation, concept and clinical study design. *IEEE ... International Conference on Rehabilitation Robotics : [proceedings]*, *2022*, 1–6. <https://doi.org/10.1109/ICORR55369.2022.9896569>（The reason for exclusion: duplicate record）
124. Krebs, H. I., Ladenheim, B., Hippolyte, C., Monterroso, L., & Mast, J. (2009). Robot-assisted task-specific training in cerebral palsy. *Developmental medicine and child neurology*, *51 Suppl 4*, 140–145. <https://doi.org/10.1111/j.1469-8749.2009.03416.x>（The reason for exclusion: duplicate record）
125. Pila, O., Duret, C., Koeppel, T., & Jamin, P. (2023). Performance-Based Robotic Training in Individuals with Subacute Stroke: Differences between Responders and Non-Responders. *Sensors (Basel, Switzerland)*, *23*(9), 4304. <https://doi.org/10.3390/s23094304>（The reason for exclusion: duplicate record）
126. Nedergård, H., Sandlund, M., Häger, C. K., & Palmcrantz, S. (2023). Users' experiences of intensive robotic-assisted gait training post-stroke - "a push forward or feeling pushed around?". *Disability and rehabilitation*, *45*(23), 3861–3868. <https://doi.org/10.1080/09638288.2022.2140848>（The reason for exclusion: duplicate record）
127. Kutner, N. G., Zhang, R., Butler, A. J., Wolf, S. L., & Alberts, J. L. (2010). Quality-of-life change associated with robotic-assisted therapy to improve hand motor function in patients with subacute stroke: a randomized clinical trial. *Physical therapy*, *90*(4), 493–504. https://doi.org/10.2522/ptj.20090160（The reason for exclusion: duplicate record）
128. Tarkka, I. M., Pitkänen, K., Popovic, D. B., Vanninen, R., & Könönen, M. (2011). Functional electrical therapy for hemiparesis alleviates disability and enhances neuroplasticity. *The Tohoku journal of experimental medicine*, *225*(1), 71–76. https://doi.org/10.1620/tjem.225.71（The reason for exclusion: Non robot-assisted task-oriented training）
129. Germanotta, M., Gower, V., Papadopoulou, D., Cruciani, A., Pecchioli, C., Mosca, R., Speranza, G., Falsini, C., Cecchi, F., Vannetti, F., Montesano, A., Galeri, S., Gramatica, F., Aprile, I., & FDG Robotic Rehabilitation Group (2020). Reliability, validity and discriminant ability of a robotic device for finger training in patients with subacute stroke. *Journal of neuroengineering and rehabilitation*, *17*(1), 1. https://doi.org/10.1186/s12984-019-0634-5（The reason for exclusion: Irrelevant study outcome）
130. Silva, F. C., da Silva, R. V. T., Meireles, S. M., Fernandes, A. D. R. C., & Natour, J. (2024). Daytime Functional Usage Versus Night-Time Wearing: Identifying the Optimal Wearing Regimen for a Custom-Made Orthosis in the Treatment of Trapeziometacarpal Osteoarthritis. *Archives of physical medicine and rehabilitation*, *105*(10), 1837–1845. https://doi.org/10.1016/j.apmr.2024.06.013（The reason for exclusion: Non robot-assisted task-oriented training）
131. Carey, J. R., Durfee, W. K., Bhatt, E., Nagpal, A., Weinstein, S. A., Anderson, K. M., & Lewis, S. M. (2007). Comparison of finger tracking versus simple movement training via telerehabilitation to alter hand function and cortical reorganization after stroke. *Neurorehabilitation and neural repair*, *21*(3), 216–232. <https://doi.org/10.1177/1545968306292381>（The reason for exclusion: Non robot-assisted task-oriented training）
132. van den Noort, J. C., Verhagen, R., van Dijk, K. J., Veltink, P. H., Vos, M. C. P. M., de Bie, R. M. A., Bour, L. J., & Heida, C. T. (2017). Quantification of Hand Motor Symptoms in Parkinson's Disease: A Proof-of-Principle Study Using Inertial and Force Sensors. *Annals of biomedical engineering*, *45*(10), 2423–2436. <https://doi.org/10.1007/s10439-017-1881-x>（The reason for exclusion: Non robot-assisted task-oriented training）
133. Wei, Y., Chen, J., Fang, R., Liu, J., Feng, M., Du, H., Wang, M., Abulihaiti, R., Ling, H., & Huang, F. (2024). Investigating the Effect of Different Types of Exercise on Upper Limb Functional Recovery in Patients with Right Hemisphere Damage Based on fNIRS. *Journal of visualized experiments : JoVE*, (204), 10.3791/65996. <https://doi.org/10.3791/65996>（The reason for exclusion: Non robot-assisted task-oriented training）
134. Kilbreath, S. L., Crosbie, J., Canning, C. G., & Lee, M. J. (2006). Inter-limb coordination in bimanual reach-to-grasp following stroke. *Disability and rehabilitation*, *28*(23), 1435–1443. （The reason for exclusion: Non robot-assisted task-oriented training）https://doi.org/10.1080/09638280600638307（The reason for exclusion: Non robot-assisted task-oriented training）
135. Hesse, S., Kuhlmann, H., Wilk, J., Tomelleri, C., & Kirker, S. G. (2008). A new electromechanical trainer for sensorimotor rehabilitation of paralysed fingers: a case series in chronic and acute stroke patients. *Journal of neuroengineering and rehabilitation*, *5*, 21. https://doi.org/10.1186/1743-0003-5-21（The reason for exclusion: duplicate record）
136. Carey, J. R., Kimberley, T. J., Lewis, S. M., Auerbach, E. J., Dorsey, L., Rundquist, P., & Ugurbil, K. (2002). Analysis of fMRI and finger tracking training in subjects with chronic stroke. *Brain : a journal of neurology*, *125*(Pt 4), 773–788. <https://doi.org/10.1093/brain/awf091>（The reason for exclusion: Non robot-assisted task-oriented training）
137. Muller, C. O., Metais, A., Boublay, N., Breuil, C., Daligault, S., Di Rienzo, F., Guillot, A., Collet, C., Krolak-Salmon, P., & Saimpont, A. (2024). Anodal transcranial direct current stimulation does not enhance the effects of motor imagery training of a sequential finger-tapping task in young adults. *Journal of sports sciences*, *42*(5), 392–403. <https://doi.org/10.1080/02640414.2024.2328418>（The reason for exclusion: Non robot-assisted task-oriented training）
138. Bhatt, E., Nagpal, A., Greer, K. H., Grunewald, T. K., Steele, J. L., Wiemiller, J. W., Lewis, S. M., & Carey, J. R. (2007). Effect of finger tracking combined with electrical stimulation on brain reorganization and hand function in subjects with stroke. *Experimental brain research*, *182*(4), 435–447. <https://doi.org/10.1007/s00221-007-1001-5>（The reason for exclusion: Non robot-assisted task-oriented training）
139. Fischer, H. C., Stubblefield, K., Kline, T., Luo, X., Kenyon, R. V., & Kamper, D. G. (2007). Hand rehabilitation following stroke: a pilot study of assisted finger extension training in a virtual environment. *Topics in stroke rehabilitation*, *14*(1), 1–12. https://doi.org/10.1310/tsr1401-1（The reason for exclusion: duplicate record）
140. Jahangir, A. W., Tan, H. J., Norlinah, M. I., Nafisah, W. Y., Ramesh, S., Hamidon, B. B., & Raymond, A. A. (2007). Intramuscular injection of botulinum toxin for the treatment of wrist and finger spasticity after stroke. *The Medical journal of Malaysia*, *62*(4), 319–322.（The reason for exclusion: Non robot-assisted task-oriented training）
141. Cauraugh, J., Light, K., Kim, S., Thigpen, M., & Behrman, A. (2000). Chronic motor dysfunction after stroke: recovering wrist and finger extension by electromyography-triggered neuromuscular stimulation. *Stroke*, *31*(6), 1360–1364. <https://doi.org/10.1161/01.str.31.6.1360>（The reason for exclusion: Non robot-assisted task-oriented training）
142. Kim, D. G., Cho, Y. W., Hong, J. H., Song, J. C., Chung, H. A., Bai, D. S., Lee, C. H., & Jang, S. H. (2008). Effect of constraint-induced movement therapy with modified opposition restriction orthosis in chronic hemiparetic patients with stroke. *NeuroRehabilitation*, *23*(3), 239–244.（The reason for exclusion: duplicate record）
143. Seniów, J., Bilik, M., Leśniak, M., Waldowski, K., Iwański, S., & Członkowska, A. (2012). Transcranial magnetic stimulation combined with physiotherapy in rehabilitation of poststroke hemiparesis: a randomized, double-blind, placebo-controlled study. *Neurorehabilitation and neural repair*, *26*(9), 1072–1079. <https://doi.org/10.1177/1545968312445635>（The reason for exclusion: duplicate record）
144. Thielbar, K. O., Lord, T. J., Fischer, H. C., Lazzaro, E. C., Barth, K. C., Stoykov, M. E., Triandafilou, K. M., & Kamper, D. G. (2014). Training finger individuation with a mechatronic-virtual reality system leads to improved fine motor control post-stroke. *Journal of neuroengineering and rehabilitation*, *11*, 171. <https://doi.org/10.1186/1743-0003-11-171>（The reason for exclusion: duplicate record）
145. Yang, X., Shi, X., Xue, X., & Deng, Z. (2023). Efficacy of Robot-Assisted Training on Rehabilitation of Upper Limb Function in Patients With Stroke: A Systematic Review and Meta-analysis. *Archives of physical medicine and rehabilitation*, *104*(9), 1498–1513. <https://doi.org/10.1016/j.apmr.2023.02.004>（The reason for exclusion: review）
146. Iwamoto, Y., Imura, T., Suzukawa, T., Fukuyama, H., Ishii, T., Taki, S., Imada, N., Shibukawa, M., Inagawa, T., Araki, H., & Araki, O. (2019). Combination of Exoskeletal Upper Limb Robot and Occupational Therapy Improve Activities of Daily Living Function in Acute Stroke Patients. *Journal of stroke and cerebrovascular diseases : the official journal of National Stroke Association*, *28*(7), 2018–2025. <https://doi.org/10.1016/j.jstrokecerebrovasdis.2019.03.006>（The reason for exclusion: duplicate record）
147. Akıncı, M., Burak, M., Yaşar, E., & Kılıç, R. T. (2023). The effects of Robot-assisted gait training and virtual reality on balance and gait in stroke survivors: A randomized controlled trial. *Gait & posture*, *103*, 215–222. <https://doi.org/10.1016/j.gaitpost.2023.05.013>（The reason for exclusion: duplicate record）
148. Wong, Y., Li, C. J., Ada, L., Zhang, T., Månum, G., & Langhammer, B. (2022). Upper Limb Training with a Dynamic Hand Orthosis in Early Subacute Stroke: A Pilot Randomized Trial. *Journal of rehabilitation medicine*, *54*, jrm00279. https://doi.org/10.2340/jrm.v54.2231（The reason for exclusion: Non robot-assisted task-oriented training）
149. Bian, L., Zhang, L., Huang, G., Song, D., Zheng, K., Xu, X., Dai, W., Ren, C., & Shen, Y. (2024). Effects of Priming Intermittent Theta Burst Stimulation With High-Definition tDCS on Upper Limb Function in Hemiparetic Patients With Stroke: A Randomized Controlled Study. *Neurorehabilitation and neural repair*, *38*(4), 268–278. https://doi.org/10.1177/15459683241233259（The reason for exclusion: Non robot-assisted task-oriented training）
150. Chen, Z. J., He, C., Guo, F., Xiong, C. H., & Huang, X. L. (2021). Exoskeleton-Assisted Anthropomorphic Movement Training (EAMT) for Poststroke Upper Limb Rehabilitation: A Pilot Randomized Controlled Trial. *Archives of physical medicine and rehabilitation*, *102*(11), 2074–2082. https://doi.org/10.1016/j.apmr.2021.06.001（The reason for exclusion: duplicate record）
151. Feng, T., Zhao, C., Dong, J., Xue, Z., Cai, F., Li, X., Hu, Z., & Xue, X. (2024). The effect of unaffected side resistance training on upper limb function reconstruction and prevention of sarcopenia in stroke patients: a randomized controlled trial. *Scientific reports*, *14*(1), 25330. https://doi.org/10.1038/s41598-024-76810-2（The reason for exclusion: duplicate record）
152. Wang, L., Wang, S., Zhang, S., Dou, Z., & Guo, T. (2023). Effectiveness and electrophysiological mechanisms of focal vibration on upper limb motor dysfunction in patients with subacute stroke: A randomized controlled trial. *Brain research*, *1809*, 148353. https://doi.org/10.1016/j.brainres.2023.148353（The reason for exclusion: duplicate record）
153. Akinci, M., Burak, M., Kasal, F. Z., Özaslan, E. A., Huri, M., & Kurtaran, Z. A. (2024). The Effects of Combined Virtual Reality Exercises and Robot Assisted Gait Training on Cognitive Functions, Daily Living Activities, and Quality of Life in High Functioning Individuals With Subacute Stroke. *Perceptual and motor skills*, *131*(3), 756–769. <https://doi.org/10.1177/00315125241235420>（The reason for exclusion: duplicate record）
154. Valero-Cuevas, F. J., Klamroth-Marganska, V., Winstein, C. J., & Riener, R. (2016). Robot-assisted and conventional therapies produce distinct rehabilitative trends in stroke survivors. *Journal of neuroengineering and rehabilitation*, *13*(1), 92. <https://doi.org/10.1186/s12984-016-0199-5>（The reason for exclusion: duplicate record）
155. Liang, S., Hong, Z. Q., Cai, Q., Gao, H. G., Ren, Y. J., Zheng, H. Q., Chen, X., & Hu, X. Q. (2024). Effects of robot-assisted gait training on motor performance of lower limb in poststroke survivors: a systematic review with meta-analysis. *European review for medical and pharmacological sciences*, *28*(3), 879–898. <https://doi.org/10.26355/eurrev_202402_35325>（The reason for exclusion: review）
156. Ghasemi, A., Sadedel, M., & Moghaddam, M. M. (2024). A wearable system to assist impaired-neck patients: Design and evaluation. *Proceedings of the Institution of Mechanical Engineers. Part H, Journal of engineering in medicine*, *238*(1), 63–77. <https://doi.org/10.1177/09544119231211362>（The reason for exclusion: duplicate record）
157. Cindy J H, R., Prange-Lasonder, G. B., Prinsen, E. C., Buurke, J. H., & Rietman, J. S. (2022). Detection thresholds for electrostimulation combined with robotic leg support in sub-acute stroke patients. *IEEE ... International Conference on Rehabilitation Robotics : [proceedings]*, *2022*, 1–5. <https://doi.org/10.1109/ICORR55369.2022.9896576>（The reason for exclusion: duplicate record）
158. Schicketmueller, A., Lamprecht, J., Hofmann, M., Sailer, M., & Rose, G. (2020). Gait Event Detection for Stroke Patients during Robot-Assisted Gait Training. *Sensors (Basel, Switzerland)*, *20*(12), 3399. <https://doi.org/10.3390/s20123399>（The reason for exclusion: duplicate record）
159. Villafañe JH, Taveggia G, Galeri S, et al. Efficacy of Short-Term Robot-Assisted Rehabilitation in Patients With Hand Paralysis After Stroke: A Randomized Clinical Trial. Hand (N Y). 2018;13(1):95-102. doi:10.1177/1558944717692096 （The reason for exclusion: duplicate record）
160. Du J, Wang S, Cheng Y, et al. Effects of Neuromuscular Electrical Stimulation Combined with Repetitive Transcranial Magnetic Stimulation on Upper Limb Motor Function Rehabilitation in Stroke Patients with Hemiplegia [retracted in: Comput Math Methods Med. 2023 Nov 1;2023:9767295. doi: 10.1155/2023/9767295]. Comput Math Methods Med. 2022;2022:9455428. Published 2022 Jan 4. doi:10.1155/2022/9455428 （The reason for exclusion: Non robot-assisted task-oriented training）
161. Huang YC, Chen PC, Tso HH, Yang YC, Ho TL, Leong CP. Effects of kinesio taping on hemiplegic hand in patients with upper limb post-stroke spasticity: a randomized controlled pilot study. Eur J Phys Rehabil Med. 2019;55(5):551-557. doi:10.23736/S1973-9087.19.05684-3 （The reason for exclusion: Non robot-assisted task-oriented training）
162. Kirac-Unal, Z., Gencay-Can, A., Karaca-Umay, E., & Cakci, F. A. (2019). The effect of task-oriented electromyography-triggered electrical stimulation of the paretic wrist extensors on upper limb motor function early after stroke: a pilot randomized controlled trial. *International journal of rehabilitation research. Internationale Zeitschrift fur Rehabilitationsforschung. Revue internationale de recherches de readaptation*, *42*(1), 74–81. https://doi.org/10.1097/MRR.0000000000000333（The reason for exclusion: Irrelevant study outcome）
163. Chiu, H. C., Ada, L., & Lee, H. M. (2014). Upper limb training using Wii Sports Resort for children with hemiplegic cerebral palsy: a randomized, single-blind trial. *Clinical rehabilitation*, *28*(10), 1015–1024. https://doi.org/10.1177/0269215514533709（The reason for exclusion: Non robot-assisted task-oriented training）
164. Chae, J., Harley, M. Y., Hisel, T. Z., Corrigan, C. M., Demchak, J. A., Wong, Y. T., & Fang, Z. P. (2009). Intramuscular electrical stimulation for upper limb recovery in chronic hemiparesis: an exploratory randomized clinical trial. *Neurorehabilitation and neural repair*, *23*(6), 569–578. https://doi.org/10.1177/1545968308328729（The reason for exclusion: Non robot-assisted task-oriented training）
165. Nijland, R., van Wegen, E., van der Krogt, H., Bakker, C., Buma, F., Klomp, A., van Kordelaar, J., Kwakkel, G., & EXPLICIT-stroke consortium (2013). Characterizing the protocol for early modified constraint-induced movement therapy in the EXPLICIT-stroke trial. *Physiotherapy research international : the journal for researchers and clinicians in physical therapy*, *18*(1), 1–15. https://doi.org/10.1002/pri.1521（The reason for exclusion: Non robot-assisted task-oriented training）
166. Kirac-Unal, Z., Gencay-Can, A., Karaca-Umay, E., & Cakci, F. A. (2019). The effect of task-oriented electromyography-triggered electrical stimulation of the paretic wrist extensors on upper limb motor function early after stroke: a pilot randomized controlled trial. *International journal of rehabilitation research. Internationale Zeitschrift fur Rehabilitationsforschung. Revue internationale de recherches de readaptation*, *42*(1), 74–81. https://doi.org/10.1097/MRR.0000000000000333（The reason for exclusion: Irrelevant study outcome）
167. Chiu, H. C., Ada, L., & Lee, H. M. (2014). Upper limb training using Wii Sports Resort for children with hemiplegic cerebral palsy: a randomized, single-blind trial. *Clinical rehabilitation*, *28*(10), 1015–1024. https://doi.org/10.1177/0269215514533709（The reason for exclusion: Non robot-assisted task-oriented training）
168. Chae, J., Harley, M. Y., Hisel, T. Z., Corrigan, C. M., Demchak, J. A., Wong, Y. T., & Fang, Z. P. (2009). Intramuscular electrical stimulation for upper limb recovery in chronic hemiparesis: an exploratory randomized clinical trial. *Neurorehabilitation and neural repair*, *23*(6), 569–578. https://doi.org/10.1177/1545968308328729（The reason for exclusion: Non robot-assisted task-oriented training）
169. Lannin, N. A., Horsley, S. A., Herbert, R., McCluskey, A., & Cusick, A. (2003). Splinting the hand in the functional position after brain impairment: a randomized, controlled trial. *Archives of physical medicine and rehabilitation*, *84*(2), 297–302. https://doi.org/10.1053/apmr.2003.50031（The reason for exclusion: Non robot-assisted task-oriented training）
170. Cazenave, L., Yurkewich, A., Hohler, C., Keller, T., Krewer, C., Jahn, K., Hirche, S., Endo, S., & Burdet, E. (2023). Hybrid Functional Electrical Stimulation and Robotic Assistance for Wrist Motion Training After Stroke: Preliminary Results. *IEEE ... International Conference on Rehabilitation Robotics : [proceedings]*, *2023*, 1–6. <https://doi.org/10.1109/ICORR58425.2023.10304736>（The reason for exclusion: duplicate record）
171. Vanoglio, F., Bernocchi, P., Mulè, C., Garofali, F., Mora, C., Taveggia, G., Scalvini, S., & Luisa, A. (2017). Feasibility and efficacy of a robotic device for hand rehabilitation in hemiplegic stroke patients: a randomized pilot controlled study. *Clinical rehabilitation*, *31*(3), 351–360. <https://doi.org/10.1177/0269215516642606>（The reason for exclusion: duplicate record）
172. Trujillo, P., Mastropietro, A., Scano, A., Chiavenna, A., Mrakic-Sposta, S., Caimmi, M., Molteni, F., & Rizzo, G. (2017). Quantitative EEG for Predicting Upper Limb Motor Recovery in Chronic Stroke Robot-Assisted Rehabilitation. *IEEE transactions on neural systems and rehabilitation engineering : a publication of the IEEE Engineering in Medicine and Biology Society*, *25*(7), 1058–1067. <https://doi.org/10.1109/TNSRE.2017.2678161>（The reason for exclusion: Non robot-assisted task-oriented training）
173. Huang, X., Naghdy, F., Naghdy, G., Du, H., & Todd, C. (2018). The Combined Effects of Adaptive Control and Virtual Reality on Robot-Assisted Fine Hand Motion Rehabilitation in Chronic Stroke Patients: A Case Study. *Journal of stroke and cerebrovascular diseases : the official journal of National Stroke Association*, *27*(1), 221–228. <https://doi.org/10.1016/j.jstrokecerebrovasdis.2017.08.027>（The reason for exclusion: Non robot-assisted task-oriented training）
174. Nankaku, M., Tanaka, H., Ikeguchi, R., Kikuchi, T., Miyamoto, S., & Matsuda, S. (2020). Effects of walking distance over robot-assisted training on walking ability in chronic stroke patients. *Journal of clinical neuroscience : official journal of the Neurosurgical Society of Australasia*, *81*, 279–283. <https://doi.org/10.1016/j.jocn.2020.09.067>（The reason for exclusion: duplicate record）
175. Christopher, S. M., & Johnson, M. J. (2014). Task-oriented robot-assisted stroke therapy of paretic limb improves control in a unilateral and bilateral functional drink task: a case study. *Annual International Conference of the IEEE Engineering in Medicine and Biology Society. IEEE Engineering in Medicine and Biology Society. Annual International Conference*, *2014*, 1194–1197. <https://doi.org/10.1109/EMBC.2014.6943810>（The reason for exclusion: Non robot-assisted task-oriented training）
176. Colombo, R., Pisano, F., Micera, S., Mazzone, A., Delconte, C., Carrozza, M. C., Dario, P., & Minuco, G. (2005). Robotic techniques for upper limb evaluation and rehabilitation of stroke patients. *IEEE transactions on neural systems and rehabilitation engineering : a publication of the IEEE Engineering in Medicine and Biology Society*, *13*(3), 311–324. <https://doi.org/10.1109/TNSRE.2005.848352>（The reason for exclusion: Non robot-assisted task-oriented training）
177. Noronha, B., Ng, C. Y., Little, K., Xiloyannis, M., Kuah, C. W. K., Wee, S. K., Kulkarni, S. R., Masia, L., Chua, K. S. G., & Accoto, D. (2022). Soft, Lightweight Wearable Robots to Support the Upper Limb in Activities of Daily Living: A Feasibility Study on Chronic Stroke Patients. *IEEE transactions on neural systems and rehabilitation engineering : a publication of the IEEE Engineering in Medicine and Biology Society*, *30*, 1401–1411. <https://doi.org/10.1109/TNSRE.2022.3175224>（The reason for exclusion: duplicate record）
178. Pignolo, L., Servidio, R., Basta, G., Carozzo, S., Tonin, P., Calabrò, R. S., & Cerasa, A. (2021). The Route of Motor Recovery in Stroke Patients Driven by Exoskeleton-Robot-Assisted Therapy: A Path-Analysis. *Medical sciences (Basel, Switzerland)*, *9*(4), 64. <https://doi.org/10.3390/medsci9040064>（The reason for exclusion: Non robot-assisted task-oriented training）
179. Zhou, Z. Q., Hua, X. Y., Wu, J. J., Xu, J. J., Ren, M., Shan, C. L., & Xu, J. G. (2022). Combined robot motor assistance with neural circuit-based virtual reality (NeuCir-VR) lower extremity rehabilitation training in patients after stroke: a study protocol for a single-centre randomised controlled trial. *BMJ open*, *12*(12), e064926. <https://doi.org/10.1136/bmjopen-2022-064926>（The reason for exclusion: Non robot-assisted task-oriented training）
180. Aisen, M. L., Krebs, H. I., Hogan, N., McDowell, F., & Volpe, B. T. (1997). The effect of robot-assisted therapy and rehabilitative training on motor recovery following stroke. *Archives of neurology*, *54*(4), 443–446. <https://doi.org/10.1001/archneur.1997.00550160075019>（The reason for exclusion: Non robot-assisted task-oriented training）
181. Hsieh, Y. W., Wu, C. Y., Wang, W. E., Lin, K. C., Chang, K. C., Chen, C. C., & Liu, C. T. (2017). Bilateral robotic priming before task-oriented approach in subacute stroke rehabilitation: a pilot randomized controlled trial. *Clinical rehabilitation*, *31*(2), 225–233. <https://doi.org/10.1177/0269215516633275>(This document meets the inclusion criteria)
182. Nakipoğlu Yuzer, G. F., Köse Dönmez, B., & Özgirgin, N. (2017). A Randomized Controlled Study: Effectiveness of Functional Electrical Stimulation on Wrist and Finger Flexor Spasticity in Hemiplegia. *Journal of stroke and cerebrovascular diseases : the official journal of National Stroke Association*, *26*(7), 1467–1471. https://doi.org/10.1016/j.jstrokecerebrovasdis.2017.03.011（The reason for exclusion: Non robot-assisted task-oriented training）
183. Renner, C. I. E., Brendel, C., & Hummelsheim, H. (2020). Bilateral Arm Training vs Unilateral Arm Training for Severely Affected Patients With Stroke: Exploratory Single-Blinded Randomized Controlled Trial. *Archives of physical medicine and rehabilitation*, *101*(7), 1120–1130. https://doi.org/10.1016/j.apmr.2020.02.007（The reason for exclusion: Non robot-assisted task-oriented training）
184. Cordo, P., Wolf, S., Lou, J. S., Bogey, R., Stevenson, M., Hayes, J., & Roth, E. (2013). Treatment of severe hand impairment following stroke by combining assisted movement, muscle vibration, and biofeedback. *Journal of neurologic physical therapy : JNPT*, *37*(4), 194–203. https://doi.org/10.1097/NPT.0000000000000023（The reason for exclusion: Non robot-assisted task-oriented training）
185. Sgandurra, G., Ferrari, A., Cossu, G., Guzzetta, A., Fogassi, L., & Cioni, G. (2013). Randomized trial of observation and execution of upper extremity actions versus action alone in children with unilateral cerebral palsy. *Neurorehabilitation and neural repair*, *27*(9), 808–815. https://doi.org/10.1177/1545968313497101（The reason for exclusion: Non robot-assisted task-oriented training）
186. Hsu, H. Y., Kuan, T. S., Tsai, C. L., Wu, P. T., Kuo, Y. L., Su, F. C., & Kuo, L. C. (2021). Effect of a Novel Perturbation-Based Pinch Task Training on Sensorimotor Performance of Upper Extremity for Patients With Chronic Stroke: A Pilot Randomized Controlled Trial. *Archives of physical medicine and rehabilitation*, *102*(5), 811–818. https://doi.org/10.1016/j.apmr.2020.11.004（The reason for exclusion: Irrelevant study outcome）
187. Calabrò, R. S., Accorinti, M., Porcari, B., Carioti, L., Ciatto, L., Billeri, L., Andronaco, V. A., Galletti, F., Filoni, S., & Naro, A. (2019). Does hand robotic rehabilitation improve motor function by rebalancing interhemispheric connectivity after chronic stroke? Encouraging data from a randomised-clinical-trial. *Clinical neurophysiology : official journal of the International Federation of Clinical Neurophysiology*, *130*(5), 767–780. https://doi.org/10.1016/j.clinph.2019.02.013（The reason for exclusion: Non robot-assisted task-oriented training）
188. Kwakkel, G., Winters, C., van Wegen, E. E., Nijland, R. H., van Kuijk, A. A., Visser-Meily, A., de Groot, J., de Vlugt, E., Arendzen, J. H., Geurts, A. C., Meskers, C. G., & EXPLICIT-Stroke Consortium (2016). Effects of Unilateral Upper Limb Training in Two Distinct Prognostic Groups Early After Stroke: The EXPLICIT-Stroke Randomized Clinical Trial. *Neurorehabilitation and neural repair*, *30*(9), 804–816. https://doi.org/10.1177/1545968315624784（The reason for exclusion: Non robot-assisted task-oriented training）
189. Berczeli, M., Chinnadurai, P., Legeza, P. T., Britz, G. W., & Lumsden, A. B. (2022). Transcarotid access for remote robotic endovascular neurointerventions: a cadaveric proof-of-concept study. *Neurosurgical focus*, *52*(1), E18. <https://doi.org/10.3171/2021.10.FOCUS21511>（The reason for exclusion: Non-RCTs）
190. Wade, E., & Winstein, C. J. (2011). Virtual reality and robotics for stroke rehabilitation: where do we go from here?. *Topics in stroke rehabilitation*, *18*(6), 685–700. <https://doi.org/10.1310/tsr1806-685>（The reason for exclusion: Non-RCTs）
191. Yokota, C., Tanaka, K., Omae, K., Kamada, M., Nishikawa, H., Koga, M., Ihara, M., Fujimoto, Y., Sankai, Y., Nakajima, T., & Minami, M. (2023). Effect of cyborg-type robot Hybrid Assistive Limb on patients with severe walking disability in acute stroke: A randomized controlled study. *Journal of stroke and cerebrovascular diseases : the official journal of National Stroke Association*, *32*(4), 107020. <https://doi.org/10.1016/j.jstrokecerebrovasdis.2023.107020>（The reason for exclusion: duplicate record）
192. Elangovan, N., Yeh, I. L., Holst-Wolf, J., & Konczak, J. (2019). A robot-assisted sensorimotor training program can improve proprioception and motor function in stroke survivors. *IEEE ... International Conference on Rehabilitation Robotics : [proceedings]*, *2019*, 660–664. https://doi.org/10.1109/ICORR.2019.8779409（The reason for exclusion: meeting abstract）
193. Yokota, C., Tanaka, K., Omae, K., Kamada, M., Nishikawa, H., Koga, M., Ihara, M., Fujimoto, Y., Sankai, Y., Nakajima, T., & Minami, M. (2023). Effect of cyborg-type robot Hybrid Assistive Limb on patients with severe walking disability in acute stroke: A randomized controlled study. *Journal of stroke and cerebrovascular diseases : the official journal of National Stroke Association*, *32*(4), 107020.
194. Flynn, N., Froude, E., Cooke, D., Dennis, J., & Kuys, S. (2022). The sustainability of upper limb robotic therapy for stroke survivors in an inpatient rehabilitation setting. *Disability and rehabilitation*, *44*(24), 7522–7527. <https://doi.org/10.1080/09638288.2021.1998664>（The reason for exclusion: duplicate record）
195. Sharma, A., Kumari, S., & Saxena, A. (2023). Commentary on "Comparative Effectiveness of Robot-Assisted Training Versus Enhanced Upper Extremity Therapy on Upper and Lower Extremity for Stroke Survivors: A Multicentre Randomized Controlled Trial". *Journal of rehabilitation medicine*, *55*, jrm5588. <https://doi.org/10.2340/jrm.v55.5588>（The reason for exclusion: duplicate record）
196. Raghavan, P., Bilaloglu, S., Ali, S. Z., Jin, X., Aluru, V., Buckley, M. C., Tang, A., Yousefi, A., Stone, J., Agrawal, S. K., & Lu, Y. (2020). The Role of Robotic Path Assistance and Weight Support in Facilitating 3D Movements in Individuals With Poststroke Hemiparesis. *Neurorehabilitation and neural repair*, *34*(2), 134–147. <https://doi.org/10.1177/1545968319887685>（The reason for exclusion: duplicate record）
197. Masiero, S., Armani, M., & Rosati, G. (2011). Upper-limb robot-assisted therapy in rehabilitation of acute stroke patients: focused review and results of new randomized controlled trial. *Journal of rehabilitation research and development*, *48*(4), 355–366. <https://doi.org/10.1682/jrrd.2010.04.0063>（The reason for exclusion: duplicate record）
198. Chen, L., Chen, Y., Fu, W. B., Huang, D. F., & Lo, W. L. A. (2022). The Effect of Virtual Reality on Motor Anticipation and Hand Function in Patients with Subacute Stroke: A Randomized Trial on Movement-Related Potential. *Neural plasticity*, *2022*, 7399995. https://doi.org/10.1155/2022/7399995（The reason for exclusion: Non robot-assisted task-oriented training）
199. Hesse, S., Tomelleri, C., Bardeleben, A., Werner, C., & Waldner, A. (2012). Robot-assisted practice of gait and stair climbing in nonambulatory stroke patients. *Journal of rehabilitation research and development*, *49*(4), 613–622. <https://doi.org/10.1682/jrrd.2011.08.0142>（The reason for exclusion: duplicate record）
200. Mazzoleni, S., Focacci, A., Franceschini, M., Waldner, A., Spagnuolo, C., Battini, E., & Bonaiuti, D. (2017). Robot-assisted end-effector-based gait training in chronic stroke patients: A multicentric uncontrolled observational retrospective clinical study. *NeuroRehabilitation*, *40*(4), 483–492. <https://doi.org/10.3233/NRE-161435>（The reason for exclusion: duplicate record）
201. Klinkwan, P., Kongmaroeng, C., Muengtaweepongsa, S., & Limtrakarn, W. (2023). Prototype development of bilateral arm mirror-like-robotic rehabilitation device for acute stroke patients. *Biomedical physics & engineering express*, *9*(4), 10.1088/2057-1976/acd11d. <https://doi.org/10.1088/2057-1976/acd11d>（The reason for exclusion: Non robot-assisted task-oriented training）
202. Reis, S. B., Bernardo, W. M., Oshiro, C. A., Krebs, H. I., & Conforto, A. B. (2021). Effects of Robotic Therapy Associated With Noninvasive Brain Stimulation on Upper-Limb Rehabilitation After Stroke: Systematic Review and Meta-analysis of Randomized Clinical Trials. *Neurorehabilitation and neural repair*, *35*(3), 256–266. https://doi.org/10.1177/1545968321989353（The reason for exclusion: review）
203. Park, S., Fraser, M., Weber, L. M., Meeker, C., Bishop, L., Geller, D., Stein, J., & Ciocarlie, M. (2020). User-Driven Functional Movement Training With a Wearable Hand Robot After Stroke. *IEEE transactions on neural systems and rehabilitation engineering : a publication of the IEEE Engineering in Medicine and Biology Society*, *28*(10), 2265–2275. <https://doi.org/10.1109/TNSRE.2020.3021691>（The reason for exclusion: duplicate record）
204. Scano, A., Chiavenna, A., Caimmi, M., Malosio, M., Tosatti, L. M., & Molteni, F. (2017). Effect of human-robot interaction on muscular synergies on healthy people and post-stroke chronic patients. *IEEE ... International Conference on Rehabilitation Robotics : [proceedings]*, *2017*, 527–532. <https://doi.org/10.1109/ICORR.2017.8009302>（The reason for exclusion: duplicate record）
205. Lee, M. J., Lee, J. H., & Lee, S. M. (2018). Effects of robot-assisted therapy on upper extremity function and activities of daily living in hemiplegic patients: A single-blinded, randomized, controlled trial. *Technology and health care : official journal of the European Society for Engineering and Medicine*, *26*(4), 659–666. <https://doi.org/10.3233/THC-181336>（The reason for exclusion: duplicate record）
206. Hsu, H. Y., Yang, K. C., Yeh, C. H., Lin, Y. C., Lin, K. R., Su, F. C., & Kuo, L. C. (2022). A Tenodesis-Induced-Grip exoskeleton robot (TIGER) for assisting upper extremity functions in stroke patients: a randomized control study. *Disability and rehabilitation*, *44*(23), 7078–7086. <https://doi.org/10.1080/09638288.2021.1980915>（The reason for exclusion: duplicate record）
207. Ahmed, T., Islam, M. R., Brahmi, B., & Rahman, M. H. (2022). Robustness and Tracking Performance Evaluation of PID Motion Control of 7 DoF Anthropomorphic Exoskeleton Robot Assisted Upper Limb Rehabilitation. *Sensors (Basel, Switzerland)*, *22*(10), 3747. <https://doi.org/10.3390/s22103747>（The reason for exclusion: duplicate record）
208. Montedoro, V., Alsamour, M., Dehem, S., Lejeune, T., Dehez, B., & Edwards, M. G. (2019). Robot Diagnosis Test for Egocentric and Allocentric Hemineglect. *Archives of clinical neuropsychology : the official journal of the National Academy of Neuropsychologists*, *34*(4), 481–494. <https://doi.org/10.1093/arclin/acy062>（The reason for exclusion: duplicate record）
209. Hsu, H. Y., Koh, C. L., Yang, K. C., Lin, Y. C., Hsu, C. H., Su, F. C., & Kuo, L. C. (2024). Effects of an assist-as-needed equipped Tenodesis-Induced-Grip Exoskeleton Robot (TIGER) on upper limb function in patients with chronic stroke. *Journal of neuroengineering and rehabilitation*, *21*(1), 5. <https://doi.org/10.1186/s12984-023-01298-2>（The reason for exclusion: duplicate record）
210. Huo, C., Shao, G., Chen, T., Li, W., Wang, J., Xie, H., Wang, Y., Li, Z., Zheng, P., Li, L., & Li, L. (2024). Effectiveness of unilateral lower-limb exoskeleton robot on balance and gait recovery and neuroplasticity in patients with subacute stroke: a randomized controlled trial. *Journal of neuroengineering and rehabilitation*, *21*(1), 213. <https://doi.org/10.1186/s12984-024-01493-9>（The reason for exclusion: duplicate record）
211. Norouzi-Gheidari, N., Archambault, P. S., & Fung, J. (2019). Robot-Assisted Reaching Performance of Chronic Stroke and Healthy Individuals in a Virtual Versus a Physical Environment: A Pilot Study. *IEEE transactions on neural systems and rehabilitation engineering : a publication of the IEEE Engineering in Medicine and Biology Society*, *27*(6), 1273–1281. <https://doi.org/10.1109/TNSRE.2019.2914015>（The reason for exclusion: duplicate record）
212. Tedla, J. S., Dixit, S., Gular, K., & Abohashrh, M. (2019). Robotic-Assisted Gait Training Effect on Function and Gait Speed in Subacute and Chronic Stroke Population: A Systematic Review and Meta-Analysis of Randomized Controlled Trials. *European neurology*, *81*(3-4), 103–111. <https://doi.org/10.1159/000500747>（The reason for exclusion: review）
213. Mazzoleni, S., Sale, P., Tiboni, M., Franceschini, M., Carrozza, M. C., & Posteraro, F. (2013). Upper limb robot-assisted therapy in chronic and subacute stroke patients: a kinematic analysis. *American journal of physical medicine & rehabilitation*, *92*(10 Suppl 2), e26–e37. <https://doi.org/10.1097/PHM.0b013e3182a1e852>（The reason for exclusion: duplicate record）
214. Kim, E., Lee, G., Lee, J., & Kim, Y. H. (2024). Simultaneous high-definition transcranial direct current stimulation and robot-assisted gait training in stroke patients. *Scientific reports*, *14*(1), 4483. https://doi.org/10.1038/s41598-024-53482-6（The reason for exclusion: Control group using additional interventions)
215. Stockbridge, M. D., Bunker, L. D., & Hillis, A. E. (2022). Reversing the Ruin: Rehabilitation, Recovery, and Restoration After Stroke. *Current neurology and neuroscience reports*, *22*(11), 745–755. <https://doi.org/10.1007/s11910-022-01231-5>（The reason for exclusion: review）
216. Pinheiro, C., Figueiredo, J., Cerqueira, J., & Santos, C. P. (2022). Robotic Biofeedback for Post-Stroke Gait Rehabilitation: A Scoping Review. *Sensors (Basel, Switzerland)*, *22*(19), 7197. <https://doi.org/10.3390/s22197197>（The reason for exclusion: review）
217. Tseng, K. C., Wang, L., Hsieh, C., & Wong, A. M. (2024). Portable robots for upper-limb rehabilitation after stroke: a systematic review and meta-analysis. *Annals of medicine*, *56*(1), 2337735. <https://doi.org/10.1080/07853890.2024.2337735>（The reason for exclusion: review）
218. Singer, B. J., Vallence, A. M., Cleary, S., Cooper, I., & Loftus, A. M. (2013). The effect of EMG triggered electrical stimulation plus task practice on arm function in chronic stroke patients with moderate-severe arm deficits. *Restorative neurology and neuroscience*, *31*(6), 681–691. <https://doi.org/10.3233/RNN-130319>（The reason for exclusion: duplicate record）
219. Aguilera-Rubio, Á., Alguacil-Diego, I. M., Mallo-López, A., Jardón Huete, A., Oña, E. D., & Cuesta-Gómez, A. (2024). Use of low-cost virtual reality in the treatment of the upper extremity in chronic stroke: a randomized clinical trial. *Journal of neuroengineering and rehabilitation*, *21*(1), 12. <https://doi.org/10.1186/s12984-024-01303-2>（The reason for exclusion: duplicate record）
220. Tang, C., Zhou, T., Zhang, Y., Yuan, R., Zhao, X., Yin, R., Song, P., Liu, B., Song, R., Chen, W., & Wang, H. (2023). Bilateral upper limb robot-assisted rehabilitation improves upper limb motor function in stroke patients: a study based on quantitative EEG. *European journal of medical research*, *28*(1), 603. <https://doi.org/10.1186/s40001-023-01565-x>（The reason for exclusion: duplicate record）
221. Xia, X., Dong, X., Huo, H., Zhang, Y., Song, J., & Wang, D. (2023). Clinical study of low-frequency acupoint electrical stimulation to improve thumb-to-finger movements after stroke: A randomized controlled trial. *Medicine*, *102*(47), e35755. https://doi.org/10.1097/MD.0000000000035755（The reason for exclusion: Irrelevant study outcome）
222. Vanoglio, F., Comini, L., Gaiani, M., Bonometti, G. P., Luisa, A., & Bernocchi, P. (2024). A Sensor-Based Upper Limb Treatment in Hemiplegic Patients: Results from a Randomized Pilot Study. *Sensors (Basel, Switzerland)*, *24*(8), 2574. https://doi.org/10.3390/s24082574（The reason for exclusion: duplicate record）
223. Akgün, İ., Demirbüken, İ., Timurtaş, E., Pehlivan, M. K., Pehlivan, A. U., Polat, M. G., Francisco, G. E., & Yozbatiran, N. (2024). Exoskeleton-assisted upper limb rehabilitation after stroke: a randomized controlled trial. *Neurological research*, *46*(11), 1074–1082. https://doi.org/10.1080/01616412.2024.2381385（The reason for exclusion: duplicate record）
224. Bernal-Jiménez, J. J., Dileone, M., Mordillo-Mateos, L., Martín-Conty, J. L., Durantez-Fernández, C., Viñuela, A., Martín-Rodríguez, F., Lerin-Calvo, A., Alcántara-Porcuna, V., & Polonio-López, B. (2024). Combining Transcranial Direct Current Stimulation With Hand Robotic Rehabilitation in Chronic Stroke Patients: A Double-Blind Randomized Clinical Trial. *American journal of physical medicine & rehabilitation*, *103*(10), 875–882. https://doi.org/10.1097/PHM.0000000000002446（The reason for exclusion: duplicate record）
225. Li, Y., Lian, Y., Chen, X., Zhang, H., Xu, G., Duan, H., Xie, X., & Li, Z. (2024). Effect of task-oriented training assisted by force feedback hand rehabilitation robot on finger grasping function in stroke patients with hemiplegia: a randomised controlled trial. *Journal of neuroengineering and rehabilitation*, *21*(1), 77. https://doi.org/10.1186/s12984-024-01372-3（The reason for exclusion: duplicate record）
226. van Ravestyn, C., Gerardin, E., Térémetz, M., Hamdoun, S., Baron, J. C., Calvet, D., Vandermeeren, Y., Turc, G., Maier, M. A., Rosso, C., Mas, J. L., Dupin, L., & Lindberg, P. G. (2024). Post-Stroke Impairments of Manual Dexterity and Finger Proprioception: Their Contribution to Upper Limb Activity Capacity. *Neurorehabilitation and neural repair*, *38*(5), 373–385. https://doi.org/10.1177/15459683241245416（The reason for exclusion: Irrelevant study outcome）
227. Hung, J. W., Yen, C. L., Chang, K. C., Chiang, W. C., Chuang, I. C., Pong, Y. P., Wu, W. C., & Wu, C. Y. (2022). A Pilot Randomized Controlled Trial of Botulinum Toxin Treatment Combined with Robot-Assisted Therapy, Mirror Therapy, or Active Control Treatment in Patients with Spasticity Following Stroke. *Toxins*, *14*(6), 415. <https://doi.org/10.3390/toxins14060415>（The reason for exclusion: duplicate record）
228. Bui, K. D., Lyn, B., Roland, M., Wamsley, C. A., Mendonca, R., & Johnson, M. J. (2022). The Impact of Cognitive Impairment on Robot-Based Upper-Limb Motor Assessment in Chronic Stroke. *Neurorehabilitation and neural repair*, *36*(9), 587–595. <https://doi.org/10.1177/15459683221110892>（The reason for exclusion: duplicate record）
229. Costa, M., Tataryn, Z., Alobaid, A., Pierre, C., Basamh, M., Somji, M., Loh, Y., Patel, A., & Monteith, S. (2023). Robotically-assisted neuro-endovascular procedures: Single-Center Experience and a Review of the Literature. *Interventional neuroradiology : journal of peritherapeutic neuroradiology, surgical procedures and related neurosciences*, *29*(2), 201–210. <https://doi.org/10.1177/15910199221082475>（The reason for exclusion: review）
230. Fonte, C., Varalta, V., Rocco, A., Munari, D., Filippetti, M., Evangelista, E., Modenese, A., Smania, N., & Picelli, A. (2021). Combined transcranial Direct Current Stimulation and robot-assisted arm training in patients with stroke: a systematic review. *Restorative neurology and neuroscience*, *39*(6), 435–446. <https://doi.org/10.3233/RNN-211218>（The reason for exclusion: duplicate record）
231. Leem, M. J., Kim, G. S., Kim, K. H., Yi, T. I., & Moon, H. I. (2019). Predictors of functional and motor outcomes following upper limb robot-assisted therapy after stroke. *International journal of rehabilitation research. Internationale Zeitschrift fur Rehabilitationsforschung. Revue internationale de recherches de readaptation*, *42*(3), 223–228. <https://doi.org/10.1097/MRR.0000000000000349>（The reason for exclusion: duplicate record）
232. Pérez, P. J., Garcia-Zapirain, B., & Mendez-Zorrilla, A. (2015). Caregiver and social assistant robot for rehabilitation and coaching for the elderly. *Technology and health care : official journal of the European Society for Engineering and Medicine*, *23*(3), 351–357. <https://doi.org/10.3233/THC-150896>（The reason for exclusion: duplicate record）
233. Nizamis, K., Athanasiou, A., Almpani, S., Dimitrousis, C., & Astaras, A. (2021). Converging Robotic Technologies in Targeted Neural Rehabilitation: A Review of Emerging Solutions and Challenges. *Sensors (Basel, Switzerland)*, *21*(6), 2084. <https://doi.org/10.3390/s21062084>（The reason for exclusion: review）
234. Khalid, S., Alnajjar, F., Gochoo, M., Renawi, A., & Shimoda, S. (2023). Robotic assistive and rehabilitation devices leading to motor recovery in upper limb: a systematic review. *Disability and rehabilitation. Assistive technology*, *18*(5), 658–672. <https://doi.org/10.1080/17483107.2021.1906960>（The reason for exclusion: review）
235. Chen, S. C., Kang, J. H., Peng, C. W., Hsu, C. C., Lin, Y. N., & Lai, C. H. (2022). Adjustable Parameters and the Effectiveness of Adjunct Robot-Assisted Gait Training in Individuals with Chronic Stroke. *International journal of environmental research and public health*, *19*(13), 8186. <https://doi.org/10.3390/ijerph19138186>（The reason for exclusion: duplicate record）
236. Yang, X., Fengyi, W., Yi, C., Lin, Q., Yang, L., Xize, L., Shaxin, L., & Yonghong, Y. (2024). Effects of robot-assisted upper limb training combined with functional electrical stimulation in stroke patients: study protocol for a randomized controlled trial. *Trials*, *25*(1), 355. <https://doi.org/10.1186/s13063-024-08199-2>（The reason for exclusion: duplicate record）
237. Hu, M. M., Wang, S., Wu, C. Q., Li, K. P., Geng, Z. H., Xu, G. H., & Dong, L. (2024). Efficacy of robot-assisted gait training on lower extremity function in subacute stroke patients: a systematic review and meta-analysis. *Journal of neuroengineering and rehabilitation*, *21*(1), 165. <https://doi.org/10.1186/s12984-024-01463-1>（The reason for exclusion: review）
238. Hesse, S., Tomelleri, C., Bardeleben, A., Werner, C., & Waldner, A. (2012). Robot-assisted practice of gait and stair climbing in nonambulatory stroke patients. *Journal of rehabilitation research and development*, *49*(4), 613–622. <https://doi.org/10.1682/jrrd.2011.08.0142>（The reason for exclusion: duplicate record）
239. Mazzoleni, S., Focacci, A., Franceschini, M., Waldner, A., Spagnuolo, C., Battini, E., & Bonaiuti, D. (2017). Robot-assisted end-effector-based gait training in chronic stroke patients: A multicentric uncontrolled observational retrospective clinical study. *NeuroRehabilitation*, *40*(4), 483–492. <https://doi.org/10.3233/NRE-161435>（The reason for exclusion: duplicate record）
240. Klinkwan, P., Kongmaroeng, C., Muengtaweepongsa, S., & Limtrakarn, W. (2023). Prototype development of bilateral arm mirror-like-robotic rehabilitation device for acute stroke patients. *Biomedical physics & engineering express*, *9*(4), 10.1088/2057-1976/acd11d. <https://doi.org/10.1088/2057-1976/acd11d>（The reason for exclusion: Non robot-assisted task-oriented training）
241. Li, N., Yang, T., Yu, P., Chang, J., Zhao, L., Zhao, X., Elhajj, I. H., Xi, N., & Liu, L. (2018). Bio-inspired upper limb soft exoskeleton to reduce stroke-induced complications. *Bioinspiration & biomimetics*, *13*(6), 066001. <https://doi.org/10.1088/1748-3190/aad8d4>（The reason for exclusion: Non robot-assisted task-oriented training）
242. Yakşi, E., Bahadır, E. S., Yaşar, M. F., Alışık, T., Kurul, R., & Demirel, A. (2023). The effect of robot-assisted gait training frequency on walking, functional recovery, and quality of life in patients with stroke. *Acta neurologica Belgica*, *123*(2), 583–590. <https://doi.org/10.1007/s13760-023-02194-1>（The reason for exclusion: duplicate record）
243. Lim, D. Y., Lai, H. S., & Yeow, R. C. (2023). A bidirectional fabric-based soft robotic glove for hand function assistance in patients with chronic stroke. *Journal of neuroengineering and rehabilitation*, *20*(1), 120. https://doi.org/10.1186/s12984-023-01250-4（The reason for exclusion: duplicate record）
244. Neo, J. R. E., Visperas, C. A., Tan, M. P. H., & Tay, S. S. (2023). Novel use of robot-assisted gait rehabilitation in a patient with stroke and blindness. *BMJ case reports*, *16*(7), e255457. <https://doi.org/10.1136/bcr-2023-255457>（The reason for exclusion: Non-RCTs）
245. Sayın, A. M., Duruturk, N., Balaban, B., & Korkusuz, S. (2023). The effect of robot-assisted walking in different modalities on cardiorespiratory responses and energy consumption in patients with subacute stroke. *Neurological research*, *45*(7), 688–694. <https://doi.org/10.1080/01616412.2023.2188520>（The reason for exclusion: Non-RCTs）
246. Uehara, S., Yuasa, A., Ushizawa, K., Kitamura, S., Yamazaki, K., Otaka, E., & Otaka, Y. (2023). Direction-dependent differences in the quality and quantity of horizontal reaching in people after stroke. *Journal of neurophysiology*, *130*(4), 861–870. <https://doi.org/10.1152/jn.00455.2022>（The reason for exclusion: duplicate record）
247. Tomida, K., Sonoda, S., Hirano, S., Suzuki, A., Tanino, G., Kawakami, K., Saitoh, E., & Kagaya, H. (2019). Randomized Controlled Trial of Gait Training Using Gait Exercise Assist Robot (GEAR) in Stroke Patients with Hemiplegia. *Journal of stroke and cerebrovascular diseases : the official journal of National Stroke Association*, *28*(9), 2421–2428. <https://doi.org/10.1016/j.jstrokecerebrovasdis.2019.06.030>（The reason for exclusion: duplicate record）
248. Volpe, B. T., Ferraro, M., Lynch, D., Christos, P., Krol, J., Trudell, C., Krebs, H. I., & Hogan, N. (2005). Robotics and other devices in the treatment of patients recovering from stroke. *Current neurology and neuroscience reports*, *5*(6), 465–470. <https://doi.org/10.1007/s11910-005-0035-y>（The reason for exclusion: duplicate record）
249. Shin, J., An, H., Yang, S., Park, C., Lee, Y., & You, S. J. H. (2022). Comparative effects of passive and active mode robot-assisted gait training on brain and muscular activities in sub-acute and chronic stroke. *NeuroRehabilitation*, *51*(1), 51–63. <https://doi.org/10.3233/NRE-210304>（The reason for exclusion: duplicate record）
250. Rikhof, C. J. H., Feenstra, Y., Fleuren, J. F. M., Buurke, J. H., Prinsen, E. C., Rietman, J. S., & Prange-Lasonder, G. B. (2024). Robot-assisted support combined with electrical stimulation for the lower extremity in stroke patients: a systematic review. *Journal of neural engineering*, *21*(2), 10.1088/1741-2552/ad377c. <https://doi.org/10.1088/1741-2552/ad377c>（The reason for exclusion: review）
251. Mehrholz J. (2019). Is Electromechanical and Robot-Assisted Arm Training Effective for Improving Arm Function in People Who Have Had a Stroke?: A Cochrane Review Summary With Commentary. *American journal of physical medicine & rehabilitation*, *98*(4), 339–340. <https://doi.org/10.1097/PHM.0000000000001133>（The reason for exclusion: review）
252. Semrau, J. A., Herter, T. M., Scott, S. H., & Dukelow, S. P. (2015). Examining Differences in Patterns of Sensory and Motor Recovery After Stroke With Robotics. *Stroke*, *46*(12), 3459–3469. <https://doi.org/10.1161/STROKEAHA.115.010750>（The reason for exclusion: duplicate record）
253. Chen, Y. W., Chiang, W. C., Chang, C. L., Lo, S. M., & Wu, C. Y. (2022). Comparative effects of EMG-driven robot-assisted therapy versus task-oriented training on motor and daily function in patients with stroke: a randomized cross-over trial. *Journal of neuroengineering and rehabilitation*, *19*(1), 6. <https://doi.org/10.1186/s12984-021-00961-w>（The reason for exclusion: duplicate record）
254. Yurkewich, A., Hebert, D., Wang, R. H., & Mihailidis, A. (2019). Hand Extension Robot Orthosis (HERO) Glove: Development and Testing With Stroke Survivors With Severe Hand Impairment. *IEEE transactions on neural systems and rehabilitation engineering : a publication of the IEEE Engineering in Medicine and Biology Society*, *27*(5), 916–926. <https://doi.org/10.1109/TNSRE.2019.2910011>（The reason for exclusion: duplicate record）
255. Joo, M. C., Jung, K. M., Kim, J. H., Jung, Y. J., Chang, W. N., & Shin, H. J. (2022). Robot-Assisted Therapy Combined with Trunk Restraint in Acute Stroke Patients: A Randomized Controlled Study. *Journal of stroke and cerebrovascular diseases : the official journal of National Stroke Association*, *31*(5), 106330. <https://doi.org/10.1016/j.jstrokecerebrovasdis.2022.106330>（The reason for exclusion: duplicate record）
256. Yeung, L. F., Lau, C. C. Y., Lai, C. W. K., Soo, Y. O. Y., Chan, M. L., & Tong, R. K. Y. (2021). Effects of wearable ankle robotics for stair and over-ground training on sub-acute stroke: a randomized controlled trial. *Journal of neuroengineering and rehabilitation*, *18*(1), 19. <https://doi.org/10.1186/s12984-021-00814-6>（The reason for exclusion: duplicate record）
257. Sloot, L. H., Baker, L. M., Bae, J., Porciuncula, F., Clément, B. F., Siviy, C., Nuckols, R. W., Baker, T., Sloutsky, R., Choe, D. K., O'Donnell, K., Ellis, T. D., Awad, L. N., & Walsh, C. J. (2023). Effects of a soft robotic exosuit on the quality and speed of overground walking depends on walking ability after stroke. *Journal of neuroengineering and rehabilitation*, *20*(1), 113. <https://doi.org/10.1186/s12984-023-01231-7>（The reason for exclusion: duplicate record）
258. Cho, K. H., & Song, W. K. (2019). Robot-Assisted Reach Training With an Active Assistant Protocol for Long-Term Upper Extremity Impairment Poststroke: A Randomized Controlled Trial. *Archives of physical medicine and rehabilitation*, *100*(2), 213–219. <https://doi.org/10.1016/j.apmr.2018.10.002>（The reason for exclusion: duplicate record）
259. Sloot, L., Bae, J., Baker, L., O'Donnell, K., Menard, N., Porciuncula, F., Choe, D., Ellis, T., Awad, L., & Walsh, C. (2022). O 089 - A soft robotic exosuit assisting the paretic ankle in patients post-stroke: Effect on muscle activation during overground walking. *Gait & posture*, *95*, 217–218. <https://doi.org/10.1016/j.gaitpost.2018.06.124>（The reason for exclusion: duplicate record）
260. Hyakutake, K., Morishita, T., Saita, K., Fukuda, H., Abe, H., Ogata, T., Kamada, S., & Inoue, T. (2022). Effect of Robot-assisted Rehabilitation to Botulinum Toxin A Injection for Upper Limb Disability in Patients with Chronic Stroke: A Case Series and Systematic Review. *Neurologia medico-chirurgica*, *62*(1), 35–44. <https://doi.org/10.2176/nmc.oa.2020-0408>（The reason for exclusion: duplicate record）
261. McCabe, J., Monkiewicz, M., Holcomb, J., Pundik, S., & Daly, J. J. (2015). Comparison of robotics, functional electrical stimulation, and motor learning methods for treatment of persistent upper extremity dysfunction after stroke: a randomized controlled trial. *Archives of physical medicine and rehabilitation*, *96*(6), 981–990. <https://doi.org/10.1016/j.apmr.2014.10.022>（The reason for exclusion: duplicate record）
262. Rodgers, H., Shaw, L., Bosomworth, H., Aird, L., Alvarado, N., Andole, S., Cohen, D. L., Dawson, J., Eyre, J., Finch, T., Ford, G. A., Hislop, J., Hogg, S., Howel, D., Hughes, N., Krebs, H. I., Price, C., Rochester, L., Stamp, E., Ternent, L., … Wilkes, S. (2017). Robot Assisted Training for the Upper Limb after Stroke (RATULS): study protocol for a randomised controlled trial. *Trials*, *18*(1), 340. <https://doi.org/10.1186/s13063-017-2083-4>（The reason for exclusion: duplicate record）
263. Hogan, N., Krebs, H. I., Rohrer, B., Palazzolo, J. J., Dipietro, L., Fasoli, S. E., Stein, J., Hughes, R., Frontera, W. R., Lynch, D., & Volpe, B. T. (2006). Motions or muscles? Some behavioral factors underlying robotic assistance of motor recovery. *Journal of rehabilitation research and development*, *43*(5), 605–618. <https://doi.org/10.1682/jrrd.2005.06.0103>（The reason for exclusion: duplicate record）
264. Fischer, H. C., Stubblefield, K., Kline, T., Luo, X., Kenyon, R. V., & Kamper, D. G. (2007). Hand rehabilitation following stroke: a pilot study of assisted finger extension training in a virtual environment. *Topics in stroke rehabilitation*, *14*(1), 1–12. https://doi.org/10.1310/tsr1401-1（The reason for exclusion: duplicate record）
265. Jahangir, A. W., Tan, H. J., Norlinah, M. I., Nafisah, W. Y., Ramesh, S., Hamidon, B. B., & Raymond, A. A. (2007). Intramuscular injection of botulinum toxin for the treatment of wrist and finger spasticity after stroke. *The Medical journal of Malaysia*, *62*(4), 319–322.（The reason for exclusion: Non robot-assisted task-oriented training）
266. Cauraugh, J., Light, K., Kim, S., Thigpen, M., & Behrman, A. (2000). Chronic motor dysfunction after stroke: recovering wrist and finger extension by electromyography-triggered neuromuscular stimulation. *Stroke*, *31*(6), 1360–1364. <https://doi.org/10.1161/01.str.31.6.1360>（The reason for exclusion: Non robot-assisted task-oriented training）
267. Kim, D. G., Cho, Y. W., Hong, J. H., Song, J. C., Chung, H. A., Bai, D. S., Lee, C. H., & Jang, S. H. (2008). Effect of constraint-induced movement therapy with modified opposition restriction orthosis in chronic hemiparetic patients with stroke. *NeuroRehabilitation*, *23*(3), 239–244.（The reason for exclusion: duplicate record）
268. Seniów, J., Bilik, M., Leśniak, M., Waldowski, K., Iwański, S., & Członkowska, A. (2012). Transcranial magnetic stimulation combined with physiotherapy in rehabilitation of poststroke hemiparesis: a randomized, double-blind, placebo-controlled study. *Neurorehabilitation and neural repair*, *26*(9), 1072–1079. <https://doi.org/10.1177/1545968312445635>（The reason for exclusion: duplicate record）
269. Thielbar, K. O., Lord, T. J., Fischer, H. C., Lazzaro, E. C., Barth, K. C., Stoykov, M. E., Triandafilou, K. M., & Kamper, D. G. (2014). Training finger individuation with a mechatronic-virtual reality system leads to improved fine motor control post-stroke. *Journal of neuroengineering and rehabilitation*, *11*, 171. <https://doi.org/10.1186/1743-0003-11-171>（The reason for exclusion: duplicate record）
270. Singer, B. J., Vallence, A. M., Cleary, S., Cooper, I., & Loftus, A. M. (2013). The effect of EMG triggered electrical stimulation plus task practice on arm function in chronic stroke patients with moderate-severe arm deficits. *Restorative neurology and neuroscience*, *31*(6), 681–691. <https://doi.org/10.3233/RNN-130319>（The reason for exclusion: duplicate record）
271. Dolganov, M. V., & Karpova, M. I. (2019). Virtual'naia real'nost' pri narushenii funktsii ruki: osobennosti primeneniia v ostrom periode insul'ta [Virtual reality in upper extremity dysfunction: specific features of usage in acute stroke]. *Voprosy kurortologii, fizioterapii, i lechebnoi fizicheskoi kultury*, *96*(5), 19–28. <https://doi.org/10.17116/kurort20199605119>（The reason for exclusion: duplicate record）
272. Trombly, C. A., Thayer-Nason, L., Bliss, G., Girard, C. A., Lyrist, L. A., & Brexa-Hooson, A. (1986). The effectiveness of therapy in improving finger extension in stroke patients. *The American journal of occupational therapy : official publication of the American Occupational Therapy Association*, *40*(9), 612–617. <https://doi.org/10.5014/ajot.40.9.612>（The reason for exclusion: duplicate record）

Klamroth-Marganska V. (2018). Stroke Rehabilitation: Therapy Robots and Assistive Devices. *Advances in experimental medicine and biology*, *1065*, 579–587. https://doi.org/10.1007/978-3-319-77932-4_35（The reason for exclusion: review）

1. Muller, C. O., Metais, A., Boublay, N., Breuil, C., Daligault, S., Di Rienzo, F., Guillot, A., Collet, C., Krolak-Salmon, P., & Saimpont, A. (2024). Anodal transcranial direct current stimulation does not enhance the effects of motor imagery training of a sequential finger-tapping task in young adults. *Journal of sports sciences*, *42*(5), 392–403. <https://doi.org/10.1080/02640414.2024.2328418>（The reason for exclusion: Non robot-assisted task-oriented training）
2. Bhatt, E., Nagpal, A., Greer, K. H., Grunewald, T. K., Steele, J. L., Wiemiller, J. W., Lewis, S. M., & Carey, J. R. (2007). Effect of finger tracking combined with electrical stimulation on brain reorganization and hand function in subjects with stroke. *Experimental brain research*, *182*(4), 435–447. <https://doi.org/10.1007/s00221-007-1001-5>（The reason for exclusion: Non robot-assisted task-oriented training）
3. Fischer, H. C., Stubblefield, K., Kline, T., Luo, X., Kenyon, R. V., & Kamper, D. G. (2007). Hand rehabilitation following stroke: a pilot study of assisted finger extension training in a virtual environment. *Topics in stroke rehabilitation*, *14*(1), 1–12. https://doi.org/10.1310/tsr1401-1（The reason for exclusion: duplicate record）
4. Jahangir, A. W., Tan, H. J., Norlinah, M. I., Nafisah, W. Y., Ramesh, S., Hamidon, B. B., & Raymond, A. A. (2007). Intramuscular injection of botulinum toxin for the treatment of wrist and finger spasticity after stroke. *The Medical journal of Malaysia*, *62*(4), 319–322.（The reason for exclusion: Non robot-assisted task-oriented training）
5. Cauraugh, J., Light, K., Kim, S., Thigpen, M., & Behrman, A. (2000). Chronic motor dysfunction after stroke: recovering wrist and finger extension by electromyography-triggered neuromuscular stimulation. *Stroke*, *31*(6), 1360–1364. <https://doi.org/10.1161/01.str.31.6.1360>（The reason for exclusion: Non robot-assisted task-oriented training）
6. Kim, D. G., Cho, Y. W., Hong, J. H., Song, J. C., Chung, H. A., Bai, D. S., Lee, C. H., & Jang, S. H. (2008). Effect of constraint-induced movement therapy with modified opposition restriction orthosis in chronic hemiparetic patients with stroke. *NeuroRehabilitation*, *23*(3), 239–244.（The reason for exclusion: duplicate record）
7. Seniów, J., Bilik, M., Leśniak, M., Waldowski, K., Iwański, S., & Członkowska, A. (2012). Transcranial magnetic stimulation combined with physiotherapy in rehabilitation of poststroke hemiparesis: a randomized, double-blind, placebo-controlled study. *Neurorehabilitation and neural repair*, *26*(9), 1072–1079. <https://doi.org/10.1177/1545968312445635>（The reason for exclusion: duplicate record）
8. Thielbar, K. O., Lord, T. J., Fischer, H. C., Lazzaro, E. C., Barth, K. C., Stoykov, M. E., Triandafilou, K. M., & Kamper, D. G. (2014). Training finger individuation with a mechatronic-virtual reality system leads to improved fine motor control post-stroke. *Journal of neuroengineering and rehabilitation*, *11*, 171. <https://doi.org/10.1186/1743-0003-11-171>（The reason for exclusion: duplicate record）
9. Singer, B. J., Vallence, A. M., Cleary, S., Cooper, I., & Loftus, A. M. (2013). The effect of EMG triggered electrical stimulation plus task practice on arm function in chronic stroke patients with moderate-severe arm deficits. *Restorative neurology and neuroscience*, *31*(6), 681–691. <https://doi.org/10.3233/RNN-130319>（The reason for exclusion: duplicate record）
10. Bakker, C. D., Massa, M., Daffertshofer, A., Pasman, J. W., van Kuijk, A. A., Kwakkel, G., & Stegeman, D. F. (2019). The addition of the MEP amplitude of finger extension muscles to clinical predictors of hand function after stroke: A prospective cohort study. *Restorative neurology and neuroscience*, *37*(5), 445–456. https://doi.org/10.3233/RNN-180890（The reason for exclusion: Non robot-assisted task-oriented training）
11. Yıldızgören, M. T., Nakipoğlu Yüzer, G. F., Ekiz, T., & Özgirgin, N. (2014). Effects of neuromuscular electrical stimulation on the wrist and finger flexor spasticity and hand functions in cerebral palsy. *Pediatric neurology*, *51*(3), 360–364. https://doi.org/10.1016/j.pediatrneurol.2014.05.009（The reason for exclusion: Non robot-assisted task-oriented training）
12. Lin, C. H., Chou, L. W., Luo, H. J., Tsai, P. Y., Lieu, F. K., Chiang, S. L., & Sung, W. H. (2015). Effects of Computer-Aided Interlimb Force Coupling Training on Paretic Hand and Arm Motor Control following Chronic Stroke: A Randomized Controlled Trial. *PloS one*, *10*(7), e0131048. https://doi.org/10.1371/journal.pone.0131048（The reason for exclusion: duplicate record）
13. Kwon, T. G., Park, E., Kang, C., Chang, W. H., & Kim, Y. H. (2016). The effects of combined repetitive transcranial magnetic stimulation and transcranial direct current stimulation on motor function in patients with stroke. *Restorative neurology and neuroscience*, *34*(6), 915–923. https://doi.org/10.3233/RNN-160654（The reason for exclusion: duplicate record）
14. Fluet, G. G., Merians, A. S., Qiu, Q., Davidow, A., & Adamovich, S. V. (2014). Comparing integrated training of the hand and arm with isolated training of the same effectors in persons with stroke using haptically rendered virtual environments, a randomized clinical trial. *Journal of neuroengineering and rehabilitation*, *11*, 126. <https://doi.org/10.1186/1743-0003-11-126> （The reason for exclusion: duplicate record）
15. Hwang, C. H., Seong, J. W., & Son, D. S. (2012). Individual finger synchronized robot-assisted hand rehabilitation in subacute to chronic stroke: a prospective randomized clinical trial of efficacy. *Clinical rehabilitation*, *26*(8), 696–704. <https://doi.org/10.1177/0269215511431473> （The reason for exclusion: duplicate record）
16. Duret, C., Courtial, O., Grosmaire, A. G., & Hutin, E. (2015). Use of a robotic device for the rehabilitation of severe upper limb paresis in subacute stroke: exploration of patient/robot interactions and the motor recovery process. *BioMed research international*, *2015*, 482389. <https://doi.org/10.1155/2015/482389>（The reason for exclusion: Non-RCTs）
17. Colombo, R., Pisano, F., Delconte, C., Mazzone, A., Grioni, G., Castagna, M., Bazzini, G., Imarisio, C., Maggioni, G., & Pistarini, C. (2017). Comparison of exercise training effect with different robotic devices for upper limb rehabilitation: a retrospective study. *European journal of physical and rehabilitation medicine*, *53*(2), 240–248. https://doi.org/10.23736/S1973-9087.16.04297-0（The reason for exclusion: Non-RCTs）
18. Rowe, J. B., Chan, V., Ingemanson, M. L., Cramer, S. C., Wolbrecht, E. T., & Reinkensmeyer, D. J. (2017). Robotic Assistance for Training Finger Movement Using a Hebbian Model: A Randomized Controlled Trial. *Neurorehabilitation and neural repair*, *31*(8), 769–780. <https://doi.org/10.1177/1545968317721975>（The reason for exclusion: duplicate record）
19. Lo, A. C., Guarino, P. D., Richards, L. G., Haselkorn, J. K., Wittenberg, G. F., Federman, D. G., Ringer, R. J., Wagner, T. H., Krebs, H. I., Volpe, B. T., Bever, C. T., Jr, Bravata, D. M., Duncan, P. W., Corn, B. H., Maffucci, A. D., Nadeau, S. E., Conroy, S. S., Powell, J. M., Huang, G. D., & Peduzzi, P. (2010). Robot-assisted therapy for long-term upper-limb impairment after stroke. *The New England journal of medicine*, *362*(19), 1772–1783. <https://doi.org/10.1056/NEJMoa0911341>（The reason for exclusion: duplicate record）
20. Taheri, H., Rowe, J. B., Gardner, D., Chan, V., Reinkensmeyer, D. J., & Wolbrecht, E. T. (2012). Robot-assisted Guitar Hero for finger rehabilitation after stroke. *Annual International Conference of the IEEE Engineering in Medicine and Biology Society. IEEE Engineering in Medicine and Biology Society. Annual International Conference*, *2012*, 3911–3917. <https://doi.org/10.1109/EMBC.2012.6346822>（The reason for exclusion: meeting abstract）
21. Alingh, J. F., Fleerkotte, B. M., Groen, B. E., Rietman, J. S., Weerdesteyn, V., van Asseldonk, E. H. F., Geurts, A. C. H., & Buurke, J. H. (2021). Effect of assist-as-needed robotic gait training on the gait pattern post stroke: a randomized controlled trial. *Journal of neuroengineering and rehabilitation*, *18*(1), 26. <https://doi.org/10.1186/s12984-020-00800-4>（The reason for exclusion: duplicate record）
22. Zhou, H. X., Hu, J., Yun, R. S., Zhao, Z. Z., Lai, M. H., Sun, L. H., & Luo, K. L. (2023). Synergy-based functional electrical stimulation and robotic-assisted for retraining reach-to-grasp in stroke: a study protocol for a randomized controlled trial. *BMC neurology*, *23*(1), 324. <https://doi.org/10.1186/s12883-023-03369-2>（The reason for exclusion: duplicate record）
23. Yun, N., Joo, M. C., Kim, S. C., & Kim, M. S. (2018). Robot-assisted gait training effectively improved lateropulsion in subacute stroke patients: a single-blinded randomized controlled trial. *European journal of physical and rehabilitation medicine*, *54*(6), 827–836. <https://doi.org/10.23736/S1973-9087.18.05077-3>（The reason for exclusion: duplicate record）
24. Ii, T., Hirano, S., Tanabe, S., Saitoh, E., Yamada, J., Mukaino, M., Watanabe, M., Sonoda, S., & Otaka, Y. (2020). Robot-assisted Gait Training Using Welwalk in Hemiparetic Stroke Patients: An Effectiveness Study with Matched Control. *Journal of stroke and cerebrovascular diseases : the official journal of National Stroke Association*, *29*(12), 105377. <https://doi.org/10.1016/j.jstrokecerebrovasdis.2020.105377>（The reason for exclusion: duplicate record）
25. Leerskov, K. S., Rikhof, C. J. H., Spaich, E. G., Dosen, S., Prange-Lasonder, G. B., Prinsen, E. C., Rietman, J. S., & Struijk, L. N. S. A. (2024). A robot-based hybrid lower limb system for Assist-As-Needed rehabilitation of stroke patients: Technical evaluation and clinical feasibility. *Computers in biology and medicine*, *179*, 108839. <https://doi.org/10.1016/j.compbiomed.2024.108839>（The reason for exclusion: duplicate record）
26. Hu, X. L., Tong, K. Y., Wei, X. J., Rong, W., Susanto, E. A., & Ho, S. K. (2013). The effects of post-stroke upper-limb training with an electromyography (EMG)-driven hand robot. *Journal of electromyography and kinesiology : official journal of the International Society of Electrophysiological Kinesiology*, *23*(5), 1065–1074. https://doi.org/10.1016/j.jelekin.2013.07.007（The reason for exclusion: Non-RCTs）
27. Duret, C., Hutin, E., Lehenaff, L., & Gracies, J. M. (2015). Do all sub acute stroke patients benefit from robot-assisted therapy? A retrospective study. *Restorative neurology and neuroscience*, *33*(1), 57–65. <https://doi.org/10.3233/RNN-140418>（The reason for exclusion: Non robot-assisted task-oriented training）
28. Sale, P., Mazzoleni, S., Lombardi, V., Galafate, D., Massimiani, M. P., Posteraro, F., Damiani, C., & Franceschini, M. (2014). Recovery of hand function with robot-assisted therapy in acute stroke patients: a randomized-controlled trial. *International journal of rehabilitation research. Internationale Zeitschrift fur Rehabilitationsforschung. Revue internationale de recherches de readaptation*, *37*(3), 236–242. <https://doi.org/10.1097/MRR.0000000000000059>（The reason for exclusion: Non robot-assisted task-oriented training）
29. Iwamoto, Y., Imura, T., Suzukawa, T., Fukuyama, H., Ishii, T., Taki, S., Imada, N., Shibukawa, M., Inagawa, T., Araki, H., & Araki, O. (2019). Combination of Exoskeletal Upper Limb Robot and Occupational Therapy Improve Activities of Daily Living Function in Acute Stroke Patients. *Journal of stroke and cerebrovascular diseases : the official journal of National Stroke Association*, *28*(7), 2018–2025. <https://doi.org/10.1016/j.jstrokecerebrovasdis.2019.03.006>（The reason for exclusion: duplicate record）
30. Akıncı, M., Burak, M., Yaşar, E., & Kılıç, R. T. (2023). The effects of Robot-assisted gait training and virtual reality on balance and gait in stroke survivors: A randomized controlled trial. *Gait & posture*, *103*, 215–222. <https://doi.org/10.1016/j.gaitpost.2023.05.013>（The reason for exclusion: duplicate record）
31. Sale, P., Franceschini, M., Mazzoleni, S., Palma, E., Agosti, M., & Posteraro, F. (2014). Effects of upper limb robot-assisted therapy on motor recovery in subacute stroke patients. *Journal of neuroengineering and rehabilitation*, *11*, 104. <https://doi.org/10.1186/1743-0003-11-104>（The reason for exclusion: duplicate record）
32. Schrader, M., Sterr, A., Kettlitz, R., Wohlmeiner, A., Buschfort, R., Dohle, C., & Bamborschke, S. (2022). The effect of mirror therapy can be improved by simultaneous robotic assistance. *Restorative neurology and neuroscience*, *40*(3), 185–194. <https://doi.org/10.3233/RNN-221263>（The reason for exclusion: duplicate record）
33. Inoue, S., Otaka, Y., Kumagai, M., Sugasawa, M., Mori, N., & Kondo, K. (2022). Effects of Balance Exercise Assist Robot training for patients with hemiparetic stroke: a randomized controlled trial. *Journal of neuroengineering and rehabilitation*, *19*(1), 12. <https://doi.org/10.1186/s12984-022-00989-6>（The reason for exclusion: duplicate record）
34. Ranzani, R., Lambercy, O., Metzger, J. C., Califfi, A., Regazzi, S., Dinacci, D., Petrillo, C., Rossi, P., Conti, F. M., & Gassert, R. (2020). Neurocognitive robot-assisted rehabilitation of hand function: a randomized control trial on motor recovery in subacute stroke. *Journal of neuroengineering and rehabilitation*, *17*(1), 115. <https://doi.org/10.1186/s12984-020-00746-7>（The reason for exclusion: duplicate record）
35. Lee, H. C., Kuo, F. L., Lin, Y. N., Liou, T. H., Lin, J. C., & Huang, S. W. (2021). Effects of Robot-Assisted Rehabilitation on Hand Function of People With Stroke: A Randomized, Crossover-Controlled, Assessor-Blinded Study. *The American journal of occupational therapy : official publication of the American Occupational Therapy Association*, *75*(1), 7501205020p1–7501205020p11. <https://doi.org/10.5014/ajot.2021.038232>（The reason for exclusion: duplicate record）
36. Novak, D., & Riener, R. (2020). Sensor Fusion in Assistive and Rehabilitation Robotics. *Sensors (Basel, Switzerland)*, *20*(18), 5235. <https://doi.org/10.3390/s20185235>（The reason for exclusion: Non robot-assisted task-oriented training）
37. Dehem, S., Gilliaux, M., Stoquart, G., Detrembleur, C., Jacquemin, G., Palumbo, S., Frederick, A., & Lejeune, T. (2019). Effectiveness of upper-limb robotic-assisted therapy in the early rehabilitation phase after stroke: A single-blind, randomised, controlled trial. *Annals of physical and rehabilitation medicine*, *62*(5), 313–320. <https://doi.org/10.1016/j.rehab.2019.04.002>（The reason for exclusion: duplicate record）
38. Leconte, P., & Ronsse, R. (2016). Performance-based robotic assistance during rhythmic arm exercises. *Journal of neuroengineering and rehabilitation*, *13*(1), 82. <https://doi.org/10.1186/s12984-016-0189-7>（The reason for exclusion: duplicate record）
39. Lo, A. C., Guarino, P., Krebs, H. I., Volpe, B. T., Bever, C. T., Duncan, P. W., Ringer, R. J., Wagner, T. H., Richards, L. G., Bravata, D. M., Haselkorn, J. K., Wittenberg, G. F., Federman, D. G., Corn, B. H., Maffucci, A. D., & Peduzzi, P. (2009). Multicenter randomized trial of robot-assisted rehabilitation for chronic stroke: methods and entry characteristics for VA ROBOTICS. *Neurorehabilitation and neural repair*, *23*(8), 775–783. <https://doi.org/10.1177/1545968309338195>（The reason for exclusion: duplicate record）
40. Hung, C. S., Hsieh, Y. W., Wu, C. Y., Lin, Y. T., Lin, K. C., & Chen, C. L. (2016). The Effects of Combination of Robot-Assisted Therapy With Task-Specific or Impairment-Oriented Training on Motor Function and Quality of Life in Chronic Stroke. *PM & R : the journal of injury, function, and rehabilitation*, *8*(8), 721–729. <https://doi.org/10.1016/j.pmrj.2016.01.008>（The reason for exclusion: duplicate record）
41. Lee, S. Y., Choi, Y. S., Kim, M. H., & Chang, W. N. (2024). Effects of robot-assisted walking training on balance, motor function, and ADL depending on severity levels in stroke patients. *Technology and health care : official journal of the European Society for Engineering and Medicine*, *32*(5), 3293–3307. <https://doi.org/10.3233/THC-232015>（The reason for exclusion: duplicate record）
42. Bay, B., Kiwus, L. M., Goßling, A., Koester, L., Blaum, C., Schrage, B., Clemmensen, P., Blankenberg, S., Waldeyer, C., Seiffert, M., & Brunner, F. J. (2024). Procedural and one-year outcomes of robotic-assisted versus manual percutaneous coronary intervention. *EuroIntervention : journal of EuroPCR in collaboration with the Working Group on Interventional Cardiology of the European Society of Cardiology*, *20*(1), 56–65. <https://doi.org/10.4244/EIJ-D-23-00375>（The reason for exclusion: duplicate record）
43. Devittori, G., Ranzani, R., Dinacci, D., Romiti, D., Califfi, A., Petrillo, C., Rossi, P., Gassert, R., & Lambercy, O. (2022). Automatic and Personalized Adaptation of Therapy Parameters for Unsupervised Robot-Assisted Rehabilitation: a Pilot Evaluation. *IEEE ... International Conference on Rehabilitation Robotics : [proceedings]*, *2022*, 1–6. <https://doi.org/10.1109/ICORR55369.2022.9896527>（The reason for exclusion: duplicate record）
44. Leonardis, D., Barsotti, M., Loconsole, C., Solazzi, M., Troncossi, M., Mazzotti, C., Castelli, V. P., Procopio, C., Lamola, G., Chisari, C., Bergamasco, M., & Frisoli, A. (2015). An EMG-Controlled Robotic Hand Exoskeleton for Bilateral Rehabilitation. *IEEE transactions on haptics*, *8*(2), 140–151. <https://doi.org/10.1109/TOH.2015.2417570>（The reason for exclusion: duplicate record）
45. Vahdat, S., Darainy, M., Thiel, A., & Ostry, D. J. (2019). A Single Session of Robot-Controlled Proprioceptive Training Modulates Functional Connectivity of Sensory Motor Networks and Improves Reaching Accuracy in Chronic Stroke. *Neurorehabilitation and neural repair*, *33*(1), 70–81. <https://doi.org/10.1177/1545968318818902>（The reason for exclusion: duplicate record）
46. Lin, Y., Qu, Q., Lin, Y., He, J., Zhang, Q., Wang, C., Jiang, Z., Guo, F., & Jia, J. (2021). Customizing Robot-Assisted Passive Neurorehabilitation Exercise Based on Teaching Training Mechanism. *BioMed research international*, *2021*, 9972560. <https://doi.org/10.1155/2021/9972560>（The reason for exclusion: duplicate record）
47. Chan, H. L., Hung, J. W., Chang, K. C., & Wu, C. Y. (2021). Myoelectric analysis of upper-extremity muscles during robot-assisted bilateral wrist flexion-extension in subjects with poststroke hemiplegia. *Clinical biomechanics (Bristol, Avon)*, *87*, 105412. <https://doi.org/10.1016/j.clinbiomech.2021.105412>（The reason for exclusion: duplicate record）
48. Ru, H., Gao, W., Ou, W., Yang, X., Li, A., Fu, Z., Huo, J., Yang, B., Zhang, Y., Xiao, X., Yang, Z., & Huang, J. (2023). A Flexible Wearable Supernumerary Robotic Limb for Chronic Stroke Patients. *Journal of visualized experiments : JoVE*, (200), 10.3791/65917. <https://doi.org/10.3791/65917>（The reason for exclusion: duplicate record）
49. Hirano, S., Saitoh, E., Imoto, D., Ii, T., Tsunoda, T., & Otaka, Y. (2024). Effects of robot-assisted gait training using the Welwalk on gait independence for individuals with hemiparetic stroke: an assessor-blinded, multicenter randomized controlled trial. *Journal of neuroengineering and rehabilitation*, *21*(1), 76. <https://doi.org/10.1186/s12984-024-01370-5>（The reason for exclusion: duplicate record）
50. Wang, L., Zheng, Y., Dang, Y., Teng, M., Zhang, X., Cheng, Y., Zhang, X., Yu, Q., Yin, A., & Lu, X. (2021). Effects of robot-assisted training on balance function in patients with stroke: A systematic review and meta-analysis. *Journal of rehabilitation medicine*, *53*(4), jrm00174. <https://doi.org/10.2340/16501977-2815>（The reason for exclusion: review）
51. Amano, Y., Noma, T., Etoh, S., Miyata, R., Kawamura, K., & Shimodozono, M. (2020). Reaching exercise for chronic paretic upper extremity after stroke using a novel rehabilitation robot with arm-weight support and concomitant electrical stimulation and vibration: before-and-after feasibility trial. *Biomedical engineering online*, *19*(1), 28. <https://doi.org/10.1186/s12938-020-00774-3>（The reason for exclusion: Non robot-assisted task-oriented training）
52. Chen, Y. W., Li, K. Y., Lin, C. H., Hung, P. H., Lai, H. T., & Wu, C. Y. (2023). The effect of sequential combination of mirror therapy and robot-assisted therapy on motor function, daily function, and self-efficacy after stroke. *Scientific reports*, *13*(1), 16841. https://doi.org/10.1038/s41598-023-43981-3（The reason for exclusion: duplicate record）
53. Zhang, B., Wong, K. P., Kang, R., Fu, S., Qin, J., & Xiao, Q. (2023). Efficacy of Robot-Assisted and Virtual Reality Interventions on Balance, Gait, and Daily Function in Patients With Stroke: A Systematic Review and Network Meta-analysis. *Archives of physical medicine and rehabilitation*, *104*(10), 1711–1719. <https://doi.org/10.1016/j.apmr.2023.04.005>（The reason for exclusion: review）
54. Yang, X., Shi, X., Xue, X., & Deng, Z. (2023). Efficacy of Robot-Assisted Training on Rehabilitation of Upper Limb Function in Patients With Stroke: A Systematic Review and Meta-analysis. *Archives of physical medicine and rehabilitation*, *104*(9), 1498–1513. <https://doi.org/10.1016/j.apmr.2023.02.004>（The reason for exclusion: review）
55. Iwamoto, Y., Imura, T., Suzukawa, T., Fukuyama, H., Ishii, T., Taki, S., Imada, N., Shibukawa, M., Inagawa, T., Araki, H., & Araki, O. (2019). Combination of Exoskeletal Upper Limb Robot and Occupational Therapy Improve Activities of Daily Living Function in Acute Stroke Patients. *Journal of stroke and cerebrovascular diseases : the official journal of National Stroke Association*, *28*(7), 2018–2025. <https://doi.org/10.1016/j.jstrokecerebrovasdis.2019.03.006>（The reason for exclusion: duplicate record）
56. Akıncı, M., Burak, M., Yaşar, E., & Kılıç, R. T. (2023). The effects of Robot-assisted gait training and virtual reality on balance and gait in stroke survivors: A randomized controlled trial. *Gait & posture*, *103*, 215–222. <https://doi.org/10.1016/j.gaitpost.2023.05.013>（The reason for exclusion: duplicate record）
57. Sale, P., Franceschini, M., Mazzoleni, S., Palma, E., Agosti, M., & Posteraro, F. (2014). Effects of upper limb robot-assisted therapy on motor recovery in subacute stroke patients. *Journal of neuroengineering and rehabilitation*, *11*, 104. <https://doi.org/10.1186/1743-0003-11-104>（The reason for exclusion: duplicate record）
58. Schrader, M., Sterr, A., Kettlitz, R., Wohlmeiner, A., Buschfort, R., Dohle, C., & Bamborschke, S. (2022). The effect of mirror therapy can be improved by simultaneous robotic assistance. *Restorative neurology and neuroscience*, *40*(3), 185–194. <https://doi.org/10.3233/RNN-221263>（The reason for exclusion: duplicate record）
59. Inoue, S., Otaka, Y., Kumagai, M., Sugasawa, M., Mori, N., & Kondo, K. (2022). Effects of Balance Exercise Assist Robot training for patients with hemiparetic stroke: a randomized controlled trial. *Journal of neuroengineering and rehabilitation*, *19*(1), 12. <https://doi.org/10.1186/s12984-022-00989-6>（The reason for exclusion: duplicate record）
60. Ranzani, R., Lambercy, O., Metzger, J. C., Califfi, A., Regazzi, S., Dinacci, D., Petrillo, C., Rossi, P., Conti, F. M., & Gassert, R. (2020). Neurocognitive robot-assisted rehabilitation of hand function: a randomized control trial on motor recovery in subacute stroke. *Journal of neuroengineering and rehabilitation*, *17*(1), 115. <https://doi.org/10.1186/s12984-020-00746-7>（The reason for exclusion: duplicate record）
61. Lee, H. C., Kuo, F. L., Lin, Y. N., Liou, T. H., Lin, J. C., & Huang, S. W. (2021). Effects of Robot-Assisted Rehabilitation on Hand Function of People With Stroke: A Randomized, Crossover-Controlled, Assessor-Blinded Study. *The American journal of occupational therapy : official publication of the American Occupational Therapy Association*, *75*(1), 7501205020p1–7501205020p11. <https://doi.org/10.5014/ajot.2021.038232>（The reason for exclusion: duplicate record）
62. Novak, D., & Riener, R. (2020). Sensor Fusion in Assistive and Rehabilitation Robotics. *Sensors (Basel, Switzerland)*, *20*(18), 5235. <https://doi.org/10.3390/s20185235>（The reason for exclusion: Non robot-assisted task-oriented training）
63. Dehem, S., Gilliaux, M., Stoquart, G., Detrembleur, C., Jacquemin, G., Palumbo, S., Frederick, A., & Lejeune, T. (2019). Effectiveness of upper-limb robotic-assisted therapy in the early rehabilitation phase after stroke: A single-blind, randomised, controlled trial. *Annals of physical and rehabilitation medicine*, *62*(5), 313–320. <https://doi.org/10.1016/j.rehab.2019.04.002>（The reason for exclusion: duplicate record）
64. Castelli E. (2023). Robotic Rehabilitation in Children. *Psychiatria Danubina*, *35*(Suppl 3), 93–94.（The reason for exclusion: Non robot-assisted task-oriented training）
65. Mehrholz J. (2019). Is Electromechanical and Robot-Assisted Arm Training Effective for Improving Arm Function in People Who Have Had a Stroke?: A Cochrane Review Summary With Commentary. *American journal of physical medicine & rehabilitation*, *98*(4), 339–340. <https://doi.org/10.1097/PHM.0000000000001133>（The reason for exclusion: review）
66. Semrau, J. A., Herter, T. M., Scott, S. H., & Dukelow, S. P. (2015). Examining Differences in Patterns of Sensory and Motor Recovery After Stroke With Robotics. *Stroke*, *46*(12), 3459–3469. <https://doi.org/10.1161/STROKEAHA.115.010750>（The reason for exclusion: duplicate record）
67. Chen, Y. W., Chiang, W. C., Chang, C. L., Lo, S. M., & Wu, C. Y. (2022). Comparative effects of EMG-driven robot-assisted therapy versus task-oriented training on motor and daily function in patients with stroke: a randomized cross-over trial. *Journal of neuroengineering and rehabilitation*, *19*(1), 6. <https://doi.org/10.1186/s12984-021-00961-w>（The reason for exclusion: duplicate record）
68. Yurkewich, A., Hebert, D., Wang, R. H., & Mihailidis, A. (2019). Hand Extension Robot Orthosis (HERO) Glove: Development and Testing With Stroke Survivors With Severe Hand Impairment. *IEEE transactions on neural systems and rehabilitation engineering : a publication of the IEEE Engineering in Medicine and Biology Society*, *27*(5), 916–926. <https://doi.org/10.1109/TNSRE.2019.2910011>（The reason for exclusion: duplicate record）
69. Joo, M. C., Jung, K. M., Kim, J. H., Jung, Y. J., Chang, W. N., & Shin, H. J. (2022). Robot-Assisted Therapy Combined with Trunk Restraint in Acute Stroke Patients: A Randomized Controlled Study. *Journal of stroke and cerebrovascular diseases : the official journal of National Stroke Association*, *31*(5), 106330. <https://doi.org/10.1016/j.jstrokecerebrovasdis.2022.106330>（The reason for exclusion: duplicate record）
70. Yeung, L. F., Lau, C. C. Y., Lai, C. W. K., Soo, Y. O. Y., Chan, M. L., & Tong, R. K. Y. (2021). Effects of wearable ankle robotics for stair and over-ground training on sub-acute stroke: a randomized controlled trial. *Journal of neuroengineering and rehabilitation*, *18*(1), 19. <https://doi.org/10.1186/s12984-021-00814-6>（The reason for exclusion: duplicate record）
71. Thimabut, N., Yotnuengnit, P., Charoenlimprasert, J., Sillapachai, T., Hirano, S., Saitoh, E., & Piravej, K. (2022). Effects of the Robot-Assisted Gait Training Device Plus Physiotherapy in Improving Ambulatory Functions in Patients With Subacute Stroke With Hemiplegia: An Assessor-Blinded, Randomized Controlled Trial. *Archives of physical medicine and rehabilitation*, *103*(5), 843–850. <https://doi.org/10.1016/j.apmr.2022.01.146>（The reason for exclusion: duplicate record）
72. Bernal-Jiménez, J. J., Dileone, M., Mordillo-Mateos, L., Martín-Conty, J. L., Durantez-Fernández, C., Viñuela, A., Martín-Rodríguez, F., Lerin-Calvo, A., Alcántara-Porcuna, V., & Polonio-López, B. (2024). Combining Transcranial Direct Current Stimulation With Hand Robotic Rehabilitation in Chronic Stroke Patients: A Double-Blind Randomized Clinical Trial. *American journal of physical medicine & rehabilitation*, *103*(10), 875–882. <https://doi.org/10.1097/PHM.0000000000002446>（The reason for exclusion: duplicate record）
73. Proulx, C. E., Higgins, J., & Gagnon, D. H. (2023). Occupational therapists' evaluation of the perceived usability and utility of wearable soft robotic exoskeleton gloves for hand function rehabilitation following a stroke. *Disability and rehabilitation. Assistive technology*, *18*(6), 953–962. <https://doi.org/10.1080/17483107.2021.1938710>（The reason for exclusion: duplicate record）
74. Rikhof, C. J. H., Leerskov, K. S., Prange-Lasonder, G. B., Prinsen, E. C., Spaich, E. G., Dosen, S., Struijk, L. N. S. A., Buurke, J. H., & Rietman, J. S. (2024). Combining robotics and functional electrical stimulation for assist-as-needed support of leg movements in stroke patients: A feasibility study. *Medical engineering & physics*, *130*, 104216. <https://doi.org/10.1016/j.medengphy.2024.104216>（The reason for exclusion: duplicate record）
75. Cauraugh, J., Light, K., Kim, S., Thigpen, M., & Behrman, A. (2000). Chronic motor dysfunction after stroke: recovering wrist and finger extension by electromyography-triggered neuromuscular stimulation. *Stroke*, *31*(6), 1360–1364. <https://doi.org/10.1161/01.str.31.6.1360>（The reason for exclusion: Non robot-assisted task-oriented training）
76. Kim, D. G., Cho, Y. W., Hong, J. H., Song, J. C., Chung, H. A., Bai, D. S., Lee, C. H., & Jang, S. H. (2008). Effect of constraint-induced movement therapy with modified opposition restriction orthosis in chronic hemiparetic patients with stroke. NeuroRehabilitation, 23(3), 239–244.（The reason for exclusion: Non robot-assisted task-oriented training）
77. Seniów, J., Bilik, M., Leśniak, M., Waldowski, K., Iwański, S., & Członkowska, A. (2012). Transcranial magnetic stimulation combined with physiotherapy in rehabilitation of poststroke hemiparesis: a randomized, double-blind, placebo-controlled study. *Neurorehabilitation and neural repair*, *26*(9), 1072–1079. <https://doi.org/10.1177/1545968312445635>（The reason for exclusion: duplicate record）
78. Thielbar, K. O., Lord, T. J., Fischer, H. C., Lazzaro, E. C., Barth, K. C., Stoykov, M. E., Triandafilou, K. M., & Kamper, D. G. (2014). Training finger individuation with a mechatronic-virtual reality system leads to improved fine motor control post-stroke. *Journal of neuroengineering and rehabilitation*, *11*, 171. <https://doi.org/10.1186/1743-0003-11-171>（The reason for exclusion: duplicate record）
79. Singer, B. J., Vallence, A. M., Cleary, S., Cooper, I., & Loftus, A. M. (2013). The effect of EMG triggered electrical stimulation plus task practice on arm function in chronic stroke patients with moderate-severe arm deficits. *Restorative neurology and neuroscience*, *31*(6), 681–691. <https://doi.org/10.3233/RNN-130319>（The reason for exclusion: Non robot-assisted task-oriented training）
80. Dolganov, M. V., & Karpova, M. I. (2019). Virtual'naia real'nost' pri narushenii funktsii ruki: osobennosti primeneniia v ostrom periode insul'ta [Virtual reality in upper extremity dysfunction: specific features of usage in acute stroke]. *Voprosy kurortologii, fizioterapii, i lechebnoi fizicheskoi kultury*, *96*(5), 19–28. <https://doi.org/10.17116/kurort20199605119>（The reason for exclusion: Non robot-assisted task-oriented training）
81. Trombly, C. A., Thayer-Nason, L., Bliss, G., Girard, C. A., Lyrist, L. A., & Brexa-Hooson, A. (1986). The effectiveness of therapy in improving finger extension in stroke patients. *The American journal of occupational therapy : official publication of the American Occupational Therapy Association*, *40*(9), 612–617. https://doi.org/10.5014/ajot.40.9.612（The reason for exclusion: Non-RCTs）
82. Celnik, P., Webster, B., Glasser, D. M., & Cohen, L. G. (2008). Effects of action observation on physical training after stroke. *Stroke*, *39*(6), 1814–1820. https://doi.org/10.1161/STROKEAHA.107.508184（The reason for exclusion: Non robot-assisted task-oriented training）
83. Ro, T., Noser, E., Boake, C., Johnson, R., Gaber, M., Speroni, A., Bernstein, M., De Joya, A., Scott Burgin, W., Zhang, L., Taub, E., Grotta, J. C., & Levin, H. S. (2006). Functional reorganization and recovery after constraint-induced movement therapy in subacute stroke: case reports. *Neurocase*, *12*(1), 50–60. https://doi.org/10.1080/13554790500493415（The reason for exclusion: Non robot-assisted task-oriented training）
84. Madhoun, H. Y., Tan, B., Feng, Y., Zhou, Y., Zhou, C., & Yu, L. (2020). Task-based mirror therapy enhances the upper limb motor function in subacute stroke patients: a randomized control trial. *European journal of physical and rehabilitation medicine*, *56*(3), 265–271. <https://doi.org/10.23736/S1973-9087.20.06070-0>（The reason for exclusion: duplicate record）
85. Ranzani, R., Lambercy, O., Metzger, J. C., Califfi, A., Regazzi, S., Dinacci, D., Petrillo, C., Rossi, P., Conti, F. M., & Gassert, R. (2020). Neurocognitive robot-assisted rehabilitation of hand function: a randomized control trial on motor recovery in subacute stroke. *Journal of neuroengineering and rehabilitation*, *17*(1), 115. <https://doi.org/10.1186/s12984-020-00746-7>（The reason for exclusion: duplicate record）
86. Ji, E. K., Wang, H. H., Jung, S. J., Lee, K. B., Kim, J. S., Jo, L., Hong, B. Y., & Lim, S. H. (2021). Graded motor imagery training as a home exercise program for upper limb motor function in patients with chronic stroke: A randomized controlled trial. *Medicine*, *100*(3), e24351. <https://doi.org/10.1097/MD.0000000000024351>（The reason for exclusion: duplicate record）
87. Meadmore, K. L., Hughes, A. M., Freeman, C. T., Cai, Z., Tong, D., Burridge, J. H., & Rogers, E. (2012). Functional electrical stimulation mediated by iterative learning control and 3D robotics reduces motor impairment in chronic stroke. *Journal of neuroengineering and rehabilitation*, *9*, 32. <https://doi.org/10.1186/1743-0003-9-32>（The reason for exclusion: without relevant date）
88. Masiero, S., Armani, M., Ferlini, G., Rosati, G., & Rossi, A. (2014). Randomized trial of a robotic assistive device for the upper extremity during early inpatient stroke rehabilitation. *Neurorehabilitation and neural repair*, *28*(4), 377–386. <https://doi.org/10.1177/1545968313513073>（The reason for exclusion: duplicate record）
89. Song, K. J., Chun, M. H., Lee, J., & Lee, C. (2021). The effect of robot-assisted gait training on cortical activation in stroke patients: A functional near-infrared spectroscopy study. *NeuroRehabilitation*, *49*(1), 65–73. <https://doi.org/10.3233/NRE-210034>（The reason for exclusion: without relevant date）
90. Stefano, M., Patrizia, P., Mario, A., Ferlini, G., Rizzello, R., & Rosati, G. (2014). Robotic upper limb rehabilitation after acute stroke by NeReBot: evaluation of treatment costs. *BioMed research international*, *2014*, 265634. <https://doi.org/10.1155/2014/265634>（The reason for exclusion: duplicate record）
91. Duret, C., & Gracies, J. M. (2014). La rééducation du membre supérieur assistée par robot contribue-t-elle à améliorer le pronostic de l'hémiparésie vasculaire ? [Does upper limb robot-assisted rehabilitation contribute to improve the prognosis of post-stroke hemiparesis?]. *Revue neurologique*, *170*(11), 671–679. https://doi.org/10.1016/j.neurol.2014.07.012（The reason for exclusion: review）
92. Yeh, T. N., & Chou, L. W. (2023). User Experience Evaluation of Upper Limb Rehabilitation Robots: Implications for Design Optimization: A Pilot Study. *Sensors (Basel, Switzerland)*, *23*(21), 9003. <https://doi.org/10.3390/s23219003>（The reason for exclusion: without relevant date）
93. Backus, D., Winchester, P., & Tefertiller, C. (2010). Translating research into clinical practice: integrating robotics into neurorehabilitation for stroke survivors. *Topics in stroke rehabilitation*, *17*(5), 362–370. <https://doi.org/10.1310/tsr1705-362>（The reason for exclusion: Non-RCTs）
94. Hesse, S., Werner, C., Schonhardt, E. M., Bardeleben, A., Jenrich, W., & Kirker, S. G. (2007). Combined transcranial direct current stimulation and robot-assisted arm training in subacute stroke patients: a pilot study. *Restorative neurology and neuroscience*, *25*(1), 9–15.（The reason for exclusion: Non-RCTs）
95. Mazzoleni, S., Turchetti, G., Palla, I., Posteraro, F., & Dario, P. (2014). Acceptability of robotic technology in neuro-rehabilitation: preliminary results on chronic stroke patients. *Computer methods and programs in biomedicine*, *116*(2), 116–122. <https://doi.org/10.1016/j.cmpb.2013.12.017>（The reason for exclusion: without relevant date）
96. Iwamoto, Y., Imura, T., Tanaka, R., Mitsutake, T., Jung, H., Suzukawa, T., Taki, S., Imada, N., Inagawa, T., Araki, H., & Araki, O. (2022). Clinical Prediction Rule for Identifying the Stroke Patients who will Obtain Clinically Important Improvement of Upper Limb Motor Function by Robot-Assisted Upper Limb. *Journal of stroke and cerebrovascular diseases : the official journal of National Stroke Association*, *31*(7), 106517. <https://doi.org/10.1016/j.jstrokecerebrovasdis.2022.106517>（The reason for exclusion: Irrelevant study outcome）
97. Wu, M., Landry, J. M., Yen, S. C., Schmit, B. D., Hornby, T. G., & Rafferty, M. (2011). A novel cable-driven robotic training improves locomotor function in individuals post-stroke. *Annual International Conference of the IEEE Engineering in Medicine and Biology Society. IEEE Engineering in Medicine and Biology Society. Annual International Conference*, *2011*, 8539–8542. <https://doi.org/10.1109/IEMBS.2011.6092107>（The reason for exclusion: duplicate record）
98. Allington, J., Spencer, S. J., Klein, J., Buell, M., Reinkensmeyer, D. J., & Bobrow, J. (2011). Supinator Extender (SUE): a pneumatically actuated robot for forearm/wrist rehabilitation after stroke. *Annual International Conference of the IEEE Engineering in Medicine and Biology Society. IEEE Engineering in Medicine and Biology Society. Annual International Conference*, *2011*, 1579–1582. <https://doi.org/10.1109/IEMBS.2011.6090459>（The reason for exclusion: Irrelevant study outcome）
99. Lora-Millan, J. S., Sanchez-Cuesta, F. J., Romero, J. P., Moreno, J. C., & Rocon, E. (2022). A unilateral robotic knee exoskeleton to assess the role of natural gait assistance in hemiparetic patients. *Journal of neuroengineering and rehabilitation*, *19*(1), 109. <https://doi.org/10.1186/s12984-022-01088-2>（The reason for exclusion: Irrelevant study outcome）
100. Hu, X., Tong, K. Y., Song, R., Tsang, V. S., Leung, P. O., & Li, L. (2007). Variation of muscle coactivation patterns in chronic stroke during robot-assisted elbow training. *Archives of physical medicine and rehabilitation*, *88*(8), 1022–1029. <https://doi.org/10.1016/j.apmr.2007.05.006>（The reason for exclusion: without relevant date）
101. Germanotta, M., Cortellini, L., Insalaco, S., & Aprile, I. (2023). Effects of Upper Limb Robot-Assisted Rehabilitation Compared with Conventional Therapy in Patients with Stroke: Preliminary Results on a Daily Task Assessed Using Motion Analysis. *Sensors (Basel, Switzerland)*, *23*(6), 3089. <https://doi.org/10.3390/s23063089>（The reason for exclusion: without relevant date）
102. Piovesan, D., Morasso, P., Giannoni, P., & Casadio, M. (2013). Arm stiffness during assisted movement after stroke: the influence of visual feedback and training. *IEEE transactions on neural systems and rehabilitation engineering : a publication of the IEEE Engineering in Medicine and Biology Society*, *21*(3), 454–465. <https://doi.org/10.1109/TNSRE.2012.2226915>（The reason for exclusion: Non-RCTs）
103. Dovat, L., Lambercy, O., Gassert, R., Maeder, T., Milner, T., Leong, T. C., & Burdet, E. (2008). HandCARE: a cable-actuated rehabilitation system to train hand function after stroke. *IEEE transactions on neural systems and rehabilitation engineering : a publication of the IEEE Engineering in Medicine and Biology Society*, *16*(6), 582–591. <https://doi.org/10.1109/TNSRE.2008.2010347>（The reason for exclusion: Non robot-assisted task-oriented training
104. Abdullah, H. A., Tarry, C., Lambert, C., Barreca, S., & Allen, B. O. (2011). Results of clinicians using a therapeutic robotic system in an inpatient stroke rehabilitation unit. *Journal of neuroengineering and rehabilitation*, *8*, 50. <https://doi.org/10.1186/1743-0003-8-50>（The reason for exclusion: Non robot-assisted task-oriented training
105. Kim, H., Miller, L. M., Fedulow, I., Simkins, M., Abrams, G. M., Byl, N., & Rosen, J. (2013). Kinematic data analysis for post-stroke patients following bilateral versus unilateral rehabilitation with an upper limb wearable robotic system. *IEEE transactions on neural systems and rehabilitation engineering : a publication of the IEEE Engineering in Medicine and Biology Society*, *21*(2), 153–164. <https://doi.org/10.1109/TNSRE.2012.2207462>（The reason for exclusion: Non robot-assisted task-oriented training
106. Lum, P. S., Burgar, C. G., Van der Loos, M., Shor, P. C., Majmundar, M., & Yap, R. (2006). MIME robotic device for upper-limb neurorehabilitation in subacute stroke subjects: A follow-up study. *Journal of rehabilitation research and development*, *43*(5), 631–642. <https://doi.org/10.1682/jrrd.2005.02.0044>（The reason for exclusion: Non robot-assisted task-oriented training
107. Fisher, S., Lucas, L., & Thrasher, T. A. (2011). Robot-assisted gait training for patients with hemiparesis due to stroke. *Topics in stroke rehabilitation*, *18*(3), 269–276. <https://doi.org/10.1310/tsr1803-269>（The reason for exclusion: Non robot-assisted task-oriented training
108. Şenocak, E., Korkut, E., Aktürk, A., & Ozer, A. Y. (2023). Is the robotic rehabilitation that is added to intensive body rehabilitation effective for maximization of upper extremity motor recovery following a stroke? A randomized controlled study. *Neurological sciences : official journal of the Italian Neurological Society and of the Italian Society of Clinical Neurophysiology*, *44*(8), 2835–2843. <https://doi.org/10.1007/s10072-023-06739-3>（The reason for exclusion: Non robot-assisted task-oriented training
109. Hu, X. L., Song, R., Tong, K. Y., Tsang, S. F., Leung, O. Y., & Li, L. (2006). Coactivations of elbow and shoulder muscles in hemiplegic persons with chronic stroke during robot-assisted training. *Conference proceedings : ... Annual International Conference of the IEEE Engineering in Medicine and Biology Society. IEEE Engineering in Medicine and Biology Society. Annual Conference*, *2006*, 4933–4935. <https://doi.org/10.1109/IEMBS.2006.259575>（The reason for exclusion: Non robot-assisted task-oriented training
110. Piovesan, D., Casadio, M., Mussa-Ivaldi, F. A., & Morasso, P. G. (2011). Multijoint arm stiffness during movements following stroke: implications for robot therapy. *IEEE ... International Conference on Rehabilitation Robotics : [proceedings]*, *2011*, 5975372. <https://doi.org/10.1109/ICORR.2011.5975372>（The reason for exclusion: duplicate record）
111. Várkuti, B., Guan, C., Pan, Y., Phua, K. S., Ang, K. K., Kuah, C. W., Chua, K., Ang, B. T., Birbaumer, N., & Sitaram, R. (2013). Resting state changes in functional connectivity correlate with movement recovery for BCI and robot-assisted upper-extremity training after stroke. *Neurorehabilitation and neural repair*, *27*(1), 53–62. <https://doi.org/10.1177/1545968312445910>（The reason for exclusion: duplicate record）
[truncated: 180,946 more chars]
