# Supplementary material for: Effectiveness of robot-assisted task-oriented training intervention for upper limb and daily living skills in stroke patients: A meta-analysis
Source: PLoS One. 2025 Jan 3;20(1):e0316633. doi: 10.1371/journal.pone.0316633 (PMC11698451; doi:10.1371/journal.pone.0316633)
Supplement: S1 File — (DOCX) [file pone.0316633.s005.docx]

**S3 Search strategy for PubMed**

#1 ("Stroke"[Majr]) OR (((((cerebral stroke) OR (cerebralvascular accident)) OR (CVA)) OR (hemiplegia)) OR (cerebrovascular stroke))

#2 ("Robotics"[Majr]) OR (((Assistive Robot) OR (Soft Robotics)) OR (End-effector))

#3 ("Hand"[Majr]) OR ((((((((hand function) OR (thumb)) OR (fingers)) OR (metacarpus)) OR (upper limb)) OR (FMA-UE)) OR (MBI)) OR (Modified Barthel Index))

#4 ("Randomized Controlled Trial" [Publication Type]) OR ((RCT) OR (random allocation))

# 5（#1 AND #2 AND #3 AND #4）
